# Rapid determination of quaternary protein structures in complex biological samples

**DOI:** 10.1038/s41467-018-07986-1

**Published:** 2019-01-14

**Authors:** Simon Hauri, Hamed Khakzad, Lotta Happonen, Johan Teleman, Johan Malmström, Lars Malmström

**Affiliations:** 1 Division of Infection Medicine, Department of Clinical Sciences Lund, Faculty of Medicine, Lund University, Klinikgatan 32, SE-22184 Lund, Sweden; 2 S3IT, University of Zurich, Winterthurerstrasse 190, CH-8057 Zurich, Switzerland; 3 Institute for Computational Science, University of Zurich, Winterthurerstrasse 190, CH-8057 Zurich, Switzerland

## Abstract

The understanding of complex biological systems is still hampered by limited knowledge of biologically relevant quaternary protein structures. Here, we demonstrate quaternary structure determination in biological samples using a combination of chemical cross-linking, high-resolution mass spectrometry and high-accuracy protein structure modeling. This approach, termed targeted cross-linking mass spectrometry (TX-MS), relies on computational structural models to score sets of targeted cross-linked peptide signals acquired using a combination of mass spectrometry acquisition techniques. We demonstrate the utility of TX-MS by creating a high-resolution quaternary model of a 1.8 MDa protein complex composed of a pathogen surface protein and ten human plasma proteins. The model is based on a dense network of cross-link distance constraints obtained directly in a mixture of human plasma and live bacteria. These results demonstrate that TX-MS can increase the applicability of flexible backbone docking algorithms to large protein complexes by providing rich cross-link distance information from complex biological samples.

## Abstract (web summary)

Protein structure determination in complex biological samples is still challenging. Here, the authors develop a computational modeling-guided cross-linking mass spectrometry method, obtaining a high-resolution model of a 1.8 MDa protein assembly from cross-links detected in a mixture of human plasma and bacteria.

## Introduction

Proteomes are organized into functional modules that act in concert to dictate cellular responses^1^. Dynamic networks of physical protein–protein interactions assemble functionally related proteins into functional modules and represent one of the fundamental principles cells use to re-organize dynamically yet remain functional. Defining the quaternary structure of functional modules at a proteome-wide scale under close-to in vivo conditions has the potential to increase the understanding of molecular processes in health and disease but has so far remained elusive. Most high-resolution methods such as X-ray crystallography, nuclear magnetic resonance, and electron cryo-microscopy require purified proteins, whereas affinity purification-mass spectrometry (AP-MS) provides information on protein connectivity in complex samples but suffer from limited structural information. In contrast, chemical cross-linking MS (XL-MS) can support structural modeling by providing evidence that two proteins are interacting by covalently linking together MS-detectable amino acid pairs^2–4^. The length of the cross-linking reagent constrains the distance between the linked residues and can together with observed or predicted protein structures provide high-accuracy structural information of proteins or protein interaction networks.

XL-MS is, however, associated with several technical challenges that limit the confident identification of cross-linked distance constraints and impedes the routine deployment of this method at a system-wide scale. The prevailing limitations in the XL-MS workflow stem from the quadratic expansion of the computational search space and the unequal fragmentation efficiency of two cross-linked peptides. The consequence of these limits is that only one of the two peptides is directly observable in the data and this, given a large search space, is not sufficient to unambiguously identify the second peptide based on just the cross-linked peptides precursor mass. Recent computational solutions and combination of MS acquisition types have partly resolved these problems demonstrating the identification of cross-links from endogenous protein complexes to provide structural information^4^. Still, previous work suffers, in general, from the identification of sparse networks of cross-linked distance constraints, where a given protein–protein interaction is often supported by one or a few data points. On the other hand, dense networks of distance constraints can strongly enhance computational protein docking and protein structure modeling to determine quaternary protein structures at high accuracy. This is of particular relevance for quaternary structures with high molecular weights, as the required density of the network is proportional to the molecular weight and number of cross-linkable sites of the protein complexes.

Computational protein docking tools such as Rosetta have recently seen improvements^5^ but are generally applied to small quaternary structures supported by existing experimental structures of the individual proteins for which tertiary structures remain similar in the bound and unbound form. This limitation stems from the large search space that, in its extreme, includes all the rotational and translational degrees of freedom and the many degrees of freedom that comes from flexible backbone and amino acid side chain modeling. Rosetta is using a complex energy function, called the Rosetta All-Atom Energy Function (REF)^6^, to estimate the fitness of the protein structure at each step of the folding trajectory. To speed up the calculations, Rosetta initially relies on a sparse representation of the structure by representing each amino acid as a centroid. Centroiding necessitates the use of a REF adapted to centroid-based representation as the needed details to compute the more physically realistic REF are lacking. In the later stages of the folding trajectories, Rosetta switches to a more realistic REF. Some folding trajectories get caught in local energy minima that are significantly different from the global energy minimum. Rosetta utilizes a powerful technique to mitigate this issue by simulating many folding trajectories and using population statistics to select the final models. A large search space requires more folding trajectories and therefore also more models. Experimental data constraints can be incorporated into the modeling to both to provide better discrimination between two models deemed equally fit by REF and to reduce the search space by excluding vast regions of the search space that are incompatible with the observed data. By using computational docking tools to construct a compendium of quaternary structural models, it becomes feasible to interrogate which of the models, with similar energy scores, are correct by using experimentally derived distance constraints.

In an effort to enable the identification of dense networks of distance constraints and consequently the modeling of large quaternary protein structures, we proposed a technical concept that we called TX-MS (targeted chemical cross-linking MS). TX-MS relies on a compendium of computationally predicted quaternary structure models to guide the MS analysis of cross-linked peptides using a combination of MS acquisition techniques to discriminate between the correct and incorrect predicted models. In this work, we used computational structural models to guide and score sets of targeted cross-link signals derived from a combination of MS acquisition techniques. The experimentally derived distance constraints are further used to improve the modeling of high-resolution quaternary structures. This represents a departure from the de novo identification of individual cross-linked peptides removing the exponential increase in the number of potential lysine–lysine pairs that typically need to be considered. Importantly, TX-MS also mitigates the unequal fragmentation issue (only one of the cross-linked peptides are represented in the data), as data from the different MS acquisition techniques over the same interface strongly corroborate each other. We used TX-MS to support the generation of dense networks of distance constraints for a protein complex directly in biological mixtures using a large host–pathogen functional module formed between the important human pathogen *Streptococcus pyogenes* and human plasma proteins as a model system. The results show that TX-MS can provide dense networks of cross-linked distance constraints that enable quaternary protein structure modeling using data acquired in biological samples.

## Results

### Structure modeling guided by MS data

**Fig. 1:**
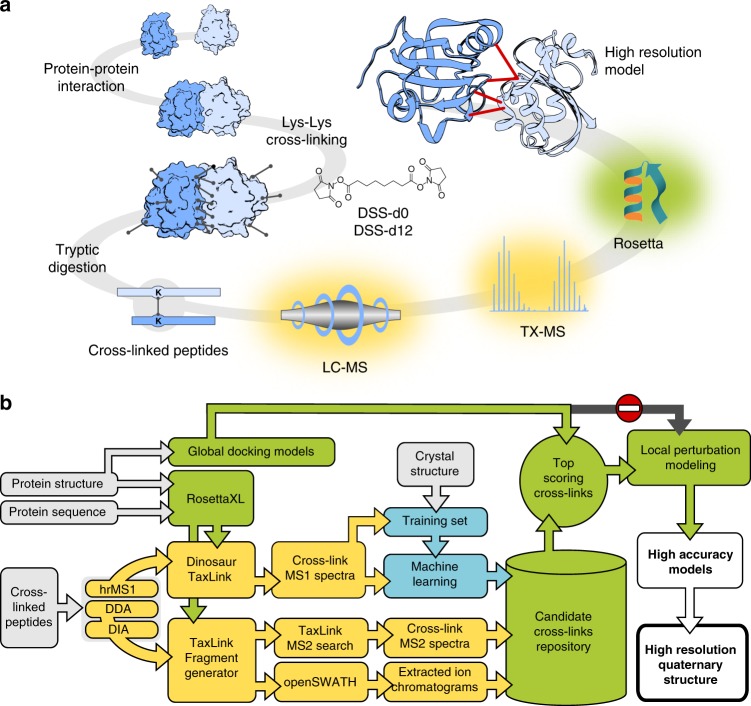
The principle of targeted cross-linking mass spectrometry (TX-MS). **a** Interacting proteins were chemically cross-linked with heavy/light DSS and digested using trypsin. Cross-linked peptide signals were targeted and extracted from LC-MS data based on theoretical cross-link assays derived from the protein sequence. The identified candidate cross-linked peptides were used to guide molecular docking of crystal structures of the targeted proteins to obtain high-accuracy models of the protein–protein interaction. **b** Schematic workflow of TX-MS. Assays for targeted cross-linked peptides were calculated from either the protein sequence or structure. Cross-linked peptides were analyzed from LC-MS data using different acquisition modes. For hrMS1-based cross-link detection, the theoretical assays were used to extract ion chromatograms from high-resolution hrMS1 spectra. A subset was utilized to train a machine to differentiate true from false-positive cross-linked peptides as defined by experimental protein structures (see Supplementary Note 3 for details on how the machine was trained). The trained machine was used to evaluate all MS1-derived cross-links. Top scoring results were used to select the best-supported models for subsequent local high-resolution quaternary structure modeling, yielding high-accuracy models. To supplement the hrMS1 results, we acquired XL peptide data with the two orthogonal MS2 methods DDA-MS and DIA-MS. Custom software processed MS2 by extracting signals from fragment ions derived from the predicted cross-linked peptides

**Fig. 2:**
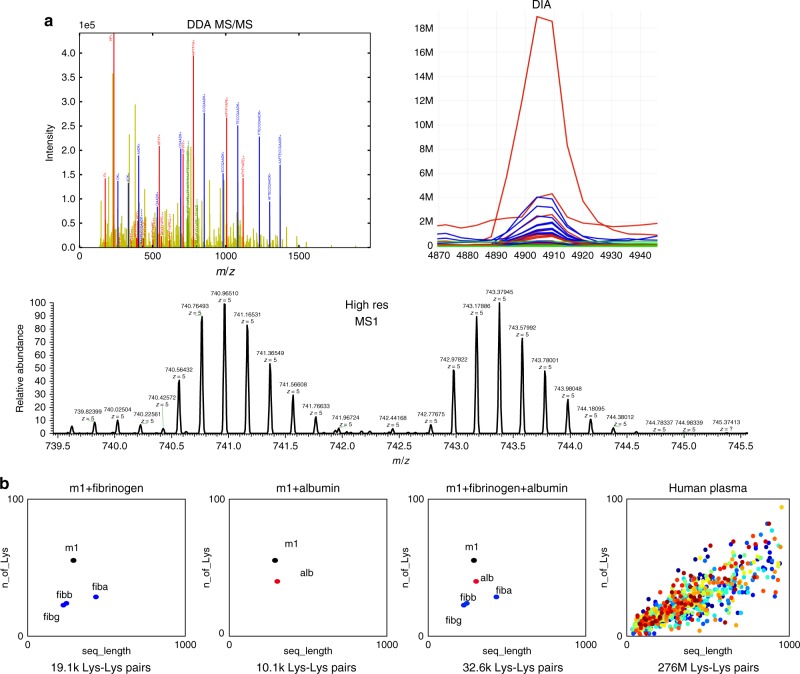
Example MS data. **a** Example data for the three experimental techniques. DDA-MS isolates a single precursor and fragments it; the fragments visible in the acquired spectra can be associated with fragments from either peptide or in heavy and light form for fragments that contain the cross-linker and parts of both peptides. DIA-MS is similar to DDA-MS but with important differences. There is no isolation of the precursor. Instead, data are acquired at high temporal sampling rates, providing the opportunity to compare elution profiles for the fragments. In hrMS1, the precursor is measured at high resolution resulting in two isotope envelopes, one for the light and one for the heavy cross-linker (**b**). The scatter plots show the number of lysines as a function of protein length of the protein identified in the sample mixture. The number of theoretical lysine–lysine pairs in each sample increases exponentially with sample complexity, from about 10,000 in the simplest mixture to >276 million in the most complex

In TX-MS, proteins or proteomes are chemically cross-linked in a dose-dependent manner and subjected to MS analysis using several MS acquisition techniques such as data-dependent acquisition (DDA), data-independent acquisition (DIA), and high-resolution MS1 (hrMS1; *R* = 280,000). The proteins of interest are subsequently modeled to create tertiary structures^7^ that are docked^8^ to produce a compendium of possible quaternary structure models (Fig. 1a). Each quaternary model is ranked using the hrMS1 data by (i) calculating the cross-linked peptide mass over charge ratio (*m*/*z*) resulting from proteolytic digestion for all theoretical cross-linkable amino acid pairs shorter than a given length cutoff and (ii) extracting and quantifying targeted hrMS1 signal for each calculated *m*/*z* and to select the quaternary models that are best explained by the acquired MS data. The top-scoring models then go through a second round of high-resolution flexible backbone protein docking^8^, and the resulting model compendium is used to define all the possible supported distance constraints. The set of distance constraints is used by a software tool to compute the theoretical cross-linked peptide and fragment-ion masses and to find evidence for them in DDA-MS and DIA-MS (Figs. 1b, 2a) in a targeted data analysis fashion as follows:

(1) In DDA-MS, we search the MS data for all theoretical *m*/*z* values for fragments of cross-linked peptides across fragment-ion spectra (MS2) and rank by cross-linked peptide sequence coverage. The candidate spectra are filtered for the correct precursor isolation mass at 0.01 Da mass error tolerance and all MS2 spectra containing cross-linked and conventional peptide fragments from the cross-linked peptide sequences are considered as top candidates (Fig. 2a).

(2) In DIA-MS, the theoretical fragments are searched using a modified openSWATH^9^ workflow to extract chromatograms for every fragment in a targeted MS data analysis fashion. The modified openSWATH software identifies high-confident peak groups from co-eluting fragments of the cross-linked peptide pairs and the isotope-labeled cross-linked fragment ions to provide the strongest evidence of occurrence in the data (Fig. 2a).

### High-resolution protein–protein docking

The final result of this analysis is a set of lysine–lysine pairs defined by a high-resolution quaternary model compendium; each lysine–lysine pair is associated with MS evidence from the three separate MS acquisition techniques, acquired from samples with an increasing concentration of disuccinimidyl suberate (DSS). Together with the negative control (no DSS added), we define a final list of putative lysine–lysine pairs and use this list to filter out the final model from the structure compendium. As a consequence, we extend the applicability of protein docking algorithms to take models as input instead of crystal structures by reducing the protein–protein docking search space drastically. The best models are picked by combing MS compatibility and REF ^6^, a structural fitness score based on a statistical energy model^10^ and a normal distribution-based scoring of the cross-linked length. The combination of protein structure modeling and MS is powerful because each method’s strength complements a relative weakness of the other. It also marks the departure from a discovery-driven workflow to an inherently hypothesis-driven method that lends itself to explore interactions of a protein of interest in a targeted approach that is more sensitive and that results in larger number of constraints, translating into higher-accuracy models with more experimental support. The complete TX-MS workflow is supported by several open source software tools largely written in python with a few tools developed in Scala and C++ and provided under permissive software licenses (Fig. 1b and Supplementary Notes 1–5, Supplementary Figures 1–7, Supplementary Tables 1–4).

### Experimental design

To demonstrate that TX-MS can generate high-density networks of cross-linked peptide distance constraints, we applied the method to investigate the quaternary structure of a host–pathogen complex of unknown structure using the important human pathogen *S. pyogenes* as a model system. Protein interactions between pathogens and their hosts are difficult to study, yet host–pathogen interaction studies are becoming increasingly important, as such studies can be used to support the design of vaccines. *S. pyogenes* is a Gram-positive bacterium that causes diverse clinical manifestations ranging from mild infections such as tonsillitis to life-threating systemic diseases, like sepsis and meningitis^11^. The bacterium produces >150 surface proteins^12^ forming a wide array of protein interactions with 290 potentially interacting human proteins^13^, including fibrinogen and serum albumin^14,15^. The dominating *S. pyogenes* surface protein in these protein interaction networks is the family of M proteins (referred to as the M- or serotype-specific M1 protein below) used to classify the >220 known serotypes. The M proteins can be divided into different repeat regions that can be present in a variable number of copies and combinations denoted as hypervariable (HVR) A-, B-, and C- repeat regions as recently reviewed^16^. It is only partially clarified how the repeat regions are capable of maintaining dynamic interactions with a large number of human proteins to form a large protein complex of unknown structure, yet escape immunity through antigen variability^17^. To determine the quaternary structure of this protein complex, we produced three sets of low-complexity samples and one set of samples with a highly complex mixture of a complete bacterial proteome and human plasma proteins (Fig. 2b). Each sample was cross-linked with increasing amounts of a heavy/light cross-linker (DSS-H12/D12; DSS) generating a total of 102 MS experiments using the three data-acquisition techniques (hrMS1, DDA, and DIA) (Supplementary Table 4).

### M1 protein interacts with fibrinogen and albumin

**Fig. 3:**
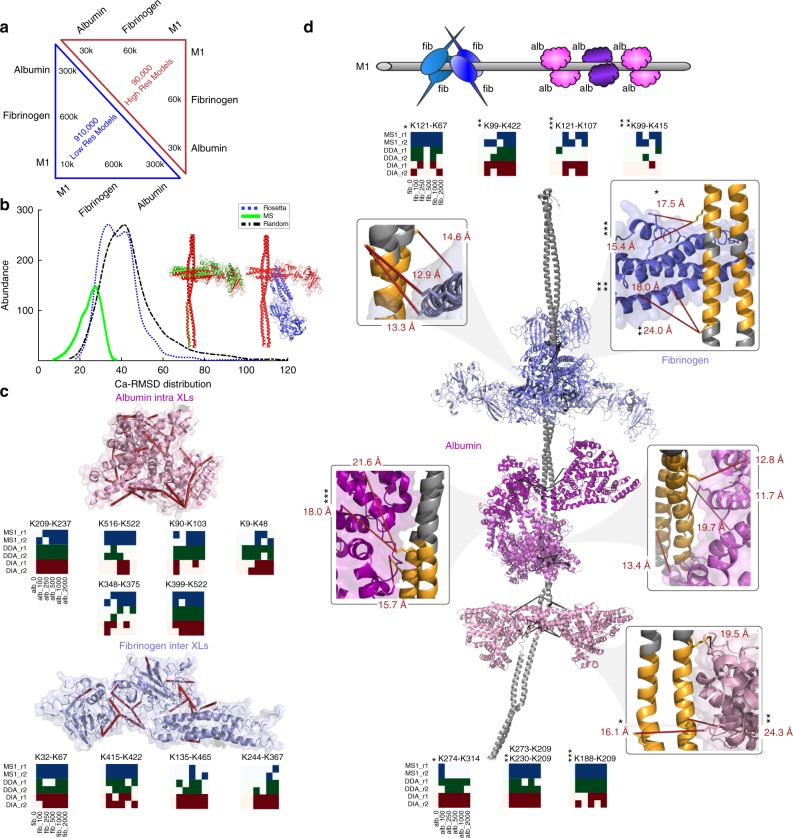
Human fibrinogen and albumin bind to the streptococcal M1 protein at several distinct interfaces. **a** A total of 900,000 low-resolution models were created by docking the M1 protein with fibrinogen and albumin. A further 90,000 high-resolution docking models were created for the highest-scoring low-resolution models. **b** Impact on hrMS1 data on molecular docking between fibrinogen and the M1 protein. The inclusion of MS1 data drastically improved the RMSD, especially models close to the native conformation <5 Å. **b** Structural representation of models selected by Rosetta score (blue) and MS1 score (green) in comparison to the reference (red). **c** High-resolution model of the streptococcal M1 protein bound to four fibrinogen molecules and six molecules of albumin at five distinct, defined binding interfaces. All depicted cross-links are <30 Å distance between the lysine residues. The heatmap-like representations show the number of observations in the three different acquisition modes. Only cross-links measured using more than one acquisition method is represented. **d** A schematic representation of the four fibrinogen and six albumin molecules bound to the M1 protein

The three low-complexity samples, consisting of mixtures of the M1 protein, serum albumin, and fibrinogen (Fig. 2b), were designed to first provide a positive control, as a crystal structure between the M1 protein and human fibrinogen exists (PDB: 2XNX), and second, to characterize the interaction between the M1 protein and human serum albumin, an important protein interaction where no experimental structure exists. Fibrinogen binding prevents phagocytosis^18^, and the interaction with human fibrinogen has been studied in detail by determining the X-ray structure for fragments of fibrinogen binding to the M1 protein^19^. Fibrinogen binds to the two B-repeats on the M1 protein, with slight differences in amino acid composition. As only a small segment of the M1 protein has an experimental structure (PDB: 2XNX), we created a full-length M1 protein model using the RosettaCM multi-template protocol as described in detail in Supplementary Note 6, Supplementary Figures 8–10, and Supplementary Tables 5-8. To ensure sufficient coverage of the large search space between these molecules, we constructed 600,000 fibrinogen and full-length M1 protein quaternary structure models (Fig. 3a). The molecular docking on its own was, as expected owing to M1 protein full-length comparative model, unable to predict the correct quaternary conformation with a root-mean-square deviation (RMSD) for α-carbons similar to random sampling when compared to the 2XNX reference crystal structure (Fig. 3b). To enhance the quality of the structural models, we applied TX-MS, using the structural compendium to guide the MS data analysis. With TX-MS, we collectively identified 67 intra and 27 inter high-confidence XL peptides in the low-complexity samples supported by up to three different MS acquisition methods as indicated by the heatmaps in Fig. 3. Scoring the 600,000 molecular docking models using the TX-MS-derived distance constraints together with 60,000 high-resolution models supported a model with an RMSD <2.2 Å. The new RMSD is close to the previously determined crystal structure and demonstrates the accuracy of TX-MS (Fig. 3b). Importantly the model correctly reveals that there are two distinct binding sites that both correspond to the M1 protein B-repeats^19^ (Fig. 3d).

### Protein complex modeling

For human serum albumin, the combined output from the 300,000 low-resolution and 30,000 high-resolution quaternary structure models and 10 TX-MS identified inter-protein XL peptides supports three separate models where serum albumin binds to the three conserved C-repeats of the M1 protein (Fig. 3d)^15,20^. Based on these models, it was possible to confidently locate the binding to the three separate binding sites, as the small variation in the sequence within the C-repeat regions of the M1 protein generates different cross-linked peptides when cross-linking the M1 protein to albumin. The results indicate that the conserved C regions represent duplications of albumin-binding M1 sequences to increase the number of bound albumins as a source for fatty acids^21^. Interestingly, the T cell- and B cell-protective C-terminal vaccine epitope (StreptInCor)^22^ harbors the albumin-binding site, which likely results in partial masking of this immunogenic epitope region in the presence of albumin as previously shown for fibrinogen^18^.

The initial number of docking models is highly system specific, where small proteins with experimentally determined structures need fewer models compared to larger proteins modeled using evolutionary distant protein structure templates. The number of models in the second step is also system dependent. As we are allowing for flexible backbones in this step, flexible proteins need more extensive sampling. As the search is constrained to only deviate within a given limit, the size of the proteins is less of a factor. More complex quaternary structures such as obligate hetero-trimers further increase the complexity of the docking steps, requiring more computational effort and more models.

In the next step, we used the human proteins with known structure as an internal control to indicate the quality of TX-MS. In total, we identified 155 intramolecular XL-peptides mapping to fibrinogen and albumin based on their crystal structures (2XNX and 1E7I, respectively; Fig. 3c). Out of these 150 (97%), Cα–Cα distances were less than the 30 Å length cutoff. Eighty-seven of the cross-links were intra albumin cross-links, whereas 41 were intra cross-links within the α–α, β–β, and γ–γ fibrinogen chains 26 between the αβγ fibrinogen chains. The supporting MS data for each cross-link of the purified M1 protein, albumin, and fibrinogen are listed in Supplementary Data 1.

### Determining quaternary structures in complex samples

**Fig. 4:**
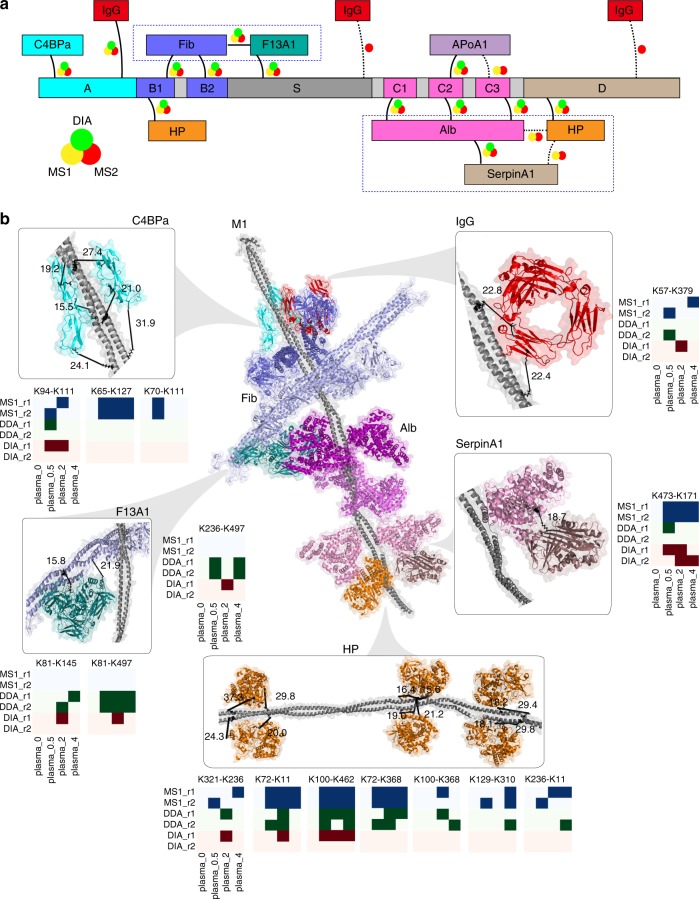
Determining the quaternary structure of M1–human plasma protein complex. The 1.8-MDa M1 protein–human plasma protein complex model created using TX-MS with measurements directly from complex biological samples. *S. pyogenes* cells were exposed to human plasma and thoroughly washed. After application of DSS, proteins were digested with trypsin and analyzed with TX-MS. **a** Schematic binding sites for the M1 protein binders after plasma adsorption on the bacterial surface. Fibrinogen and albumin were confirmed, and direct (IgG, haptoglobin [HP]) and indirect (alpha-1-antitrypsin [SerpinA1] and coagulation factor XIII A [F13A]) were identified. **b** Updated binding interface model of the M1 protein after plasma adsorption on the bacterial surface. Zoomed views show all the observed cross-links, and the heatmap-like thumbnails represent observation frequencies for the different acquisition methods. The three pairs of albumin molecules bind to the three M1 protein C-repeats; SerpinA1 is bound to Alb, and we detected no evidence for direct interaction of that with M1. The fibrinogen heterotrimers bind to the B-repeats. F13A1 is cross-linked to both fibrinogen and the M1 protein. HP binds to a part of the B1-repeat. The C4b-binding protein (C4BPa) and IgG bind to the A-region of the M1 protein, also known as the hypervariable region, show possible binding competition in this region. Finally, we detected three separate binding sites of IgG, but for two of them, we have only MS2 support. The model can be downloaded as a PDB file or a Pymol session

In contrast to the synthetic samples above, we analyzed high-complexity samples to determine the quaternary structure of the whole M1 protein–human plasma protein network and so far uncharacterized interactions between M1 protein-bound plasma proteins. In this experiment, intact bacteria were incubated in plasma and surface interacting human proteins isolated through centrifugation of the intact bacteria^23^ followed by the addition of a dilution series of heavy/light cross-linker. The cross-linked *S. pyogenes* proteome and the bound human proteins were subsequently trypsin digested to generate highly complex samples with >276 million theoretical lysine–lysine pairs (Fig. 2a). However, the number of lysine–lysine pairs that are considered by TX-MS is orders of magnitudes lower as the vast majority of pairs are not supported by the structure models selected after the first docking round. In previous work, we have identified >20 human proteins that interact with the M1 protein using AP-MS that likely represents a combination of direct and indirect protein bindings to the M1 protein. To determine the quaternary structure of the M1–human plasma protein complex, we constructed in total 1,410,000 models for 10 of the M1 interactors in addition to the models previously generated for serum albumin and fibrinogen (Supplementary Tables 7 and 8). These models were used to guide the MS data acquisition as outlined above. In addition to serum albumin and fibrinogen, TX-MS identified cross-links between the M1 protein and four of the tested proteins, C4b-binding protein (C4BP), the immunoglobulin IgG, apolipoprotein A1, and haptoglobin (Fig. 4a). The identified cross-linked distance constraints indicate that the heavy/light cross-linker can stabilize potentially weak or transient interactions as in the case of C4BP, which was supported by three unique cross-links per interaction and was previously shown to predominately bind other types of M proteins^24,25^ and the M-like protein H^26^. To verify the C4BP–M1 protein interaction, we carried out an additional TX-MS experiment using commercial C4BP. The experiment resulted in six additional cross-links that all supported the model created using only the data from the original experiment. Results and experimental details are described in Supplementary Note 8, Supplementary Figures 11–14, and Supplementary Table 9. Interestingly, we also identified a total of 48 inter cross-links between interacting human proteins. Twenty-one of these are found between the coagulation factor XIIIA bound to fibrinogen (14 cross-links; 8, 4, and 2 to α, β, and γ chains, respectively) and alpha-1-antitrypsin (SerpinA1) bound to albumin (7 cross-links) demonstrating a large quaternary structure of both host–pathogen and host–host interactions. The remaining 27 inter-protein human cross-links are found in Supplementary Table 8. Collectively across all experiments, we identified 204 distinct inter-protein cross-links supported by up to the three different MS acquisition methods, with an average of 13.6 constraints per interface. The number of identified inter-protein cross-links is on par with the number of identified cross-links from large-scale XL-MS analysis of full cell lysates. However, in contrast to previous work, the identified cross-links are not distributed across several functional modules but are rather confined within one protein complex, considerably increasing the number of cross-link observations per protein-binding interface.

To demonstrate that dense network of distance constraints benefits the molecular modeling, we used all the generated XL distance constraint for local perturbation modeling to create a comprehensive binding model of the M1 protein on the bacterial surface (Fig. 4b). This M1-binding protein module contains ten human plasma proteins and has an estimated size of 1.8 MDa. The model reveals a highly dense and organized structure where the interacting proteins are distributed along the M1 protein. The modeled structure reveals that only a minor part of the M1 protein is surfaced exposed when bound to the human plasma proteins. Collectively, these results explain how the repeat regions are efficiently used to line up several plasma proteins along the M1 protein to prevent phagocytosis, inhibiting complement activation and securing nutrients for the bacterium and at the same time masking conserved and vulnerable surface epitopes in the binding interfaces with human proteins. We anticipate that this model will contribute to the understanding of the relationship between the molecular organization of the M protein family and the interaction with human host proteins, which may have implications for the design of vaccines for *S. pyogenes*.

## Discussion

In this work, we demonstrate a targeted cross-link MS strategy to create a high-accuracy model of a 1.8-MDa multi-species protein complex consisting of 11 unique polypeptide chains. We identified an average of 13.6 distance constraints per interaction, thereby demonstrating that the method described here, TX-MS, can model large quaternary protein structures by extracting a high-density network of distance constraints directly from complex, unfractionated, biological samples. The deep integration of XL-MS and protein structure modeling enables us to overcome limitations associated with each method; a scoring function allows us to model larger quaternary structures, also with limited access to experimental tertiary structures. The generated structural models enable the analysis of sets of cross-linked peptides, thereby reducing the necessity of confidently identifying both peptides in a single MS spectrum.

The directed MS data analysis guided by a compendium of computationally predicted quaternary structure models enables the departure from the traditional de novo identification of individual cross-links; instead, TX-MS uses hrMS1 data to score low-resolution protein–protein docking models, then uses the best-supported models to identify multiple highly informative cross-links in a targeted data analysis approach. The final models are created through a high-resolution flexible backbone docking protocol that uses both models and all identified distance constraints as input. The targeted data analysis approach allowed us to integrate data from three separate MS acquisition methods. The use of three different MS acquisition protocols is advantageous as the methods generated similar but not perfectly overlapping sets of identified cross-links allowing us to discriminate between correct and incorrect models in a powerful way. In this work, we required each interface to be supported by three or more cross-links and that at least one of these cross-links could be identified using all three acquisition methods. All reported interfaces had significantly more support than this strict requirement and the on average 13.6 identified cross-links per interface demonstrates the sensitivity of the method. True negative protein–protein interactions, such as M1 protein–serpinA and M1 protein–C1Q, were rejected by TX-MS as they fell below our inclusion criteria, allowing us to correctly model these proteins as indirect interactors of the M1 protein. The complete TX-MS workflow is supported by several open source software tools that are found in a pre-installed software container designed to make software portable by installing all needed software and their dependencies in a file that can be easily shared (Supplementary Note 7).

The modeling of the quaternary structure of the host–pathogen protein complex was, in the final steps, made using high-resolution flexible backbone docking protocols using low-resolution models supported by the experimentally derived distance constraints. In this case, access to high-resolution atomic structures or Rosetta-generated macromolecular models shows that TX-MS is capable of generating quaternary structures of large protein complexes in noisy biological backgrounds without the need for monodispersed stable protein complexes for co-crystallization or cryo-EM studies. Furthermore, the confident identification of cross-links provides the computational coordinates representing a given cross-linked peptide pair. These computational coordinates can be iteratively improved and incorporated in MS assay libraries and re-used in future experiments. We anticipate that high-confident libraries of MS assays for cross-linked peptides distance constraints can, in future work, be used to determine dynamic changes of the quaternary structures in biological samples after, for example, chemical and genetic perturbation. Ultimately, the applicability of the method is restricted by the ability to model the quaternary structure in Rosetta, which in turn is restricted by the availability of experimental tertiary structures of the monomers, docking protocols, energy functions, and computational power; as all these are continuously improved, the applicability of TX-MS is increasing rapidly. In conclusion, the flexibility and generic nature of TX-MS enable the workflow to be extended to model the quaternary structure of other types of protein complexes and may represent a valuable tool to improve our understanding of protein biochemistry and protein complex formation in general.

## Methods

### Protein cloning and expression

The *S. pyogenes* open reading frame (amino acids 42–484) encoding for the M1 protein (UniProt ID: Q99XV0, emm1) was cloned at the Lund Protein Production Platform (LP3) (Lund, Sweden). The encoding sequence was ordered as a synthetic construct from Genscript (NJ, USA), cloned into the EcoRV site of pUC57, and subsequently subcloned into a pNIC28-Bsa4-based vector incorporating a tandem affinity purification tag (histidine-hemagglutinin-StrepII-tobacco etch virus protease recognition site) at the C-terminus of the construct. The M1 protein was expressed in Luria-Bertani Broth (Difco) at 37 °C in *E. coli* BL21 (DE3) cells. Protein expression was induced with 1 mM IPTG at OD_600_ 0.5–0.6. The M1 protein was purified from harvested cells using an in-house prepared fibrinogen-column^15^. Pooled fractions from the fibrinogen column were dialyzed against 1× phosphate-buffered saline (PBS) pH 7.4, and loaded on Ni-coupled Imac Sepharose 6 Fast Flow (GE Healthcare). The column was washed with 20 mM imidazole in 1× PBS pH 7.4, and bound protein was eluted with 500 mM imidazole in 1× PBS pH 7.4 using gravity flow. Pooled fractions from the Ni-column were buffer exchanged into 1× PBS pH 7.4 and concentrated using Millipore Amicon 30 kDa molecular weight cutoff concentrators. Purified protein was stored at −80 °C until usage.

### Commercial proteins and human plasma

Albumin from human serum (A3782) and fibrinogen from human plasma (F4883) were obtained from Sigma. Pooled normal human plasma from healthy donors was purchased from Innovative Research (MI, USA).

### Cross-linking of purified proteins

Fifty micrograms of purified M1 protein was incubated with 66 μg of albumin (1:1 molar ratio) or 100 μg of fibrinogen (2:1 molar ratio due to an average of 50% impurities in the commercial fibrinogen) resuspended in 1× PBS pH 7.4 at 37 °C, 500 rpm, 30 min^2^. Alternatively, 50 μg of purified M1 protein was incubated together with 66 μg of albumin and 100 μg of fibrinogen at 37 °C, 500 rpm, 30 min. Heavy/light DSS (DSS-H12/D12, Creative Molecules Inc., https://www.creativemolecules.com) resuspended in dimethylformamide (DMF) was added to final concentrations of 0, 100, 250, 500, 1000, and 2000 μM and incubated for a further of 30 min at 37 °C, 900 rpm. The cross-linking reaction was quenched with a final concentration of 50 mM ammonium bicarbonate at 37 °C, 500 rpm, 15 min.

### Cross-linking of plasma adsorption samples

The *S. pyogenes* strain SF370, a clinical isolate of the M1 serotype, was grown at 37 °C and 5% CO_2_ from a single hemolytic colony to mid-exponential phase (OD_620nm_ ∼ 0.4) in Todd–Hewitt broth supplemented with 0.3% (w/v) yeast extract. The cells were harvested by centrifugation (3500 × *g*, 5 min), washed with HEPES-buffer, recentrifuged, and resuspended to an approximate concentration of 1 × 10^9^ colony-forming units/ml. Four hundred microliters of pooled normal human plasma was mixed with 100 μL of bacteria and incubated at 37 °C, 30 min, 500 rpm^13^. The bacteria with adsorbed plasma proteins were again harvested by centrifugation (5000 × *g*, 5 min) and washed three times with HEPES-buffer, with subsequent centrifugations. The bacteria with adsorbed plasma proteins were resuspended in HEPES buffer, and heavy/light DSS in DMF was added to final concentrations of 0, 500, 2000, and 4000 μM and incubated for 60 min at 37 °C, 900 rpm. The cross-linking reaction was quenched with a final concentration of 50 mM ammonium bicarbonate at 37 °C, 500 rpm, 30 min. The surface proteins with attached plasma proteins were digested off with 2 μg trypsin (Promega)^23^, prior to cell debris removal by centrifugation (1000 × *g*, 15 min), supernatant recovery, and heat inactivation of any remaining pathogenic bacteria (85 °C, 5 min) and finally sample preparation for MS.

### Sample preparation for mass spectrometry

Samples from cross-linking of purified proteins and cross-linking of plasma adsorption were denatured in 8 M urea–100 mM ammonium bicarbonate, and the cysteine bonds reduced with 5 mM *tris*(2-carboxyethyl)phosphine (37 °C, 30 min) and alkylated with 5 mM iodoacetamide (22 °C, 60 min). Samples were diluted with 100 mM ammonium bicarbonate to a final urea concentration of 1.5 M, and sequencing-grade lysyl endopeptidase (37 °C, 2 h) (Wako Chemicals) followed by trypsin (37 °C, 18 h) (Promega) was added for protein digestion. Digested samples were acidified with 10% formic acid to a pH of 3.0, and the peptides were subsequently purified with C18 reverse-phase spin columns according to the manufacturer’s instructions (Macrospin columns, Harvard Apparatus). Dried peptides were reconstituted in 2% acetonitrile and 0.2% formic acid prior to MS analyses.

### MS experiments

Modern MS-based proteomics relies on several data acquisition protocols, each with strengths and weaknesses. Here we rely on three different acquisition strategies; hrMS1, DDA (MS2), and DIA. We cover each method in more detail throughout supplementary notes, but in brief, MS1 measures the intact peptide ions at high resolution, both in the *m*/*z* and time dimensions. DDA does a quick scan of the eluting peptides and then uses a simple algorithm to select a few of the peptides to fragment and measure. In DIA, we fragment several peptides at once, thereby increasing the capacity, enabling us to comprehensively measure the resulting fragments at high time resolution; the penalty is highly complex and convoluted data that requires specialized software to analyze. Supplementary Table 4 contains the sample id of all experimental data (hrMS1, MS2-DDA, and DIA).

### Liquid chromatography-MS

All MS measurements were performed on a Q Exactive Plus (Thermo Scientific) connected to an EASY-nLC 1000 liquid chromatography system (Thermo Scientific). Peptides 1 µg were separated by C18 reverse-phase chromatography using a 25-cm EASY-Spray column (P/N: ES802, column temperature 45 °C) with a linear gradient from 5% to 35% acetonitrile in aqueous 0.1% formic acid at a flow rate of 300 nl/min for 60 min (DDA), 90 min (hrMS1), or 120 min (DIA). Column equilibration and sample loading were performed at 600 bar. Resolution (*R*) is defined at 200 *m*/*z*. For hrMS1, high-resolution MS scans (*R* = 280,000) were acquired using automatic gain control (AGC) was set to 1e6 and a fill time of 100 ms (MS). For DDA, the 15 most intense precursor ions of charges ≥2 from an MS1 scan (*R* = 70,000) were allowed to be fragmented and measured at *R* = 17,500. AGC was set to 1e6 for both MS and MS/MS with ion accumulation times of 100 ms (MS) and 60 ms (MS/MS). Precursor ions were fragmented using higher-energy dissociation (HCD) at a normalized collision energy of 30.

For DIA, one MS1 scan (*R* = 70,000; mass range from 400 to 1200 *m*/*z*) was followed by 32 MS/MS full fragmentation scans (*R* = 35,000) using an isolation window of 26 *m*/*z* (0.5 *m*/*z* overlap between consecutive windows). AGC was set to 1e6 for both MS and MS/MS with ion accumulation times of 100 ms (MS) and 120 ms (MS/MS). Precursor ions were fragmented using HCD at a normalized collision energy of 30.

### Code availability

The software is available via a singularity container in zenodo with the 10.5281/zenodo.1438111, making it easy to use as software installation is not necessary. Detailed instructions are provided in Supplementary Note 7.

### Reporting Summary

Further information on experimental design is available in the Nature Research Reporting Summary linked to this article.

## Supplementary information

Supplementary Information

Description of Additional Supplementary Files

Supplementary Data 1

Reporting Summary

## Data Availability

All structural models and PyMOL sessions to support the findings of this study are available in zenodo with the 10.5281/zenodo.1438111. MS data have been deposited to ProteomeXchange via the MassIVE partner repository with accession codes MSV000082982 and PXD011969. A reporting summary for this article is available as a Supplementary Information file. All other data supporting the findings of this study are available from the corresponding authors on reasonable request.
