# Supplementary material for: Rapid determination of quaternary protein structures in complex biological samples
Source: Nat Commun. 2019 Jan 14;10:192. doi: 10.1038/s41467-018-07986-1 (PMC6331586; doi:10.1038/s41467-018-07986-1)
Supplement: Supplementary file 1 — Supplementary Information [file 41467_2018_7986_MOESM1_ESM.pdf]

Supplementary Notes 1

Comparative modeling

Rosetta Comparative Modeling protocol (RosettaCM) [1] is a high-resolution comparative modeling protocol which makes use of both Cartesian space and Torsion space to make a model of a protein structure by assembling its homologs. As the inputs to the protocol, it uses a set of homolog sequence alignment together with a fragment file prepared by de novo prediction of the unaligned region to model the 3D structure of the given protein. According to the results found in CASP10 (Critical Assessment of Techniques for Protein Structure Prediction) blind evaluation, RosettaCM generates models with more accurate side-chain and backbone conformations than other methods like MODELLER program [2] or I-TASSER [3] or any other methods that explicitly recombine multiple templates. The Supplementary Figure 1 shows the complete flowchart of RosettaCM modeling protocol. The input files to RosettaCM and the methods that generate these input data are as follows:

- The sequence alignment file of the main/given protein to proteins with defined structures. It can be generated using different web-based servers such as PSIBLAST [4] or Hhsearch [5].
- The standard Rosetta de novo modeling fragment sets which are used to model unaligned regions and also to explore deviation from the templates in the aligned regions. It can be generated by Rosetta software suite or by using the ROBETTA server.

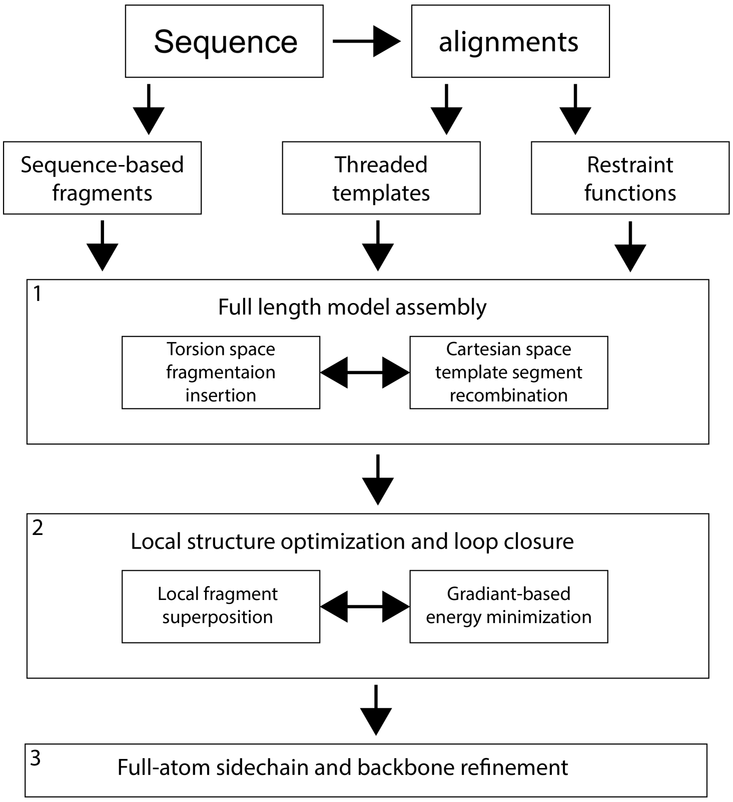

Supplementary Figure 1: RosettaCM protocol complete flowchart [1].

RosettaCM makes use of three different stages to make models from provided inputs for a protein of interest:

- Stage 1: The Rosetta-Threading protocol is used to thread the query sequence into each individual template and the threaded models would be aligned with the global frame. Then by using Monte Carlo approach and Rosetta low-resolution energy function the full-chain models will be created. This step also will be penalized according to the three-dimensional separation between adjacent residues in the sequence and by template structure distance restraint. For building the final structures, the Rosetta fold-tree approaches [6] is used in a way that the global position of each segment in the fold-tree is represented in Cartesian space while the backbone and side-chain conformation of the residues are represented in torsion space. Finally, by

incorporating two Monte Carlo moves the Cartesian space and torsion space are recombined by template-derived fragments and Rosetta de novo fragments consequently. The achieved results always have poor geometry at segment boundaries.

- Stage 2: This stage improves model geometry and also by using Monte Carlo sampling explore conformational changes away from the starting template.
- Stage 3: At last, side chains are built on and the structure is optimized by standard Rosetta full-atom refinement using a physically realistic energy function

Supplementary Notes 2

Protein-protein docking

Molecular modeling have been changed fundamentally in recent years by the introduction of low-resolution modeling techniques and fragment-based movers that uses parts of known protein structures to ensure that perturbations to the model simulation adheres to basic geometrical constraints that underpin the energy-landscape of proteins and their folding pathways [7]. This revolution and the increased understanding it provided have now let us design enzymes [8] in computers and to create new protein topologies [9] and self-assembling proteins [10]. The massive increase of useful application on these fields can be attributed to a better understanding of what governs the free energy difference between folded/unfolded and bound/unbound in combination with smarter and high-resolution "moves" together with vastly larger computational resources [11, 12]. Protein-protein docking and flexible-backbone protein-protein docking can now be carried out by routine as long as experimental structures or high-quality homology models exist [13].

We used **RosettaDock** to generate docking models which have been shown excellent effectiveness in protein-protein docking according to the obtained results on Critical Assessment of PRediction of Interactions (CAPRI). RosettaDock is a Monte Carlo (MC) based multi-scale docking algorithm that incorporates both a low-resolution, centroid-mode, coarse-grain stage and a high-resolution, all-atom refinement stage that optimizes both rigid-body orientation and side-chain conformation (Supplementary Figure **2**). The algorithm roughly follows the biophysical theory of an encounter complex formation followed by a transition to a bound state. Typically the algorithm starts from either a random initial orientation of the two partners (global docking) or an initial orientation that is randomly perturbed from a user-defined starting pose (local perturbation). From there, the partner proteins are represented coarsely, where side chains are replaced by a single unified pseudo-atom or centroid. During this phase, a 500-step Monte Carlo search was done with adaptive rotation and translational steps adjusted dynamically to achieve an acceptance rate of 25%. The ScoreFunction used in this stage primarily consists of a "bump" term, a contact term, and docking-specific statistical residue environment and residue-residue pair-wise potentials (Supplementary Table **1**).

Once the centroid-mode stage is complete, the lowest energy structure accessed during that stage is selected for high-resolution refinement. During high-resolution refinement, centroid pseudo-atoms are replaced with the side-chain atoms at their initial unbound conformations. Then 50 Monte Carlo Minimization (MCM) steps are made in which:

- The rigid-body position is perturbed by a random direction and magnitude specified by a Gaussian distribution around 0.1 Å and 3.0°
- The rigid-body orientation is energy-minimized
- The side-chain conformations are optimized with RotamerTrials, followed by a test of the Metropolis criteria.

Every eight steps, an additional combinatorial side-chain optimization is carried out using the full side-chain packing algorithm, followed by an additional Metropolis criteria check. To reduce the time devoted to the computationally expensive energy-minimization for unproductive rigid-body moves, minimization is skipped if a rigid-body move results in a change in score of greater than +15. The all-atom score function used in this stage primarily consists of Van der Waals attractive and repulsive terms, a solvation term, an explicit hydrogen bonding term, a statistical residue-residue pair-wise interaction term, an internal side-chain conformational energy term, and an electrostatic term.

For particular targets, a variety of RosettaDock sampling strategies is often used to improve the chance of achieving an accurate structure prediction. If no prior structural or biochemical information is known about the protein interaction of interest, global docking is used to randomize the initial docking poses. From there, score filters and clustering are used to identify clusters of acceptable low-energy structures for further docking and refinement. In most cases, there is some known information about the complex, either in the form of related protein complexes or in biochemical or bioinformatics data which identify probable regions of interaction on the protein partners. In these cases, users manually arrange the starting docking pose to a configuration that is compatible with the information and carries out a local docking perturbation. Additionally, users can set distance-based filters that bias sampling towards those docking poses that are compatible with specified constraints [14].

For each docking modeling, at first, each of partners in the complex will be relaxed which keeps atoms as close as possible to the original positions in the crystal structures while simultaneously minimizes the energy function. For this purpose, the Rosetta Fast Relax algorithm is used. Relax does not carry out any extensive refinement and only searches the local conformational space neighborhood. Moreover, the dock-low-res protocol

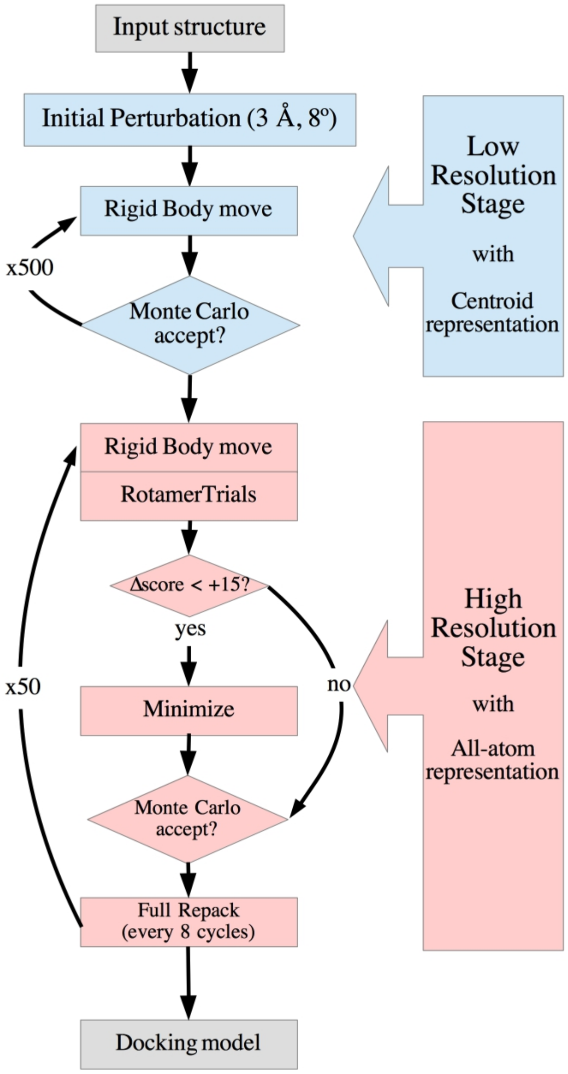

Supplementary Figure 2: RosettaDock workflow [14].

Supplementary Table 1: RosettaDock scoring components and weights [14].

Component	name	weight
**Centroid docking score function**		
Contact	interchain_contact	2.0
Bump	interchain_vdw	1.0
Environment	interchain_env	1.0
Pair-wise interaction	interchain_pair	1.0
**All-atom docking score function**		
Van der Waals (attractive)	fa_atr	0.338
Van der Waals (repulsive)	fa_rep	0.044
Dunbrack side-chain	fa_dun	0.036
Solvation	fa_sol	0.242
Pair-wise interaction	fa_pair	0.164
Hydrogen Bond	hbond_ir_bb, hbond_sr_bb, hbond_bb_sc, hbond_sc	0.245
Electrostatic	hack_elec	0.026

generates thousands of decoys together with a related score file (global docking). The "interchain-cen" score is computed, in this part which is constituted from different energy terms like interchain_contact, interchain_env, interchain_pair, and interchain_vdw. The global docking results and the scores are then used to calculate the "best docking interface" in order to run high-res protocol (local perturbation) to obtain final precise docking models. Note that the number of initial docking models needed for the method to converge is highly system dependent where small proteins with high-resolution crystal structures need orders of magnitude less low-resolution models compared to large, fibrous proteins where no crystal structures of the native nor of close homologs exist. Since determining the number of models, a-priory is not trivial, we tend to produce more models than minimally needed.

After finding the binding interface, the top structures satisfied the criteria of the selected binding interface would be selected for local docking with high-resolution. In this part, hundreds of docking models will be generated concentrating on the specific docking interface to find the best docking constitution. For high-resolution docking, the Rosetta Talaris 2014 is selected as a score function which contains several energy terms such as: fa_atr, fa_dun, fa_elec, fa_pair, fa_rep, fa_sol, hbond_bb_sc, hbond_lr_bb, and hbond_sc. Moreover, we set the docking perturbation parameter to 3 Å translation and 8°rotation. For sidechain packing, extra rotamers were used for the χ_1 for all residues and for χ_2 for aromatic residues, and unbound rotamers were included as well [15].

Finally, the top models according to the Rosetta score are selected and the computational cross-links are generated by a prototyped application, **RosettaXL**. RosettaXL (XL: cross link) is a simple but useful application which produces the cross-linked peptide according to the user-defined threshold and makes a .kojak format of XLs which are used for mass spectrometry analysis. RosettaXL also produces a SQLite data base table to store all generated information related to the XL-peptides which is useful for the data management and analysis. Supplementary Table 2 provides more details about the output format in the SQLite table.

Supplementary Table 2: More details about information on RosettaXL generated SQLite table.

Column header	Details
**struct_id**	Selected complex (from a list or a silent file) to generate computational XLs.
**total_score**	Rosetta energy score for the whole complex (Talaris 2014 here).
**chain_res1**	The name of first chain and the number of first Lysine on that chain.
**chain_res2**	The name of second chain and the number of first Lysine on that chain.
**Eu_dist**	Euclidean distance between two Lysines in the XL.
**enzyme**	Selected enzymatic digestion for making computational XLs. Here it can be Trypsin or Glucin.
**type**	Inter_XL or Intra_XL which refers to XLs between different chains or within the same chain.
**XL_peptide**	Computational XL generated in kojak format.

Supplementary Note 3

Machine Learning based MS1 data analysis

We used here a Machine Learning (ML) approach to find top XL peptides using isotopic patterns derived from hrMS1 experimental data. A popular ensemble learning approach, bagging (bootstrap aggregating), is used to recognize non-trivial patterns in the hrMS1 data. Bagging is an ensemble-based approach in ML which makes an aggregated predictor by generating multiple versions of a basic predictor. These multiple versions would be constituted of bootstrap replicates of the training data set and using these again as a new training data sets. Bagging is well-established to avoid overfitting due to creating of the variance amongst the training set by sampling and replacing it. According to the literature, in comparison to single predictor approaches, bagging can provide substantial gains in accuracy [16].

Discovering XL patterns in hrMS1 data is difficult by statistical approaches due to the level of noise, and high data dimensionality. Meanwhile, the number of real XLs is a few among all possible computational XLs. Machine Learning as a powerful tool, can distinguish the isotopic pattern of real XLs in the MS1 data. The machine needs to be trained on features extracted from experimental data where the training data plays a key role in the whole process. According to the literature, the M1 protein and fibrinogen create a protein-protein interaction network with a specific cross-like pattern. Atomic coordinates and structure factors for constituted complex have been deposited with the Protein Data Bank with PDB IDs 2XNX and 2XNY. So it provides a strong training data and a case study in a way that we can use different chains e.g. DEF-GHI of 2XNX to predict ABC or JKL and verify the generated output models with the native crystallized complex.

As it is shown in Supplementary Figure 3, by considering chains DEF-GHI from 2XNX (in blue color), a feature table is initialized by using Dinosaur and Taxlink on all generated computational XLs by RosettaXL. We considered the total number of 13 different features which are: monoisotopic masses {lightMonoMz, heavyMonoMz}, feature retention times {rtApexLight, rtApexHeavy}, masses {massLight, massHeavy}, intensity values {intLight, intHeavy}, scores provided by dinosaur {intScore, rtScore, massScore, scoreSum}, and feature charge states {z}. This table has been used then to train the machine in a way that all inter XLs that are below 30 Å have been considered as good XL (class 1 - red dot in Supplementary Figure 4) and all inter XLs in the range of 50 Å and above have been considered as bad or impossible XLs (class 0 - blue dot in Supplementary Figure 4) while the rest are excluded from the training data. Because 2XNX contains four fibrinogen macromolecules that bind M1 protein in two different binding sites, it is possible to predict each binding interface, by considering two others on the opposite side. Thus, we trained the machine here by using feature patterns of M1-2XNX-DEF-GHI to predict M1-2XNX-ABC.

The accuracy of the algorithm is tested using a 10-fold cross validation technique which is approximately 98% on the whole dataset and about 83% for class 1 data as Supplementary Figure 4 indicates (which is important here as we want to recognize the pattern of true XLs among all possibilities). We further tested this algorithm on intra XLs of fibrinogen and albumin which is explained on the main article. Finally, to select the number of components, we followed the law of diminishing returns in ensemble construction and set the number of components equal to the class labels (two here) [17]. Increasing this number can overfit the data and reduce the accuracy on the test set. The ML-based analysis workflow is shown in Supplementary Figure 5. The software package (in Python) together with the training data are provided online.

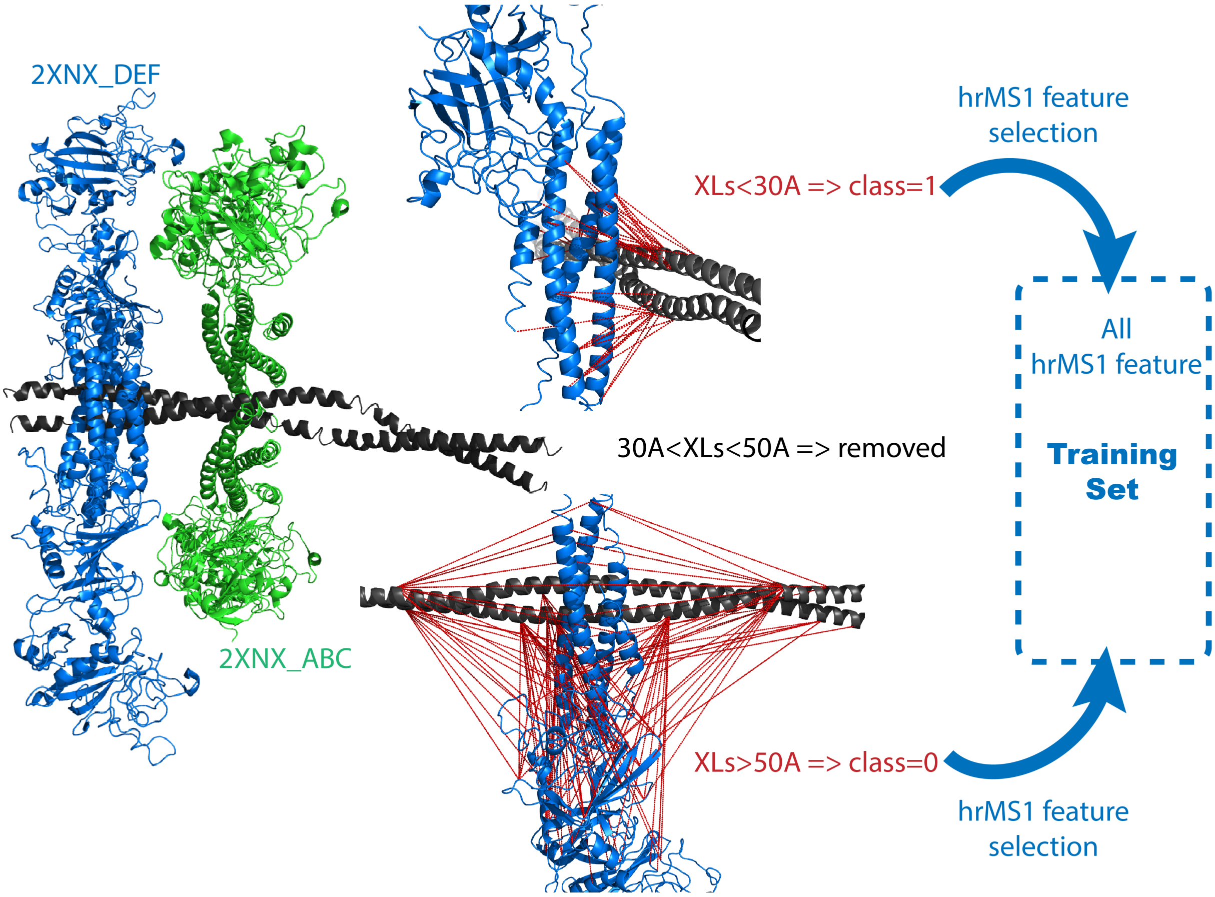

Supplementary Figure 3: Feature selection for the training set. All computational XLs are analyzed on 2XNX crystal structure and two classes {0, 1} are made according to the Euclidean distances. All features are computed using Dinosaur and Taxlink from h1MS1 samples for these XLs to generate the training set.

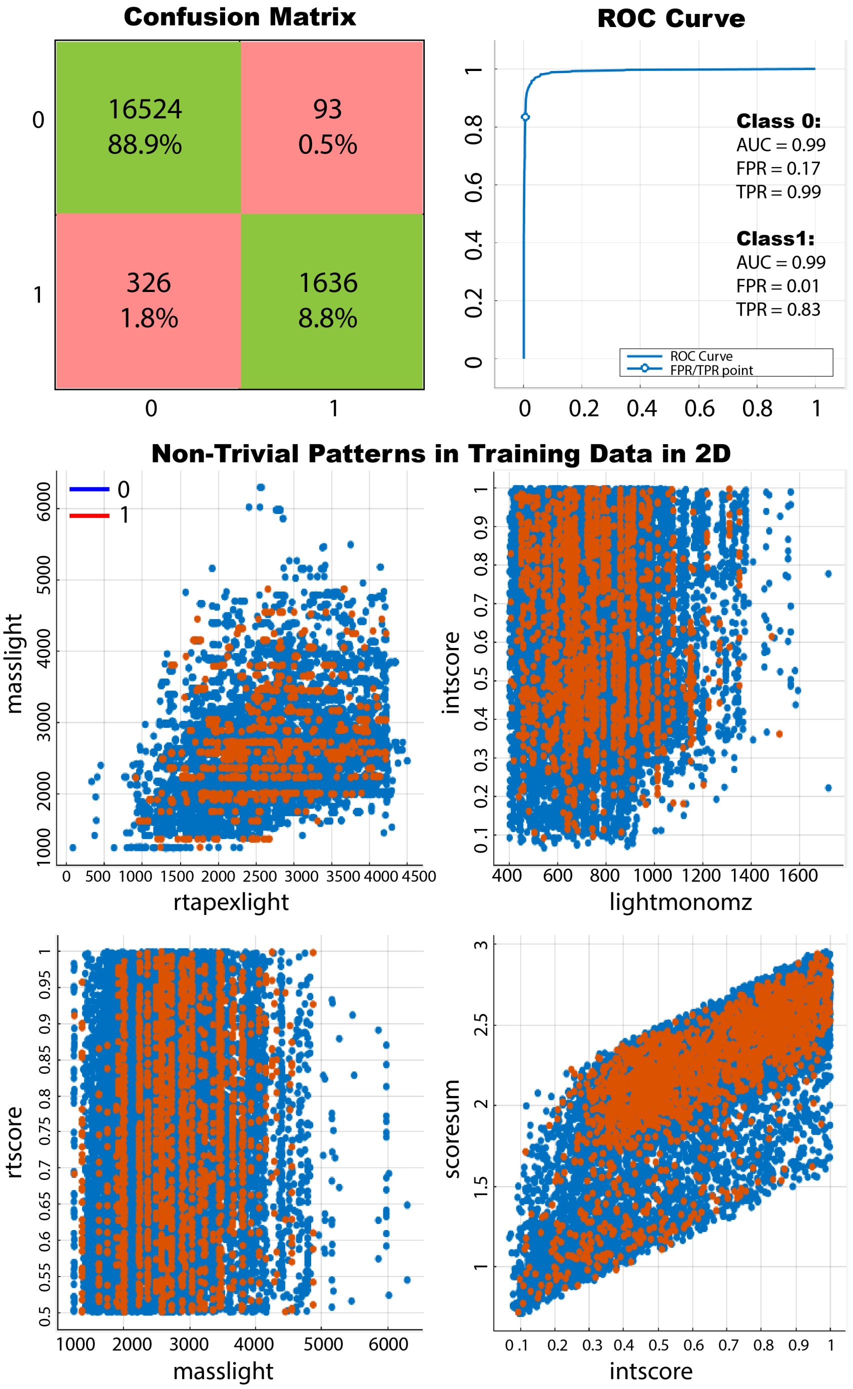

Supplementary Figure 4: Machine Learning approach to analyze hrMS1 experimental data and find isotopic patterns of real XLs. Confusion Matrix and ROC plot are the results of applying bagging on the training data by 10-fold cross validation. Four plots below show the distribution of non-trivial patterns of real XLs on the features derived from hrMS1 data. All plots are generated by MATLAB Classification Learner toolbox.

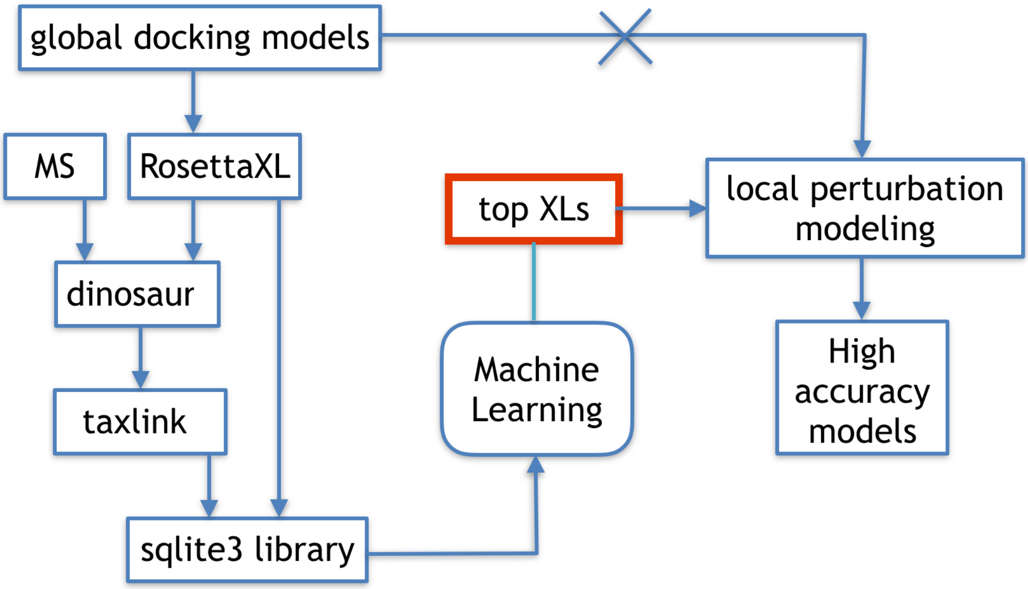

Supplementary Figure 5: Machine learning based workflow to analyze hrMS1 data. Dinosaur is a feature finding algorithm for detection of isotopic patterns in HPLC-mass spectrometry data, and Taxlink was developed for cross-linking mass spectrometry. The workflow shows that we do not follow the traditional docking approach from low-resolution models to high-resolution local perturbation models. We use experimental data as constraints to predict binding interface that is supported by the majority of XLs, and then we make high-resolution models in that particular binding interface.

Supplementary Note 4

MS2 Data analysis

The MS2-analysis is developed as a Python package that helps to search through the MS2-DDA data and find those spectra that match the theoretical XL-peptide fragments the best. The algorithm takes a list of XLs which can be provided by RosettaXL -Supplementary Note 2- or from hrMS1 analysis output -Supplementary Note 3- together with the desired DDA file (in the MGF format) as the inputs. The MGF format is a simple human-readable format for MS2 data. It allows storing MS2 peak lists and experimental parameters. The output is a SQLite table contains following information: XL, mgf_file, spectrum_id, spectrum_num, delta, charge, peaks_num, fragSc, coverage, and covered_Frags. Supplementary Table 3 provides more details about each of these information.

Supplementary Table 3: More details about information on MS2 SQLite table.

Column header	Details
**XL**	Selected XL for MS2 analysis.
**mgf_file**	Selected MGF file for searching all fragments and finding best spectrum.
**spectrum_id**	The id (spectrum name in MGF file) of selected spectrum as the best spectra.
**spectrum_num**	The number of selected spectra in MGF file.
**delta**	Selected delta for the whole analysis and peak detection (default is 0.01 for precursor and 0.05 for fragments)
**charge**	Best charge state which based on that, more peaks detected and top spectra is selected.
**peaks_num**	Number of fragments detected on the selected spectra.
**fragSc**	The intensity-based score that the selected spectra obtained.
**coverage**	The ratio of number of detected fragments on selected spectra to the all peaks on that spectra.
**covered_Frags**	A list of all detected fragments in selected spectra.

The algorithm considers both “y” and “b” ion-types together with all charge states (precursor charge states 3-8 and fragment charge states 1-3) and stores them all to the SQL table. Accordingly, we consider all possible charge states for precursor and fragments and compute all mass/charge ratios and then search them all in each spectra of the MGF file. Furthermore, the precursor mass/charge value is used as a filter to reduce the time complexity in a way that those spectra in the MGF file would only be considered if they have the same precursor mass/charge with the selected XL. By using this filter, thousands of spectra will be filtered out due to unmatched precursor mass/charge value which increase the processing speed by the order of magnitude. Meanwhile, we considered a delta window for this comparison to be able to cover all isotopic mass of one special XL. The general workflow is shown on Supplementary Figure 6).

We have implemented a novel scoring system here. For this purpose, we considered three important factors; user-defined intensity filter, intensity segmentation, and the existence of the cross-linker arm in fragments. Accordingly, if the spectra hit the XL-fragment with m/z value, the intensity level is checked, and with respect to that, the spectra achieve a weighted positive score. Meanwhile, the existence of the cross-linker arm has also a positive impact on the score weight. Finally, those spectra that match with highest intensity the XL-fragments contains the cross-linker arm will obtain the highest score. Please note that the user-defined intensity filter could also remove the base level of noise in the scoring system. The parallel implementation together with using of Python dictionary which is a well-defined/fast data structure help MS2-analysis to have an efficient running speed. Moreover, storing all the output data in SQL tables provides the possibility of a vast and crucial statistical analysis on the output data. Meanwhile, the fig_maker.py code in this package helps to generate Scalable Vector Graphics (SVG) images from a user-defined number of top XLs according to the fragment score. The output images will be stored according to the score they obtained. Finally, the prerequisites to run the code consist of installing Pyteomics [18] and SQLite3 Python libraries and having software installed for MGF conversion.

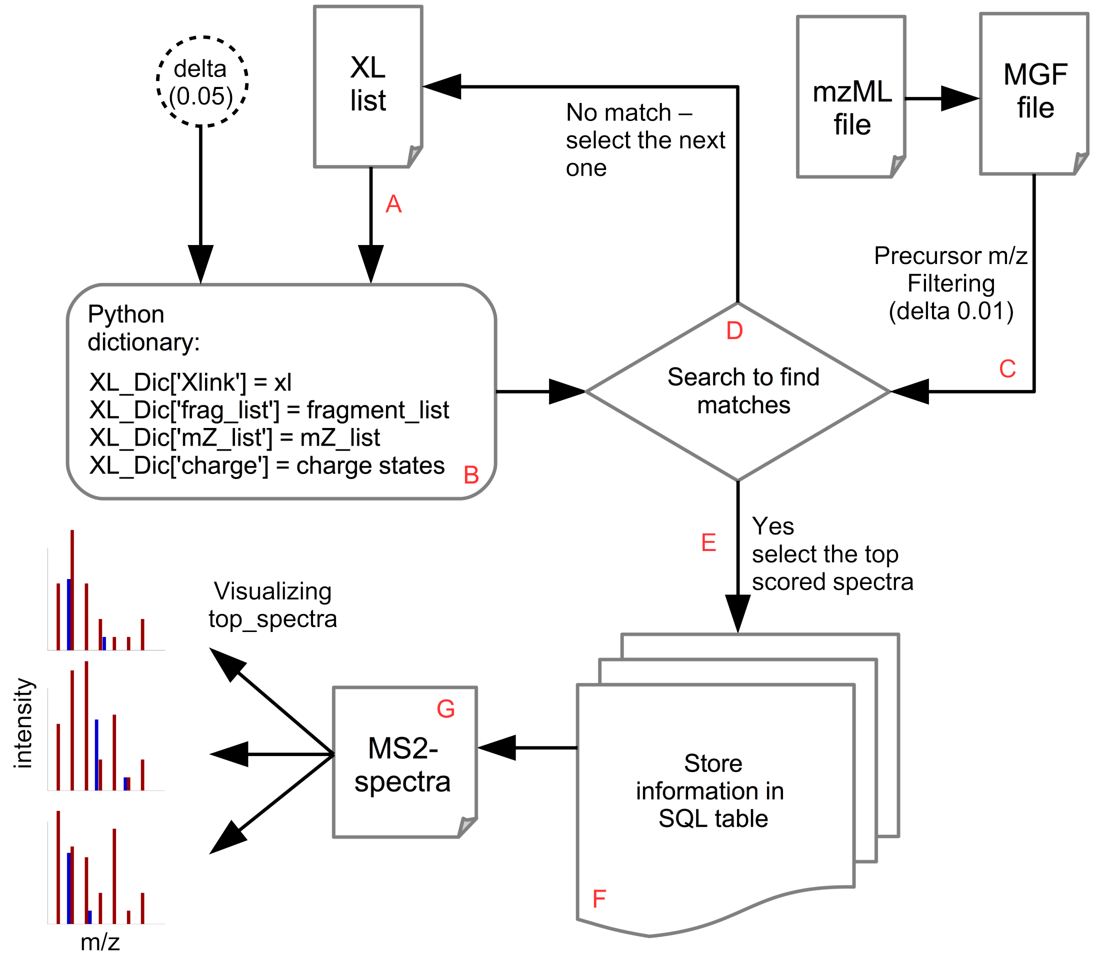

Supplementary Figure 6: MS2 workflow. (A) The XL file is parsed, and the first XL is selected. (B) All fragments of the selected XL are generated together with mass/charge values and stored in a Python data structure; "dictionary". (C) The precursor mass/charge value is computed, and considered as a filter to search through MGF spectra. An isotopic window is considered to cover all possible isotopic masses, and all spectra within this isotopic window will be stored in a different file for further analysis. (D) For every fragment in the dictionary, the mass/charge value is compared with the mass/charge value of filtered MGF spectra. For every hit in the range of predefined delta (0.05), the spectra will achieve a positive score. We proposed here a weighted score system to make it more informative and to reduce as much as possible the noise impact on the scoring process. (E) The score lists of all spectra are checked and the top spectra according to the score (number of supported fragments) will be selected. (F) All the information are stored in an SQL table, and the algorithm will be repeated with the next XL. (G) Top spectra will be visualized, and required information will be saved in a separate file for more analysis.

Supplementary Note 5

Data Independent Acquisition (DIA) analysis

Data independent data acquisition followed by targeted data analysis is the newest data acquisition technique. Here, systematic fragment intensities are collected in a similar fashion to SRM but with the big difference that many peptides are fragmented and measured at once. This increases the throughput to levels similar to shotgun and retains the quantitative accuracy of SRM. The sensitivity is higher compared to shotgun but lower than SRM. SWATH-MS depends on highly specialized algorithms to de-convolute the complex data and quantifies the peptide ions [19].

Here, the DIA analysis workflow, is a Python package contains different methods (in term of implementation strategy) for DIA analysis. We used XL-based DIA analysis approach which is indeed based on theoretical fragments of each XL. As it is shown in Supplementary Figure 7, by having a set of input XLs, the workflow starts with generating all possible theoretical fragments for the first XL on the list. Then by considering fragment charge levels 1 to 3 and precursor charge levels 3 to 8 a CSV file is made contains all m/z values in two columns which the first one contains the precursor m/z values and the second one stores the fragments m/z values.

The process then be continued by converting the CSV file to TraML file (using OpenMS file converter) and then by calling OpenSWATH makes a chromatogram by considering all DIA experimental files (which could be defined as command line option). The output is a set of vectors which needs to be visualized for pattern recognition.

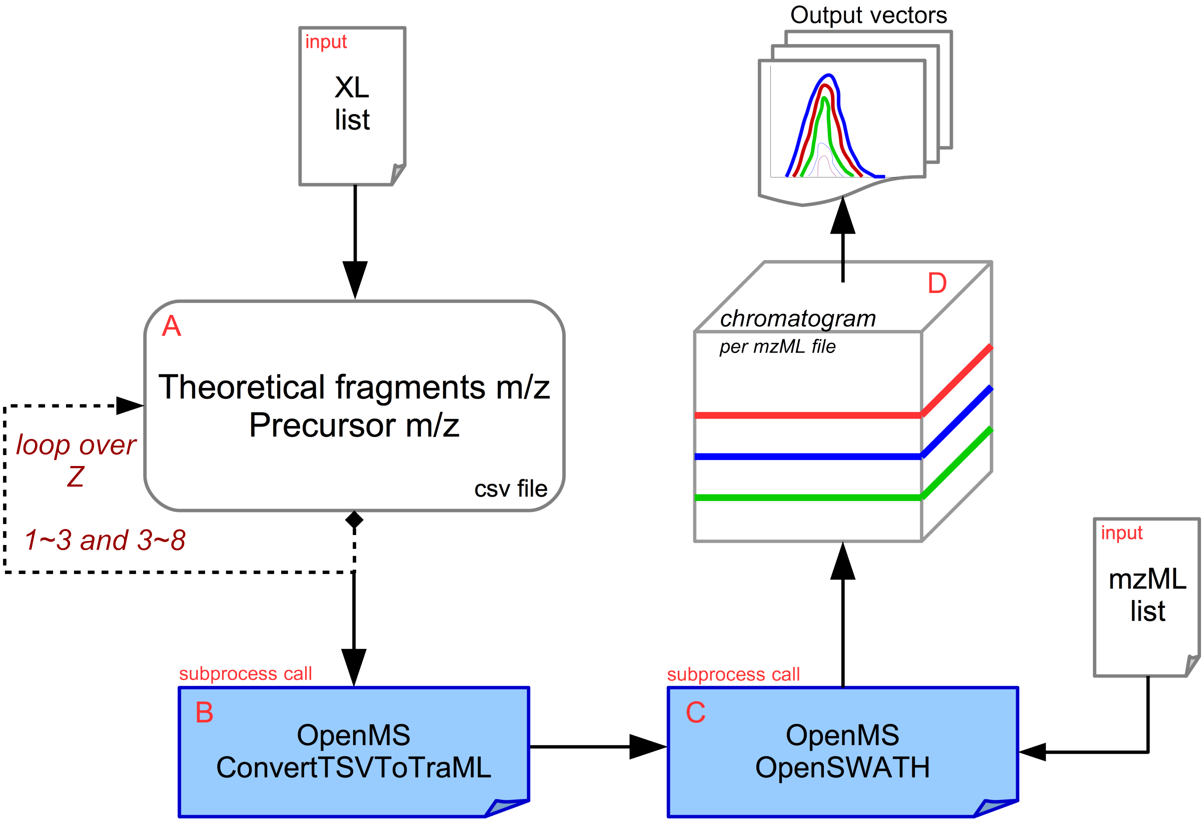

Supplementary Figure 7: DIA workflow. (A) The input XL is parsed and all fragments together with a list of m/z values with respect to the different charge values will be generated and stored in a CSV file. (B) OpenMS function "ConvertTSVToTraML" makes a TraML file within a subprocess call. (C) "OpenSWATH" then is called to make the chromatograms by considering mzML input list. (D) Each chromatogram is analyzed and the intensity vectors are generated and visualized to search for possible patterns. Blue blocks are related to OpenMS software suit which are called as subproces in the package.

Supplementary Table 4: List of MS experiments and data. All the data here are traceable on openBIS web server.

Sample ID	MS1	MS2-DDA	DIA
M1+albumin_0_DSS_1	Lotta_S1605_073	Lotta_S1605_071	Lotta_S1605_072
M1+albumin_0_DSS_2	Lotta_S1605_076	Lotta_S1605_074	Lotta_S1605_075
M1+albumin_100_DSS_1	Lotta_S1605_079	Lotta_S1605_077	Lotta_S1605_078
M1+albumin_100_DSS_2	Lotta_S1605_082	Lotta_S1605_080	Lotta_S1605_081
M1+albumin_250_DSS_1	Lotta_S1605_085	Lotta_S1605_083	Lotta_S1605_084
M1+albumin_250_DSS_2	Lotta_S1605_088	Lotta_S1605_086	Lotta_S1605_087
M1+albumin_500_DSS_1	Lotta_S1605_091	Lotta_S1605_089	Lotta_S1605_090
M1+albumin_500_DSS_2	Lotta_S1605_094	Lotta_S1605_092	Lotta_S1605_093
M1+albumin_1000_DSS_1	Lotta_S1605_097	Lotta_S1605_095	Lotta_S1605_096
M1+albumin_1000_DSS_2	Lotta_S1605_100	Lotta_S1605_098	Lotta_S1605_099
M1+albumin_2000_DSS_1	Lotta_S1605_103	Lotta_S1605_101	Lotta_S1605_102
M1+albumin_2000_DSS_2	Lotta_S1605_106	Lotta_S1605_104	Lotta_S1605_105
M1+Fibrinogen+Albumin_0000_DSS	Simon_S1606_093		
M1+Fibrinogen+Albumin_0100_DSS	Simon_S1606_094		
M1+Fibrinogen+Albumin_0250_DSS	Simon_S1606_095		
M1+Fibrinogen+Albumin_0500_DSS	Simon_S1606_096		
M1+Fibrinogen+Albumin_1000_DSS	Simon_S1606_097		
M1+Fibrinogen+Albumin_2000_DSS	Simon_S1606_098		
M1+Fibrinogen_0000_DSS_1	Simon_S1606_115	Simon_S1501_074	Simon_S1505_180
M1+Fibrinogen_0000_DSS_2	Simon_S1606_116	Simon_S1501_075	Simon_S1505_181
M1+Fibrinogen_0100_DSS_1	Simon_S1606_117	Simon_S1501_076	Simon_S1505_182
M1+Fibrinogen_0100_DSS_2	Simon_S1606_118	Simon_S1501_077	Simon_S1505_183
M1+Fibrinogen_0250_DSS_1	Simon_S1606_119	Simon_S1501_078	Simon_S1505_184
M1+Fibrinogen_0250_DSS_2	Simon_S1606_120	Simon_S1501_079	Simon_S1505_185
M1+Fibrinogen_0500_DSS_1	Simon_S1606_122	Simon_S1501_081	Simon_S1505_187
M1+Fibrinogen_0500_DSS_2	Simon_S1606_123	Simon_S1501_082	Simon_S1505_188
M1+Fibrinogen_1000_DSS_1	Simon_S1606_124	Simon_S1501_083	Simon_S1505_189
M1+Fibrinogen_1000_DSS_2	Simon_S1606_125	Simon_S1501_084	Simon_S1505_190
M1+Fibrinogen_2000_DSS_1	Simon_S1606_126	Simon_S1501_085	Simon_S1505_191
M1+Fibrinogen_2000_DSS_2	Simon_S1606_127	Simon_S1501_086	Simon_S1505_192
PA-TXMS_AP1_0.0mM_DSS_01	Simon_S1605_245	Simon_S1606_063	Simon_S1606_083
PA-TXMS_AP1_0.0mM_DSS_02	Simon_S1605_246	Simon_S1606_064	Simon_S1606_084
PA-TXMS_AP1_0.5mM_DSS_01	Simon_S1605_247	Simon_S1606_065	Simon_S1606_085
PA-TXMS_AP1_0.5mM_DSS_02	Simon_S1605_248	Simon_S1606_066	Simon_S1606_086
PA-TXMS_AP1_2.0mM_DSS_01	Simon_S1605_251	Simon_S1606_068	Simon_S1606_088
PA-TXMS_AP1_2.0mM_DSS_02	Simon_S1605_252	Simon_S1606_069	Simon_S1606_089
PA-TXMS_AP1_4.0mM_DSS_01	Simon_S1605_253	Simon_S1606_070	Simon_S1606_090
PA-TXMS_AP1_4.0mM_DSS_02	Simon_S1605_255	Simon_S1606_071	Simon_S1606_091

Supplementary Note 6

Multi-template Comparative Modeling of M1 protein

Group A Streptococcus (GAS) has a crucial surface-bound virulence factor known as M1 protein (UniProt id Q99XV0). Despite the effort of several research groups, the complete tertiary structure of M1 protein remains unknown. Indeed, understanding the tertiary structure is a key factor to reveal the function of the protein and its interactions with host proteins, such as plasma and saliva proteins. As the main goal of this study is to find the interactions between M1 protein and plasma proteins, the first step is to model the full-length tertiary structure of the M1 protein. To do that, we have proposed a 3D structure model of M1 protein using the Rosetta Comparative Modeling protocol (RCM) [1] -Supplementary Note 1- by incorporating available homologues with known tertiary structure and all possible information as the constraints of the modeling. The partial structure of M1 which is a conformationally dynamic coiled-coil dimer (PDB id 2XNX) [20] plays a key role in our prediction.

M1 protein (UniProt id Q99XV0) has an amino acid sequence of 484 residues. The first part of the sequence (approximately residues 1 to 41) is predicted by Robetta server to contain a "Signal Peptide-YSIRK-signal" which is a long and single alpha-helix of hydrophobic amino acids (Supplementary Figure 8). Meanwhile, the end part (approximately residues 460 to 484) of the sequence is predicted to be a "Transmembrane - Gram-pos-anchor" domain which always has an alpha-helical or beta-barrels like structure. The rest part (approximately residues 42 to 459) constitute a "Coiled-Coil" structure according to the all results derived from coiled-coil servers such as MARCOIL [21] and PCOILS [22] (Supplementary Figure 9).

Accordingly, to predict the 3D structure of this protein using RosettaCM, the sequence can simply be divided to three or four different parts which provide a better understanding of the whole structure. The strategy we considered here is based on a powerful computer science approach called dynamic programming (dynamic optimization) which break down a complex problem into several simpler sub-problems and solve them once to achieve the final solution. The final 3D structure could be achieved by incorporating another comparative modeling approach using the predicted results for each sub-devisions.

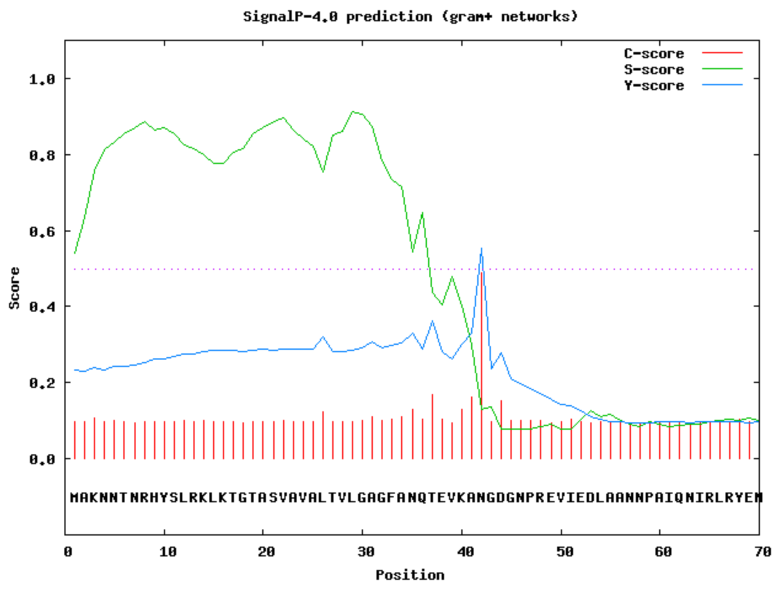

Supplementary Figure 8: M1-protein sequence analysis by Robetta (obtained Signal Peptide region).

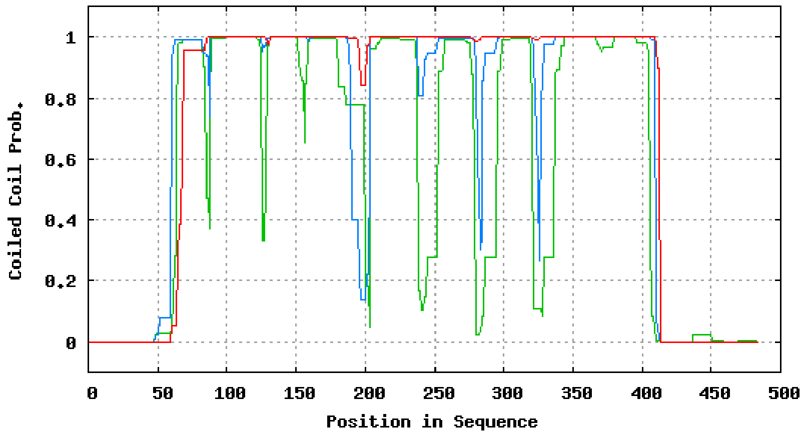

Supplementary Figure 9: M1-protein sequence analysis by PCOIL server (defined the Coiled-Coil region).

The basic homolog analysis of the full-length sequence of the M1 protein is achieved by pGenTHREADER [23] which is shown in Supplementary Table **5**. Meanwhile, a better homolog sequence analysis has been achieved by dividing the sequence into four different parts as follows:

- Signal peptide region: covers the first part (N-terminal) of the sequence.
- Coiled-coil region(s) (one or two parts): cover most of the sequence and has a key role in protein docking analysis.
- Disordered region: from residue 398 to residue 456 which is not modeled.
- Anchor: covers the last part (C-terminal) of the sequence.

Accordingly, several homologues proteins have been found for each of these parts. Supplementary Table **8** shows all the PDB ids of selected proteins for multi-template comparative modeling. By considering these homologs as a structural template, we have addressed the problem of M1 protein modeling. A graphical view is also provided in Supplementary Figure **10**.

Supplementary Table 5: M1 protein complete homolog analysis by pGenTHREADER.

NO	Conf	NetScore	p_value	Pairwise_E	Solvation_E	Aln_Score	Aln_Len	Str_Len	Seq_Len	PDBchain
1	CERT	94.688	1e-08	-532.6	-18.3	369	339	396	484	4hpqC0
2	CERT	93.44	2e-08	-273.8	-10	466	137	137	484	2otoA0
3	CERT	83.354	2e-07	-151.2	-4	438.1	108	108	484	2xnxM0
4	CERT	75.123	1e-06	-429.5	-5.1	267	384	475	484	1hciA0
5	CERT	67.808	7e-06	-400.9	-12.3	226	311	322	484	3edvA0
6	CERT	67.753	7e-06	-419.6	-10.3	249	155	155	484	2efrA0
7	CERT	66.794	9e-06	-650.8	-11	156	334	445	484	4l6yA0
8	CERT	64.644	2e-05	-316.1	-15.9	224	288	318	484	1u4qA0
9	CERT	63.576	2e-05	-191.1	-20.3	217	445	602	484	1ciiA0
10	CERT	62.684	2e-05	-447.9	-17.6	186	230	323	484	2p01A0
11	CERT	62.514	2e-05	-429.7	-7.8	213	156	160	484	2v71A0
12	CERT	62.437	3e-05	-325.1	-17.3	226	158	158	484	4gkwA0
13	CERT	62.178	3e-05	-263.6	-13.3	232	234	239	484	3s84A0
14	CERT	60.372	4e-05	-197.8	-12.5	239	220	237	484	3na7A0
15	CERT	60.263	4e-05	-205.1	-1.5	229	348	697	484	2xs1A0
16	CERT	60.145	4e-05	-375.1	-15.6	201	151	155	484	2efrA0
17	CERT	59.898	5e-05	-400.2	-10.7	176	296	309	484	2ch7A0
18	CERT	59.815	5e-05	-312.5	-14.1	216	152	152	484	3o0zA0
19	CERT	58.424	6e-05	-396.5	-14.5	160	307	376	484	4dylA0
20	CERT	58.301	7e-05	-278.8	-6.6	211	223	229	484	4tqlA0
21	CERT	57.056	9e-05	-324.3	-10.8	202	127	152	484	3o0zA0

Supplementary Table 6: General properties of homologues protein used for partial modeling of M1.

Region	Residue Number	Homolog PDB ID
Signal Peptide	1-41	4GWP, 3LB6
Coiled-Coil parts 1 and 2	42-459	2XNX, 2OTO, 1C1G, 2EFR, 2DFS, 2KK9, 4CGK
Anchor	460-484	2JWA, 2KLU, 2KS1, 2N2A, 2WW8, 3UXF

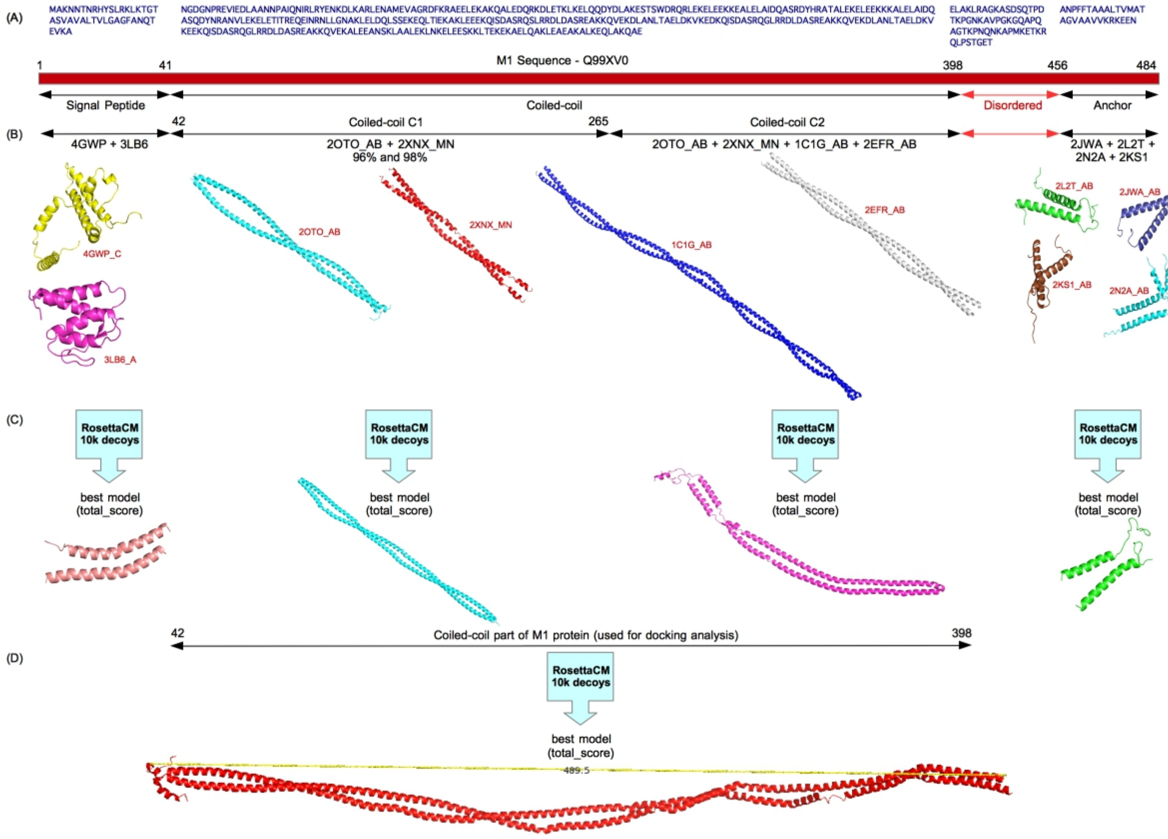

Supplementary Figure 10: A graphical view of M1-protein multi-template comparative modeling using RosettaCM. (A) the Robetta server sequence analysis reveals four different modeling criteria: signal peptide, coiled-coil region, disordered region, and anchor. (B) selected homologs for each part according to the sequence identity and alignment score. (C) producing 10k decoys by RosettaCM for each part and obtaining the best model according to the Rosetta energy function. (D) using generated models as the input to RosettaCM and produce 10k decoys to find the near native structure for the main coiled-coil region. The produced model's length is about 49nm and by considering an average cell-wall thickness of approximately 10 nm it will be reasonable to use the model for docking analysis.

Supplementary Table 7: General information about selected protein complexes.

UniProt ID	PDB IDs	Abbreviation	Description	Mass (Da)
P02671, P02675, P02679	2XNX, 3GHG	FIB	Fibrinogen alpha, beta and gama chains	94,973, 55,928, 51,512
P02768	1E7I, 1E78, 1AO6	ALB	Serum albumin	69,367
Q99XV0	2XNX	M1	M protein type 1	54,221
Q6PYX1	4QGT	IgG	Hepatitis B virus receptor binding protein	38,162
P00738	4WJG, 4X0L	HP	Hemoglobin	45,205
P01009	2D26	SERPINA1	Alpha-1-antitrypsin	46,737
P00488	1F13	F13A1	Coagulation factor XIII A chain	83,267
P10909	1C5G	CLU	Clusterin	52,495
P02745, P02746, P02747	2WNU	C1Q	Complement C1q subcomponent subunit A, B, C	26,017, 26,722, 25,774
P02647	–	APOA1	Apolipoprotein A-I	30,778
P04003	5HYU, 5HZP	C4BP	C4b-binding protein alpha chain	67,033

Supplementary Table 8: Cross-link data for M1-protein interactions in complex biological samples. The table is separated according to the results from purified (top) and plasma (down) samples.

Complex	Low-res models	High-res models	XLs-total	hrMS1	MS/MS	DIA
M1-FIB	600,000	60,000	27	24	7	7
M1-ALB	300,000	30,000	29	13	21	7
ALB-ALB	–	–	88	55	39	31
FIB-FIB	10,000	1000	68	53	19	19
M1-HP	100,000	10,000	39	11	35	5
M1-IgG	30,000	1000	6	1	6	1
M1-F13A1	100,000	10,000	13	8	5	1
M1-C4BPa	30,000	1000	9	3	7	1
M1-APOA1	–	–	11	1	11	1
M1-CLU	–	–	13	1	13	1
M1-C1Q	100,000	–	0	0	0	0
M1-SerpinA1	100,000	–	1	0	1	0
FIB-F13A1	10,000	1000	14	4	10	2
ALB-SerpinA1	10,000	1000	7	3	5	1
HP-ALB	10,000	–	20	0	20	0
HP-SerpinA1	10,000	–	7	0	7	0
**Total**	**1,410,000**	**115,000**	**352**	**177**	**206**	**77**

Supplementary Note 7

Instruction note

TX-MS is provided as a software package contains several tools applicable to be installed and used separately or inside the package. The package contains three main parts; 1- MS1 analysis, 2- MS2 analysis, and 3- DIA analysis. Although there are some prerequisites for each part, most of the analysis is written in Python 2.7 and can be used simply in command line. Here, we explain the general instructions to make use of each analysis part in detail.

MS1 analysis: We proposed a machine learning based approach for MS1 analysis (see Supplementary Note 3). The workflow is based on Dinosaur and Taxlink which are written in JAVA and easy to install and use. The inputs/outputs, requirements, and all the commands to use the MS1 analysis are as follow:

[Commands]

1- *python ./seq2xl.py -s1 sequence1.fasta -s2 sequence2.fasta*

A Python code to make all possible XL combinations from sequence1, and sequence2 and store all XLs in a .txt file in the working directory.
-sequence1.fasta, and sequence2.fasta are the .fasta files of the proteins of interest. Each file should only contain sequence without any identification. Another formats like .txt files can be used as well.

2- *java -jar ./dinosaur/target/Dinosaur-1.1.3.free.jar –verbose –profiling –concurrency=4 ms1.mzML*

Dinosaur as explained in Supplementary Note 3, make .features.tsv file from input .mzML file.
-ms1.mzML is the general MS1 data in mzML format.

3- *java -jar ./taxlink/Taxlink-0.8.1.jar dinosaur.features.tsv ALL_XLs.txt*

Taxlink combines the output of 3-1 and 3-2 (all_XLs and dinosaur feature file) and make a .csv file containing all the features of selected XLs. This file is the test file for Machine Learning algorithm in the next step.

4- *python ./TX_Learning.py –train training_set.csv –test taxlink.isopaits.csv –ensemble [integer] –kfold [5 or 10 or 20]*

TX_Learning is the main application to run the machine learning algorithm and find top XLs. The training set is provided as mentioned above, the .csv file is the output of Taxlink, the ensemble options, can be set from 2 to any number. Here we set it up to 2 as we have two classes for good=1 or bad=0 XLs. Kfold option can be set for 5 to 20-fold cross validation. The output here is a file named top_XLs.txt contains top XLs from MS1 analysis and is the main output of the whole analysis.
-training_set.csv is the training set to train machine learning algorithm. This file is prepared according to known XLs and the features they generate and is already provided inside the package.

[Outputs] top_XLs.txt

The .txt file contains top XLs in kojak format.

MS2 analysis: MS2 analysis works based on making theoretical fragments and computing m/z for them to investigate MS2 data for finding the best matched spectra (see Supplementary Note 4). The inputs/outputs, requirements, and all the commands to use MS1 analysis are as follow:

[Commands]

1- *python ./ms2_analysis.py -x XLs.txt -m ms2.mgf -d 0.01*

This command run the MS2 analysis for the input set of XLs over the .mgf file to find the top spectra.
-ms2.mgf is the general MS2 output data converted to mgf format.
-XLs.txt is a file containing all the XLs supposed to be considered with MS2 analysis. They could be the output of seq2xl to consider all possible XLs or the output of MS1 analysis to consider only top_XLs from MS1.
-delta is an integer number between 0.01 and 0.05 and defines the peak detection window.

[Outputs] top_spectra_images.png, ms2_data.sqlite

For all selected XLs a .png figure will be generated to show the quality of detected spectra. Meanwhile, a sqlite library contains all the required information such as score, detected fragments, charge value, precursor mass/z and etc (see Supplementary Note 4).

Data availability: We also provide both MS data and modeling data under the following URL:

https://doi.org/10.5281/zenodo.1438111

Supplementary Note 8

M1-C4BPa interaction

Expression and purification of TEV-cleaved M1 protein

The S. pyogenes open reading frame (amino acids 42-484) encoding for the M1 protein (UniProt ID: Q99XV0, emm1) was cloned at the Lund Protein Production Platform (LP3) (Lund, Sweden) as described in the main manuscript text. The M1 protein was expressed at the LP3 in E.coli TUNER (DE3) cells in Terrific Broth (TB) (Difco) supplemented with 50 uG/mL of kanamycin at 30° C. Protein expression was induced with 1 mM IPTG at OD600 0.65. The expressed cells were harvested in a JLA 8.100 rotor, 6000 g, 4° C, 20 min, resuspended in 60 ml of 50 mM NaPO4, 300 mM NaCl, 20 mM imidazole, pH 8.0 supplemented with EDTA-free Complete Protease Inhibitor tablets (Roche), and passed twice through a French Pressure Cell at 18000 psi. The resulting lysate was ultracentrifuged in a Ti 50.2 rotor, 45000 rpm, 60 min, 4° C, and subsequently passed through a 0.22 mm filter prior to loading the filtered supernatant on a HisTrap HP column (GE Healthcare). The column was washed with 50 mM NaPO4, 300 mM NaCl, 20 mM imidazole, pH 8.0, and bound protein was eluted using a gradient of 20-500 mM imidazole in 50 mM NaPO4, 300 mM NaCl, pH 8.0. The peak fractions were pooled and mixed with Tobacco Mosaic Etch Virus (TEV) protease at an enzyme:substrate mass ratio of 1:10 supplemented with 1 mM DTT to produce recombinant M1 protein without the affinity-tag attached. The digestion mixture was dialyzed against 50 mM NaPO4, 300 mM NaCl, 20 mM imidazole, 1 mM DTT, pH 8.0 in a 3500 MWCO dialysis tube. The dialyzed protein preparation was reapplied onto a HisTrap HP column, and washed with 50 mM NaPO4, 300 mM NaCl, 20 mM imidazole, pH 8.0. The TEV cleaved M1 protein was as expected found in the flow through and wash fractions, as removal of the affinity-tag prevents binding. These fractions were pooled, concentrated, and passed through a 0.22 mm filter prior to loading the sample on a HiLoad 26/600 Superdex 75 size exclusion column (GE Healthcare) in 1xPBS, pH 7.4. The peak fractions were collected, concentrated using Millipore Amicon 3000 Da molecular weight cutoff concentrators and the purified TEV-cleaved M1 protein was stored at -80° C until usage.

Commercial proteins

Complement system C4-binding protein (C4BP) was obtained from Complement Technology.

Cross-linking of TEV-cleaved M1 protein and C4BP

Cross-linking reactions were done essentially as described in the main manuscript text with a few modifications. Briefly, 10 μG of purified TEV-cleaved M1 protein was incubated with 10 μG of C4BP in 1 x PBS pH 7.4 at 37° C 850 rpm 30 min. Heavy/light DSS (DSS-H12/D12, Creative Molecules Inc., www. creativemolecules.com) resuspended in dimethyl sulfoxide (DMSO) was added to final concentrations of 500 or 1000 μM and incubated for a further of 30 min at 37° C 850 rpm. The reactions were subsequently quenched and digested for mass spectrometry as described in the main manuscript text, except for that here SOLAμ HRP plates (Thermo Scientific) were used for peptide purification.

Liquid Chromatography-Mass Spectrometry

MS measurements on crosslinked M1-C4BP samples were performed on a Q Exactive HFX (Thermo Scientific) connected to an EASY-nLC 1200 liquid chromatography system (Thermo Scientific). Peptides were separated on an EASY-Spray column (Thermo Scientific; ID 75 μm x 50 cm, column temperature 45° C) operated at a constant pressure of 900 bar. A two-step gradient of buffer B (80% acetonitrile, 0.1% formic acid) in buffer A (aqueous 0.1% formic acid) was applied at a flow rate of 300 nl min-1. In the first step a gradient of 10 to 30% of buffer B was run for 45 min followed by a 30 to 45% gradient of buffer B in 10 min. One full MS scan (resolution 60,000 @ 200 m/z; mass range 350?1,400m/z) was followed by MS/MS scans (resolution 15,000 @ 200 m/z). The precursor ions were isolated with 1.3 m/z isolation width and fragmented using higher-energy collisional-induced dissociation at a normalized collision energy of 27. Charge state screening was enabled, and singly charged ions as well as precursors with a charge state above 6 were rejected. The dynamic exclusion window was set to 10 s. The automatic gain control was set to 3×10^6 for MS and 1×10^5 for MS/MS with ion accumulation times of 45 ms and 60 ms, respectively. The intensity threshold for precursor ion selection was set to 1.7×10^4.

Data analysis

By considering all computational XLs (all possible K-K pairs equal to 2788 XLs) for M1-C4BPa (inter XLs) and C4BPa itself (intra XLs), we investigated all DDA spectra with 0.01 narrow delta window for both precursor and fragments m/z. In results, we found 14 new XLs contain 6 inter and 8 intra XLs. 3 out of 6 new inter XLs support three XLs that we found in plasma samples overlapping the same binding interface completely on both M1 (hyper variable region) and C4BPa and therefore justify the model we proposed in Figure 3 of the article. The 3 others support the same binding interface on M1 but different spot on C4BPa. As there is no crystal structure for the full length C4BPa, we can not validate these two, but we report them as they might be helpful for further investigation on this structure in the future. Supplementary Figure **11** shows a high accuracy model (the same model as presented in Figure 3) with these 5 new inter/intra XLs together with three XLs we found on Plasma sample experiment mapped on the structure. Supplementary Table **9** provides more details about these XLs such as the exact peptide, the position of Lysin on the sequence, the Euclidean distance, and etc. Supplementary Figures **12** to **14** show the supporting MS2 spectra of these 14 XLs.

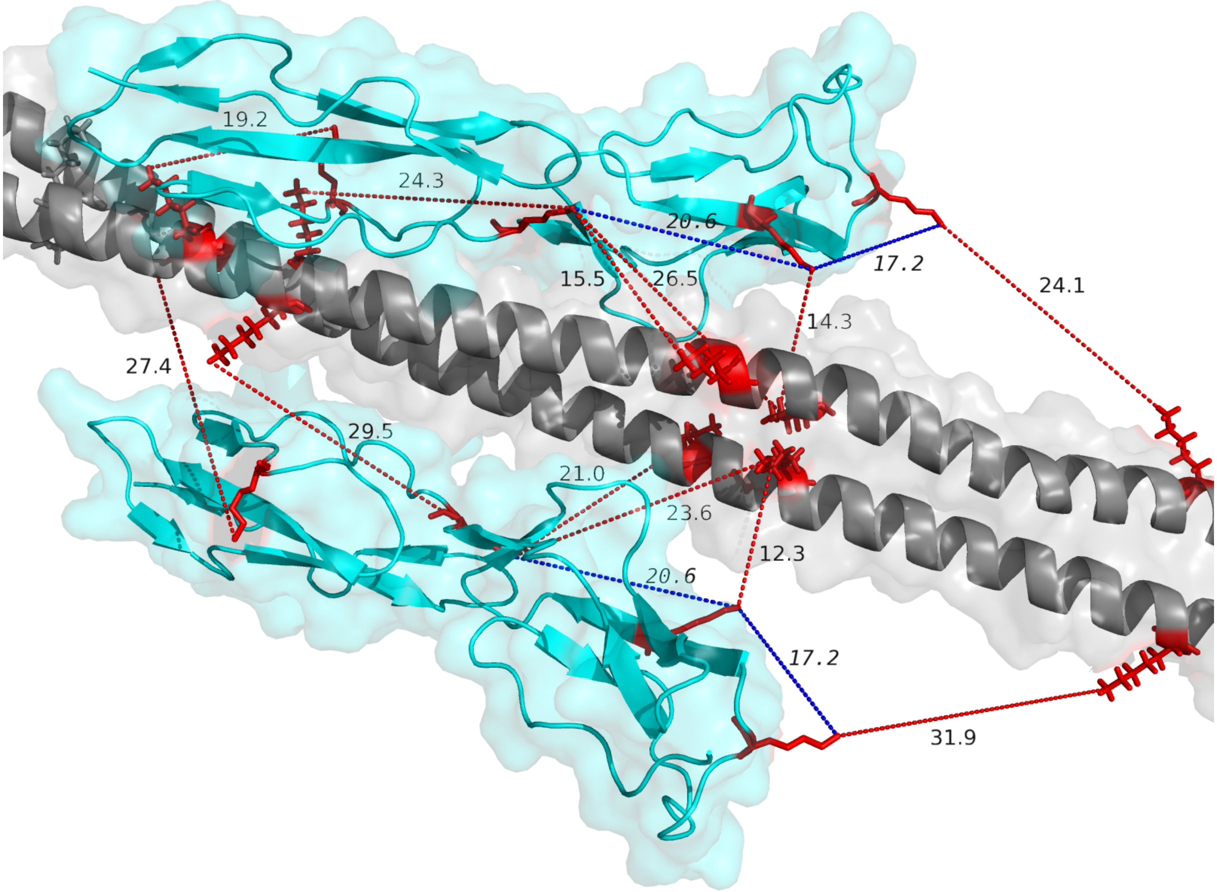

Supplementary Figure 11: High accuracy model of M1-C4BPa. Inter and intra XLs are mapped on the structure in red and blue colors while M1 is in gray and C4BPa is in cyan. The length of the XLs are shown in Angstrom (Å) which are all within acceptable distance.

Supplementary Table 9: More details on 14 new inter/intra XLs for M1-C4BPa. The distance is computed according to the model that we predicted for M1 and partial structure of C4BPa.

**XLs**	**K1_seq position**	**K2_seq position**	**distance (Å)**	**type**
**-.ELEEKK(5)–TGTTLKYTCLPGYVR(6).-**	99	78	14.3	inter XLs
**-.ELEEKK(5)–SHSTQTLTCNSDGEWVYNTFCIYKR(24).-**	99	111	26.5	inter XLs
**-.ELEEKK(5)–MALEVYKLSLEIEQLELQR(7).-**	99	573	–	inter XLs
**-.DLETKLK(5)–SHSTQTLTCNSDGEWVYNTFCIYKR(24).-**	70	111	29.5	inter XLs
**-.KALELAIDQASQDYNR(1)–KPELVNGR(1).-**	129	486	–	inter XLs
**-.YENKDLK(4)–PTTDEPTTVICQKNLR(13).-**	30	350	–	inter XLs
**-.KPELVNGR(1)–LSCSYSHWSAPAPQCKALCR(16).-**	486	481	–	intra XLs
**-.FKTGTTLK(2)–TGTTLKYTCLPGYVR(6).-**	72	78	17.2	intra XLs
**-.EEIIYECDKGYILVGQAK(9)–IAHGHYKQSSSYSFFK(7).-**	456	438	–	intra XLs
**-.EEIIYECDKGYILVGQAK(9)–LSCSYSHWSAPAPQCKALCR(16).-**	456	481	–	intra XLs
**-.QSTLDKEL(6)–CEWETPEGCEQVLTGKR(16).-**	595	553	–	intra XLs
**-.TGTTLKYTCLPGYVR(6)–WTPYQGCEALCCPEPKLNNGEITQHR(16).-**	78	369	–	intra XLs
**-.SHSTQTLTCNSDGEWVYNTFCIYKR(24)–TGTTLKYTCLPGYVR(6).-**	111	78	20.6	intra XLs
**-.CEWETPEGCEQVLTGKR(16)–LMQCLPNPEDVKMALEVYK(12).-**	553	566	–	intra XLs

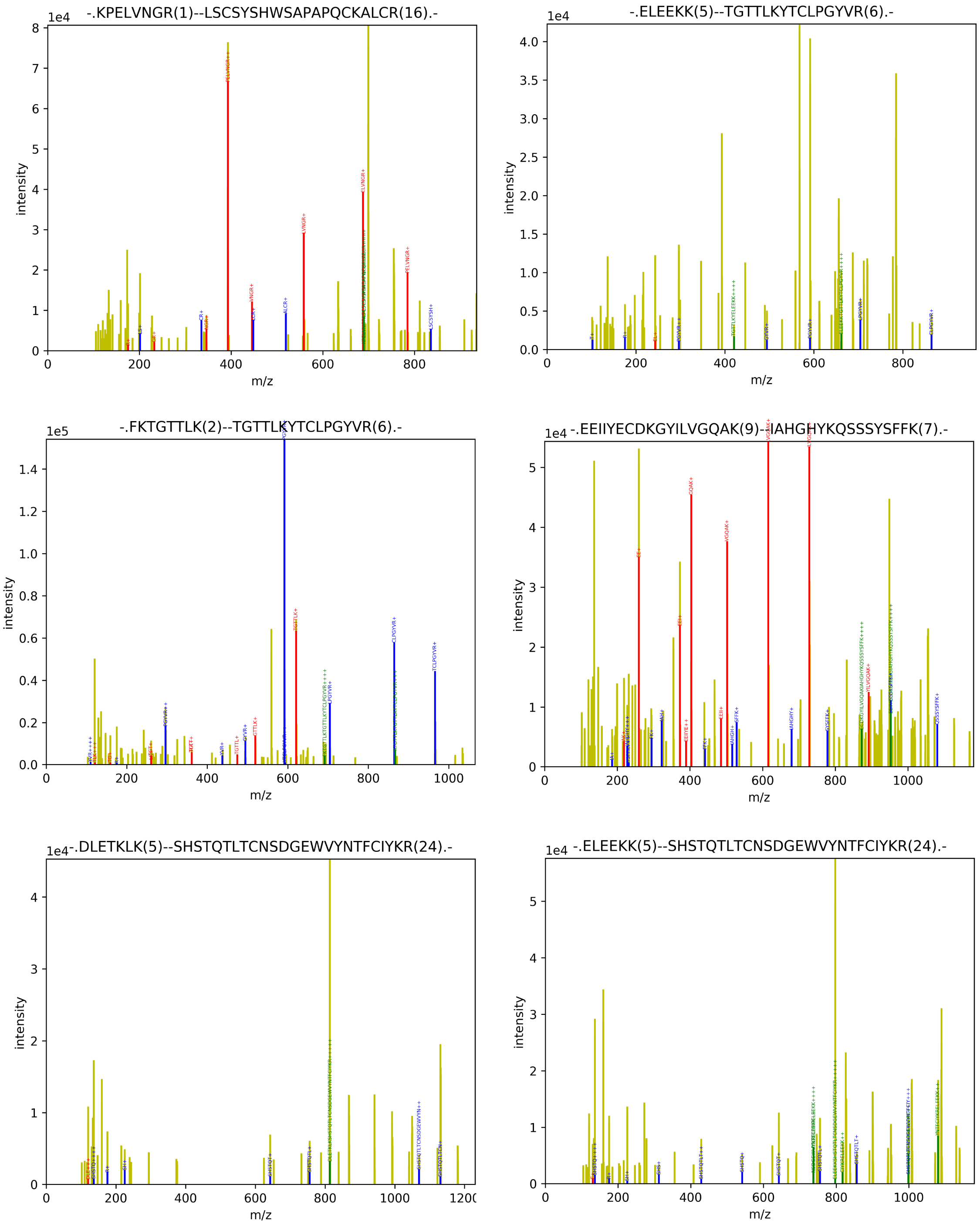

Supplementary Figure 12: MS2 spectra.

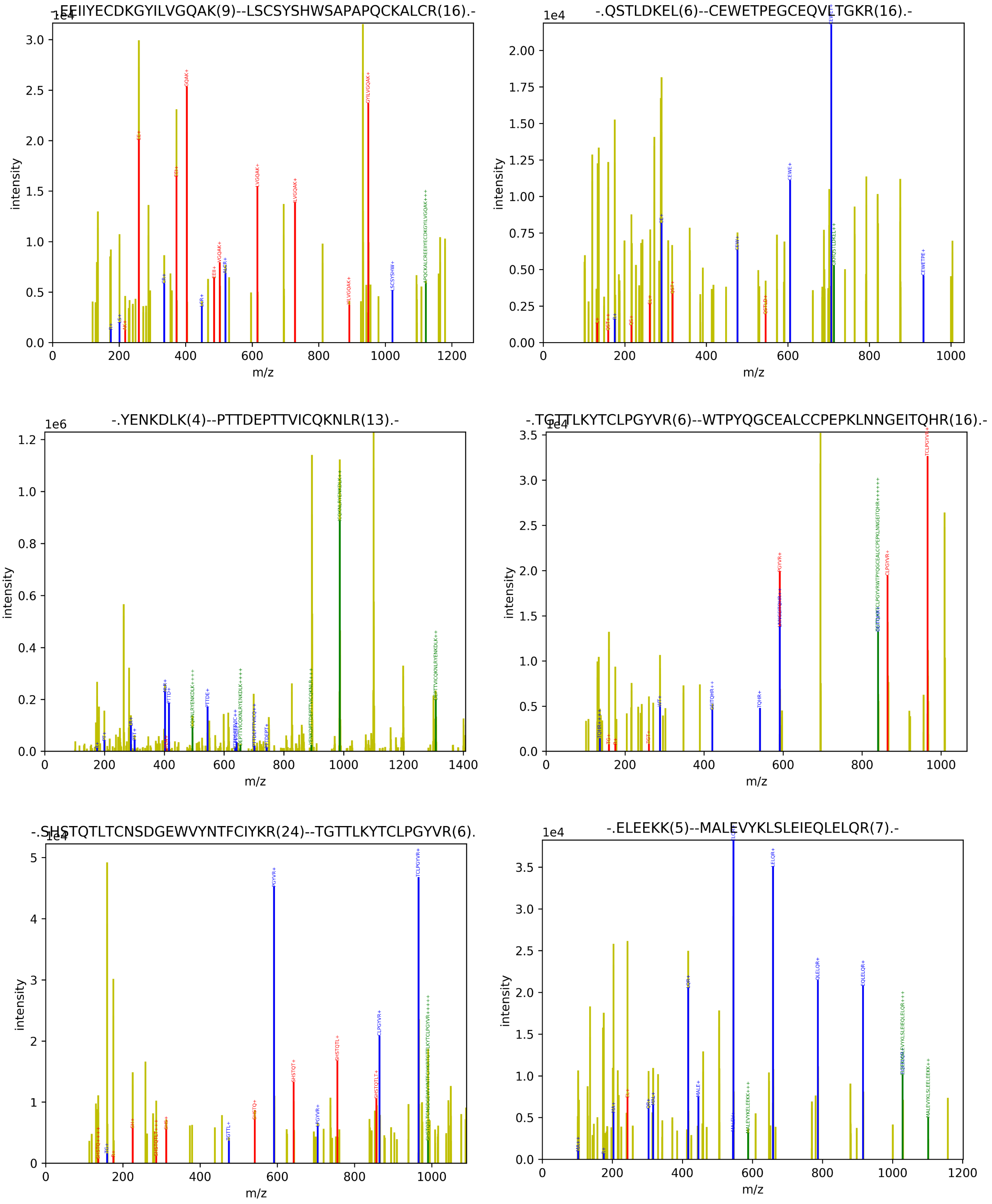

Supplementary Figure 13: MS2 spectra.

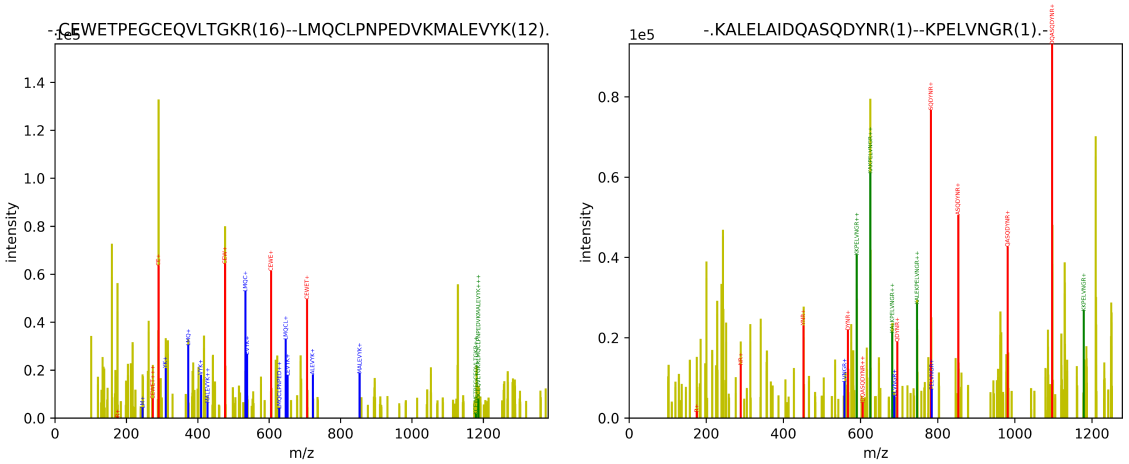

Supplementary Figure 14: MS2 spectra.

Supplementary References

[1] Yifan Song, Frank DiMaio, Ray Yu-Ruei Wang, David Kim, Chris Miles, TJ Brunette, James Thompson, and David Baker. High resolution comparative modeling with rosettacm. *Structure (London, England : 1993)*, 21(10):10.1016/j.str.2013.08.005, 10 2013. doi: 10.1016/j.str.2013.08.005. URL http://www.ncbi.nlm.nih.gov/pmc/articles/PMC3811137/.

[2] N. Eswar. Comparative protein structure modeling using modeller. *Curr. Protoc. Bioinformatics*, Chapter 5, 2006.

[3] D. Xu. Automated protein structure modeling in casp9 by i-tasser pipeline combined with quark-based ab initio folding and fg-md-based structure refinement. *Proteins*, 79:147–160, 2011.

[4] S.F. Altschul. Basic local alignment search tool. *J. Mol. Biol.*, 215:403–410, 1990.

[5] M. Remmert. Hhblits: lightning-fast iterative protein sequence searching by hmm-hmm alignment. *Nat. Methods*, 9:173–175, 2012.

[6] R. Das. Macromolecular modeling with rosetta. *Annu. Rev. Biochem.*, 77:363–382, 2008.

[7] Philip Bradley, Lars Malmström, Bin Qian, Jack Schonbrun, Dylan Chivian, David E. Kim, Jens Meiler, Kira M.S. Misura, and David Baker. Free modeling with rosetta in casp6. *Proteins: Structure, Function, and Bioinformatics*, 61(S7):128–134, 2005. ISSN 1097-0134. doi: 10.1002/prot.20729. URL http://dx.doi.org/10.1002/prot.20729.

[8] Daniela Rothlisberger, Olga Khersonsky, Andrew M. Wollacott, Lin Jiang, Jason DeChancie, Jamie Betker, Jasmine L. Gallaher, Eric A. Althoff, Alexandre Zanghellini, Orly Dym, Shira Albeck, Kendall N. Houk, Dan S. Tawfik, and David Baker. Kemp elimination catalysts by computational enzyme design. *Nature*, 453 (7192):190–195, 05 2008. URL http://dx.doi.org/10.1038/nature06879.

[9] Brian Kuhlman, Gautam Dantas, Gregory C. Ireton, Gabriele Varani, Barry L. Stoddard, and David Baker. Design of a novel globular protein fold with atomic-level accuracy. *Science*, 302(5649):1364–1368, 2003. ISSN 0036-8075. doi: 10.1126/science.1089427. URL http://science.sciencemag.org/content/302/5649/1364.

[10] Neil P. King, William Sheffler, Michael R. Sawaya, Breanna S. Vollmar, John P. Sumida, Ingemar André, Tamir Gonen, Todd O. Yeates, and David Baker. Computational design of self-assembling protein nanomaterials with atomic level accuracy. *Science*, 336(6085):1171–1174, 2012. ISSN 0036-8075. doi: 10.1126/science.1219364. URL http://science.sciencemag.org/content/336/6085/1171.

[11] Lars Malmström, Michael Riffle, Charlie E M Strauss, Dylan Chivian, Trisha N Davis, Richard Bonneau, and David Baker. Superfamily assignments for the yeast proteome through integration of structure prediction with the gene ontology. *PLoS Biology*, 5(4):e76, 04 2007. doi: 10.1371/journal.pbio.0050076. URL http://www.ncbi.nlm.nih.gov/pmc/articles/PMC1828141/.

[12] Kevin Drew, Patrick Winters, Glenn L Butterfoss, Viktors Berstis, Keith Uplinger, Jonathan Armstrong, Michael Riffle, Erik Schweighofer, Bill Bovermann, David R Goodlett, Trisha N Davis, Dennis Shasha, Lars Malmström, and Richard Bonneau. The proteome folding project: Proteome-scale prediction of structure and function. *Genome Research*, 21(11):1981–1994, 11 2011. doi: 10.1101/gr.121475.111. URL http://www.ncbi.nlm.nih.gov/pmc/articles/PMC3205581/.

[13] Jeffrey J Gray. High-resolution protein–protein docking. *Current Opinion in Structural Biology*, 16 (2):183 – 193, 2006. ISSN 0959-440X. doi: http://dx.doi.org/10.1016/j.sbi.2006.03.003. URL http://www.sciencedirect.com/science/article/pii/S0959440X0600042X. Theory and simulation/Macromolecular assemblagesJoel Janin and Michael Levitt/Edward H Egelman and Andrew {GW} Leslie.

[14] Sidhartha Chaudhury, Monica Berrondo, Brian D. Weitzner, Pravin Muthu, Hannah Bergman, and Jeffrey J. Gray. Benchmarking and analysis of protein docking performance in rosetta v3.2. *PLoS ONE*, 6(8):1–13, 08 2011. doi: 10.1371/journal.pone.0022477. URL http://dx.doi.org/10.1371%2Fjournal.pone.0022477.

[15] Chu Wang, Ora Schueler-Furman, and David Baker. Improved side-chain modeling for protein–protein docking. *Protein Science*, 14(5):1328–1339, 2005. ISSN 1469-896X. doi: 10.1110/ps.041222905. URL http://dx.doi.org/10.1110/ps.041222905.

[16] Leo Breiman. Bagging predictors. *Machine Learning*, 24(2):123–140, Aug 1996. ISSN 1573-0565. doi: 10.1007/BF00058655. URL http://dx.doi.org/10.1007/BF00058655.

[17] Hamed R. Bonab and Fazli Can. A theoretical framework on the ideal number of classifiers for online ensembles in data streams. In *Proceedings of the 25th ACM International on Conference on Information and Knowledge Management*, CIKM '16, pages 2053–2056, New York, NY, USA, 2016. ACM. ISBN 978-1-4503-4073-1. doi: 10.1145/2983323.2983907. URL http://doi.acm.org/10.1145/2983323.2983907.

[18] Anton A. Goloborodko, Lev I. Levitsky, Mark V. Ivanov, and Mikhail V. Gorshkov. Pyteomics—a python framework for exploratory data analysis and rapid software prototyping in proteomics. *Journal of The American Society for Mass Spectrometry*, 24(2):301–304, Feb 2013. ISSN 1879-1123. doi: 10.1007/s13361-012-0516-6. URL http://dx.doi.org/10.1007/s13361-012-0516-6.

[19] Hannes L Rost, George Rosenberger, Pedro Navarro, Ludovic Gillet, Sasa M Miladinovic, Olga T Schubert, Witold Wolski, Ben C Collins, Johan Malmstrom, Lars Malmstrom, and Ruedi Aebersold. Openswath enables automated, targeted analysis of data-independent acquisition ms data. *Nat Biotech*, 32(3):219–223, 03 2014. URL http://dx.doi.org/10.1038/nbt.2841.

[20] Pauline Macheboeuf, Cosmo Buffalo, Chi-yu Fu, Annelies S. Zinkernagel, Jason N. Cole, John E. Johnson, Victor Nizet, and Partho Ghosh. Streptococcal m1 protein constructs a pathological host fibrinogen network. *Nature*, 472(7341):64–68, 04 2011. URL http://dx.doi.org/10.1038/nature09967.

[21] M. Delorenzi. An hmm model for coiled-coil domains and a comparison with pssm-based predictions. *bioinformatics*, 18(4):617–625, 2002.

[22] A. Lupas. Predicting coiled coils from protein sequences. *Science*, 252:1162–1164, 1999.

[23] Anna Lobley. pgenthreader and pdomthreader. *Bioinformatics*, 25(14):1761–1767, 2009.
