# Supplementary material for: Rapid determination of quaternary protein structures in complex biological samples
Source: Nat Commun. 2019 Jan 14;10:192. doi: 10.1038/s41467-018-07986-1 (PMC6331586; doi:10.1038/s41467-018-07986-1)
Supplement: Supplementary file 2 — Description of Additional Supplementary Files [file 41467_2018_7986_MOESM2_ESM.docx]

**Description of Additional Supplementary Files**

**File Name**: Supplementary Data 1

**Description**: Data from 76 XLs identified by hrMS1, MS2, and DIA. Each XL is presented in three panels with the reference peptides (in red and blue) and the protein name (in black) above. Panel a) shows MS2 spectra selected by Taxlink. Fragments in red are related to the first peptide, fragments in blue are related to the second peptide and the green contain the cross-linker. Panel b) shows the DIA analysis with the same color scheme. Panel c) contains the MS1 analysis data.
