# Supplementary material for: Rapid determination of quaternary protein structures in complex biological samples
Source: Nat Commun. 2019 Jan 14;10:192. doi: 10.1038/s41467-018-07986-1 (PMC6331586; doi:10.1038/s41467-018-07986-1)
Supplement: Supplementary file 3 — Supplementary Data 1 [file 41467_2018_7986_MOESM3_ESM.pdf]

Supplementary Data 1

XL identification by three different acquisition methods.

According to the three orthogonal acquisition methods, 76 XLs are selected which have the support of all approaches together. Each figure below contains three parts and titled with the reference peptides (in red and blue) and the protein names (in black). Part **a** shows MS2 spectra selected from Taxlink MS2 analysis package. Fragments in red are related to the first peptide, fragments in blue are related to the second peptide and the green ones, contain the cross-linker arm. Part **b** shows the DIA analysis with the same color schema. Finally, part **c** contains the MS1 analysis data.

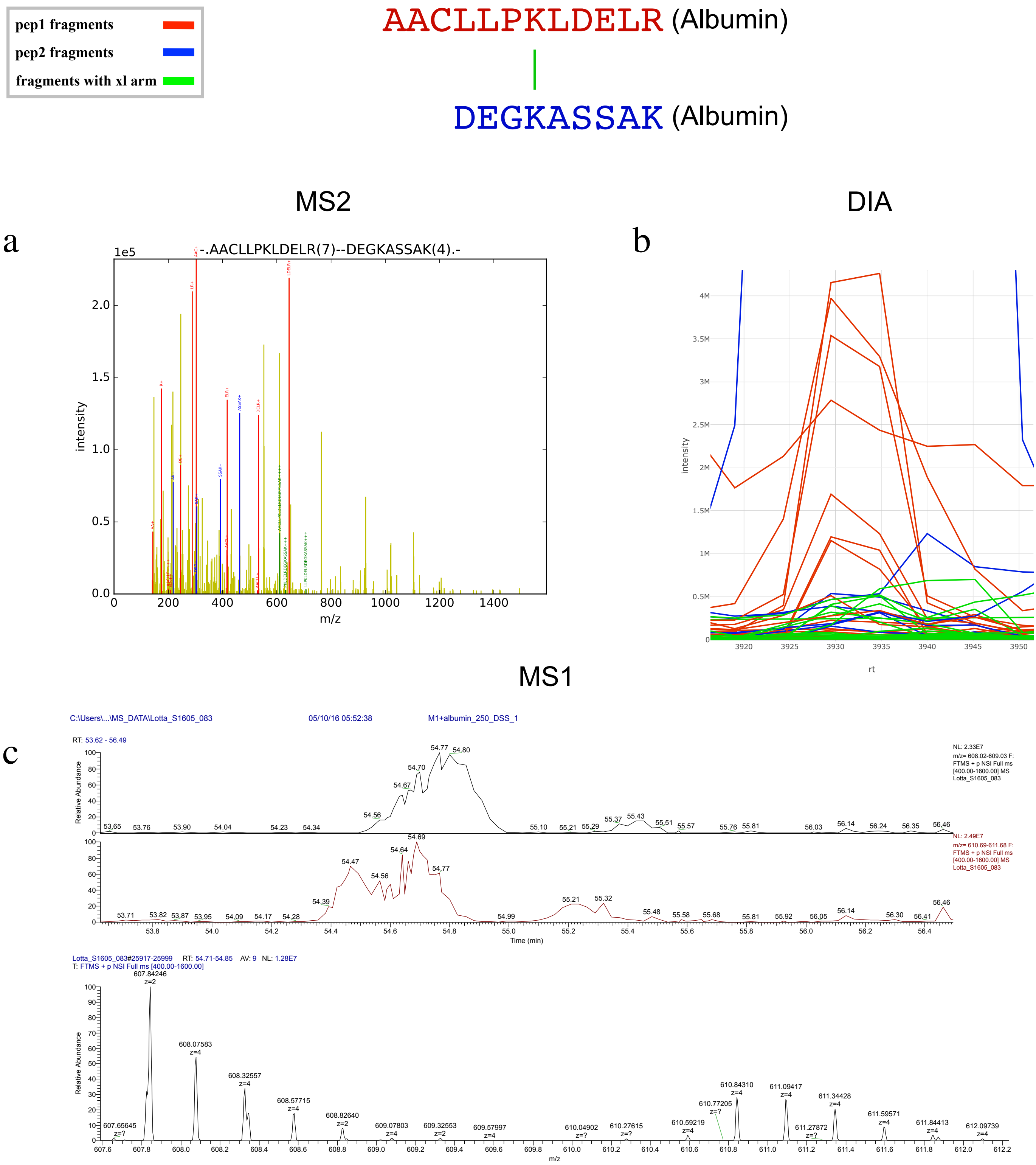

pep1 fragments
pep2 fragments
fragments with xl arm
AACLLPKLDELR (Albumin)
DEGKASSAK (Albumin)
MS2
DIA
a
-.AACLLPKLDELR(7)--DEGKASSAK(4).-
1e5
intensity
m/z
b
intensity
rt
MS1
c
C:\Users\...\MS_DATA\Lotta_S1605_083
05/10/16 05:52:38
M1+albumin_250_DSS_1
RT: 53.62 - 56.49
Relative Abundance
NL: 2.33E7
m/z= 608.02-609.03 F: FTMS + p NSI Full ms [400.00-1600.00] MS Lotta_S1605_083
NL: 2.49E7
m/z= 610.69-611.68 F: FTMS + p NSI Full ms [400.00-1600.00] MS Lotta_S1605_083
Time (min)
Lotta_S1605_083#25917-25999 RT: 54.71-54.85 AV: 9 NL: 1.28E7
T: FTMS + p NSI Full ms [400.00-1600.00]
Relative Abundance
m/z

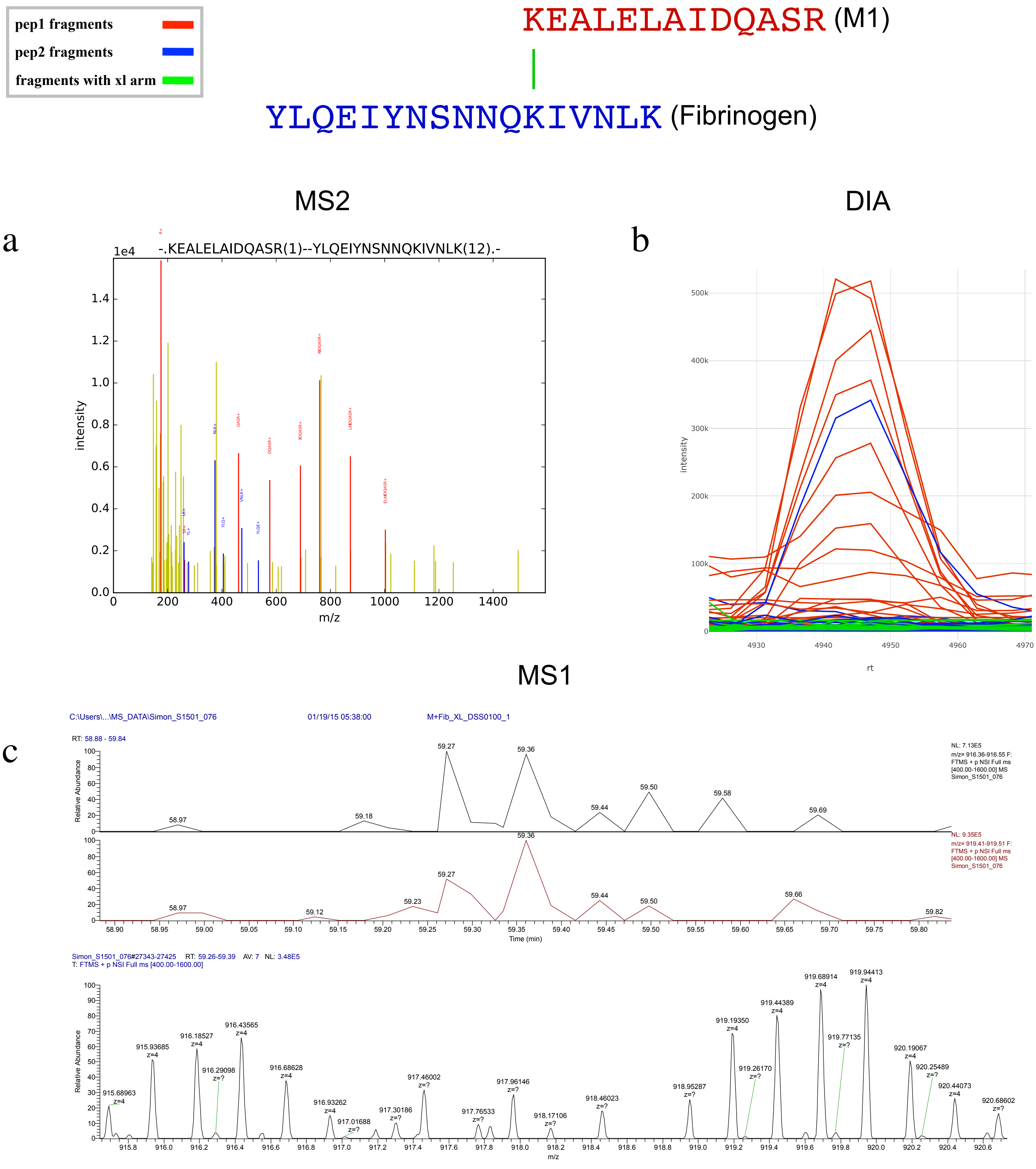

pep1 fragments
pep2 fragments
fragments with xl arm
KEALELAIDQASR (M1)
YLQEIYNSNNQKIVNLK (Fibrinogen)
MS2
DIA
a
-.KEALELAIDQASR(1)--YLQEIYNSNNQKIVNLK(12).-
1e4
intensity
m/z
b
intensity
rt
MS1
c
C:\Users\...\MS_DATA\Simon_S1501_076
01/19/15 05:38:00
M+Fib_XL_DSS0100_1
RT: 58.88 - 59.84
NL: 7.13E5
m/z= 916.36-916.55 F: FTMS + p NSI Full ms [400.00-1600.00] MS Simon_S1501_076
NL: 9.35E5
m/z= 919.41-919.51 F: FTMS + p NSI Full ms [400.00-1600.00] MS Simon_S1501_076
Relative Abundance
Time (min)
Simon_S1501_076#27343-27425 RT: 59.26-59.39 AV: 7 NL: 3.48E5
T: FTMS + p NSI Full ms [400.00-1600.00]
Relative Abundance
m/z

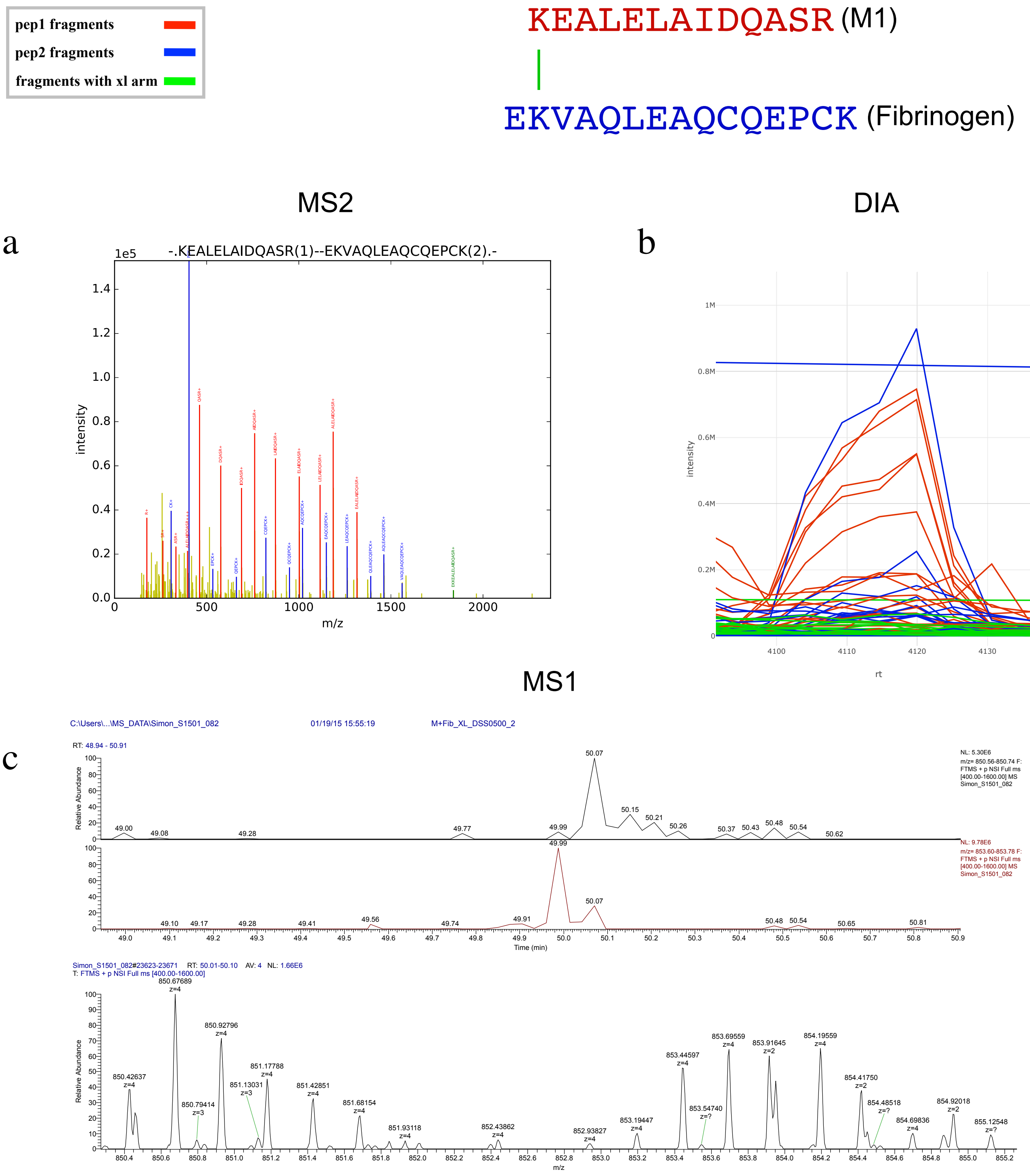

pep1 fragments
pep2 fragments
fragments with xl arm
KEALELAIDQASR (M1)
EKVAQLEAQCQEPCK (Fibrinogen)
MS2
DIA
a
b
-.KEALELAIDQASR(1)--EKVAQLEAQCQEPCK(2).-
intensity
m/z
rt
MS1
c
C:\Users\...\MS_DATA\Simon_S1501_082
01/19/15 15:55:19
M+Fib_XL_DSS0500_2
RT: 48.94 - 50.91
Relative Abundance
Time (min)
NL: 5.30E6
m/z= 850.56-850.74 F: FTMS + p NSI Full ms [400.00-1600.00] MS Simon_S1501_082
NL: 9.78E6
m/z= 853.60-853.78 F: FTMS + p NSI Full ms [400.00-1600.00] MS Simon_S1501_082
Simon_S1501_082#23623-23671 RT: 50.01-50.10 AV: 4 NL: 1.66E6
T: FTMS + p NSI Full ms [400.00-1600.00]
m/z

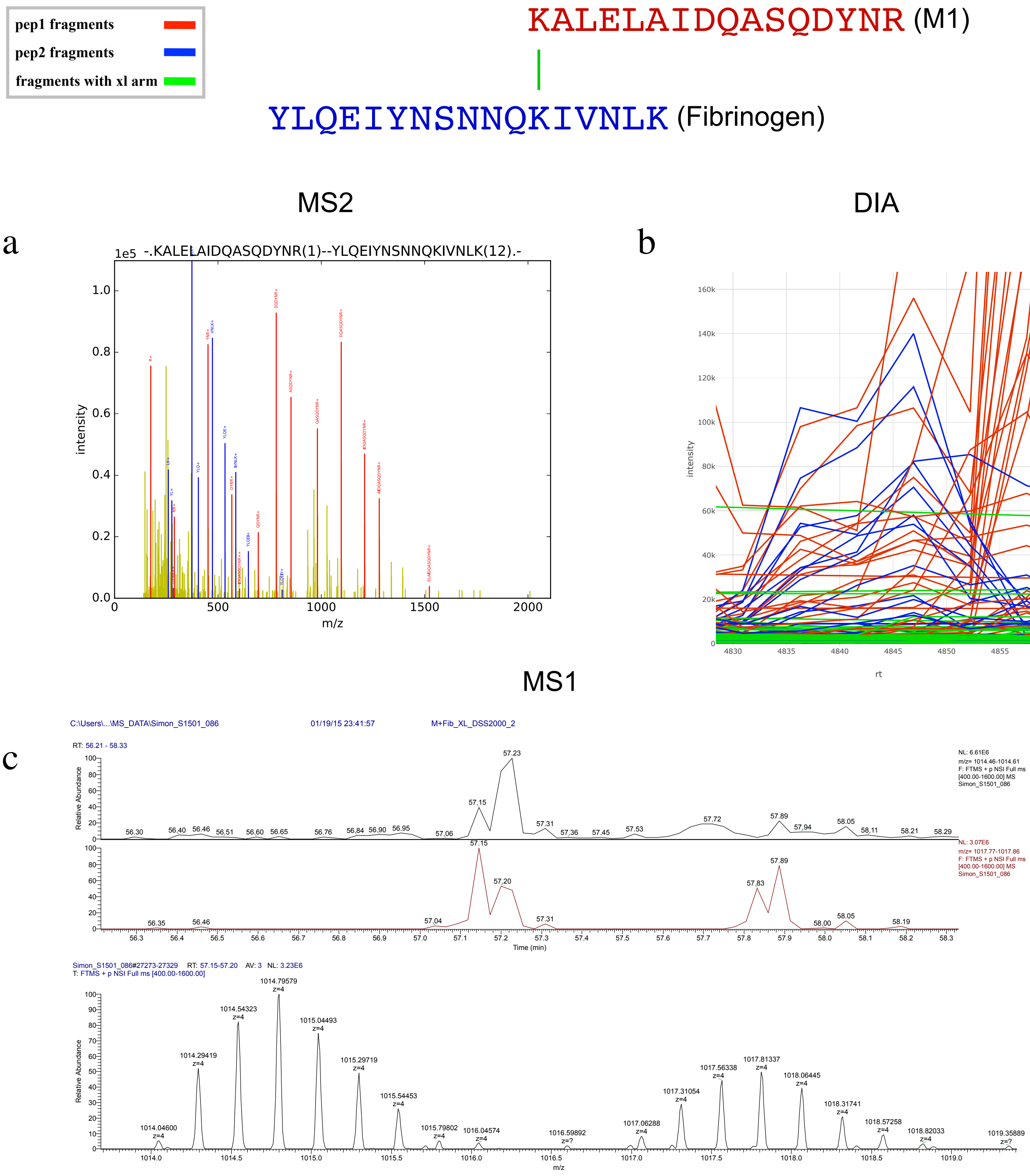

pep1 fragments
pep2 fragments
fragments with xl arm
KALELAIDQASQDYNR (M1)
YLQEIYNSNNQKIVNLK (Fibrinogen)
MS2
DIA
a
b
-.KALELAIDQASQDYNR(1)--YLQEIYNSNNQKIVNLK(12).-
1e5
intensity
m/z
rt
MS1
c
C:\Users\...\MS_DATA\Simon_S1501_086
01/19/15 23:41:57
M+Fib_XL_DSS2000_2
RT: 56.21 - 58.33
Relative Abundance
Time (min)
NL: 6.61E6
m/z= 1014.46-1014.61
F: FTMS + p NSI Full ms [400.00-1600.00] MS
Simon_S1501_086
NL: 3.07E6
m/z= 1017.77-1017.86
F: FTMS + p NSI Full ms [400.00-1600.00] MS
Simon_S1501_086
Simon_S1501_086#27273-27329 RT: 57.15-57.20 AV: 3 NL: 3.23E6
T: FTMS + p NSI Full ms [400.00-1600.00]
1014.79579 z=4
1014.54323 z=4
1015.04493 z=4
1014.29419 z=4
1015.29719 z=4
1015.54453 z=4
1014.04600 z=4
1015.79802 z=4
1016.04574 z=4
1016.59892 z=?
1017.06288 z=4
1017.31054 z=4
1017.56338 z=4
1017.81337 z=4
1018.06445 z=4
1018.31741 z=4
1018.57258 z=4
1018.82033 z=4
1019.35889 z=?
m/z

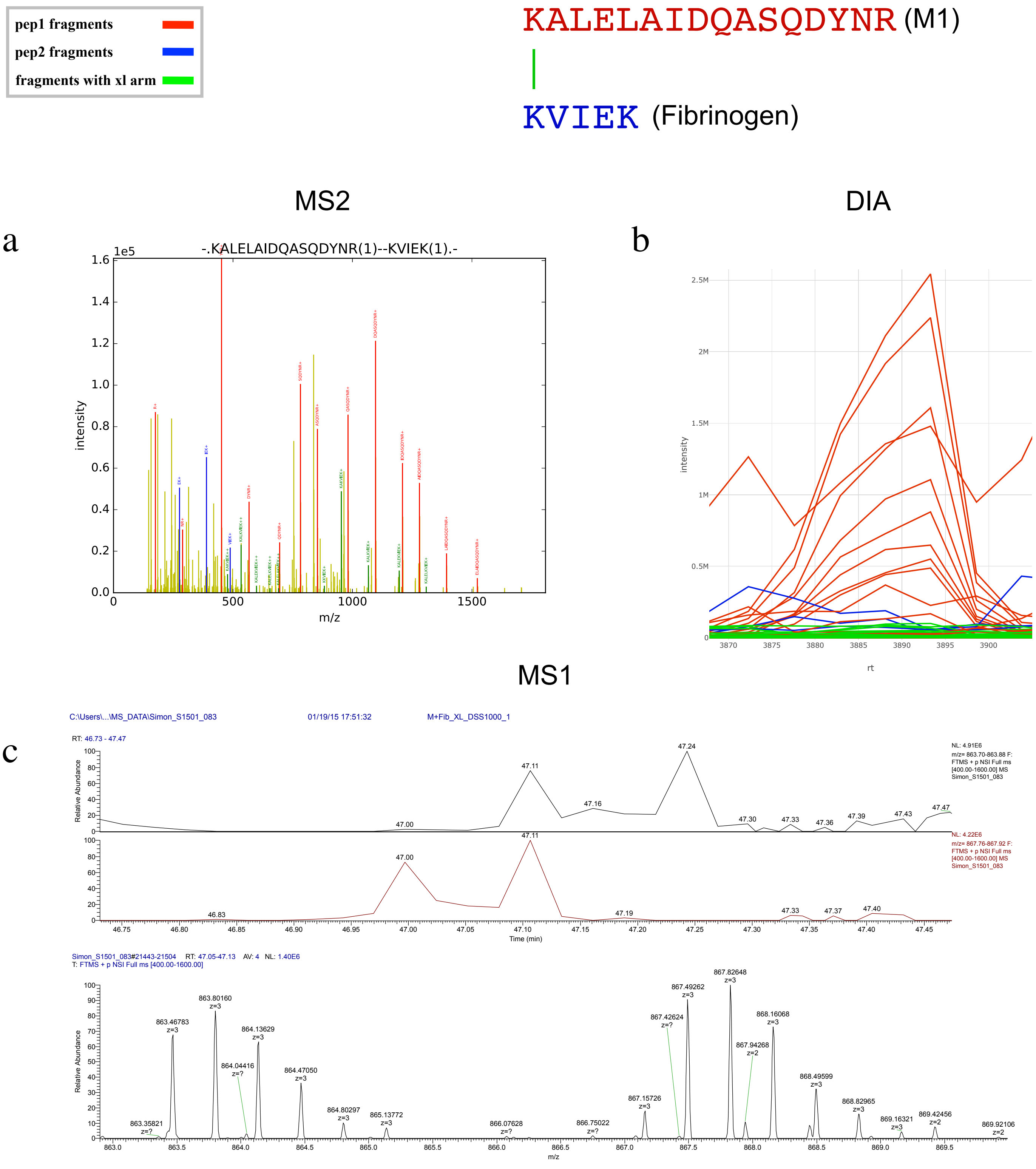
pep1 fragments
pep2 fragments
fragments with xl arm
KALELAIDQASQDYNR (M1)
KVIEK (Fibrinogen)
MS2
DIA
a
-.KALELAIDQASQDYNR(1)--KVIEK(1).-
1e5
intensity
m/z
b
intensity
rt
MS1
c
C:\Users\...\MS_DATA\Simon_S1501_083
01/19/15 17:51:32
M+Fib_XL_DSS1000_1
RT: 46.73 - 47.47
Relative Abundance
NL: 4.91E6
m/z= 863.70-863.88 F: FTMS + p NSI Full ms [400.00-1600.00] MS Simon_S1501_083
NL: 4.22E6
m/z= 867.76-867.92 F: FTMS + p NSI Full ms [400.00-1600.00] MS Simon_S1501_083
Time (min)
Simon_S1501_083#21443-21504 RT: 47.05-47.13 AV: 4 NL: 1.40E6
T: FTMS + p NSI Full ms [400.00-1600.00]
Relative Abundance
m/z

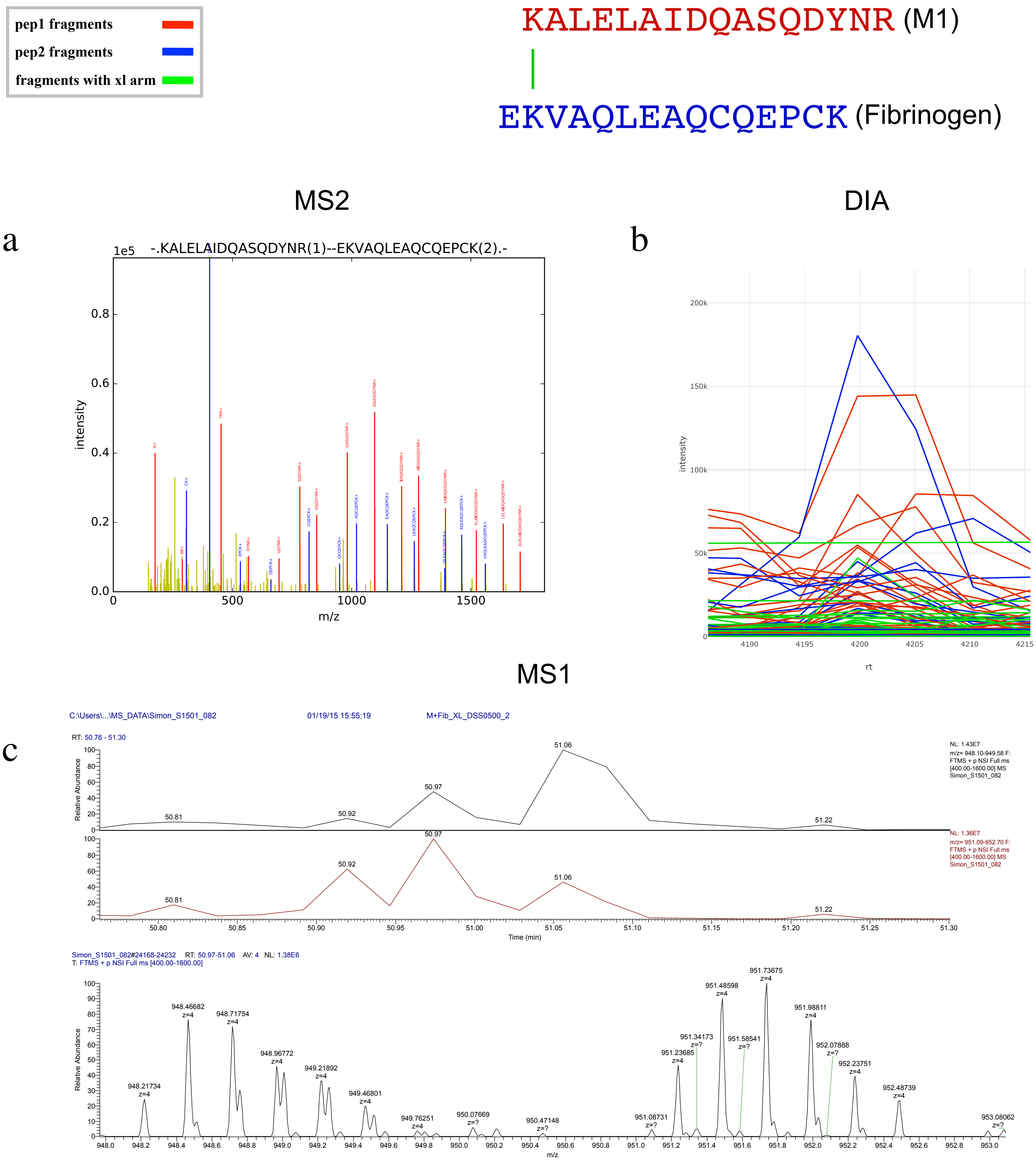

pep1 fragments
pep2 fragments
fragments with xl arm
KALELAIDQASQDYNR (M1)
EKVAQLEAQCQEPCK (Fibrinogen)
MS2
DIA
a
-.KALELAIDQASQDYNR(1)--EKVAQLEAQCQEPCK(2).-
intensity
m/z
b
intensity
rt
MS1
c
C:\Users\...\MS_DATA\Simon_S1501_082
01/19/15 15:55:19
M+Fib_XL_DSS0500_2
RT: 50.76 - 51.30
Relative Abundance
Time (min)
Simon_S1501_082#24168-24232 RT: 50.97-51.06 AV: 4 NL: 1.38E6
T: FTMS + p NSI Full ms [400.00-1600.00]
m/z

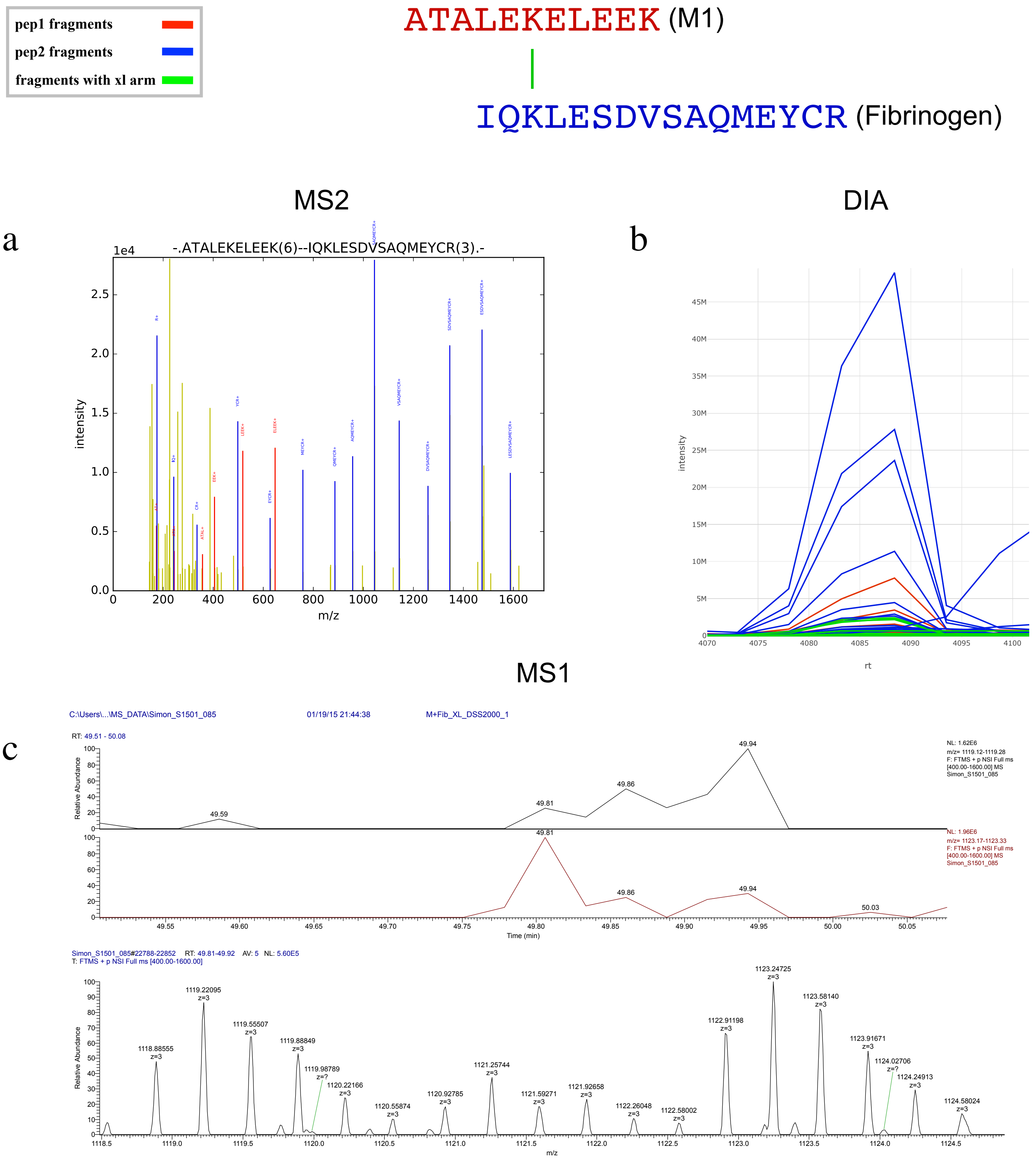

pep1 fragments
pep2 fragments
fragments with xl arm
ATALEKELEEK (M1)
IQKLESDVSAQMEYCR (Fibrinogen)
MS2
DIA
a
-.ATALEKELEEK(6)--IQKLESDVSAQMEYCR(3).-
1e4
intensity
m/z
b
intensity
rt
MS1
c
C:\Users\...\MS_DATA\Simon_S1501_085
01/19/15 21:44:38
M+Fib_XL_DSS2000_1
RT: 49.51 - 50.08
NL: 1.62E6
m/z= 1119.12-1119.28
F: FTMS + p NSI Full ms [400.00-1600.00] MS Simon_S1501_085
NL: 1.96E6
m/z= 1123.17-1123.33
F: FTMS + p NSI Full ms [400.00-1600.00] MS Simon_S1501_085
Relative Abundance
Time (min)
Simon_S1501_085#22788-22852 RT: 49.81-49.92 AV: 5 NL: 5.60E5
T: FTMS + p NSI Full ms [400.00-1600.00]
Relative Abundance
m/z

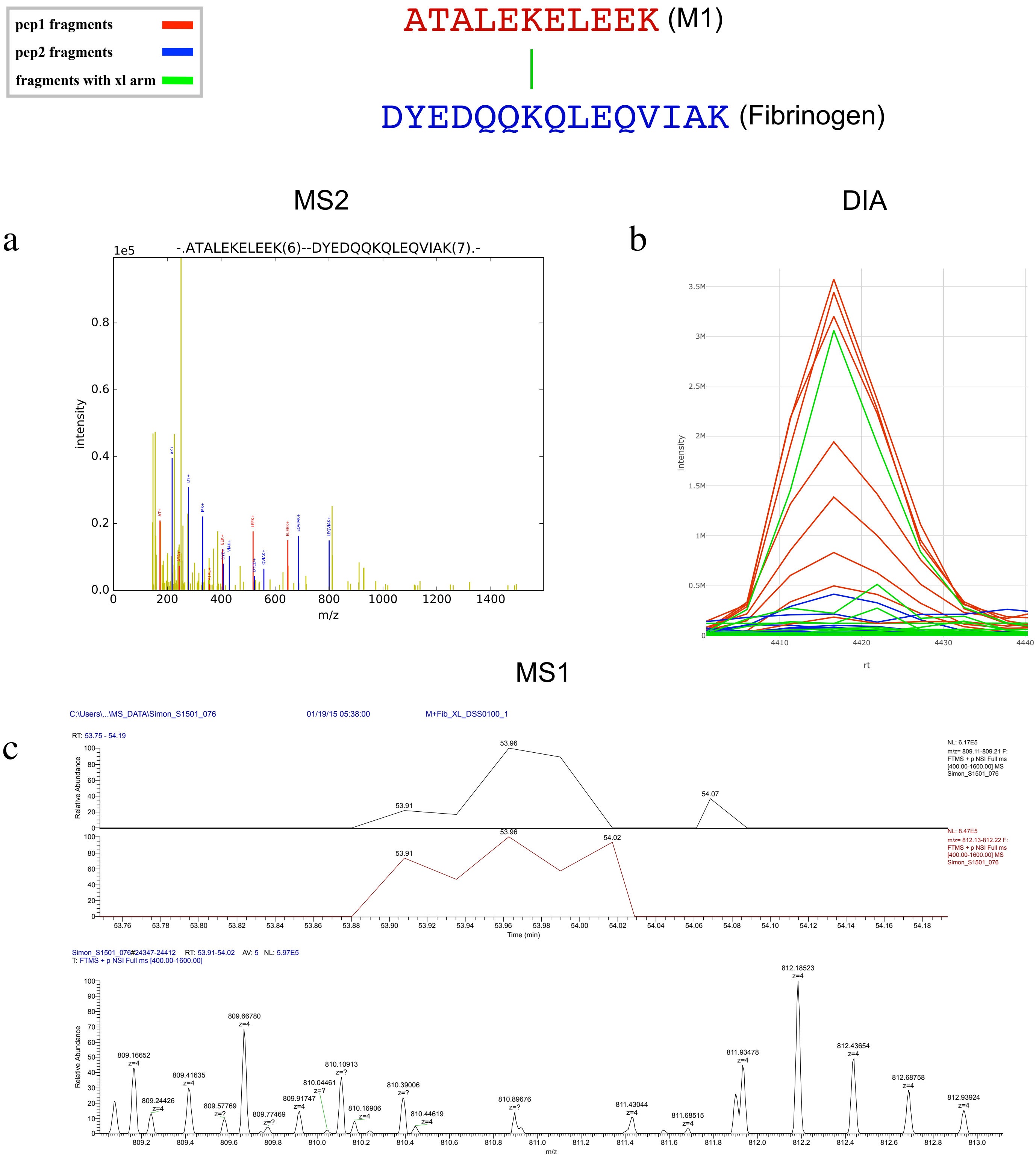
pep1 fragments
pep2 fragments
fragments with xl arm
ATALEKELEEK (M1)
DYEDQQKQLEQVIAK (Fibrinogen)
MS2
DIA
a
b
-.ATALEKELEEK(6)--DYEDQQKQLEQVIAK(7).-
1e5
intensity
m/z
rt
MS1
c
C:\Users\...\MS_DATA\Simon_S1501_076
01/19/15 05:38:00
M+Fib_XL_DSS0100_1
RT: 53.75 - 54.19
NL: 6.17E5
m/z= 809.11-809.21 F: FTMS + p NSI Full ms [400.00-1600.00] MS Simon_S1501_076
NL: 8.47E5
m/z= 812.13-812.22 F: FTMS + p NSI Full ms [400.00-1600.00] MS Simon_S1501_076
Relative Abundance
Time (min)
Simon_S1501_076#24347-24412 RT: 53.91-54.02 AV: 5 NL: 5.97E5
T: FTMS + p NSI Full ms [400.00-1600.00]
812.18523 z=4
809.66780 z=4
812.43654 z=4
811.93478 z=4
809.16652 z=4
809.41635 z=4
810.10913 z=?
812.68758 z=4
810.39006 z=?
812.93924 z=4
m/z

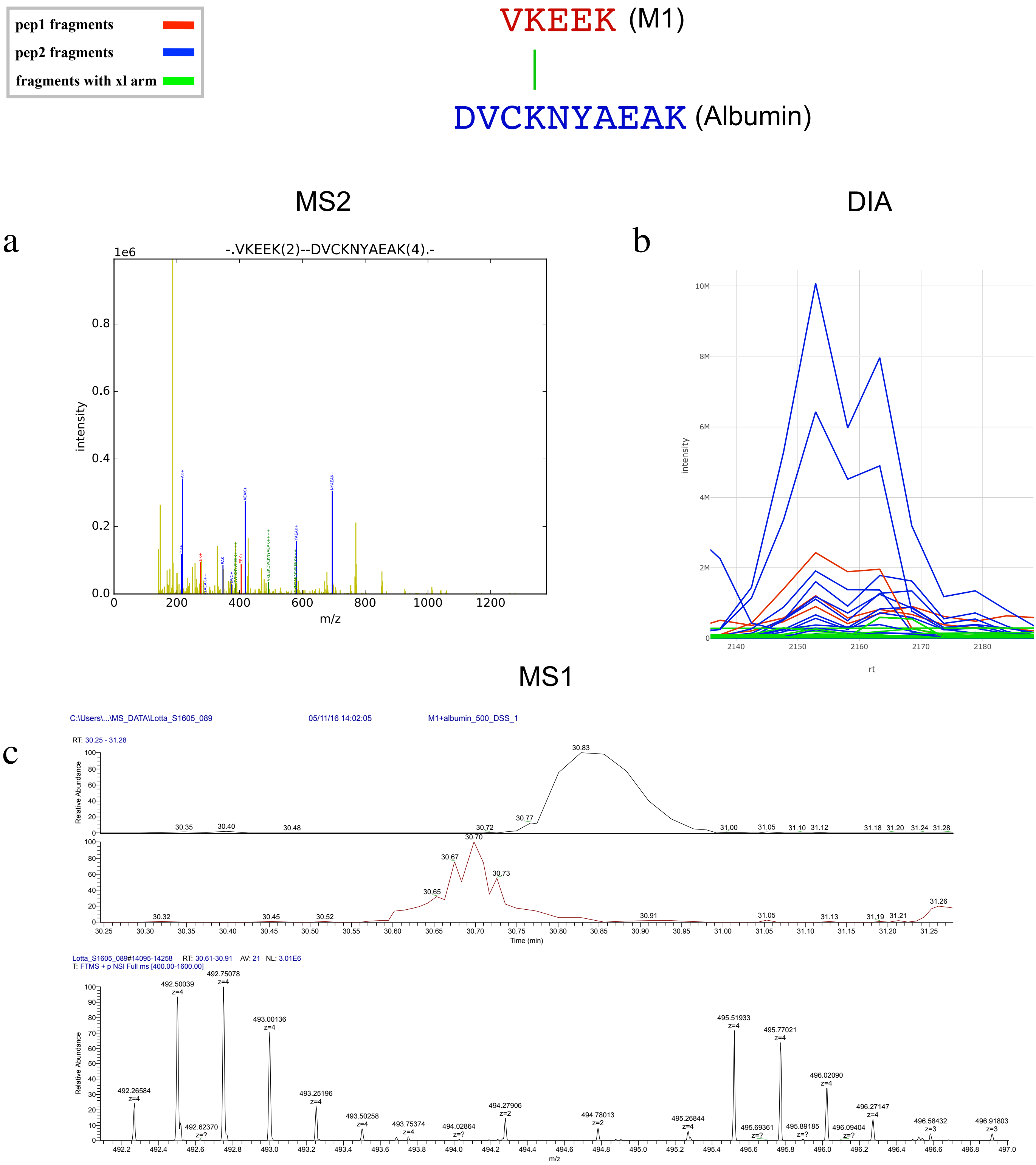

pep1 fragments
pep2 fragments
fragments with xl arm
VKEEK (M1)
DVCKNYAEAK (Albumin)
MS2
DIA
a
1e6
-.VKEEK(2)--DVCKNYAEAK(4).-
intensity
m/z
b
intensity
rt
MS1
c
C:\Users\...\MS_DATA\Lotta_S1605_089
05/11/16 14:02:05
M1+albumin_500_DSS_1
RT: 30.25 - 31.28
Relative Abundance
Time (min)
Lotta_S1605_089#14095-14258 RT: 30.61-30.91 AV: 21 NL: 3.01E6
T: FTMS + p NSI Full ms [400.00-1600.00]
Relative Abundance
m/z

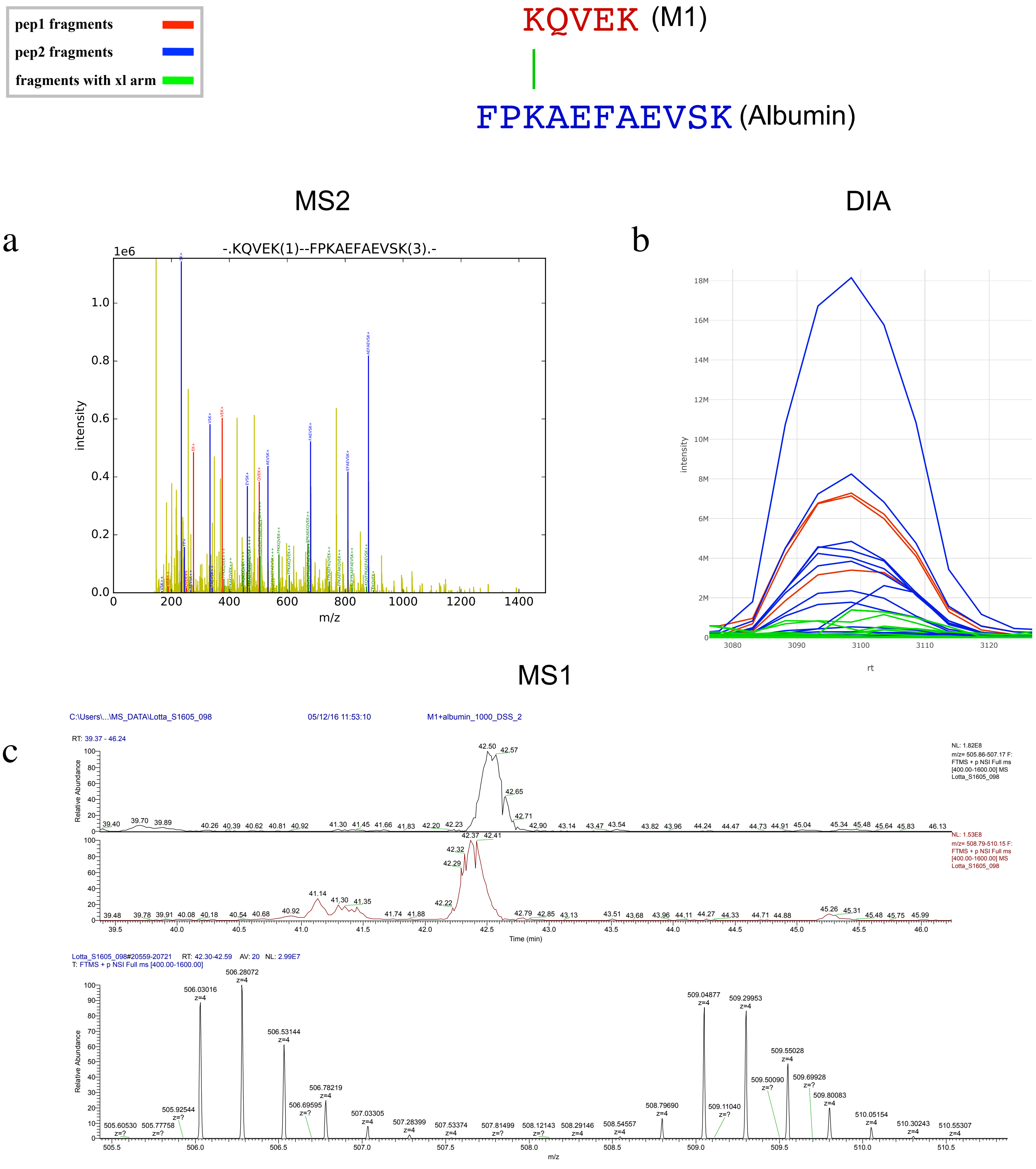

pep1 fragments
pep2 fragments
fragments with xl arm
KQVEK (M1)
FPKAEFAEVSK (Albumin)
MS2
DIA
a
-.KQVEK(1)--FPKAEFAEVSK(3).-
intensity
m/z
b
intensity
rt
MS1
c
C:\Users\...\MS_DATA\Lotta_S1605_098
05/12/16 11:53:10
M1+albumin_1000_DSS_2
RT: 39.37 - 46.24
NL: 1.82E8
m/z= 505.86-507.17 F: FTMS + p NSI Full ms [400.00-1600.00] MS Lotta_S1605_098
NL: 1.53E8
m/z= 508.79-510.15 F: FTMS + p NSI Full ms [400.00-1600.00] MS Lotta_S1605_098
Relative Abundance
Time (min)
Lotta_S1605_098#20559-20721 RT: 42.30-42.59 AV: 20 NL: 2.99E7
T: FTMS + p NSI Full ms [400.00-1600.00]
Relative Abundance
m/z

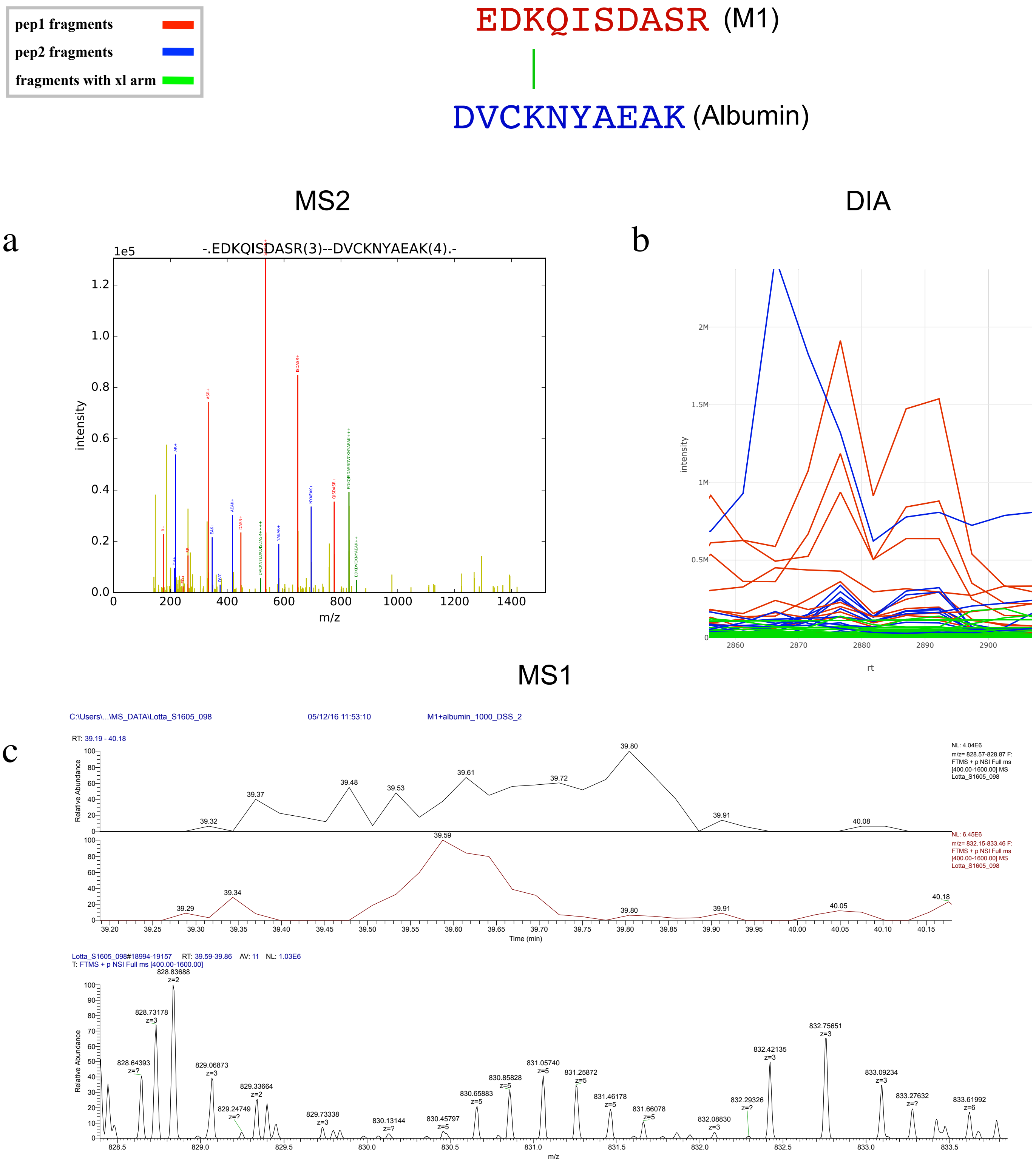
pep1 fragments
pep2 fragments
fragments with xl arm
EDKQISDASR (M1)
DVCKNYAEAK (Albumin)
MS2
DIA
a
b
-.EDKQISDASR(3)--DVCKNYAEAK(4).-
1e5
intensity
m/z
rt
MS1
c
C:\Users\...\MS_DATA\Lotta_S1605_098
05/12/16 11:53:10
M1+albumin_1000_DSS_2
RT: 39.19 - 40.18
NL: 4.04E6
m/z= 828.57-828.87 F: FTMS + p NSI Full ms [400.00-1600.00] MS Lotta_S1605_098
NL: 6.45E6
m/z= 832.15-833.46 F: FTMS + p NSI Full ms [400.00-1600.00] MS Lotta_S1605_098
Relative Abundance
Time (min)
Lotta_S1605_098#18994-19157 RT: 39.59-39.86 AV: 11 NL: 1.03E6
T: FTMS + p NSI Full ms [400.00-1600.00]
828.83688 z=2
828.73178 z=3
828.64393 z=?
829.06873 z=3
829.33664 z=2
829.24749 z=?
829.73338 z=3
830.13144 z=?
830.45797 z=5
830.65883 z=5
830.85828 z=5
831.05740 z=5
831.25872 z=5
831.46178 z=5
831.66078 z=5
832.08830 z=3
832.29326 z=?
832.42135 z=3
832.75651 z=3
833.09234 z=3
833.27632 z=?
833.61992 z=6
m/z

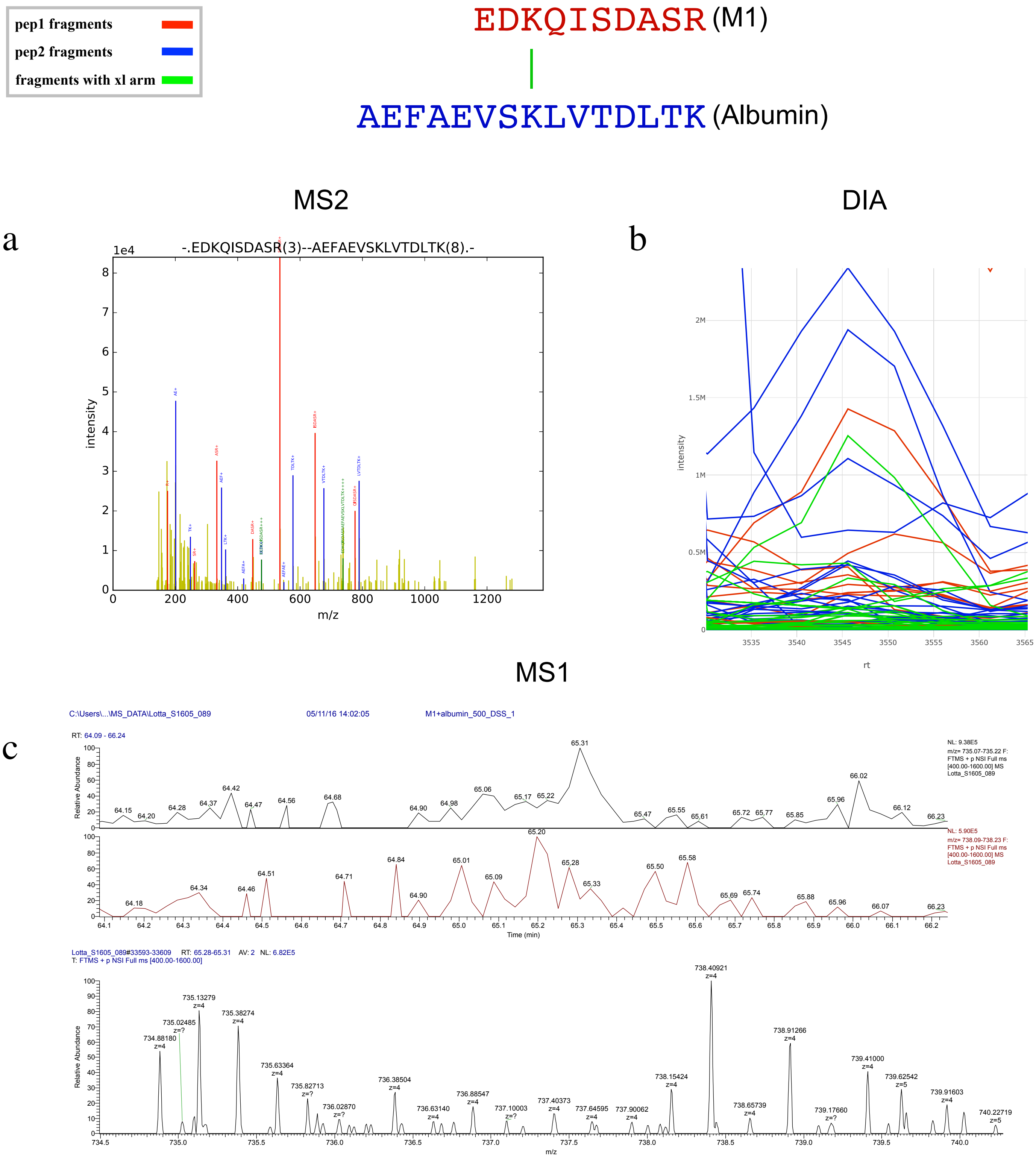
pep1 fragments
pep2 fragments
fragments with xl arm
EDKQISDASR (M1)
AEFAEVSKLVTDLTK (Albumin)
MS2
DIA
a
-.EDKQISDASR(3)--AEFAEVSKLVTDLTK(8).-
1e4
intensity
m/z
b
intensity
rt
MS1
c
C:\Users\...\MS_DATA\Lotta_S1605_089
05/11/16 14:02:05
M1+albumin_500_DSS_1
RT: 64.09 - 66.24
NL: 9.38E5
m/z= 735.07-735.22 F: FTMS + p NSI Full ms [400.00-1600.00] MS Lotta_S1605_089
NL: 5.90E5
m/z= 738.09-738.23 F: FTMS + p NSI Full ms [400.00-1600.00] MS Lotta_S1605_089
Relative Abundance
Time (min)
Lotta_S1605_089#33593-33609 RT: 65.28-65.31 AV: 2 NL: 6.82E5
T: FTMS + p NSI Full ms [400.00-1600.00]
Relative Abundance
m/z

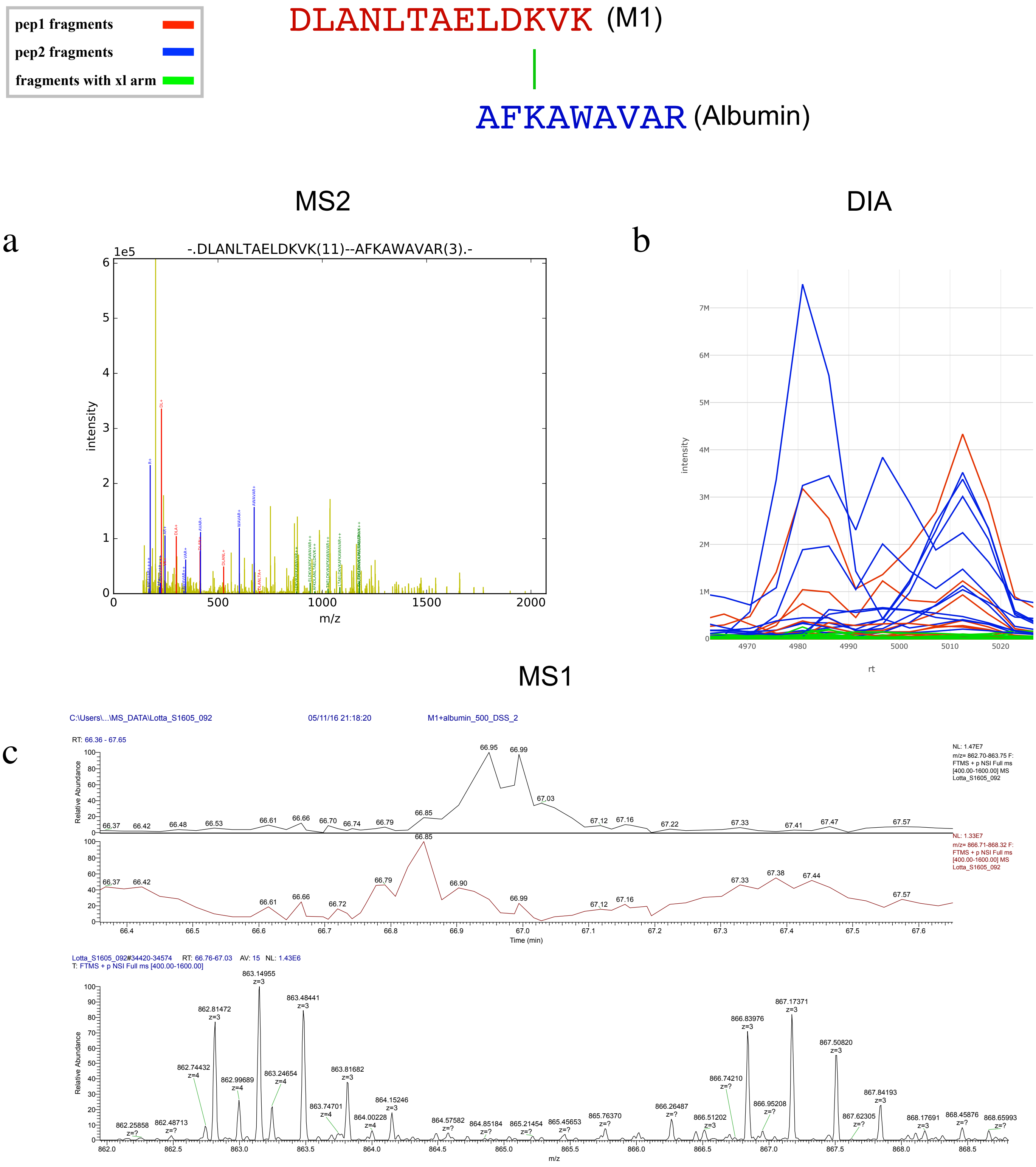

pep1 fragments
pep2 fragments
fragments with xl arm
DLANLTAELDKVK (M1)
AFKAWAVAR (Albumin)
MS2
DIA
a
-.DLANLTAELDKVK(11)--AFKAWAVAR(3).-
1e5
intensity
m/z
b
intensity
rt
MS1
c
C:\Users\...\MS_DATA\Lotta_S1605_092
05/11/16 21:18:20
M1+albumin_500_DSS_2
RT: 66.36 - 67.65
NL: 1.47E7
m/z= 862.70-863.75 F: FTMS + p NSI Full ms [400.00-1600.00] MS Lotta_S1605_092
NL: 1.33E7
m/z= 866.71-868.32 F: FTMS + p NSI Full ms [400.00-1600.00] MS Lotta_S1605_092
Relative Abundance
Time (min)
Lotta_S1605_092#34420-34574 RT: 66.76-67.03 AV: 15 NL: 1.43E6
T: FTMS + p NSI Full ms [400.00-1600.00]
Relative Abundance
m/z

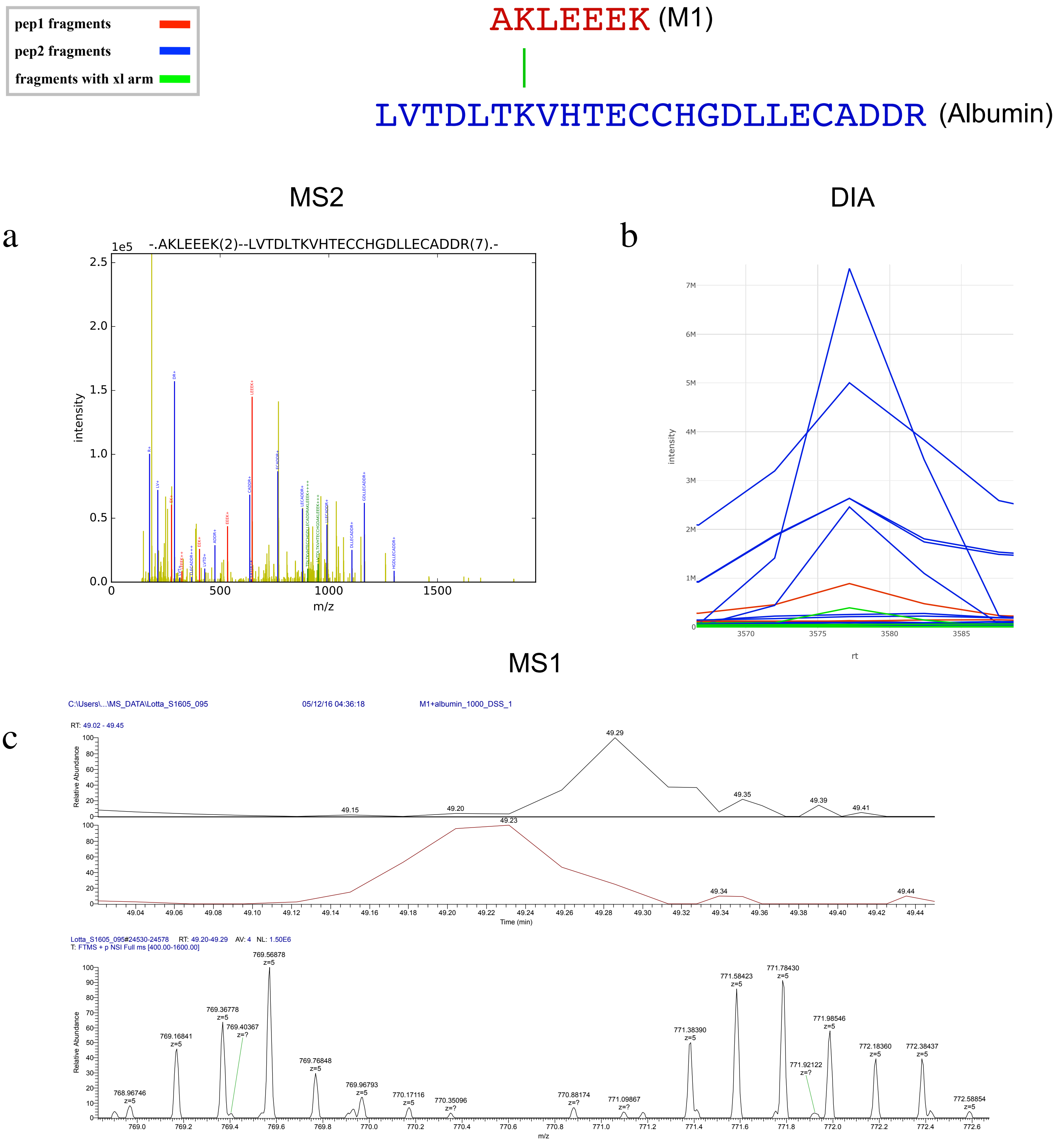

pep1 fragments
pep2 fragments
fragments with xl arm
AKLEEEK (M1)
LVTDLTKVHTECCHGDLLECADDR (Albumin)
MS2
DIA
a
-.AKLEEEK(2)--LVTDLTKVHTECCHGDLLECADDR(7).-
1e5
intensity
m/z
b
intensity
rt
MS1
c
C:\Users\...\MS_DATA\Lotta_S1605_095
05/12/16 04:36:18
M1+albumin_1000_DSS_1
RT: 49.02 - 49.45
Relative Abundance
Time (min)
Lotta_S1605_095#24530-24578 RT: 49.20-49.29 AV: 4 NL: 1.50E6
T: FTMS + p NSI Full ms [400.00-1600.00]
Relative Abundance
m/z

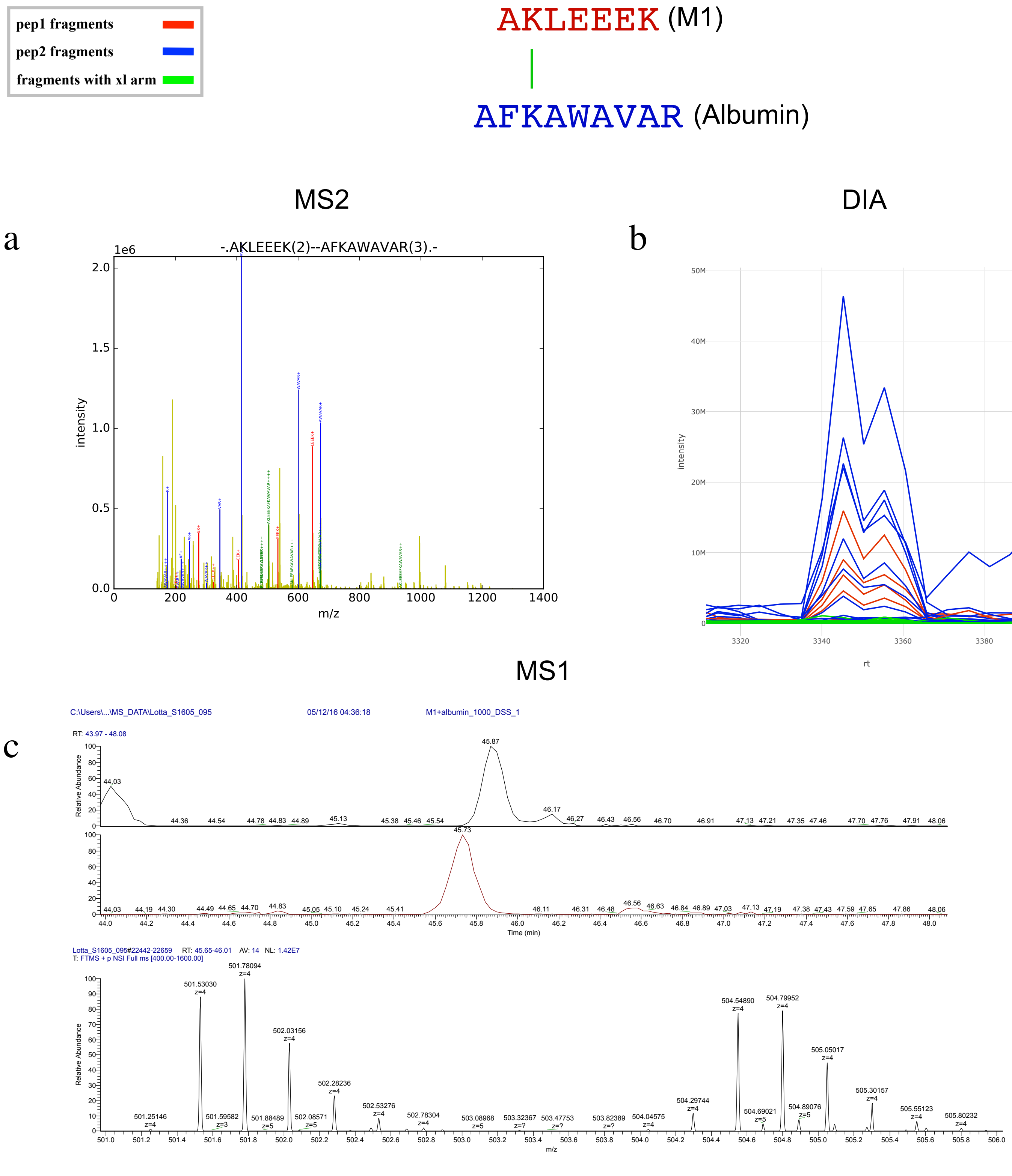

pep1 fragments
pep2 fragments
fragments with xl arm
AKLEEEK (M1)
AFKAWAVAR (Albumin)
MS2
DIA
a
-.AKLEEEK(2)--AFKAWAVAR(3).-
intensity
m/z
b
intensity
rt
MS1
c
C:\Users\...\MS_DATA\Lotta_S1605_095
05/12/16 04:36:18
M1+albumin_1000_DSS_1
RT: 43.97 - 48.08
Relative Abundance
Time (min)
Lotta_S1605_095#22442-22659 RT: 45.65-46.01 AV: 14 NL: 1.42E7
T: FTMS + p NSI Full ms [400.00-1600.00]
Relative Abundance
m/z

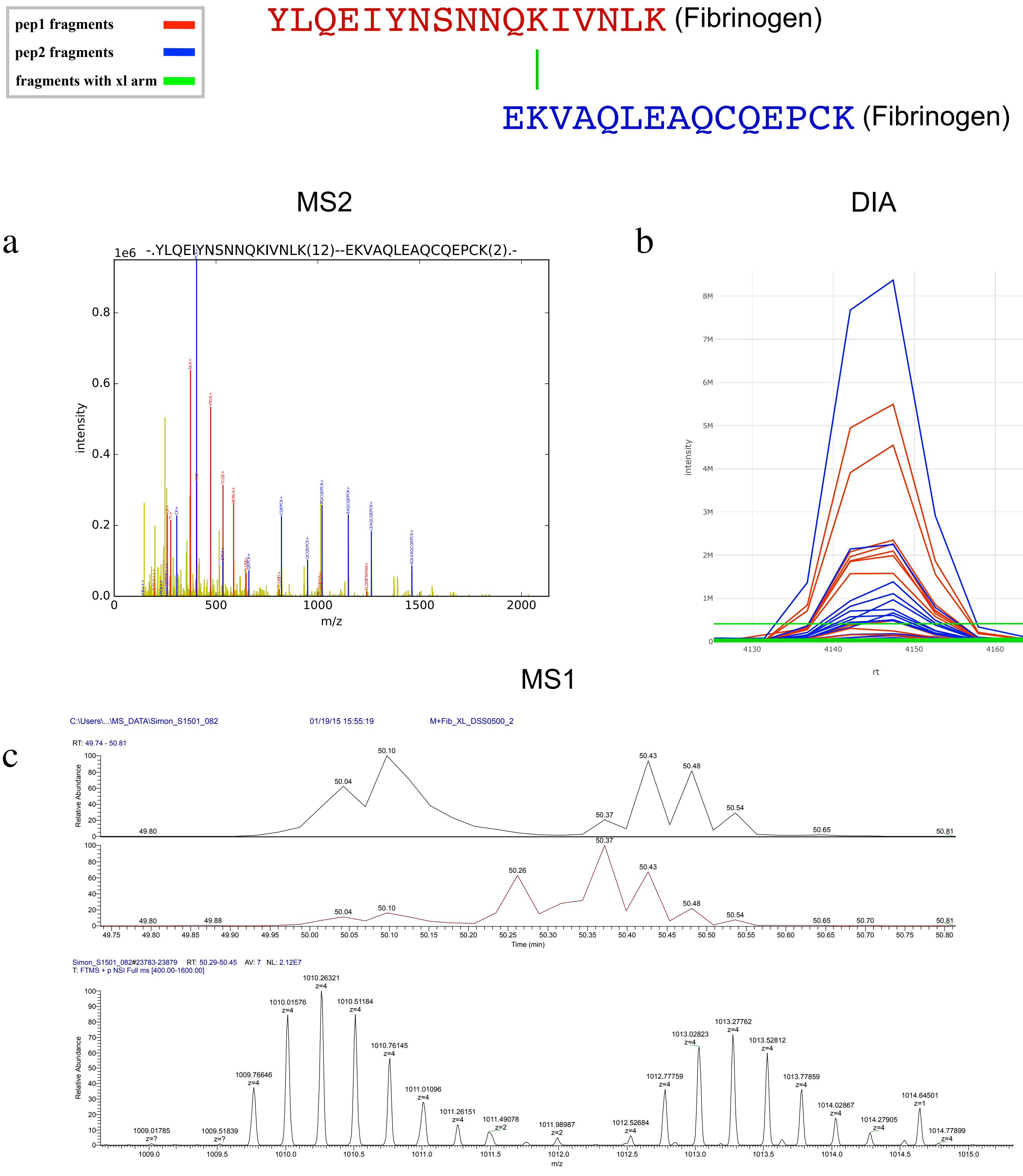

pep1 fragments
pep2 fragments
fragments with xl arm
YLQEIYNSNNQKIVNLK (Fibrinogen)
EKVAQLEAQCQEPCK (Fibrinogen)
MS2
DIA
a
b
-.YLQEIYNSNNQKIVNLK(12)--EKVAQLEAQCQEPCK(2).-
1e6
intensity
m/z
0.0
0.2
0.4
0.6
0.8
0
500
1000
1500
2000
intensity
rt
0
1M
2M
3M
4M
5M
6M
7M
8M
4130
4140
4150
4160
MS1
c
C:\Users\...\MS_DATA\Simon_S1501_082
01/19/15 15:55:19
M+Fib_XL_DSS0500_2
RT: 49.74 - 50.81
Relative Abundance
Time (min)
Simon_S1501_082#23783-23879 RT: 50.29-50.45 AV: 7 NL: 2.12E7
T: FTMS + p NSI Full ms [400.00-1600.00]
Relative Abundance
m/z
1009.01785 z=?
1009.51839 z=?
1009.76646 z=4
1010.01576 z=4
1010.26321 z=4
1010.51184 z=4
1010.76145 z=4
1011.01096 z=4
1011.26151 z=4
1011.49078 z=2
1011.98987 z=2
1012.52684 z=4
1012.77759 z=4
1013.02823 z=4
1013.27762 z=4
1013.52812 z=4
1013.77859 z=4
1014.02867 z=4
1014.27905 z=4
1014.64501 z=1
1014.77899 z=4

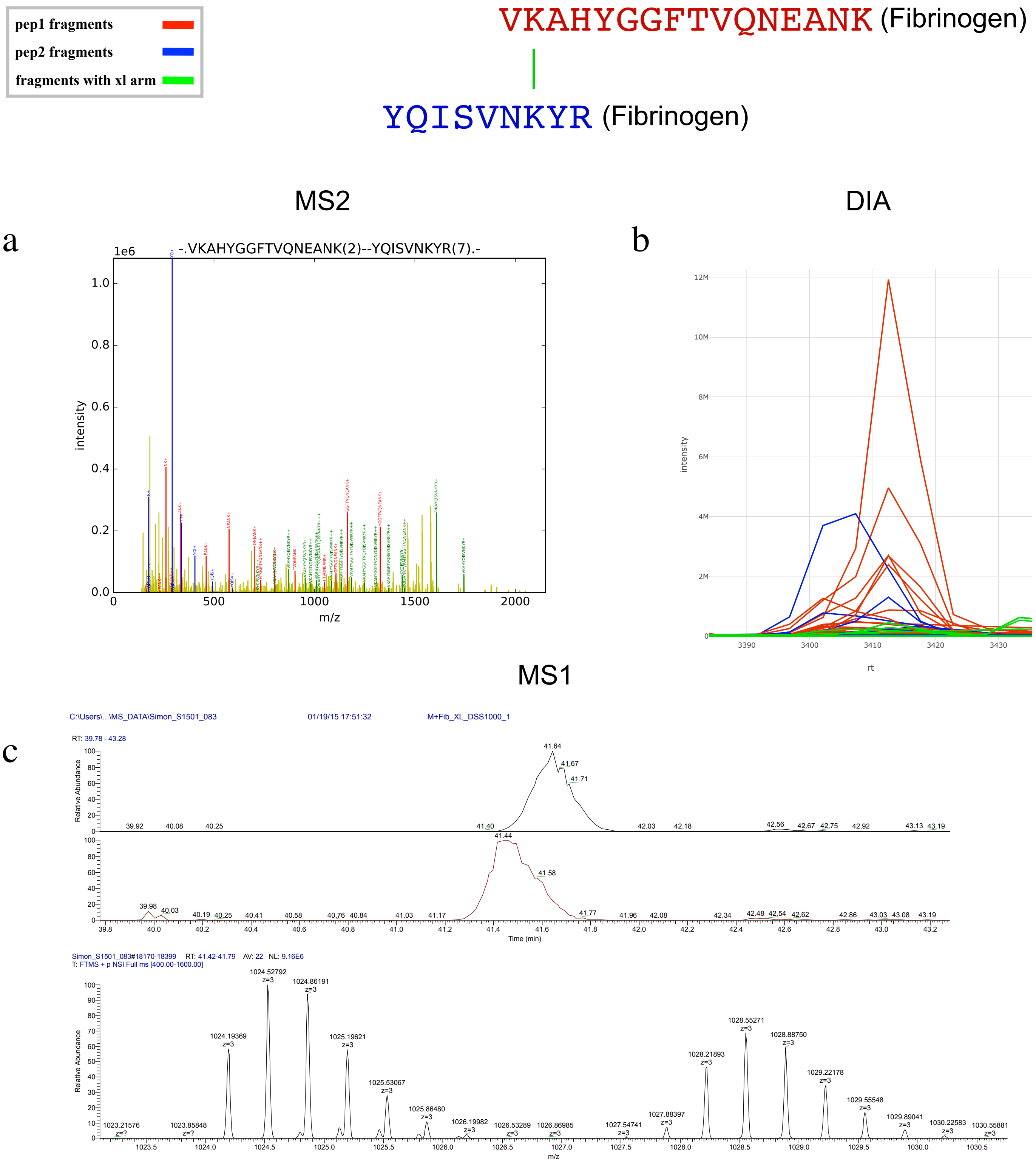
pep1 fragments
pep2 fragments
fragments with xl arm
VKAHYGGFTVQNEANK (Fibrinogen)
YQISVNKYR (Fibrinogen)
MS2
DIA
a
-.VKAHYGGFTVQNEANK(2)--YQISVNKYR(7).-
1e6
intensity
m/z
b
intensity
rt
MS1
c
C:\Users\...\MS_DATA\Simon_S1501_083
01/19/15 17:51:32
M+Fib_XL_DSS1000_1
RT: 39.78 - 43.28
Relative Abundance
Time (min)
Simon_S1501_083#18170-18399 RT: 41.42-41.79 AV: 22 NL: 9.16E6
T: FTMS + p NSI Full ms [400.00-1600.00]
Relative Abundance
m/z

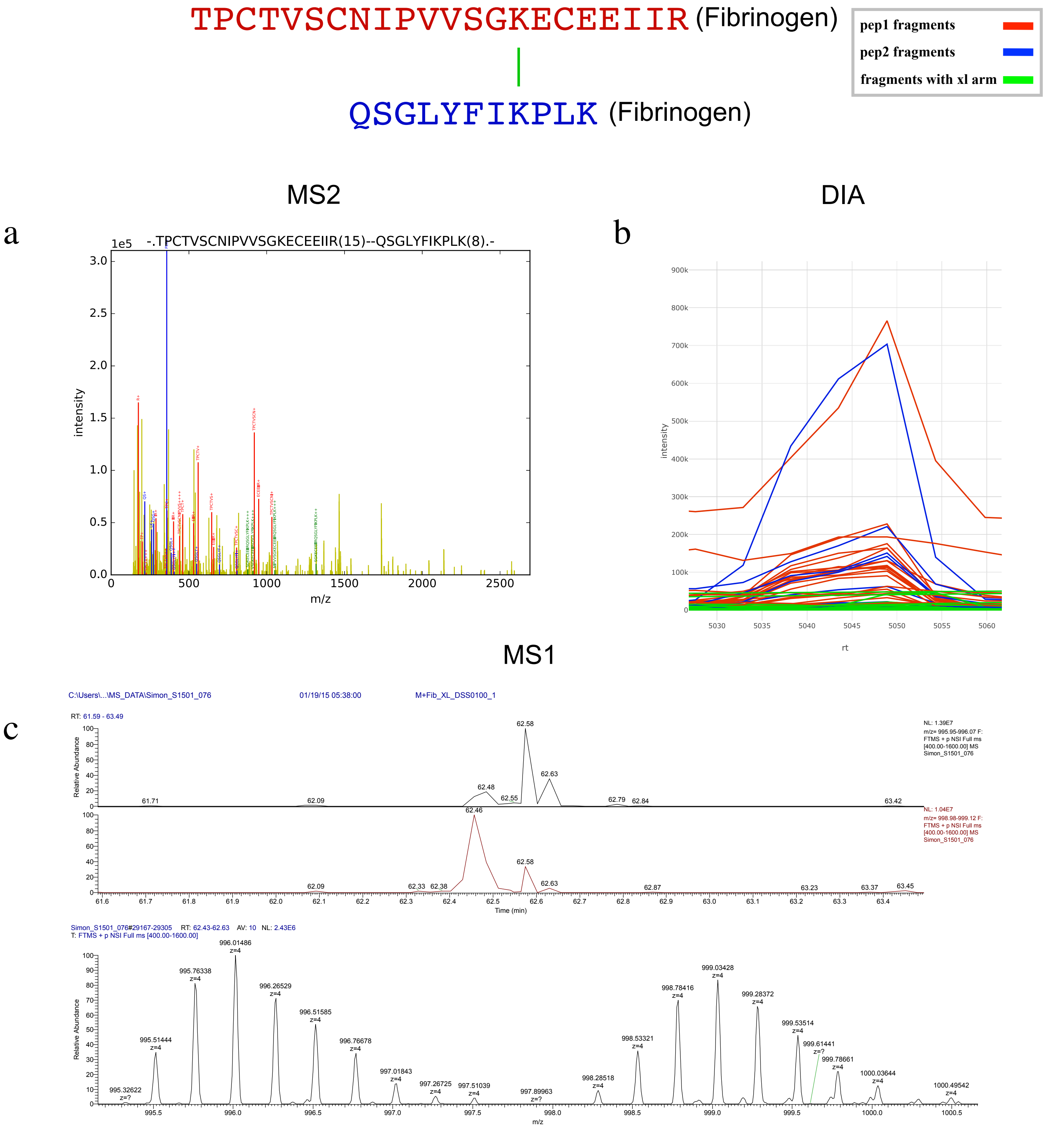

TPCTVSCNIPVVSGKECEEIIR (Fibrinogen)
QSGLYFIKPLK (Fibrinogen)
pep1 fragments
pep2 fragments
fragments with xl arm
MS2
DIA
a
-.TPCTVSCNIPVVSGKECEEIIR(15)--QSGLYFIKPLK(8).-
1e5
intensity
m/z
b
intensity
rt
MS1
c
C:\Users\...\MS_DATA\Simon_S1501_076
01/19/15 05:38:00
M+Fib_XL_DSS0100_1
RT: 61.59 - 63.49
NL: 1.39E7
m/z= 995.95-996.07 F: FTMS + p NSI Full ms [400.00-1600.00] MS Simon_S1501_076
NL: 1.04E7
m/z= 998.98-999.12 F: FTMS + p NSI Full ms [400.00-1600.00] MS Simon_S1501_076
Relative Abundance
Time (min)
Simon_S1501_076#29167-29305 RT: 62.43-62.63 AV: 10 NL: 2.43E6
T: FTMS + p NSI Full ms [400.00-1600.00]
Relative Abundance
m/z

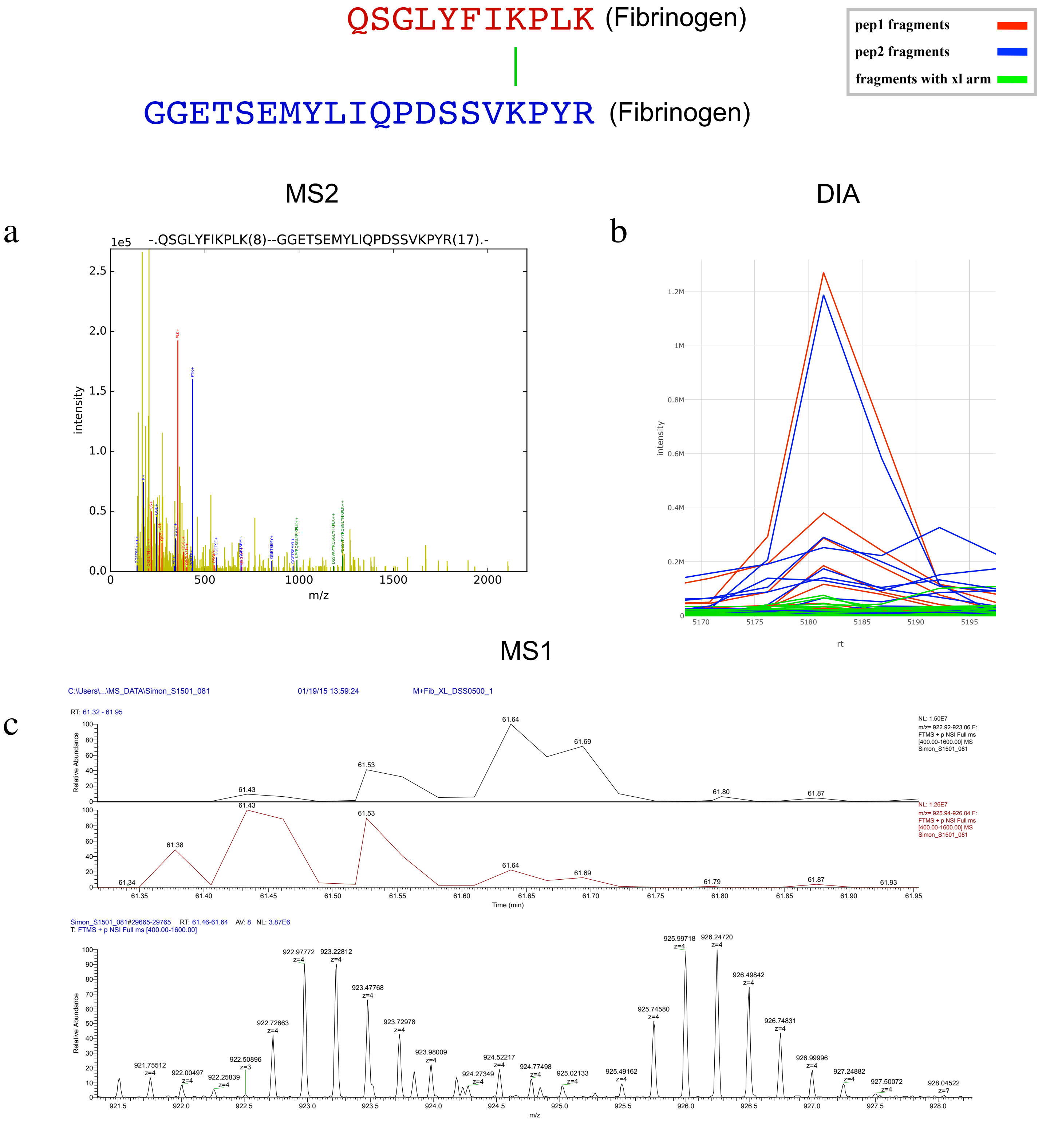
QSGLYFIKPLK (Fibrinogen)
GGETSEMYLIQPDSSVKPYR (Fibrinogen)
pep1 fragments
pep2 fragments
fragments with xl arm
MS2
DIA
a
-.QSGLYFIKPLK(8)--GGETSEMYLIQPDSSVKPYR(17).-
1e5
intensity
m/z
b
intensity
rt
MS1
c
C:\Users\...\MS_DATA\Simon_S1501_081
01/19/15 13:59:24
M+Fib_XL_DSS0500_1
RT: 61.32 - 61.95
Relative Abundance
NL: 1.50E7
m/z= 922.92-923.06 F: FTMS + p NSI Full ms [400.00-1600.00] MS Simon_S1501_081
NL: 1.26E7
m/z= 925.94-926.04 F: FTMS + p NSI Full ms [400.00-1600.00] MS Simon_S1501_081
Time (min)
Simon_S1501_081#29665-29765 RT: 61.46-61.64 AV: 8 NL: 3.87E6
T: FTMS + p NSI Full ms [400.00-1600.00]
m/z

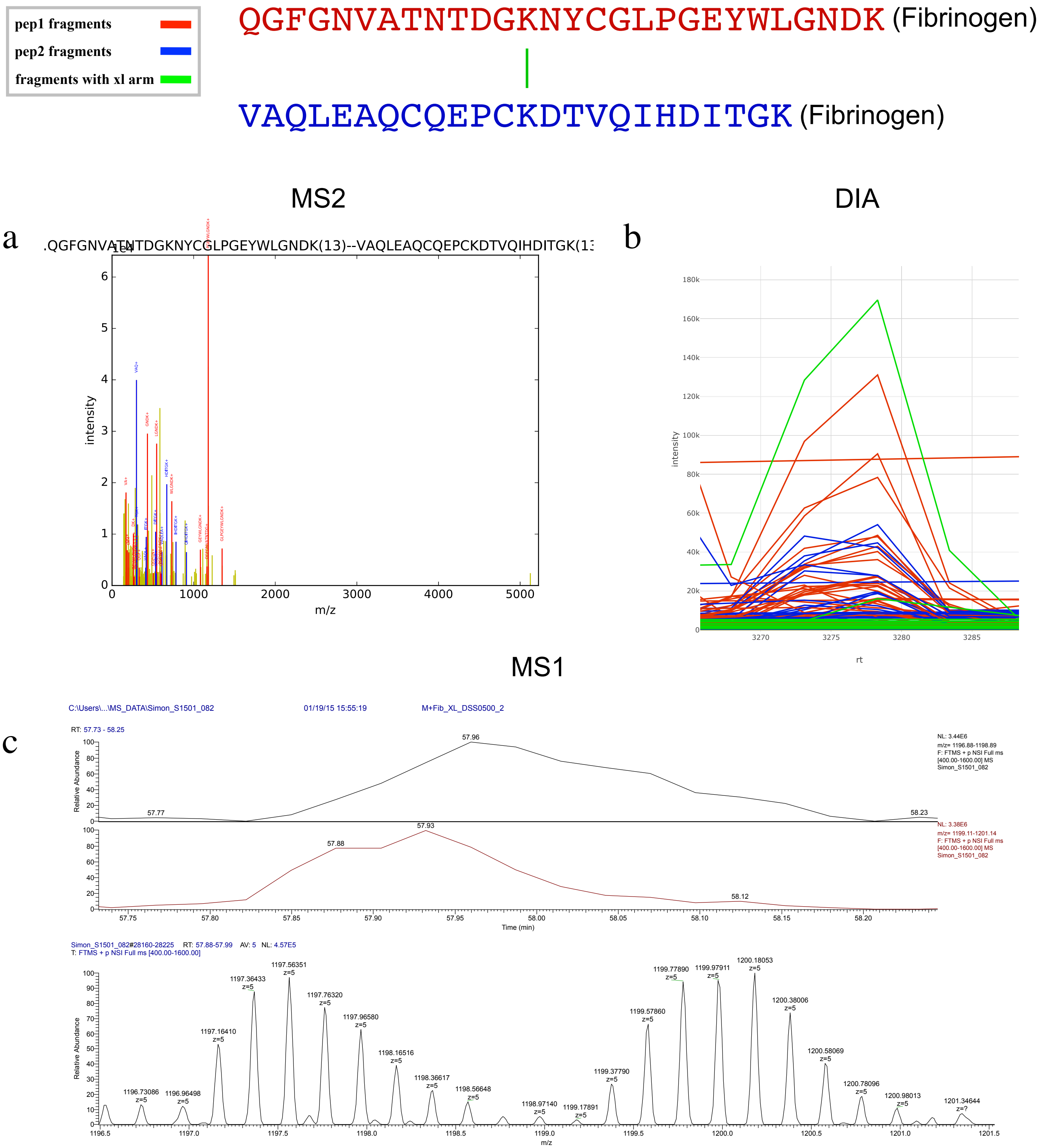

pep1 fragments
pep2 fragments
fragments with xl arm
QGFGNVATNTDGKNYCGLPGEYWLGNDK (Fibrinogen)
VAQLEAQCQEPCKDTVQIHDITGK (Fibrinogen)
MS2
DIA
a
.QGFGNVATNTDGKNYCGLPGEYWLGNDK(13)--VAQLEAQCQEPCKDTVQIHDITGK(13
1e4
intensity
m/z
b
intensity
rt
MS1
c
C:\Users\...\MS_DATA\Simon_S1501_082
01/19/15 15:55:19
M+Fib_XL_DSS0500_2
RT: 57.73 - 58.25
NL: 3.44E6
m/z= 1196.88-1198.89
F: FTMS + p NSI Full ms [400.00-1600.00] MS
Simon_S1501_082
NL: 3.38E6
m/z= 1199.11-1201.14
F: FTMS + p NSI Full ms [400.00-1600.00] MS
Simon_S1501_082
Relative Abundance
Time (min)
Simon_S1501_082#28160-28225 RT: 57.88-57.99 AV: 5 NL: 4.57E5
T: FTMS + p NSI Full ms [400.00-1600.00]
Relative Abundance
m/z

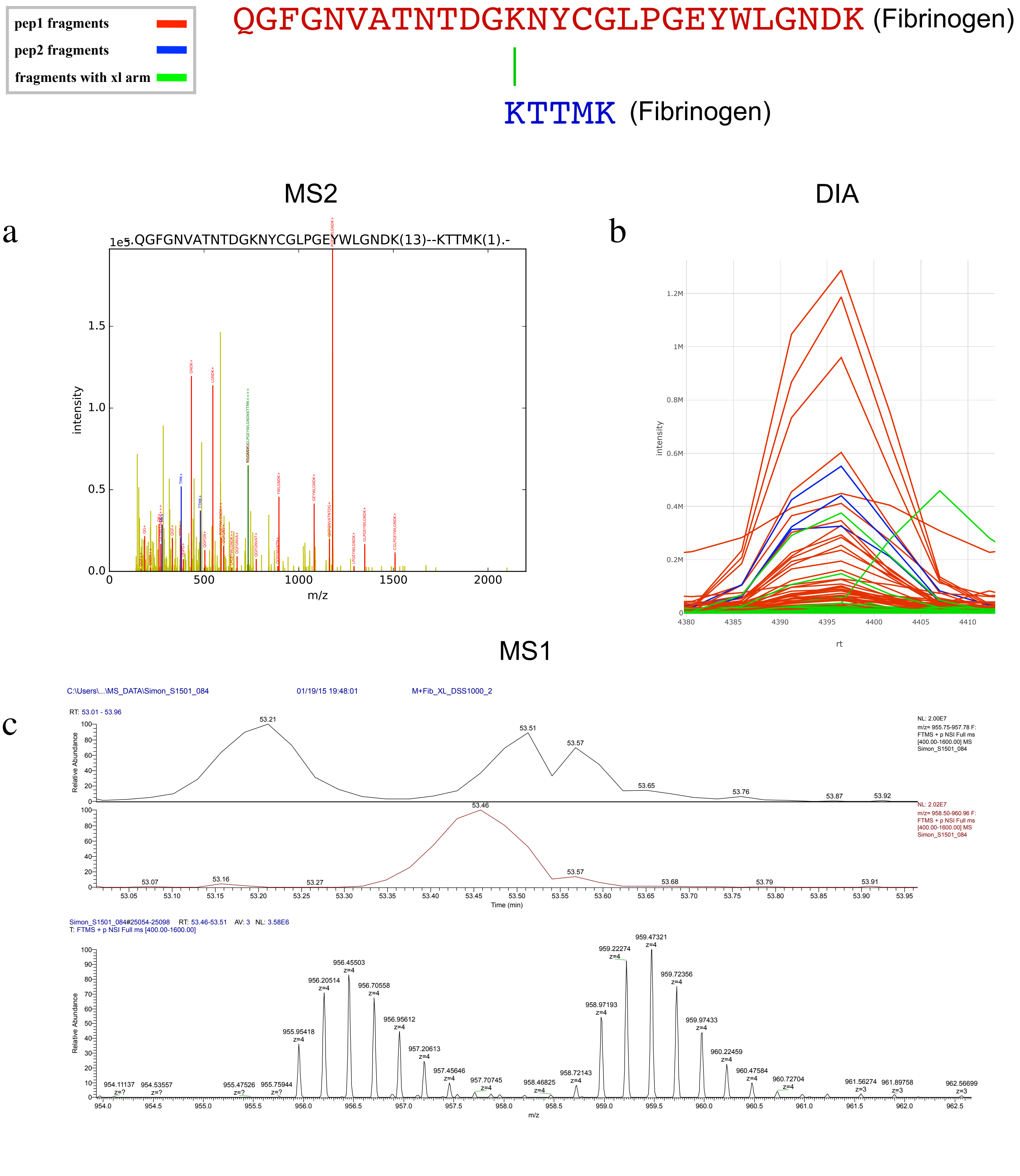

pep1 fragments
pep2 fragments
fragments with xl arm
QGFGNVATNTDGKNYCGLPGEYWLGNDK (Fibrinogen)
KTTMK (Fibrinogen)
MS2
DIA
a
1e5.QGFGNVATNTDGKNYCGLPGEYWLGNDK(13)--KTTMK(1).-
intensity
m/z
b
intensity
rt
MS1
c
C:\Users\...\MS_DATA\Simon_S1501_084
01/19/15 19:48:01
M+Fib_XL_DSS1000_2
RT: 53.01 - 53.96
Relative Abundance
NL: 2.00E7
m/z= 955.75-957.78 F: FTMS + p NSI Full ms [400.00-1600.00] MS Simon_S1501_084
NL: 2.02E7
m/z= 958.50-960.96 F: FTMS + p NSI Full ms [400.00-1600.00] MS Simon_S1501_084
Time (min)
Simon_S1501_084#25054-25098 RT: 53.46-53.51 AV: 3 NL: 3.58E6
T: FTMS + p NSI Full ms [400.00-1600.00]
Relative Abundance
m/z

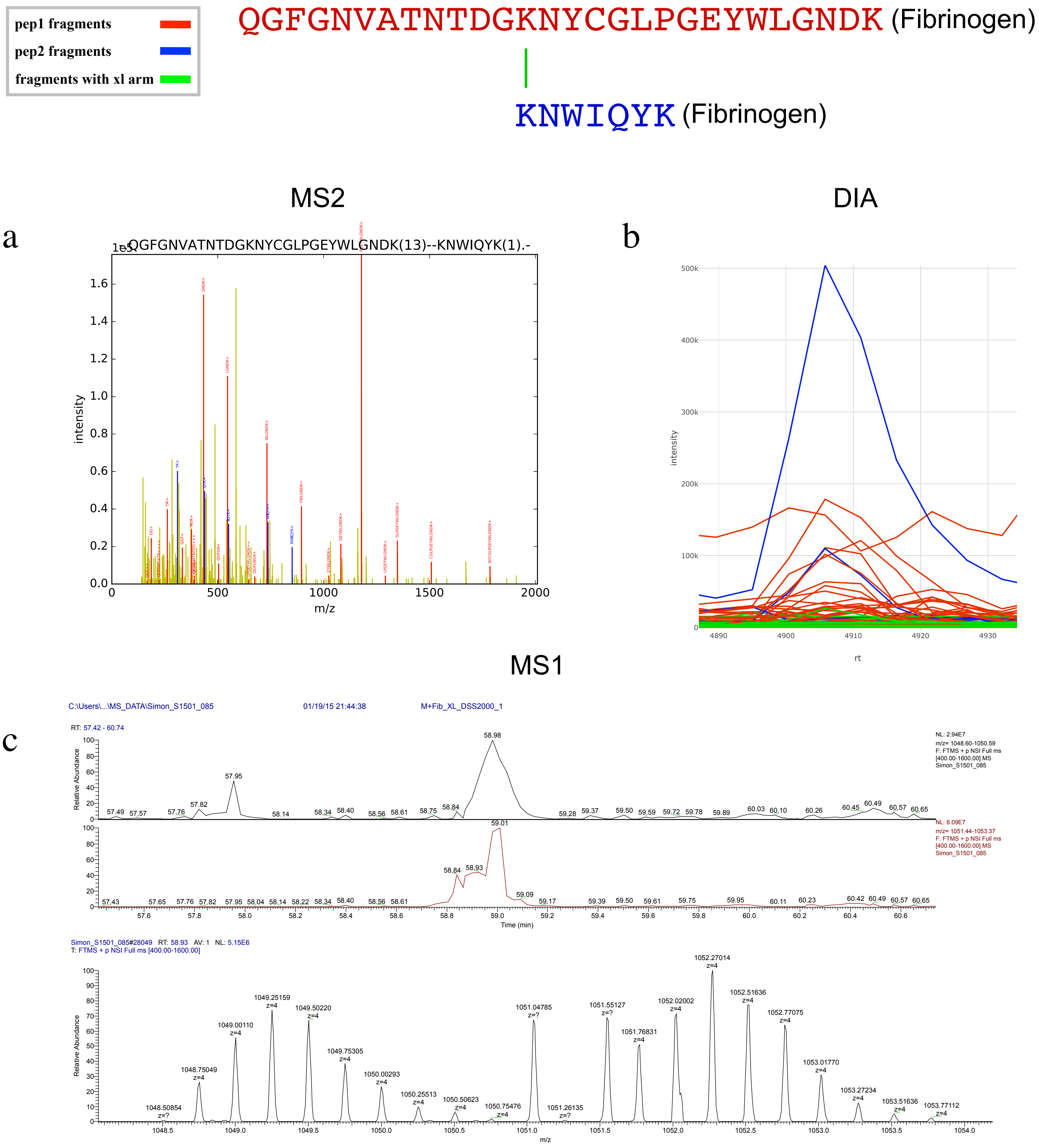
pep1 fragments
pep2 fragments
fragments with xl arm
QGFGNVATNTDGKNYCGLPGEYWLGNDK (Fibrinogen)
KNWIQYK (Fibrinogen)
MS2
DIA
a
QGFGNVATNTDGKNYCGLPGEYWLGNDK(13)--KNWIQYK(1).-
1e5
intensity
m/z
b
intensity
rt
MS1
c
C:\Users\...\MS_DATA\Simon_S1501_085
01/19/15 21:44:38
M+Fib_XL_DSS2000_1
RT: 57.42 - 60.74
NL: 2.94E7
m/z= 1048.60-1050.59
F: FTMS + p NSI Full ms [400.00-1600.00] MS
Simon_S1501_085
NL: 6.09E7
m/z= 1051.44-1053.37
F: FTMS + p NSI Full ms [400.00-1600.00] MS
Simon_S1501_085
Relative Abundance
Time (min)
Simon_S1501_085#28049 RT: 58.93 AV: 1 NL: 5.15E6
T: FTMS + p NSI Full ms [400.00-1600.00]
Relative Abundance
m/z

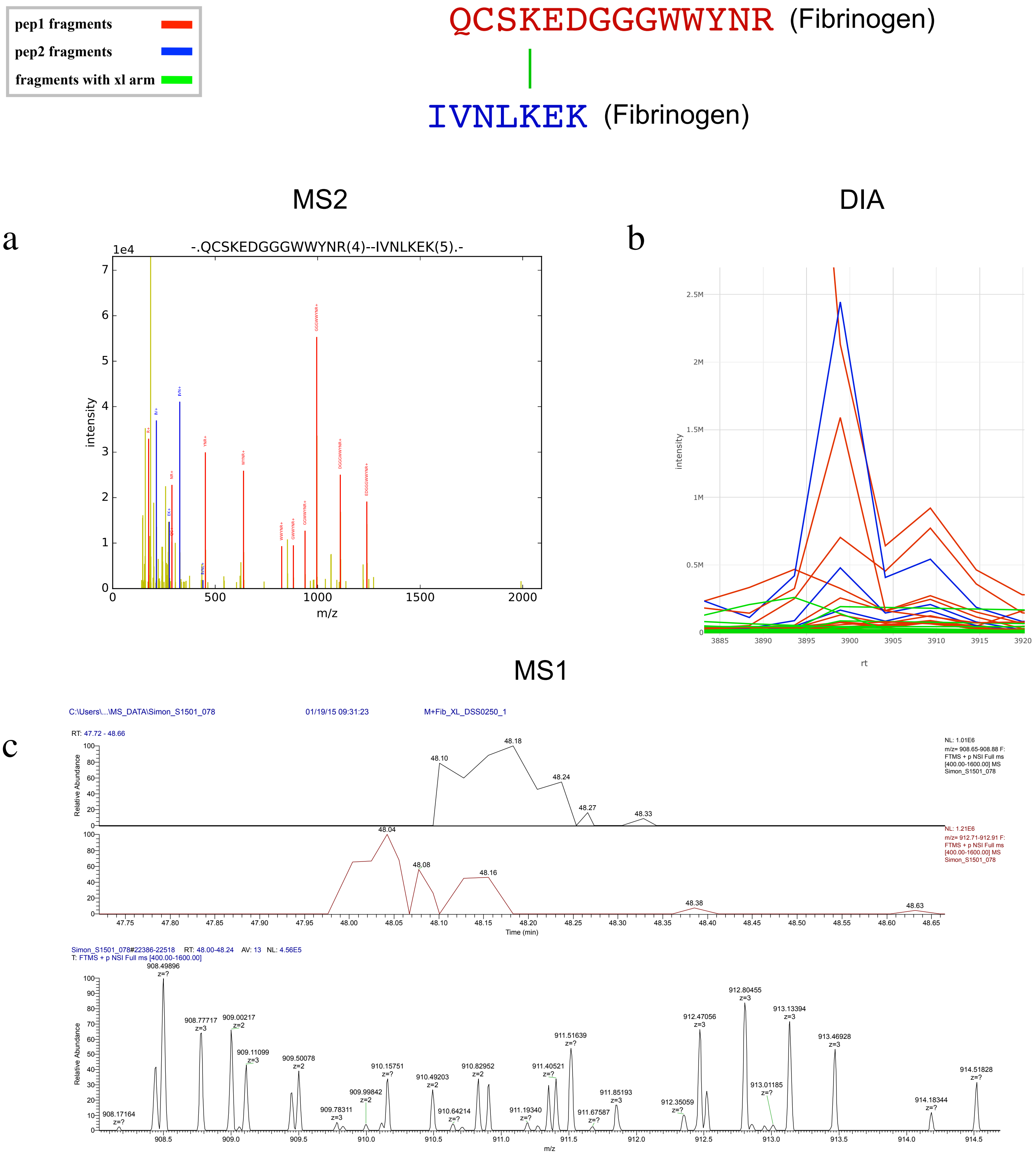
pep1 fragments
pep2 fragments
fragments with xl arm
QCSKEDGGGWWYNR (Fibrinogen)
IVNLKEK (Fibrinogen)
MS2
DIA
a
-.QCSKEDGGGWWYNR(4)--IVNLKEK(5).-
intensity
m/z
b
intensity
rt
MS1
c
C:\Users\...\MS_DATA\Simon_S1501_078
01/19/15 09:31:23
M+Fib_XL_DSS0250_1
RT: 47.72 - 48.66
NL: 1.01E6
m/z= 908.65-908.88 F: FTMS + p NSI Full ms [400.00-1600.00] MS Simon_S1501_078
NL: 1.21E6
m/z= 912.71-912.91 F: FTMS + p NSI Full ms [400.00-1600.00] MS Simon_S1501_078
Relative Abundance
Time (min)
Simon_S1501_078#22386-22518 RT: 48.00-48.24 AV: 13 NL: 4.56E5
T: FTMS + p NSI Full ms [400.00-1600.00]
Relative Abundance
m/z

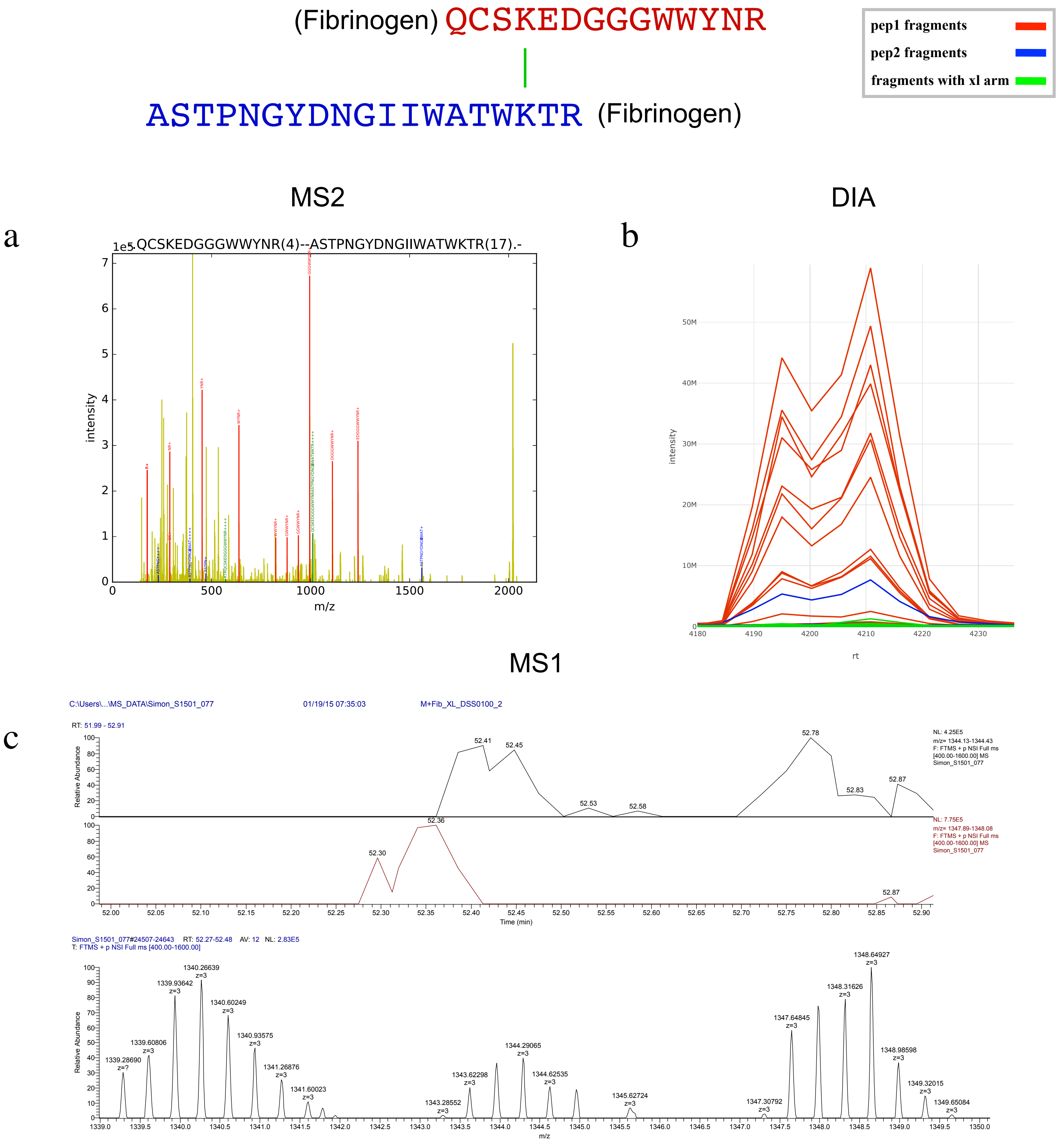

(Fibrinogen) QCSKEDGGGWWYNR
ASTPNGYDNGIIWATWKTR (Fibrinogen)
pep1 fragments
pep2 fragments
fragments with xl arm
MS2
DIA
a
b
1e5.QCSKEDGGGWWYNR(4)--ASTPNGYDNGIIWATWKTR(17).-
intensity
m/z
rt
MS1
c
C:\Users\...\MS_DATA\Simon_S1501_077
01/19/15 07:35:03
M+Fib_XL_DSS0100_2
RT: 51.99 - 52.91
NL: 4.25E5
m/z= 1344.13-1344.43
F: FTMS + p NSI Full ms [400.00-1600.00] MS
Simon_S1501_077
NL: 7.75E5
m/z= 1347.89-1348.08
F: FTMS + p NSI Full ms [400.00-1600.00] MS
Simon_S1501_077
Relative Abundance
Time (min)
Simon_S1501_077#24507-24643 RT: 52.27-52.48 AV: 12 NL: 2.83E5
T: FTMS + p NSI Full ms [400.00-1600.00]
Relative Abundance
m/z

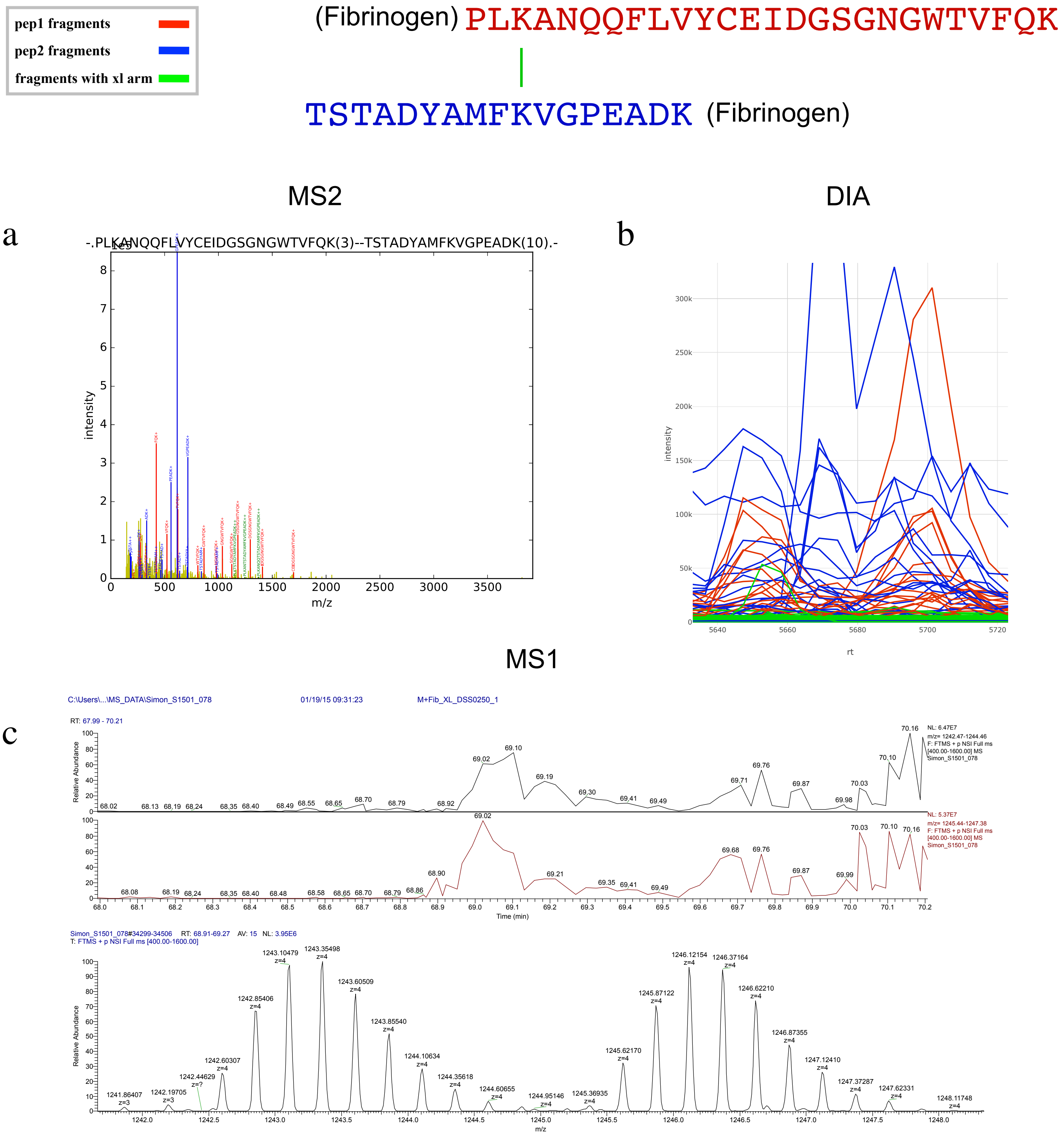

pep1 fragments
pep2 fragments
fragments with xl arm
(Fibrinogen) PLKANQQFLVYCEIDGSGNGWTVFQK
TSTADYAMFKVGPEADK (Fibrinogen)
MS2
DIA
a
-.PLKANQQFLVYCEIDGSGNGWTVFQK(3)--TSTADYAMFKVGPEADK(10).-
intensity
m/z
b
intensity
rt
MS1
c
C:\Users\...\MS_DATA\Simon_S1501_078
01/19/15 09:31:23
M+Fib_XL_DSS0250_1
RT: 67.99 - 70.21
NL: 6.47E7
m/z= 1242.47-1244.46
F: FTMS + p NSI Full ms [400.00-1600.00] MS Simon_S1501_078
NL: 5.37E7
m/z= 1245.44-1247.38
F: FTMS + p NSI Full ms [400.00-1600.00] MS Simon_S1501_078
Relative Abundance
Time (min)
Simon_S1501_078#34299-34506 RT: 68.91-69.27 AV: 15 NL: 3.95E6
T: FTMS + p NSI Full ms [400.00-1600.00]
Relative Abundance
m/z

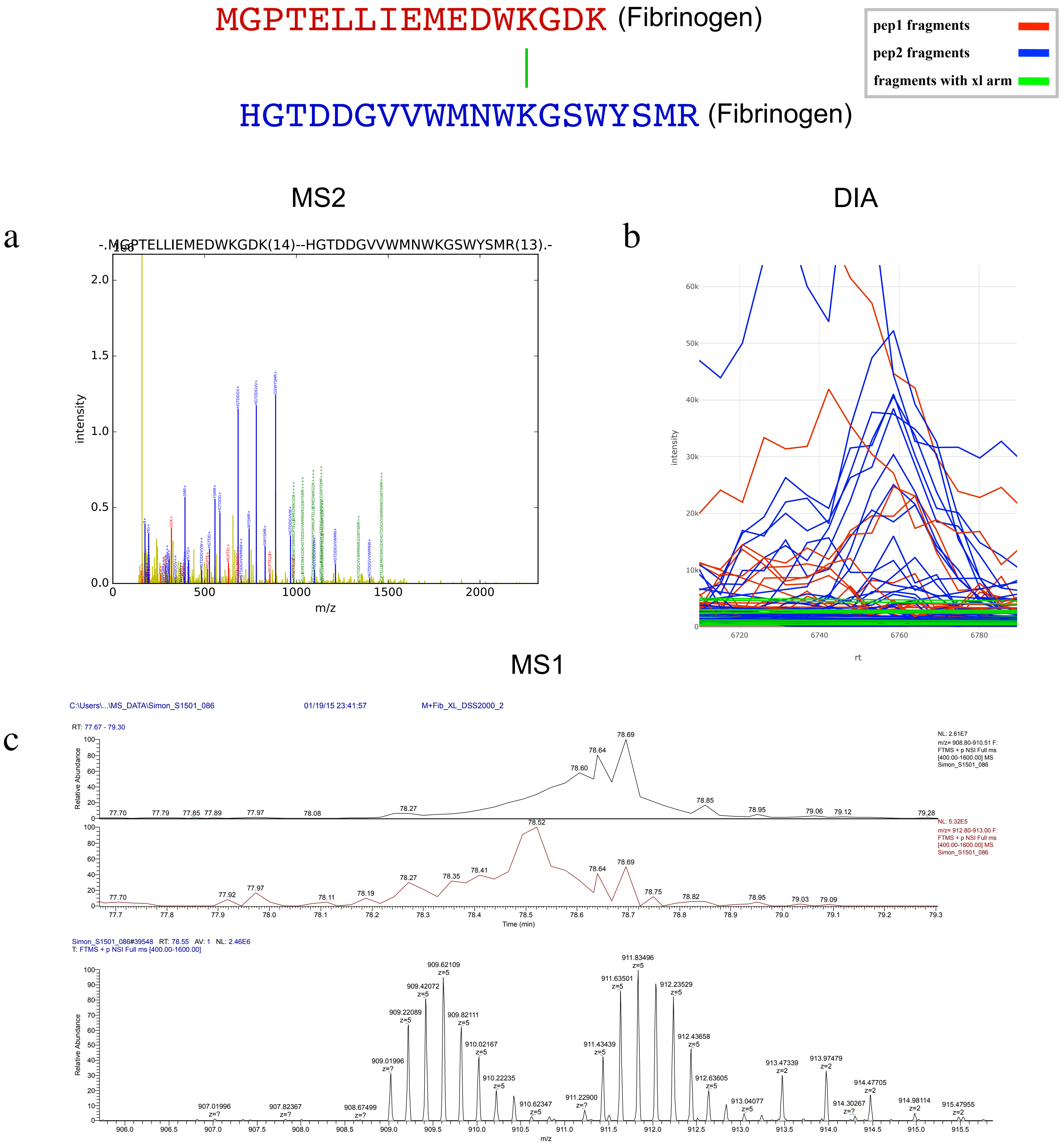
MGPTELLIEMEDWKGDK (Fibrinogen)
HGTDDGVVWMNWKGSWYSMR (Fibrinogen)
pep1 fragments
pep2 fragments
fragments with xl arm
MS2
DIA
a
-.MGPTELLIEMEDWKGDK(14)--HGTDDGVVWMNWKGSWYSMR(13).-
intensity
m/z
b
intensity
rt
MS1
c
C:\Users\...\MS_DATA\Simon_S1501_086
01/19/15 23:41:57
M+Fib_XL_DSS2000_2
RT: 77.67 - 79.30
NL: 2.61E7
m/z= 908.80-910.51 F: FTMS + p NSI Full ms [400.00-1600.00] MS Simon_S1501_086
NL: 5.32E5
m/z= 912.80-913.00 F: FTMS + p NSI Full ms [400.00-1600.00] MS Simon_S1501_086
Relative Abundance
Time (min)
Simon_S1501_086#39548 RT: 78.55 AV: 1 NL: 2.46E6
T: FTMS + p NSI Full ms [400.00-1600.00]
Relative Abundance
m/z

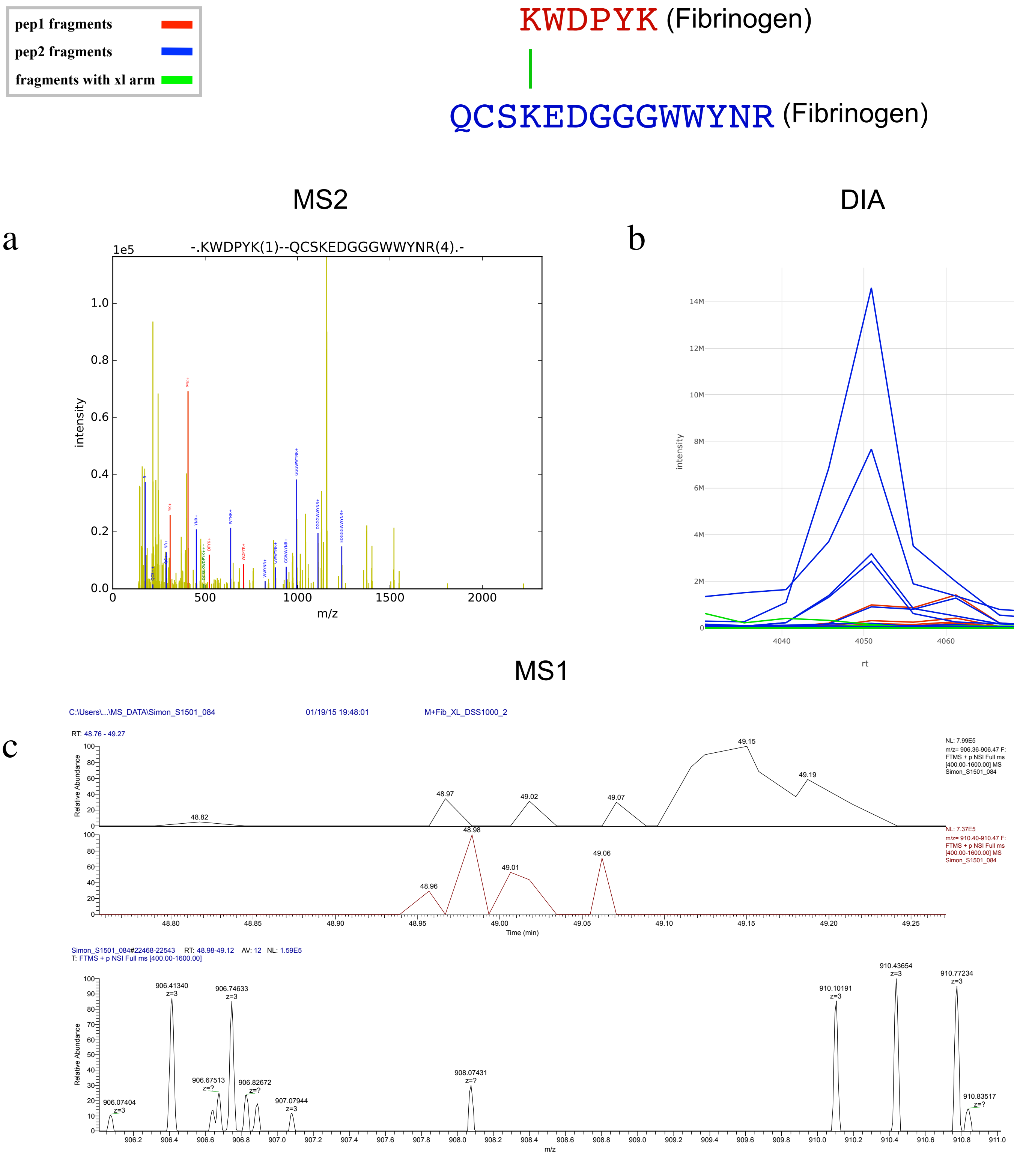
pep1 fragments
pep2 fragments
fragments with xl arm
KWDPYK (Fibrinogen)
QCSKEDGGGWWYNR (Fibrinogen)
MS2
DIA
a
-.KWDPYK(1)--QCSKEDGGGWWYNR(4).-
1e5
intensity
m/z
b
intensity
rt
MS1
C:\Users\...\MS_DATA\Simon_S1501_084
01/19/15 19:48:01
M+Fib_XL_DSS1000_2
c
RT: 48.76 - 49.27
Relative Abundance
NL: 7.99E5
m/z= 906.36-906.47 F: FTMS + p NSI Full ms [400.00-1600.00] MS Simon_S1501_084
NL: 7.37E5
m/z= 910.40-910.47 F: FTMS + p NSI Full ms [400.00-1600.00] MS Simon_S1501_084
Time (min)
Simon_S1501_084#22468-22543 RT: 48.98-49.12 AV: 12 NL: 1.59E5
T: FTMS + p NSI Full ms [400.00-1600.00]
Relative Abundance
m/z

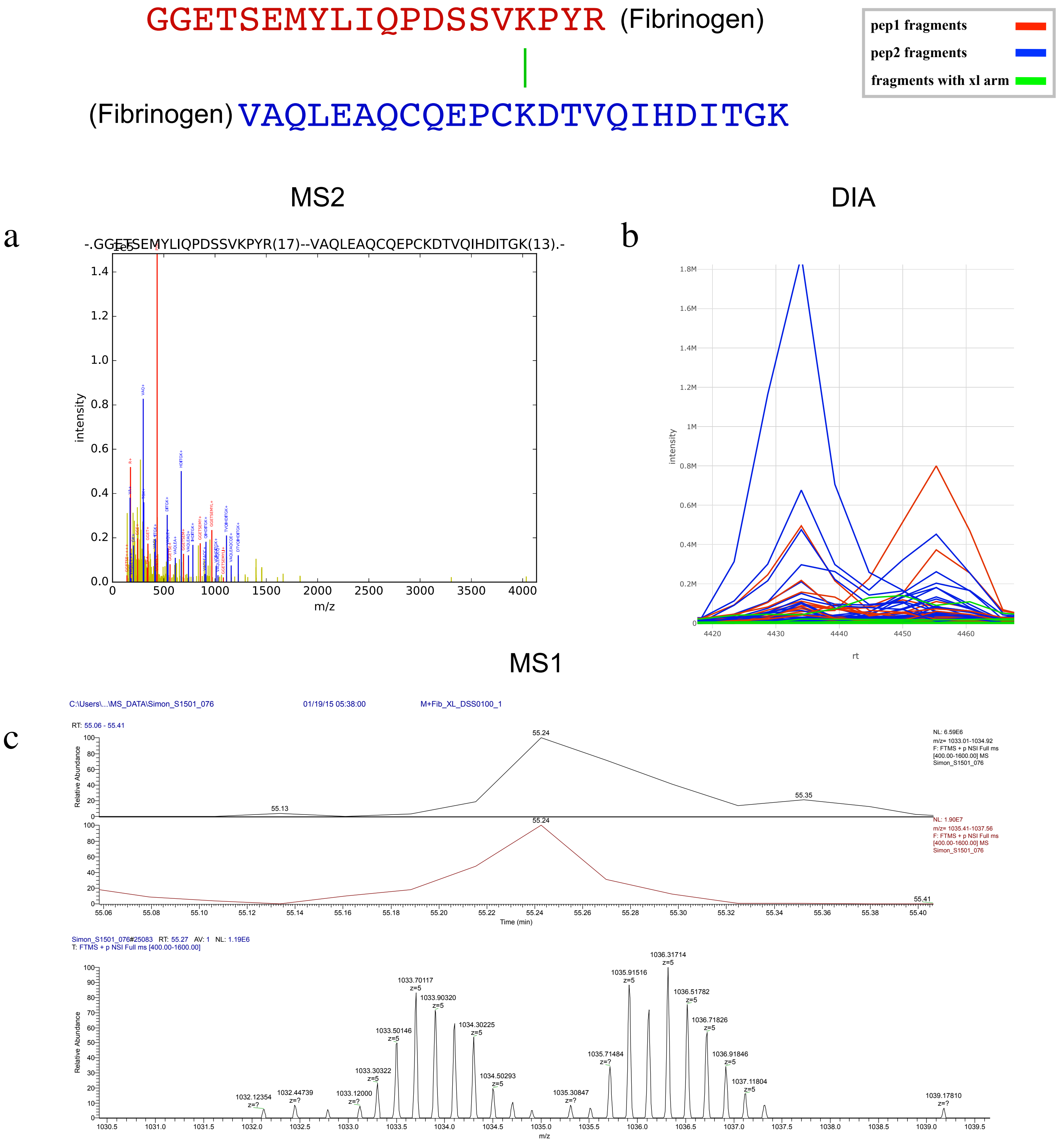

GGETSEMYLIQPDSSVKPYR (Fibrinogen)
(Fibrinogen) VAQLEAQCQEPCKDTVQIHDITGK
pep1 fragments
pep2 fragments
fragments with xl arm
MS2
DIA
a
-.GGETSEMYLIQPDSSVKPYR(17)--VAQLEAQCQEPCKDTVQIHDITGK(13).-
1e5
intensity
m/z
b
intensity
rt
MS1
c
C:\Users\...\MS_DATA\Simon_S1501_076
01/19/15 05:38:00
M+Fib_XL_DSS0100_1
RT: 55.06 - 55.41
Relative Abundance
NL: 6.59E6
m/z= 1033.01-1034.92
F: FTMS + p NSI Full ms [400.00-1600.00] MS Simon_S1501_076
NL: 1.90E7
m/z= 1035.41-1037.56
F: FTMS + p NSI Full ms [400.00-1600.00] MS Simon_S1501_076
Time (min)
Simon_S1501_076#25083 RT: 55.27 AV: 1 NL: 1.19E6
T: FTMS + p NSI Full ms [400.00-1600.00]
m/z

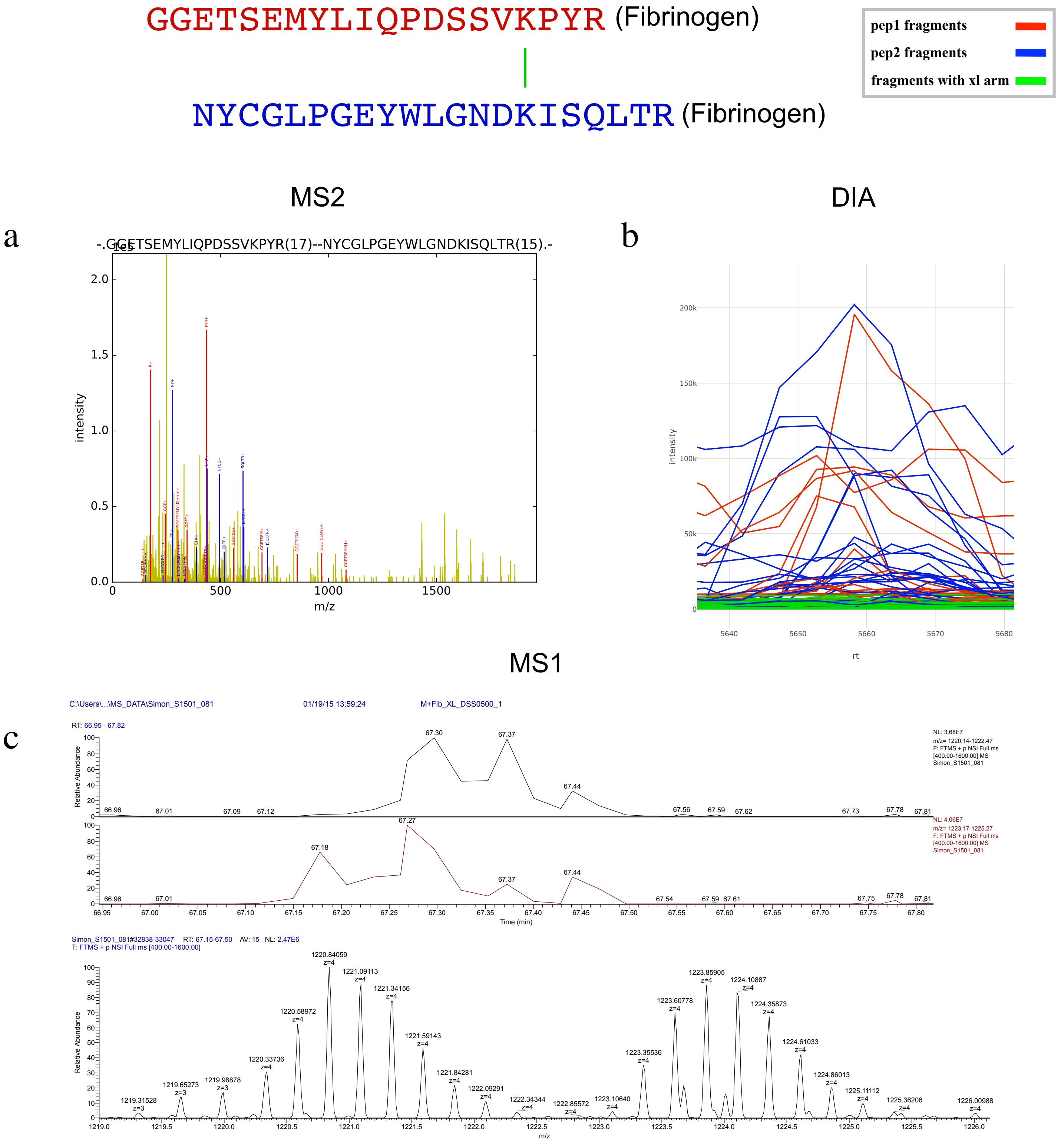
GGETSEMYLIQPDSSVKPYR (Fibrinogen)
NYCGLPGEYWLGNDKISQLTR (Fibrinogen)
pep1 fragments
pep2 fragments
fragments with xl arm
MS2
DIA
a
-.GGETSEMYLIQPDSSVKPYR(17)--NYCGLPGEYWLGNDKISQLTR(15).-
1e5
intensity
m/z
b
intensity
rt
MS1
c
C:\Users\...\MS_DATA\Simon_S1501_081
01/19/15 13:59:24
M+Fib_XL_DSS0500_1
RT: 66.95 - 67.82
Relative Abundance
NL: 3.68E7
m/z= 1220.14-1222.47
F: FTMS + p NSI Full ms [400.00-1600.00] MS Simon_S1501_081
NL: 4.06E7
m/z= 1223.17-1225.27
F: FTMS + p NSI Full ms [400.00-1600.00] MS Simon_S1501_081
Time (min)
Simon_S1501_081#32838-33047 RT: 67.15-67.50 AV: 15 NL: 2.47E6
T: FTMS + p NSI Full ms [400.00-1600.00]
Relative Abundance
m/z

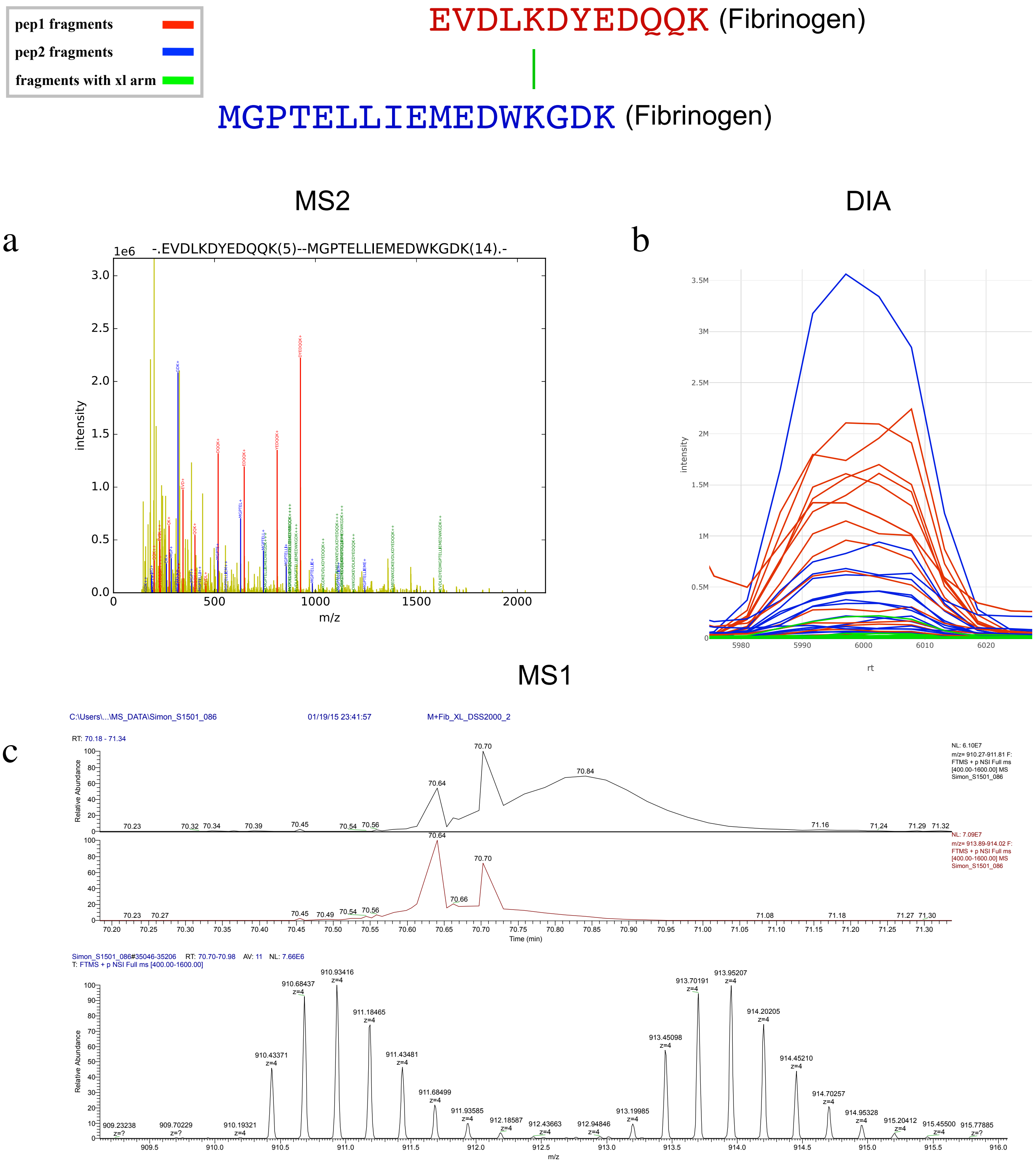
pep1 fragments
pep2 fragments
fragments with xl arm
EVDLKDYEDQQK (Fibrinogen)
MGPTELLIEMEDWKGDK (Fibrinogen)
MS2
DIA
a
-.EVDLKDYEDQQK(5)--MGPTELLIEMEDWKGDK(14).-
1e6
intensity
m/z
b
intensity
rt
MS1
c
C:\Users\...\MS_DATA\Simon_S1501_086
01/19/15 23:41:57
M+Fib_XL_DSS2000_2
RT: 70.18 - 71.34
NL: 6.10E7
m/z= 910.27-911.81 F: FTMS + p NSI Full ms [400.00-1600.00] MS Simon_S1501_086
NL: 7.09E7
m/z= 913.89-914.02 F: FTMS + p NSI Full ms [400.00-1600.00] MS Simon_S1501_086
Relative Abundance
Time (min)
Simon_S1501_086#35046-35206 RT: 70.70-70.98 AV: 11 NL: 7.66E6
T: FTMS + p NSI Full ms [400.00-1600.00]
Relative Abundance
m/z

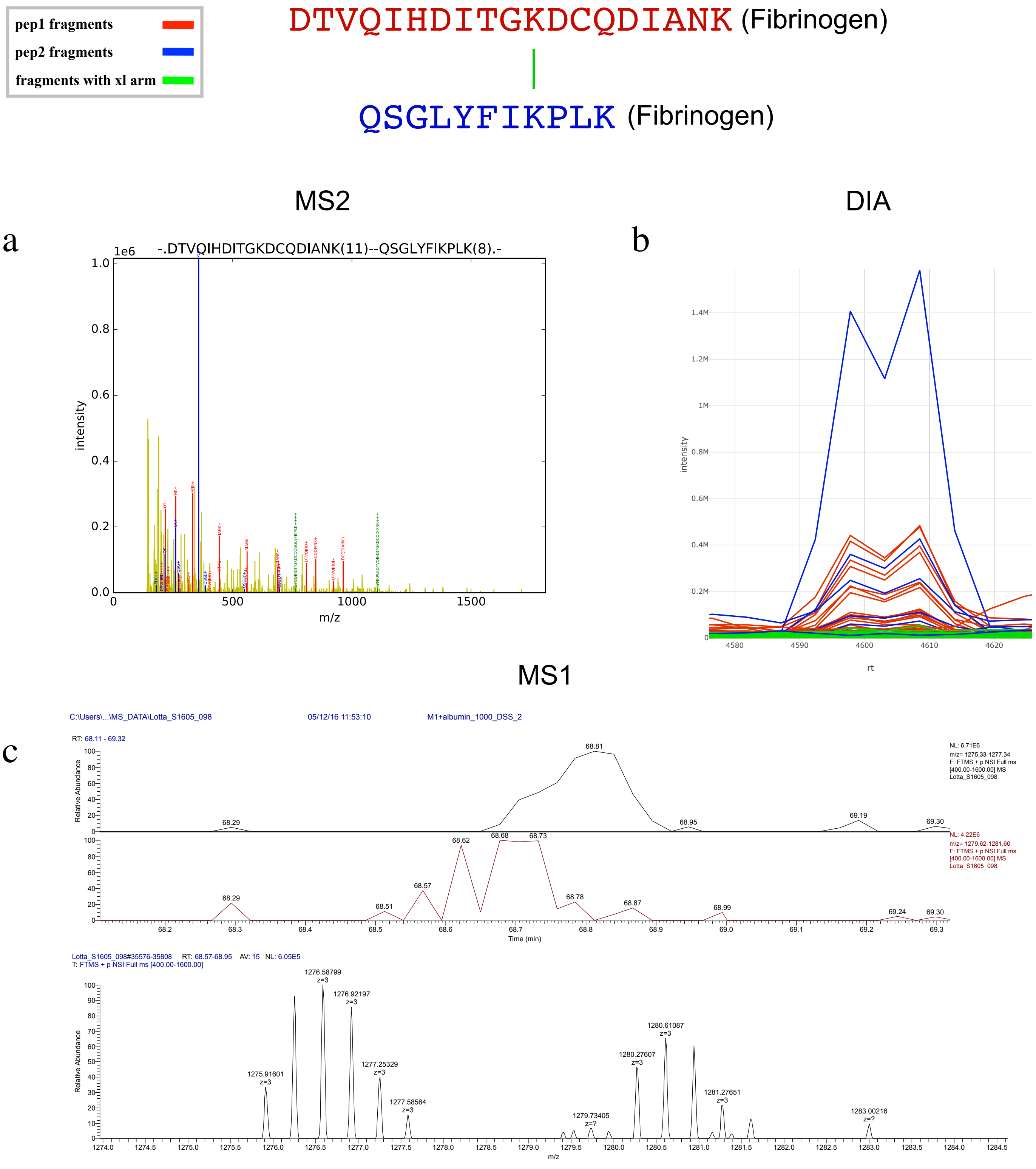
pep1 fragments
pep2 fragments
fragments with xl arm
DTVQIHDITGKDCQDIANK (Fibrinogen)
QSGLYFIKPLK (Fibrinogen)
MS2
DIA
a
-.DTVQIHDITGKDCQDIANK(11)--QSGLYFIKPLK(8).-
1e6
intensity
m/z
b
intensity
rt
MS1
c
C:\Users\...\MS_DATA\Lotta_S1605_098
05/12/16 11:53:10
M1+albumin_1000_DSS_2
RT: 68.11 - 69.32
Relative Abundance
NL: 6.71E6
m/z= 1275.33-1277.34
F: FTMS + p NSI Full ms [400.00-1600.00] MS
Lotta_S1605_098
NL: 4.22E6
m/z= 1279.62-1281.60
F: FTMS + p NSI Full ms [400.00-1600.00] MS
Lotta_S1605_098
Time (min)
Lotta_S1605_098#35576-35808 RT: 68.57-68.95 AV: 15 NL: 6.05E5
T: FTMS + p NSI Full ms [400.00-1600.00]
1275.91601 z=3
1276.58799 z=3
1276.92197 z=3
1277.25329 z=3
1277.58564 z=3
1279.73405 z=?
1280.27607 z=3
1280.61087 z=3
1281.27651 z=3
1283.00216 z=?
m/z

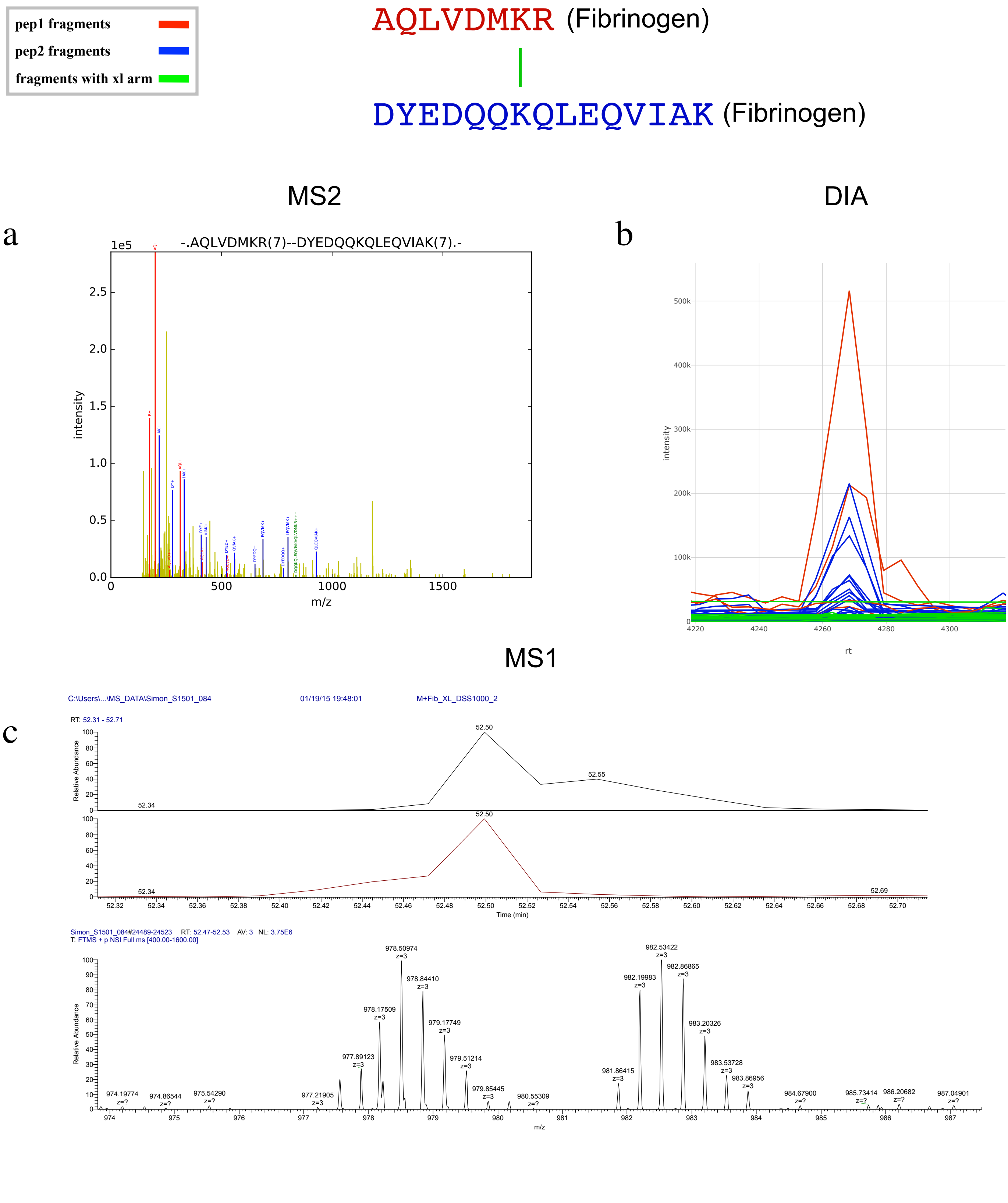
pep1 fragments
pep2 fragments
fragments with xl arm
AQLVDMKR (Fibrinogen)
DYEDQQKQLEQVIAK (Fibrinogen)
MS2
DIA
a
-.AQLVDMKR(7)--DYEDQQKQLEQVIAK(7).-
intensity
m/z
b
intensity
rt
MS1
c
C:\Users\...\MS_DATA\Simon_S1501_084
01/19/15 19:48:01
M+Fib_XL_DSS1000_2
RT: 52.31 - 52.71
Relative Abundance
Time (min)
Simon_S1501_084#24489-24523 RT: 52.47-52.53 AV: 3 NL: 3.75E6
T: FTMS + p NSI Full ms [400.00-1600.00]
978.50974 z=3
982.53422 z=3
m/z

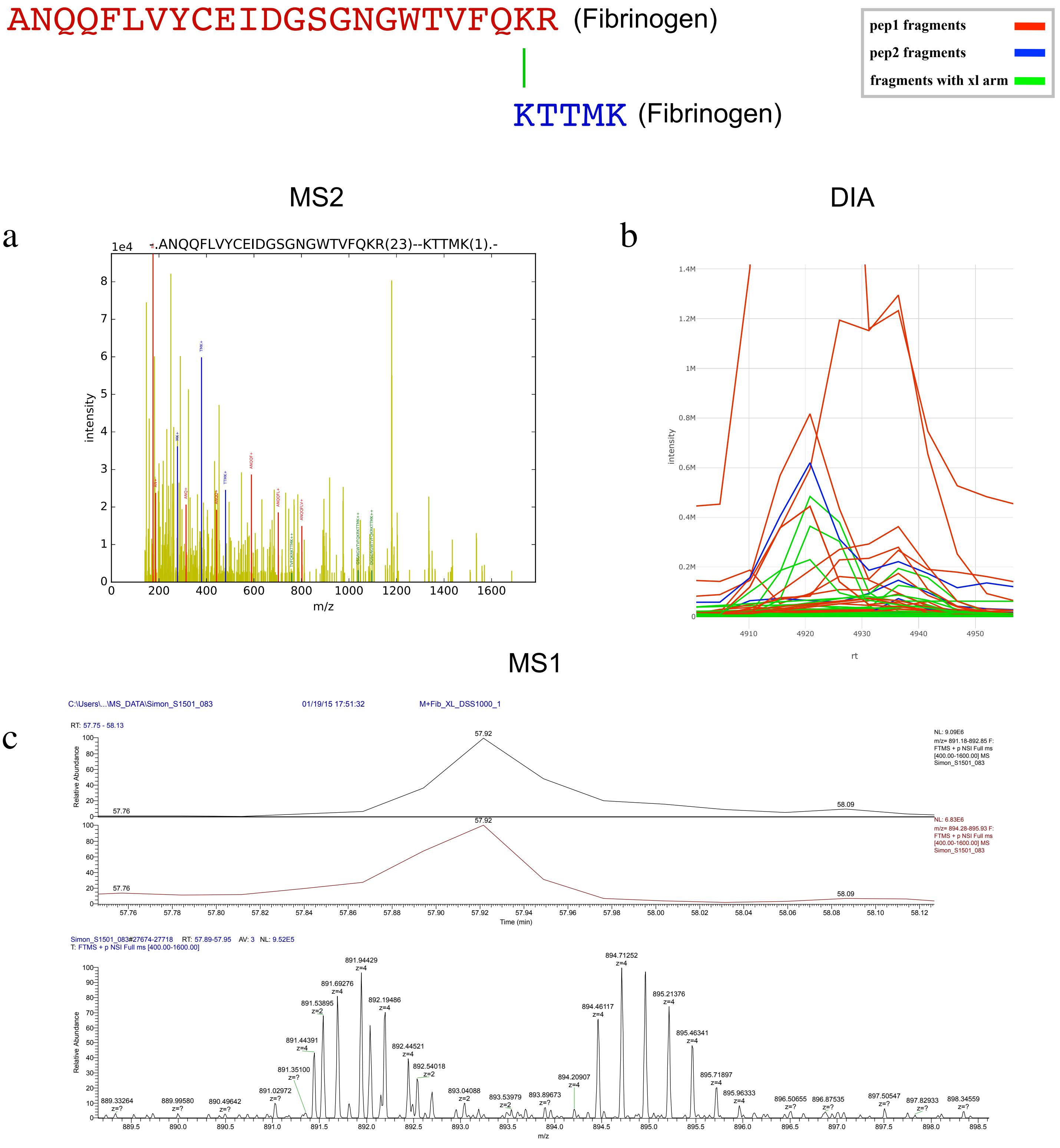
ANQQFLVYCEIDGSGNGWTVFQKR (Fibrinogen)
KTTMK (Fibrinogen)
pep1 fragments
pep2 fragments
fragments with xl arm
MS2
DIA
a
b
-.ANQQFLVYCEIDGSGNGWTVFQKR(23)--KTTMK(1).-
1e4
intensity
m/z
rt
MS1
c
C:\Users\...\MS_DATA\Simon_S1501_083
01/19/15 17:51:32
M+Fib_XL_DSS1000_1
RT: 57.75 - 58.13
NL: 9.09E6
m/z= 891.18-892.85 F: FTMS + p NSI Full ms [400.00-1600.00] MS Simon_S1501_083
NL: 6.83E6
m/z= 894.28-895.93 F: FTMS + p NSI Full ms [400.00-1600.00] MS Simon_S1501_083
Relative Abundance
Time (min)
Simon_S1501_083#27674-27718 RT: 57.89-57.95 AV: 3 NL: 9.52E5
T: FTMS + p NSI Full ms [400.00-1600.00]

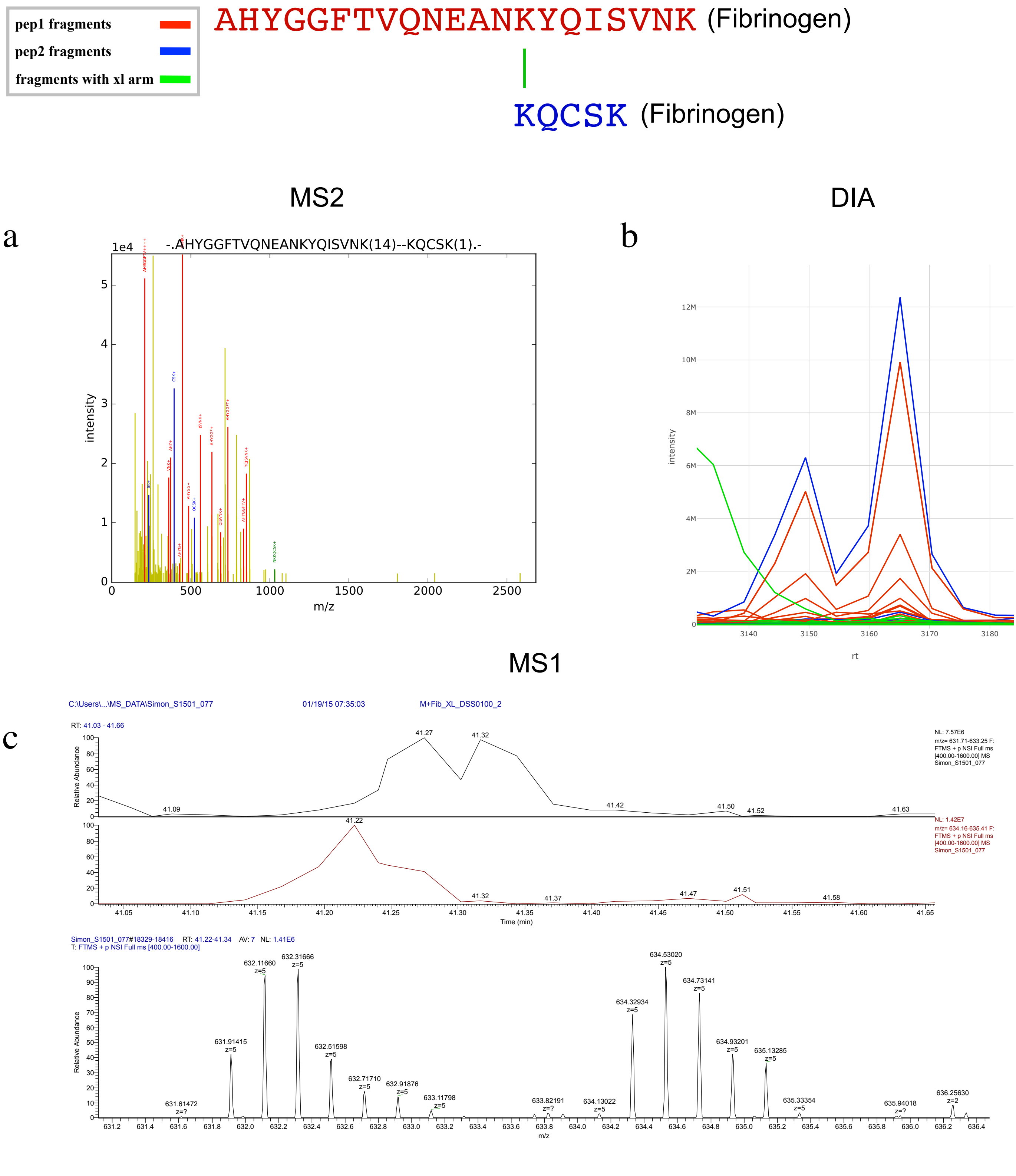
pep1 fragments
pep2 fragments
fragments with xl arm
AHYGGFTVQNEANKYQISVNK (Fibrinogen)
KQCSK (Fibrinogen)
MS2
DIA
a
-.AHYGGFTVQNEANKYQISVNK(14)--KQCSK(1).-
1e4
intensity
m/z
b
intensity
rt
MS1
c
C:\Users\...\MS_DATA\Simon_S1501_077
01/19/15 07:35:03
M+Fib_XL_DSS0100_2
RT: 41.03 - 41.66
Relative Abundance
Time (min)
Simon_S1501_077#18329-18416 RT: 41.22-41.34 AV: 7 NL: 1.41E6
T: FTMS + p NSI Full ms [400.00-1600.00]
NL: 7.57E6
m/z= 631.71-633.25 F: FTMS + p NSI Full ms [400.00-1600.00] MS Simon_S1501_077
NL: 1.42E7
m/z= 634.16-635.41 F: FTMS + p NSI Full ms [400.00-1600.00] MS Simon_S1501_077
m/z

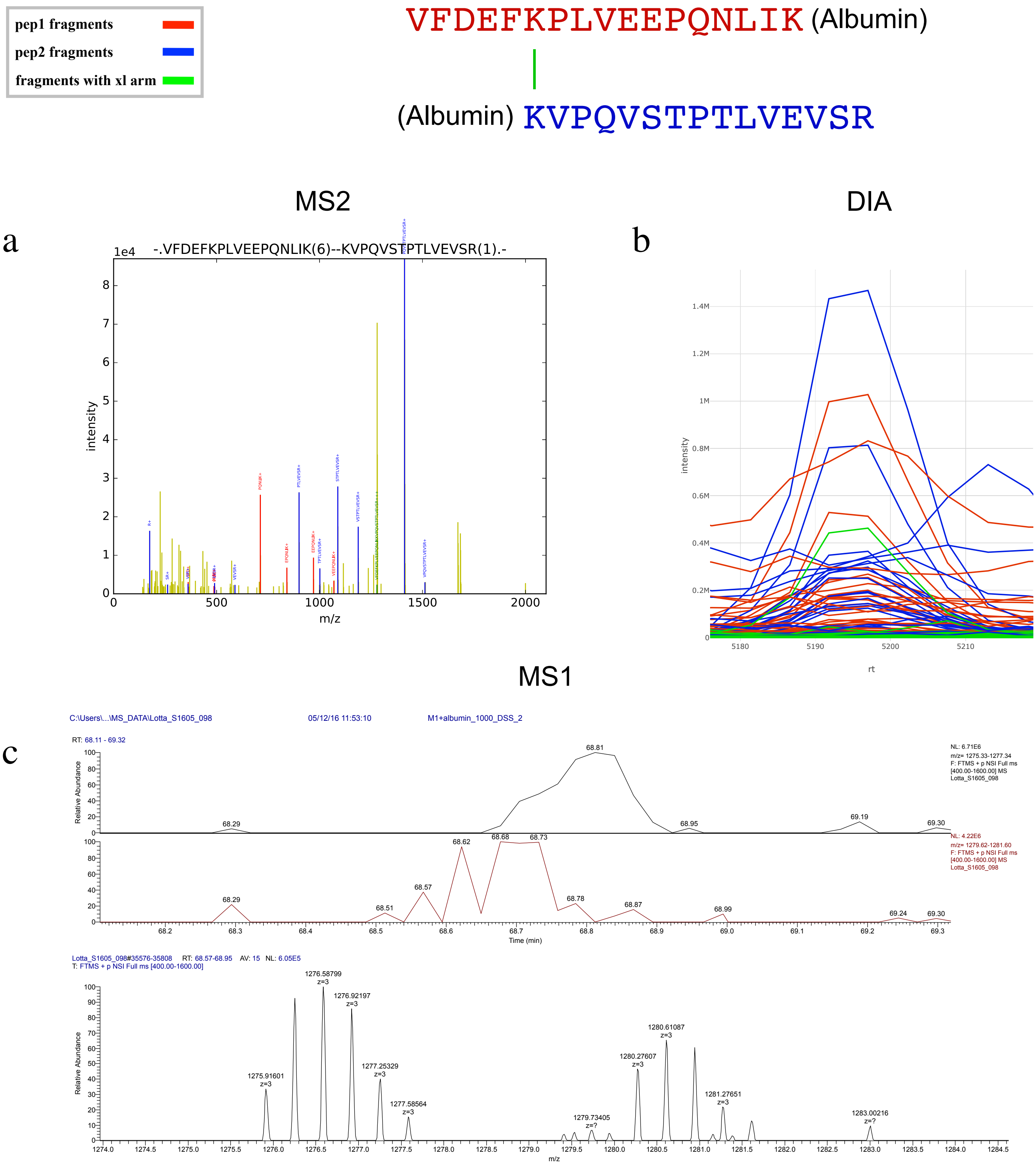

pep1 fragments
pep2 fragments
fragments with xl arm
VFDEFKPLVEEPQNLIK (Albumin)
(Albumin) KVPQVSTPTLVEVSR
MS2
DIA
a
b
-.VFDEFKPLVEEPQNLIK(6)--KVPQVSTPTLVEVSR(1).-
1e4
intensity
m/z
rt
MS1
c
C:\Users\...\MS_DATA\Lotta_S1605_098
05/12/16 11:53:10
M1+albumin_1000_DSS_2
RT: 68.11 - 69.32
Relative Abundance
NL: 6.71E6
m/z= 1275.33-1277.34
F: FTMS + p NSI Full ms [400.00-1600.00] MS Lotta_S1605_098
NL: 4.22E6
m/z= 1279.62-1281.60
F: FTMS + p NSI Full ms [400.00-1600.00] MS Lotta_S1605_098
Time (min)
Lotta_S1605_098#35576-35808 RT: 68.57-68.95 AV: 15 NL: 6.05E5
T: FTMS + p NSI Full ms [400.00-1600.00]
1275.91601 z=3
1276.58799 z=3
1276.92197 z=3
1277.25329 z=3
1277.58564 z=3
1279.73405 z=?
1280.27607 z=3
1280.61087 z=3
1281.27651 z=3
1283.00216 z=?
m/z

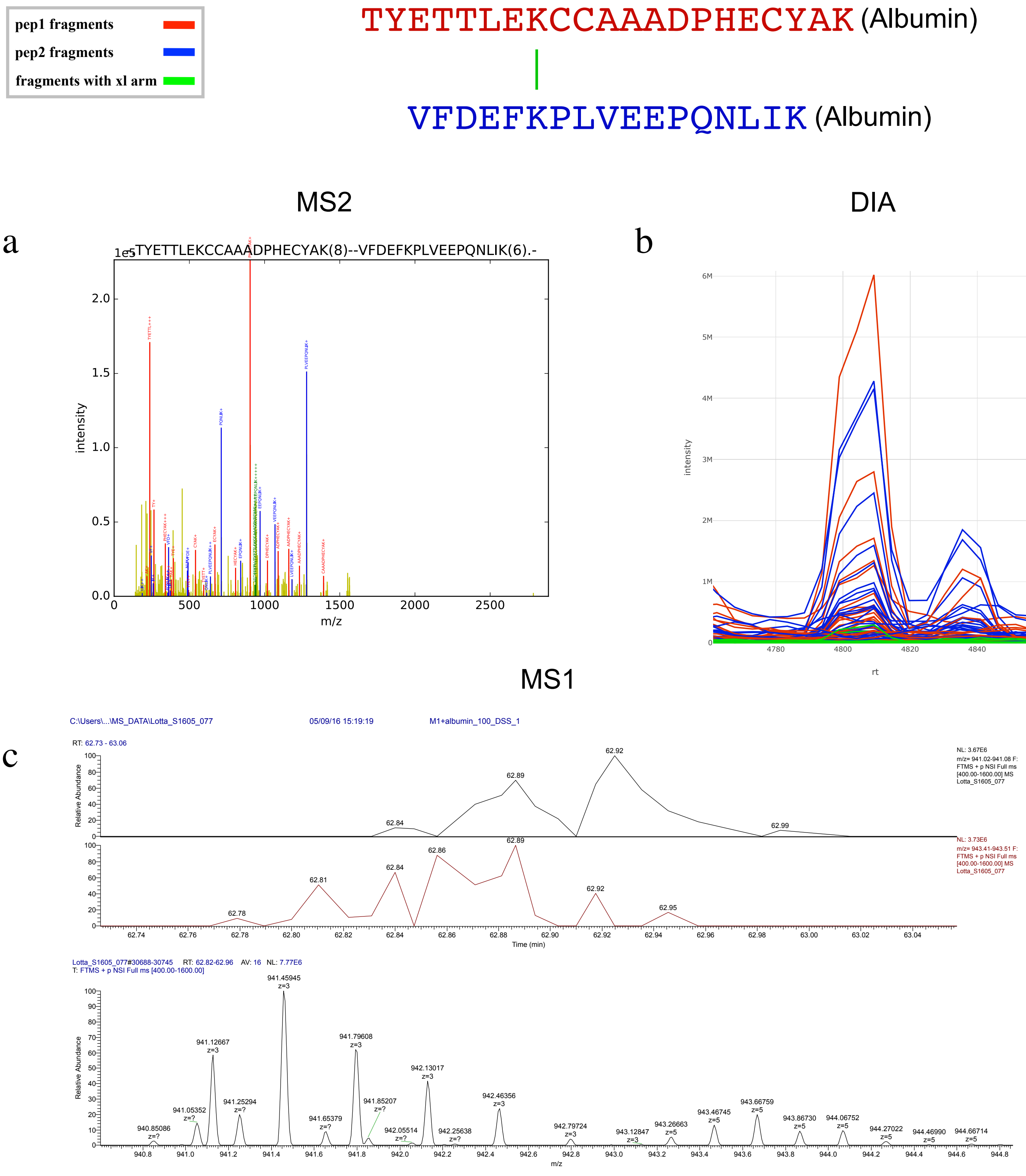

pep1 fragments
pep2 fragments
fragments with xl arm
TYETTLEKCCAAADPHECYAK (Albumin)
VFDEFKPLVEEPQNLIK (Albumin)
MS2
DIA
a
TYETTLEKCCAAADPHECYAK(8)--VFDEFKPLVEEPQNLIK(6).-
intensity
m/z
b
intensity
rt
MS1
c
C:\Users\...\MS_DATA\Lotta_S1605_077
05/09/16 15:19:19
M1+albumin_100_DSS_1
RT: 62.73 - 63.06
NL: 3.67E6
m/z= 941.02-941.08 F: FTMS + p NSI Full ms [400.00-1600.00] MS Lotta_S1605_077
NL: 3.73E6
m/z= 943.41-943.51 F: FTMS + p NSI Full ms [400.00-1600.00] MS Lotta_S1605_077
Relative Abundance
Time (min)
Lotta_S1605_077#30688-30745 RT: 62.82-62.96 AV: 16 NL: 7.77E6
T: FTMS + p NSI Full ms [400.00-1600.00]
Relative Abundance
m/z

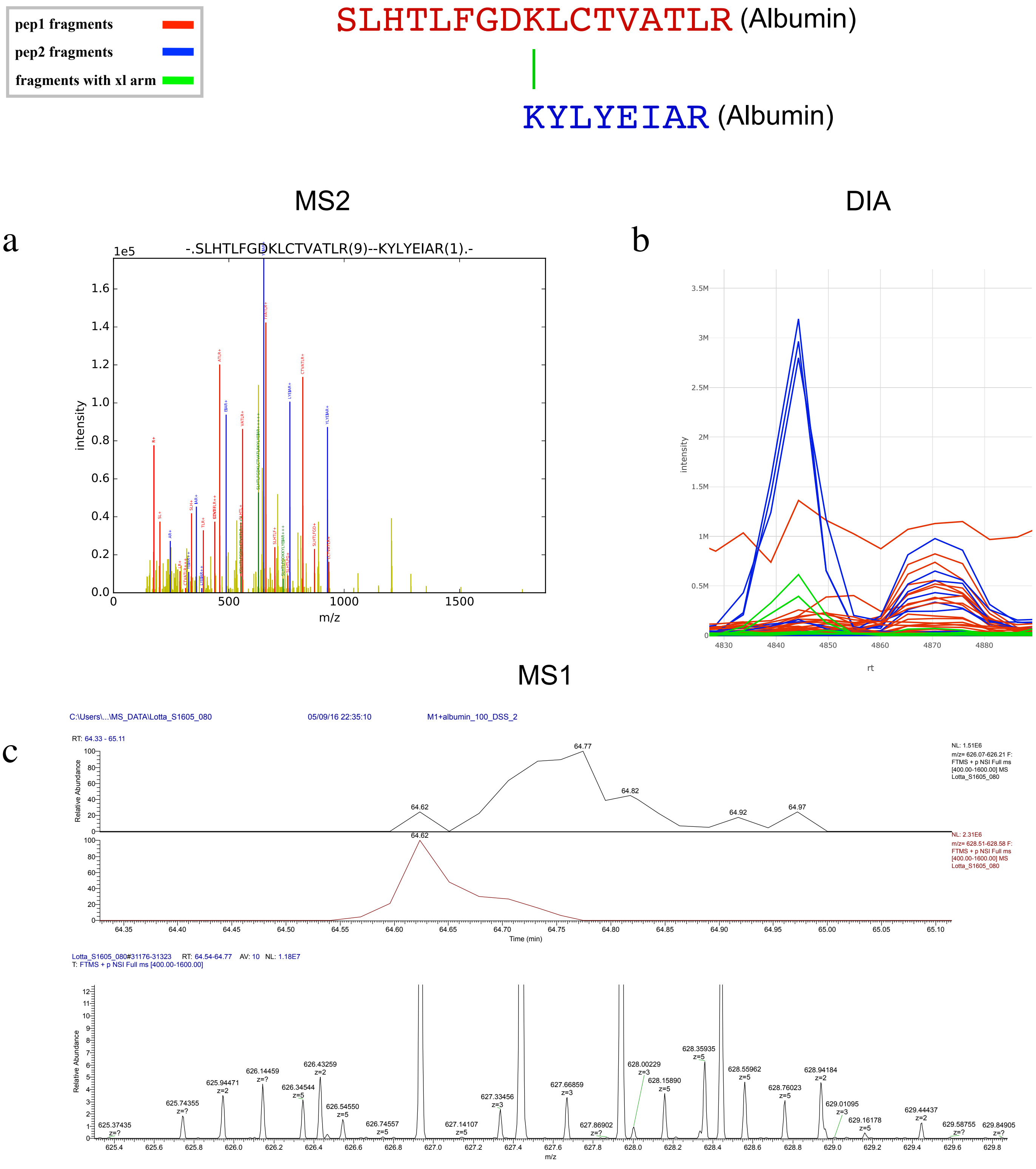

pep1 fragments
pep2 fragments
fragments with xl arm
SLHTLFGDKLCTVATLR (Albumin)
KYLYEIAR (Albumin)
MS2
DIA
a
-.SLHTLFGDKLCTVATLR(9)--KYLYEIAR(1).-
1e5
intensity
m/z
b
intensity
rt
MS1
c
C:\Users\...\MS_DATA\Lotta_S1605_080
05/09/16 22:35:10
M1+albumin_100_DSS_2
RT: 64.33 - 65.11
NL: 1.51E6
m/z= 626.07-626.21 F: FTMS + p NSI Full ms [400.00-1600.00] MS Lotta_S1605_080
NL: 2.31E6
m/z= 628.51-628.58 F: FTMS + p NSI Full ms [400.00-1600.00] MS Lotta_S1605_080
Relative Abundance
Time (min)
Lotta_S1605_080#31176-31323 RT: 64.54-64.77 AV: 10 NL: 1.18E7
T: FTMS + p NSI Full ms [400.00-1600.00]
Relative Abundance
m/z

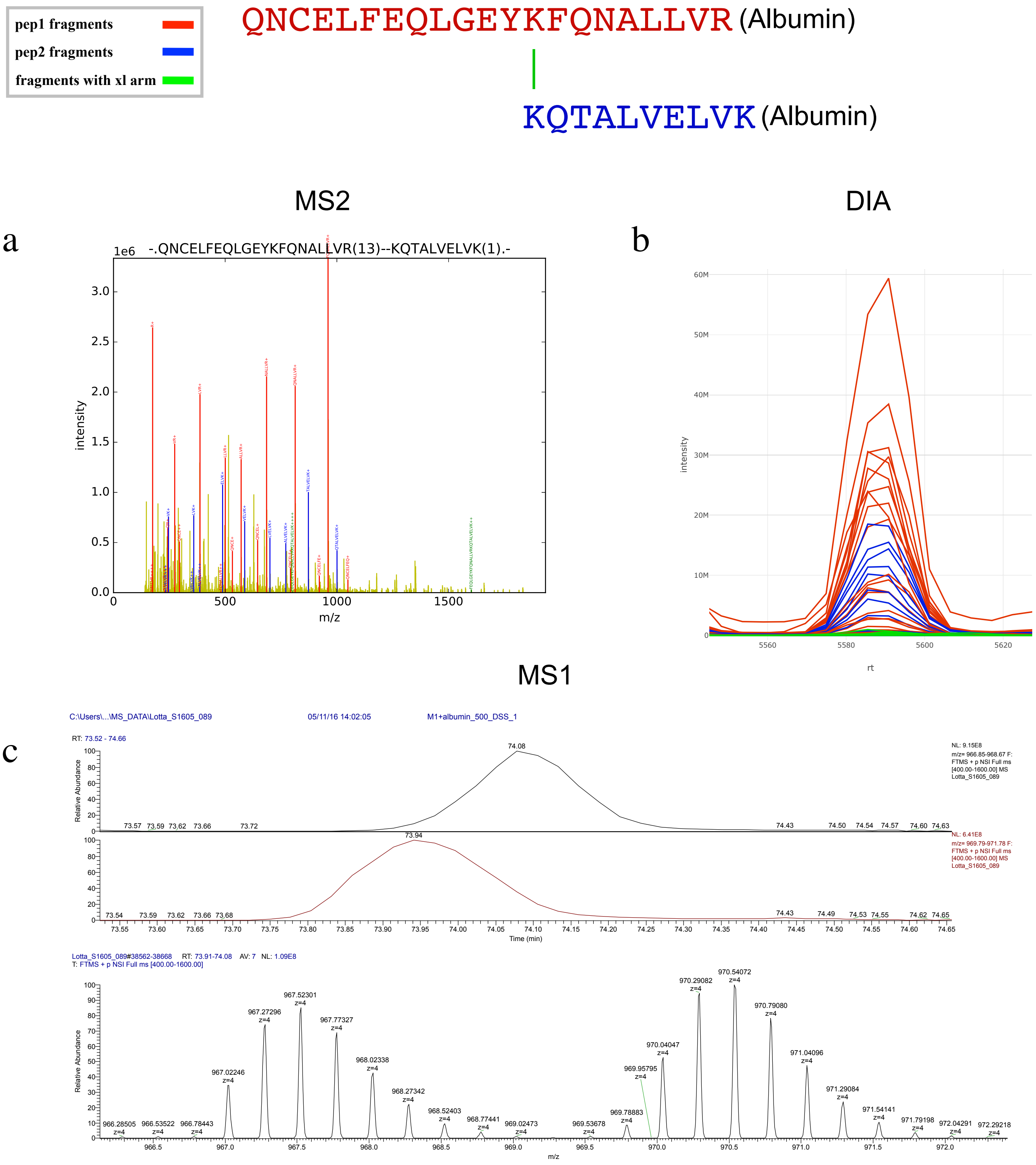
pep1 fragments
pep2 fragments
fragments with xl arm
QNCELFEQLGEYKFQNALLVR (Albumin)
KQTALVELVK (Albumin)
MS2
DIA
a
-.QNCELFEQLGEYKFQNALLVR(13)--KQTALVELVK(1).-
1e6
intensity
m/z
b
intensity
rt
MS1
c
C:\Users\...\MS_DATA\Lotta_S1605_089
05/11/16 14:02:05
M1+albumin_500_DSS_1
RT: 73.52 - 74.66
NL: 9.15E8
m/z= 966.85-968.67 F: FTMS + p NSI Full ms [400.00-1600.00] MS Lotta_S1605_089
NL: 6.41E8
m/z= 969.79-971.78 F: FTMS + p NSI Full ms [400.00-1600.00] MS Lotta_S1605_089
Relative Abundance
Time (min)
Lotta_S1605_089#38562-38668 RT: 73.91-74.08 AV: 7 NL: 1.09E8
T: FTMS + p NSI Full ms [400.00-1600.00]
Relative Abundance
m/z

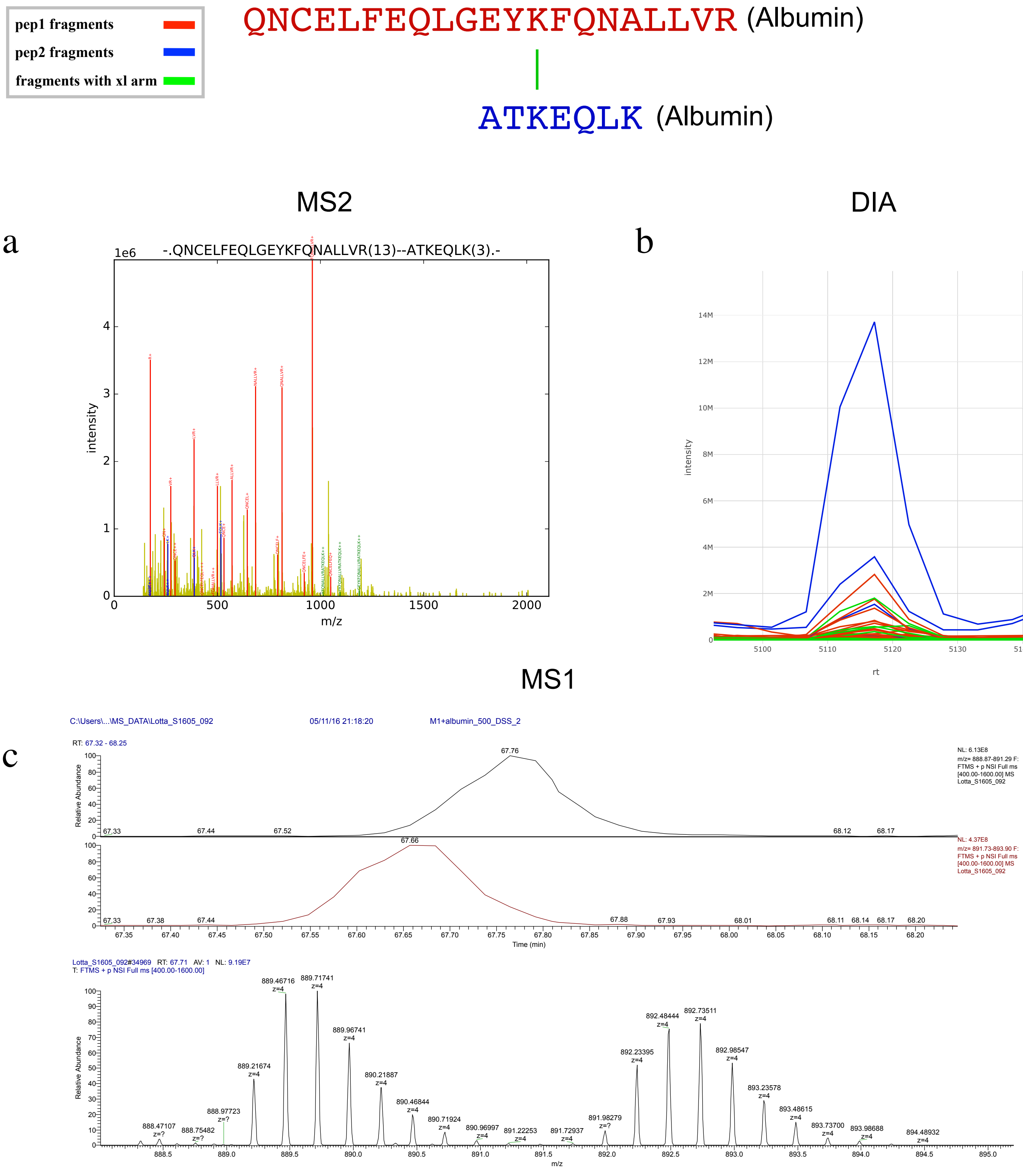

pep1 fragments
pep2 fragments
fragments with xl arm
QNCELFEQLGEYKFQNALLVR (Albumin)
ATKEQLK (Albumin)
MS2
DIA
a
-.QNCELFEQLGEYKFQNALLVR(13)--ATKEQLK(3).-
1e6
intensity
m/z
b
intensity
rt
MS1
c
C:\Users\...\MS_DATA\Lotta_S1605_092
05/11/16 21:18:20
M1+albumin_500_DSS_2
RT: 67.32 - 68.25
Relative Abundance
NL: 6.13E8
m/z= 888.87-891.29 F: FTMS + p NSI Full ms [400.00-1600.00] MS Lotta_S1605_092
NL: 4.37E8
m/z= 891.73-893.90 F: FTMS + p NSI Full ms [400.00-1600.00] MS Lotta_S1605_092
Time (min)
Lotta_S1605_092#34969 RT: 67.71 AV: 1 NL: 9.19E7
T: FTMS + p NSI Full ms [400.00-1600.00]
Relative Abundance
m/z

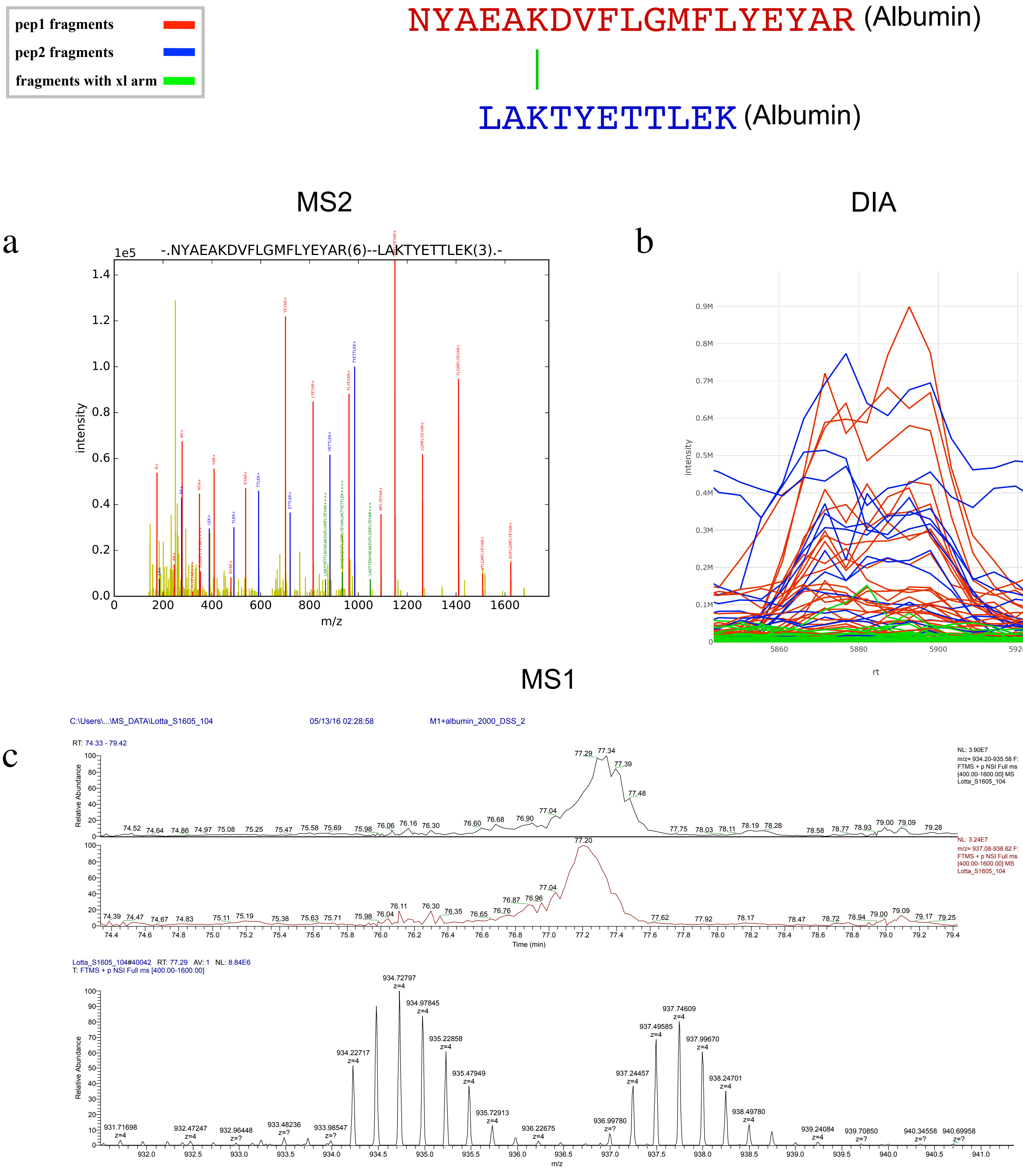
pep1 fragments
pep2 fragments
fragments with xl arm
NYAEAKDVFLGMFLYEYAR (Albumin)
LAKTYETTLEK (Albumin)
MS2
DIA
a
-.NYAEAKDVFLGMFLYEYAR(6)--LAKTYETTLEK(3).-
intensity
m/z
b
intensity
rt
MS1
c
C:\Users\...\MS_DATA\Lotta_S1605_104
05/13/16 02:28:58
M1+albumin_2000_DSS_2
RT: 74.33 - 79.42
NL: 3.90E7
m/z= 934.20-935.58 F: FTMS + p NSI Full ms [400.00-1600.00] MS Lotta_S1605_104
NL: 3.24E7
m/z= 937.08-938.62 F: FTMS + p NSI Full ms [400.00-1600.00] MS Lotta_S1605_104
Relative Abundance
Time (min)
Lotta_S1605_104#40042 RT: 77.29 AV: 1 NL: 8.84E6
T: FTMS + p NSI Full ms [400.00-1600.00]
Relative Abundance
m/z

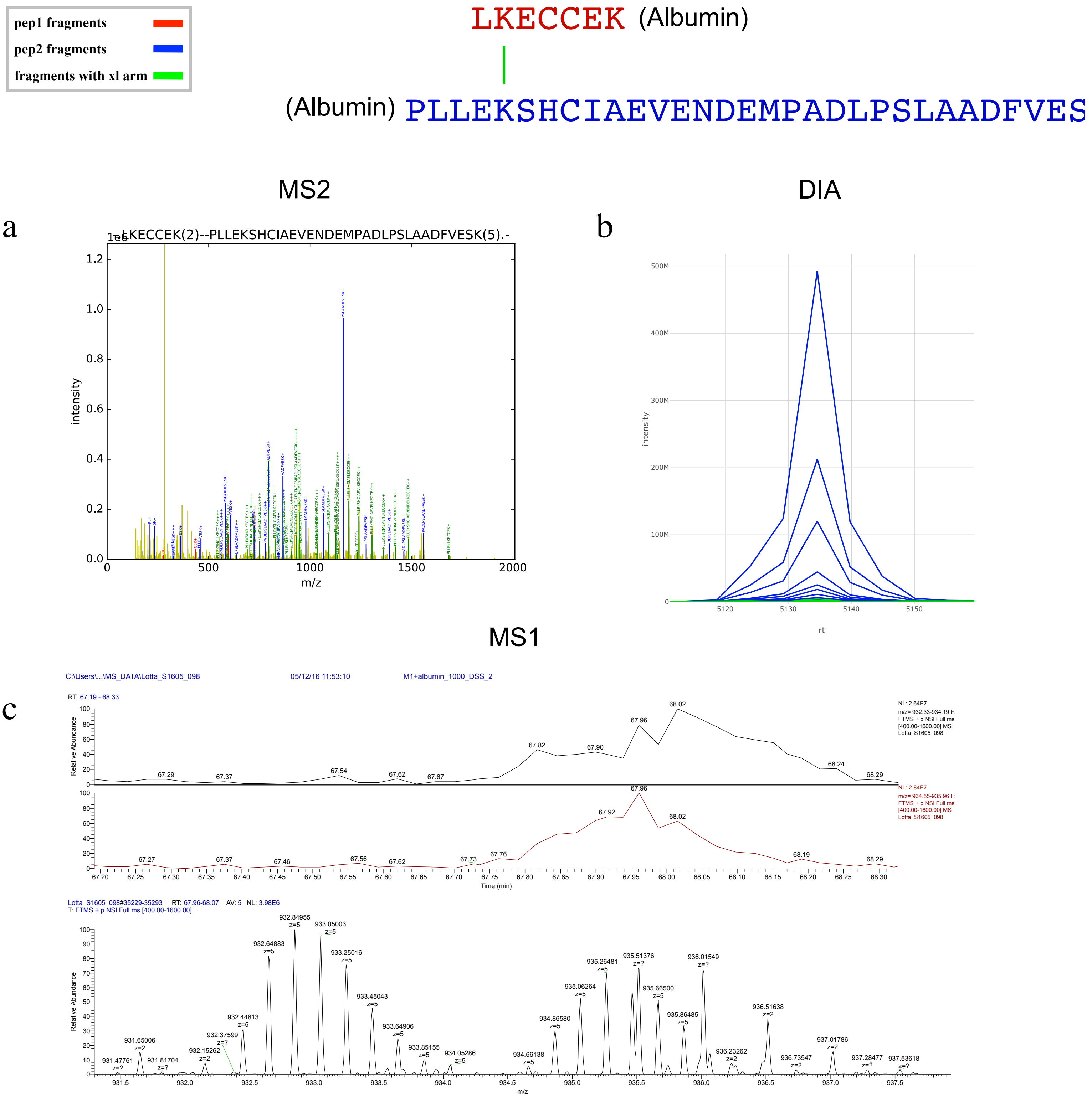

pep1 fragments
pep2 fragments
fragments with xl arm
LKECCEK (Albumin)
(Albumin) PLLEKSHCIAEVENDEMPADLPSLAADFVES
MS2
DIA
a
LKECCEK(2)--PLLEKSHCIAEVENDEMPADLPSLAADFVESK(5).-
intensity
m/z
b
intensity
rt
MS1
c
C:\Users\...\MS_DATA\Lotta_S1605_098
05/12/16 11:53:10
M1+albumin_1000_DSS_2
RT: 67.19 - 68.33
Relative Abundance
NL: 2.64E7
m/z= 932.33-934.19 F: FTMS + p NSI Full ms [400.00-1600.00] MS Lotta_S1605_098
NL: 2.84E7
m/z= 934.55-935.96 F: FTMS + p NSI Full ms [400.00-1600.00] MS Lotta_S1605_098
Time (min)
Lotta_S1605_098#35229-35293 RT: 67.96-68.07 AV: 5 NL: 3.98E6
T: FTMS + p NSI Full ms [400.00-1600.00]
Relative Abundance
m/z

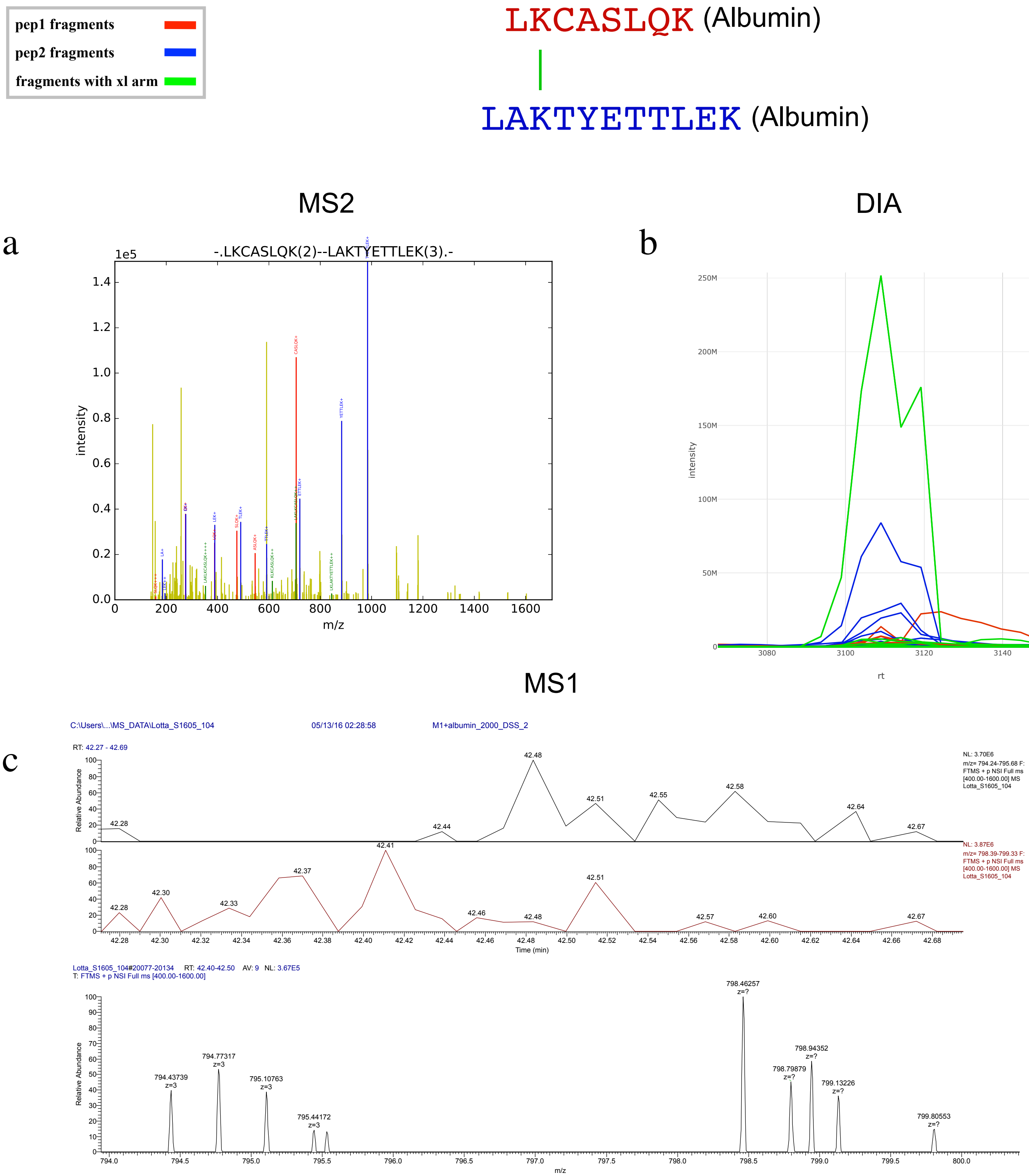

pep1 fragments
pep2 fragments
fragments with xl arm
LKCASLQK (Albumin)
LAKTYETTLEK (Albumin)
MS2
a
-.LKCASLQK(2)--LAKTYETTLEK(3).-
1e5
intensity
m/z
DIA
b
250M
200M
150M
100M
50M
0
intensity
3080
3100
3120
3140
rt
MS1
c
C:\Users\...\MS_DATA\Lotta_S1605_104
05/13/16 02:28:58
M1+albumin_2000_DSS_2
RT: 42.27 - 42.69
NL: 3.70E6
m/z= 794.24-795.68 F: FTMS + p NSI Full ms [400.00-1600.00] MS Lotta_S1605_104
NL: 3.87E6
m/z= 798.39-799.33 F: FTMS + p NSI Full ms [400.00-1600.00] MS Lotta_S1605_104
Relative Abundance
Time (min)
Lotta_S1605_104#20077-20134 RT: 42.40-42.50 AV: 9 NL: 3.67E5
T: FTMS + p NSI Full ms [400.00-1600.00]
794.43739 z=3
794.77317 z=3
795.10763 z=3
795.44172 z=3
798.46257 z=?
798.79879 z=?
798.94352 z=?
799.13226 z=?
799.80553 z=?
m/z

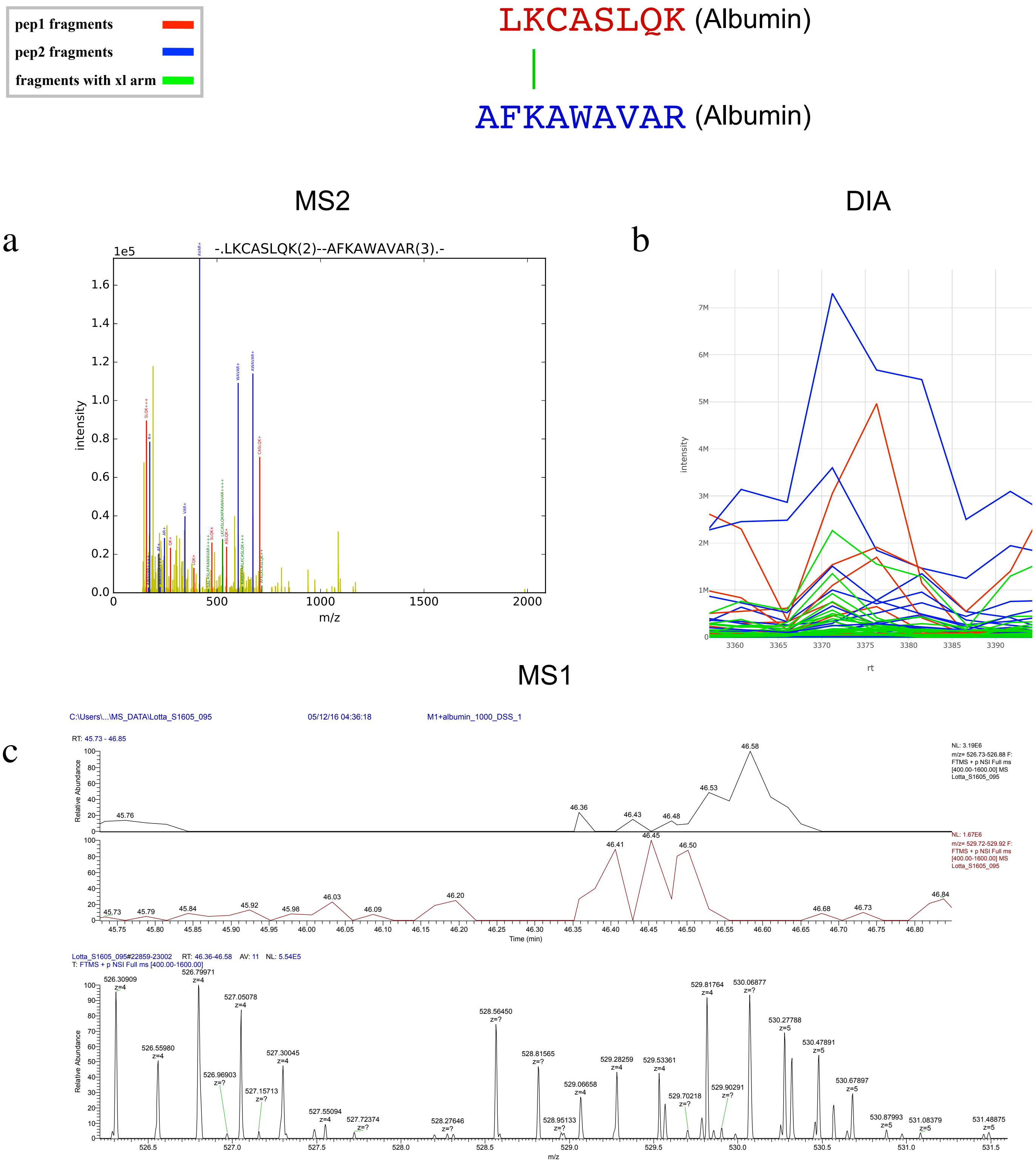

pep1 fragments
pep2 fragments
fragments with xl arm
LKCASLQK (Albumin)
AFKAWAVAR (Albumin)
MS2
DIA
a
-.LKCASLQK(2)--AFKAWAVAR(3).-
1e5
intensity
m/z
b
intensity
rt
MS1
c
C:\Users\...\MS_DATA\Lotta_S1605_095
05/12/16 04:36:18
M1+albumin_1000_DSS_1
RT: 45.73 - 46.85
Relative Abundance
NL: 3.19E6
m/z= 526.73-526.88 F: FTMS + p NSI Full ms [400.00-1600.00] MS Lotta_S1605_095
NL: 1.67E6
m/z= 529.72-529.92 F: FTMS + p NSI Full ms [400.00-1600.00] MS Lotta_S1605_095
Time (min)
Lotta_S1605_095#22859-23002 RT: 46.36-46.58 AV: 11 NL: 5.54E5
T: FTMS + p NSI Full ms [400.00-1600.00]
Relative Abundance
m/z

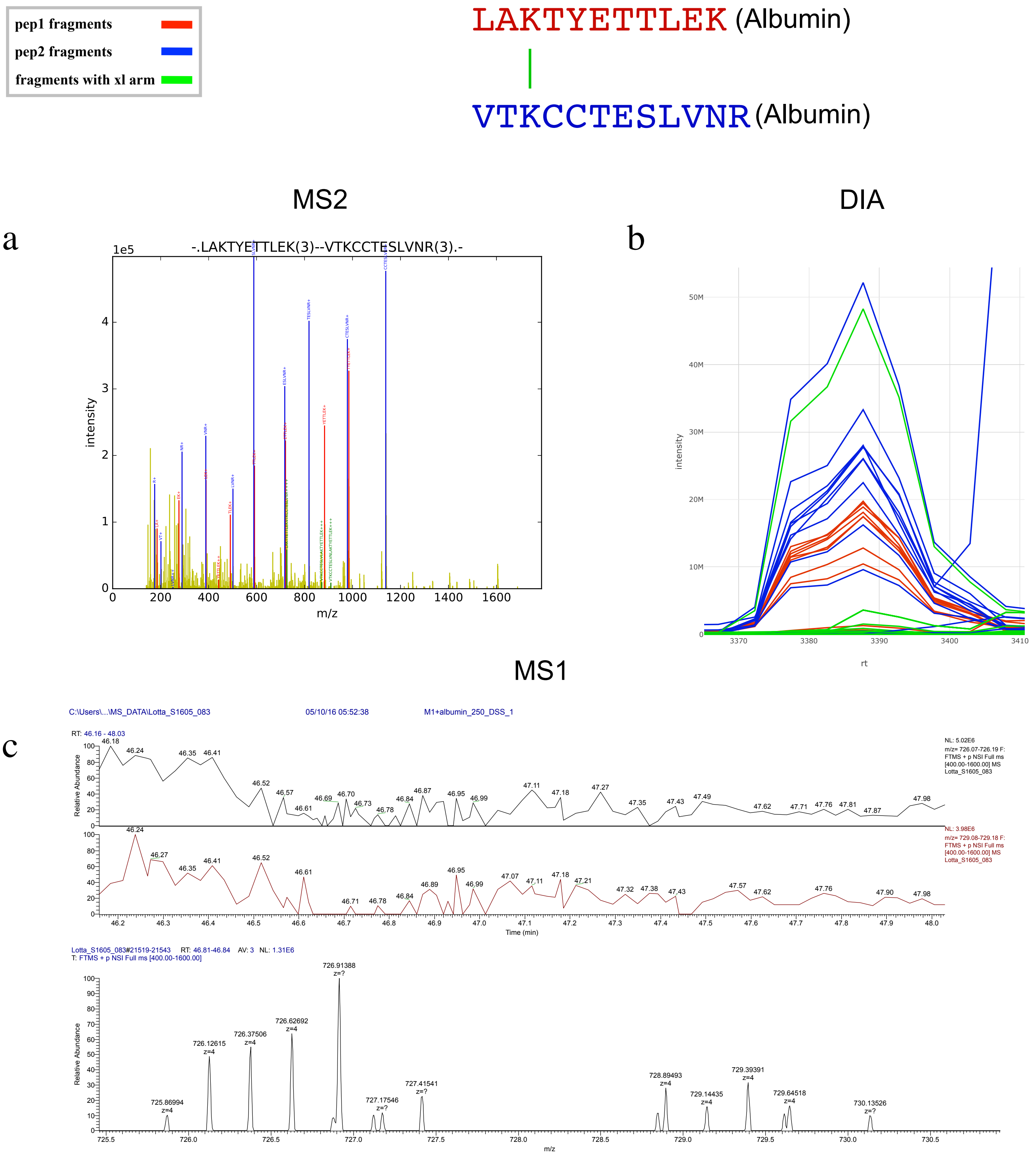
pep1 fragments
pep2 fragments
fragments with xl arm
LAKTYETTLEK (Albumin)
VTKCCTESLVNR (Albumin)
MS2
DIA
a
-.LAKTYETTLEK(3)--VTKCCTESLVNR(3).-
1e5
intensity
m/z
b
intensity
rt
MS1
c
C:\Users\...\MS_DATA\Lotta_S1605_083
05/10/16 05:52:38
M1+albumin_250_DSS_1
RT: 46.16 - 48.03
Relative Abundance
NL: 5.02E6
m/z= 726.07-726.19 F: FTMS + p NSI Full ms [400.00-1600.00] MS Lotta_S1605_083
NL: 3.98E6
m/z= 729.08-729.18 F: FTMS + p NSI Full ms [400.00-1600.00] MS Lotta_S1605_083
Time (min)
Lotta_S1605_083#21519-21543 RT: 46.81-46.84 AV: 3 NL: 1.31E6
T: FTMS + p NSI Full ms [400.00-1600.00]
725.86994 z=4
726.12615 z=4
726.37506 z=4
726.62692 z=4
726.91388 z=?
727.17546 z=?
727.41541 z=?
728.89493 z=4
729.14435 z=4
729.39391 z=4
729.64518 z=4
730.13526 z=?
m/z

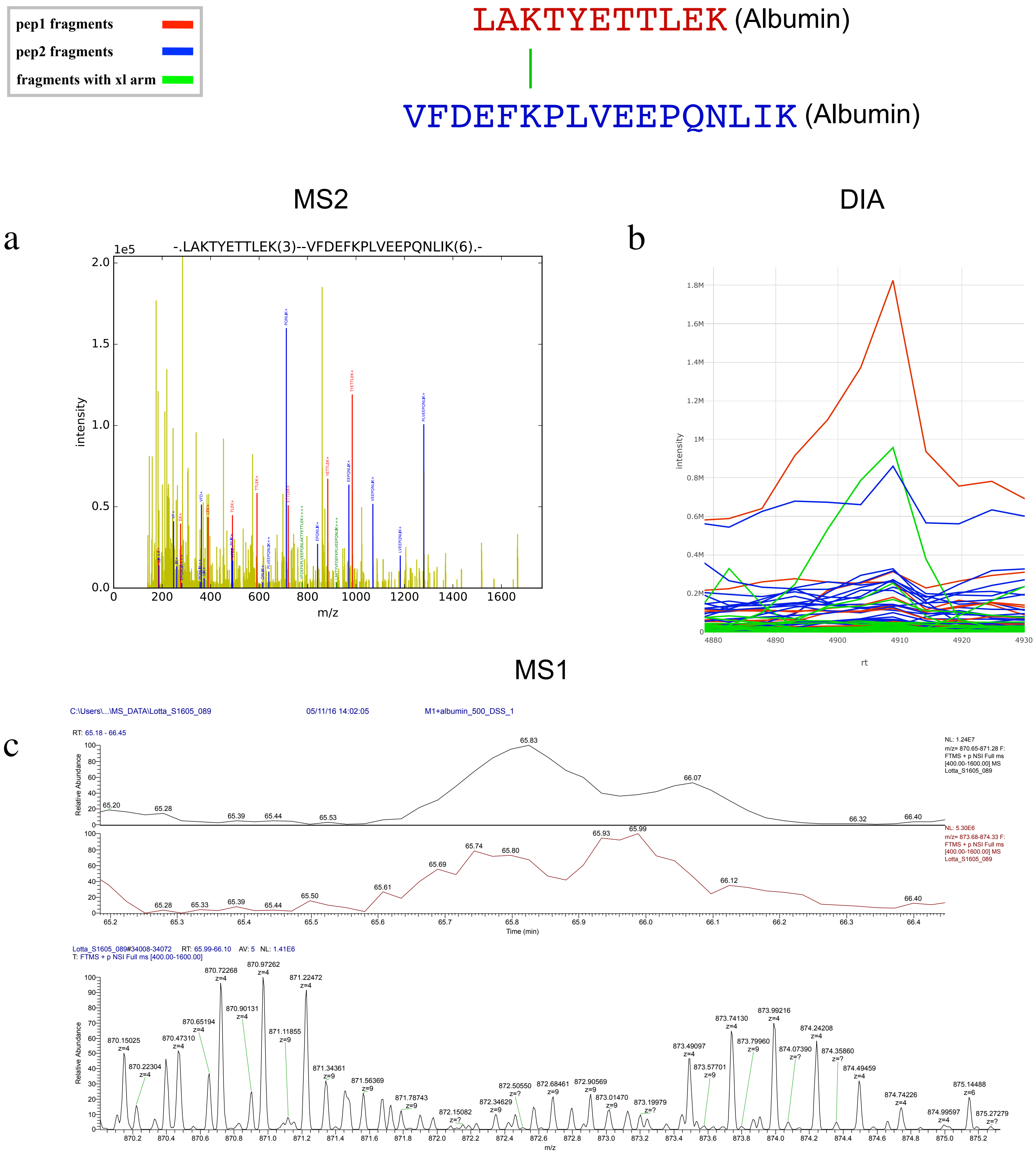

pep1 fragments
pep2 fragments
fragments with xl arm
LAKTYETTLEK (Albumin)
VFDEFKPLVEEPQNLIK (Albumin)
MS2
DIA
a
-.LAKTYETTLEK(3)--VFDEFKPLVEEPQNLIK(6).-
intensity
m/z
b
intensity
rt
MS1
c
C:\Users\...\MS_DATA\Lotta_S1605_089
05/11/16 14:02:05
M1+albumin_500_DSS_1
RT: 65.18 - 66.45
NL: 1.24E7
m/z= 870.65-871.28 F: FTMS + p NSI Full ms [400.00-1600.00] MS Lotta_S1605_089
NL: 5.30E6
m/z= 873.68-874.33 F: FTMS + p NSI Full ms [400.00-1600.00] MS Lotta_S1605_089
Relative Abundance
Time (min)
Lotta_S1605_089#34008-34072 RT: 65.99-66.10 AV: 5 NL: 1.41E6
T: FTMS + p NSI Full ms [400.00-1600.00]
Relative Abundance
m/z

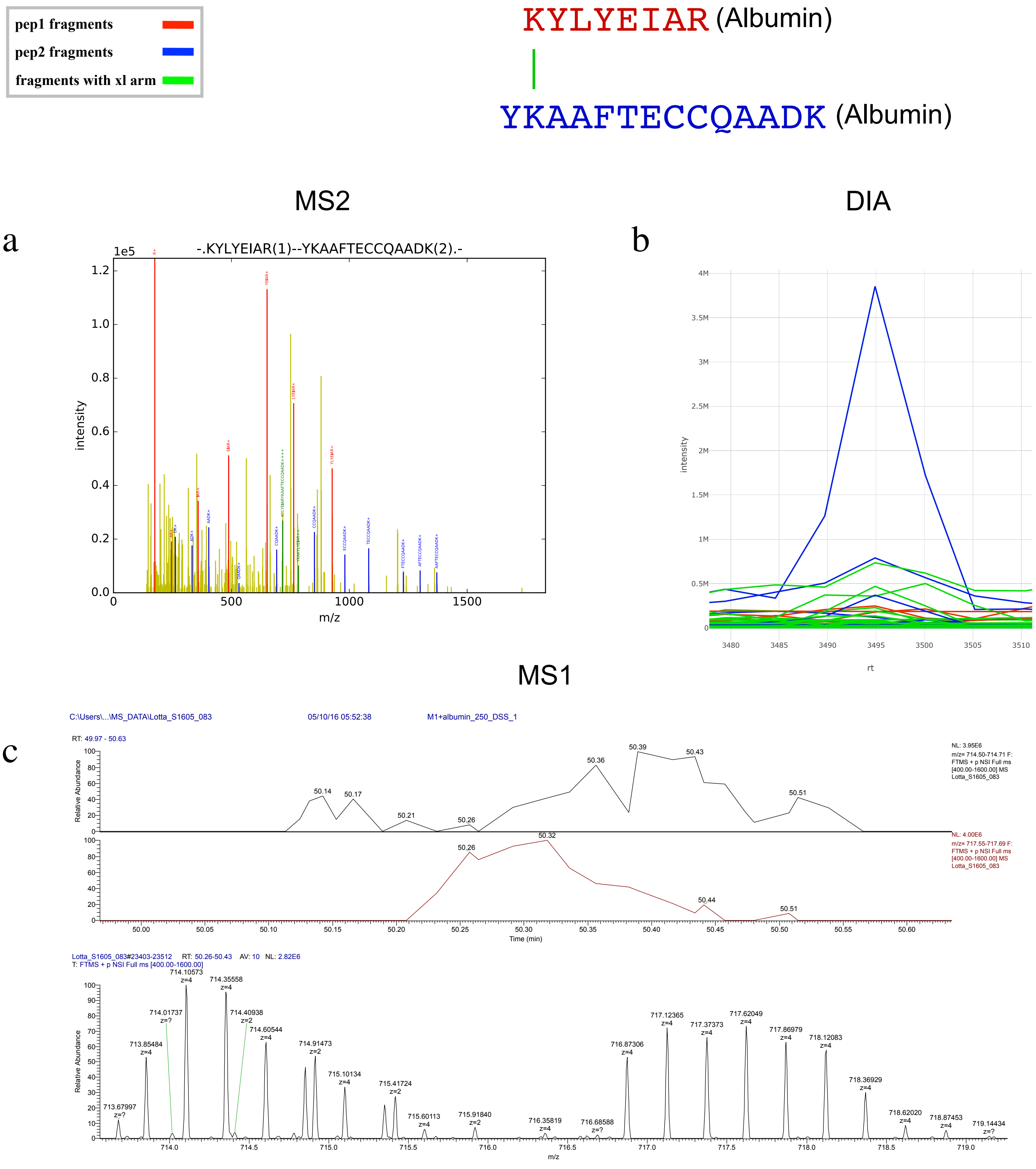

pep1 fragments
pep2 fragments
fragments with xl arm
KYLYEIAR (Albumin)
YKAAFTECCQAADK (Albumin)
MS2
DIA
a
b
-.KYLYEIAR(1)--YKAAFTECCQAADK(2).-
intensity
m/z
rt
MS1
c
C:\Users\...\MS_DATA\Lotta_S1605_083
05/10/16 05:52:38
M1+albumin_250_DSS_1
RT: 49.97 - 50.63
NL: 3.95E6
m/z= 714.50-714.71 F: FTMS + p NSI Full ms [400.00-1600.00] MS Lotta_S1605_083
NL: 4.00E6
m/z= 717.55-717.69 F: FTMS + p NSI Full ms [400.00-1600.00] MS Lotta_S1605_083
Relative Abundance
Time (min)
Lotta_S1605_083#23403-23512 RT: 50.26-50.43 AV: 10 NL: 2.82E6
T: FTMS + p NSI Full ms [400.00-1600.00]

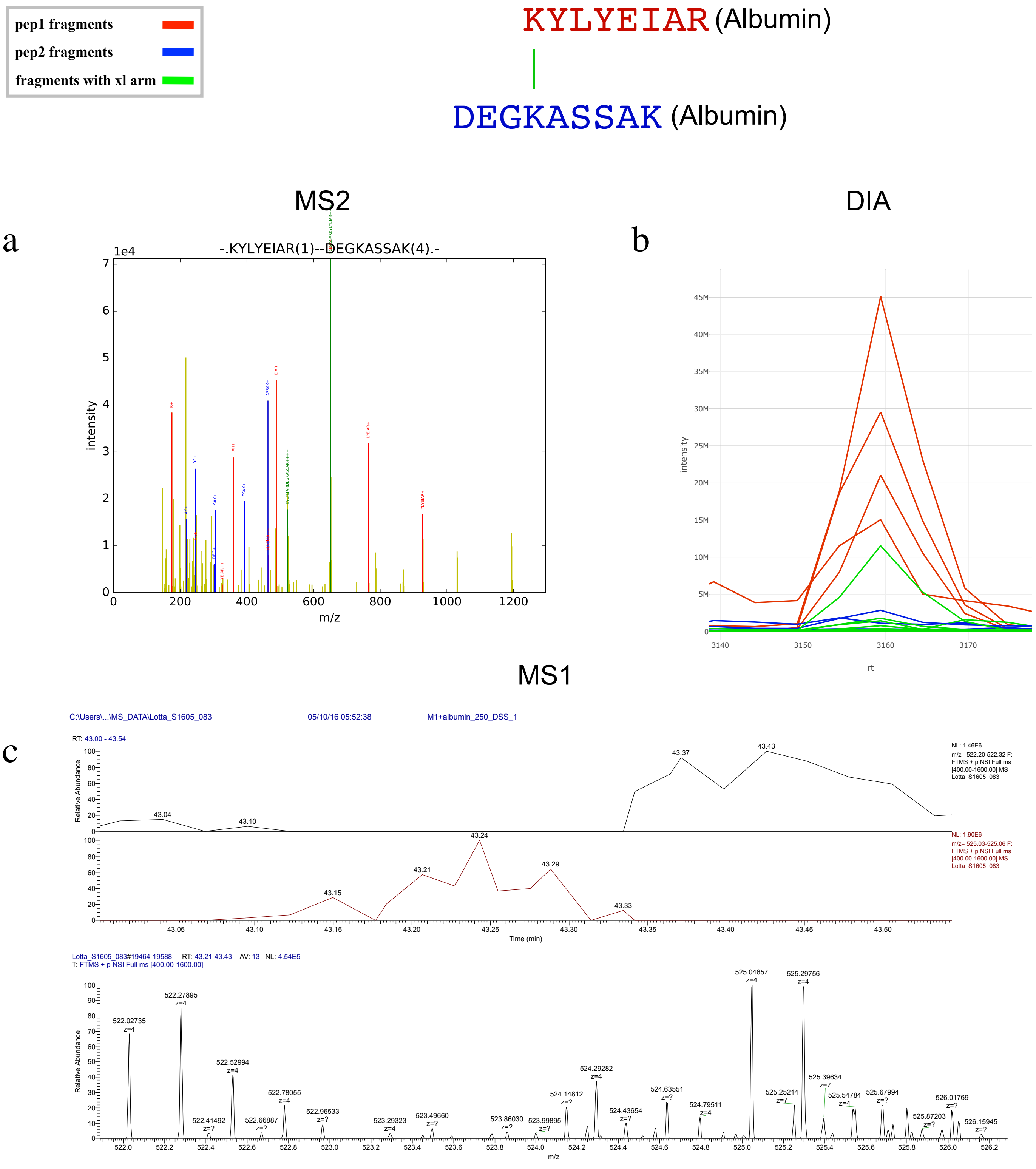
pep1 fragments
pep2 fragments
fragments with xl arm
KYLYEIAR (Albumin)
DEGKASSAK (Albumin)
MS2
DIA
a
b
-.KYLYEIAR(1)--DEGKASSAK(4).-
intensity
m/z
rt
MS1
c
C:\Users\...\MS_DATA\Lotta_S1605_083
05/10/16 05:52:38
M1+albumin_250_DSS_1
RT: 43.00 - 43.54
NL: 1.46E6
m/z= 522.20-522.32 F: FTMS + p NSI Full ms [400.00-1600.00] MS Lotta_S1605_083
NL: 1.90E6
m/z= 525.03-525.06 F: FTMS + p NSI Full ms [400.00-1600.00] MS Lotta_S1605_083
Relative Abundance
Time (min)
Lotta_S1605_083#19464-19588 RT: 43.21-43.43 AV: 13 NL: 4.54E5
T: FTMS + p NSI Full ms [400.00-1600.00]
m/z

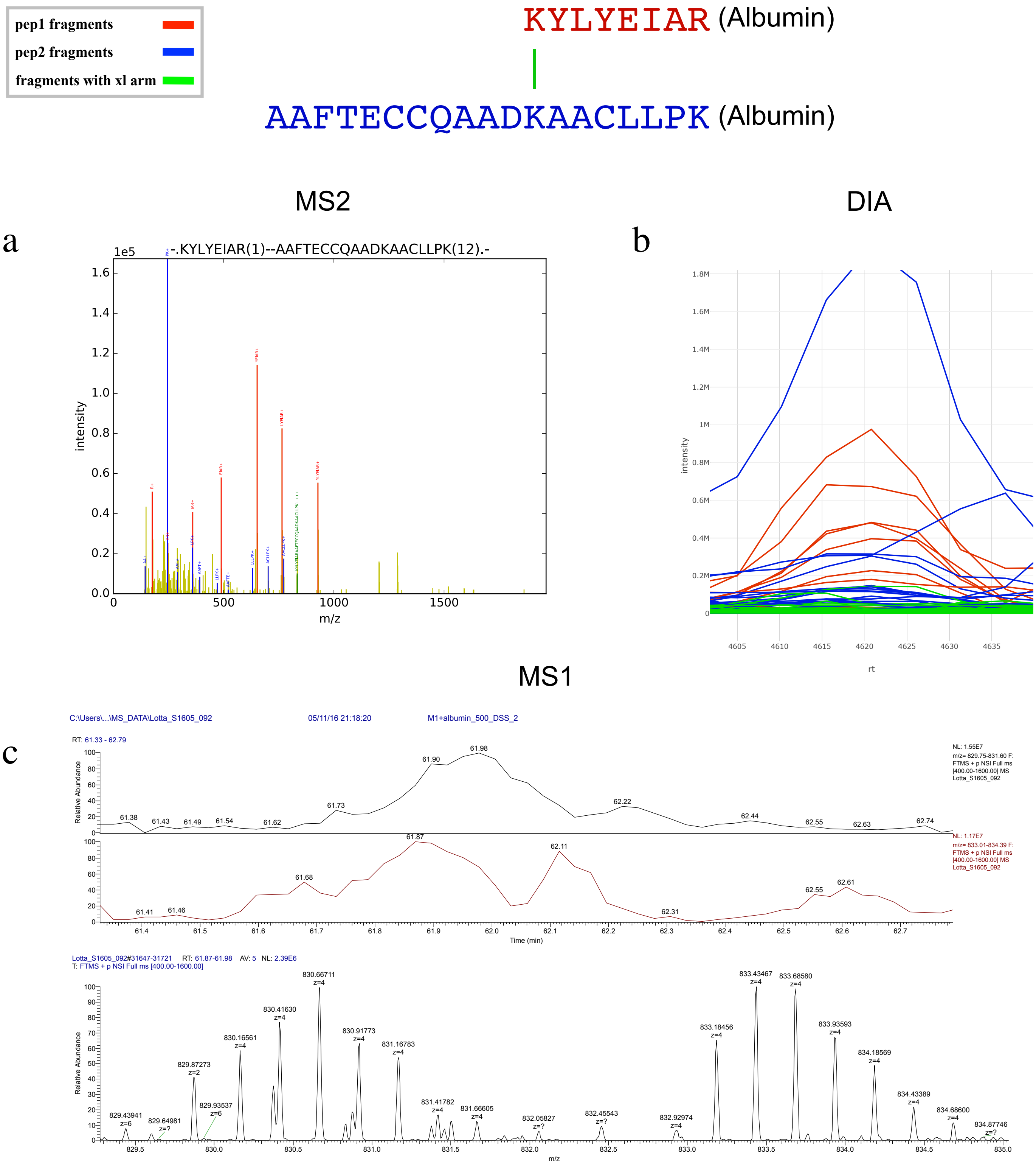
pep1 fragments
pep2 fragments
fragments with xl arm
KYLYEIAR (Albumin)
AAFTECCQAADKAACLLPK (Albumin)
MS2
DIA
a
-.KYLYEIAR(1)--AAFTECCQAADKAACLLPK(12).-
intensity
m/z
b
intensity
rt
MS1
c
C:\Users\...\MS_DATA\Lotta_S1605_092
05/11/16 21:18:20
M1+albumin_500_DSS_2
RT: 61.33 - 62.79
Relative Abundance
NL: 1.55E7
m/z= 829.75-831.60 F: FTMS + p NSI Full ms [400.00-1600.00] MS Lotta_S1605_092
NL: 1.17E7
m/z= 833.01-834.39 F: FTMS + p NSI Full ms [400.00-1600.00] MS Lotta_S1605_092
Time (min)
Lotta_S1605_092#31647-31721 RT: 61.87-61.98 AV: 5 NL: 2.39E6
T: FTMS + p NSI Full ms [400.00-1600.00]
Relative Abundance
m/z

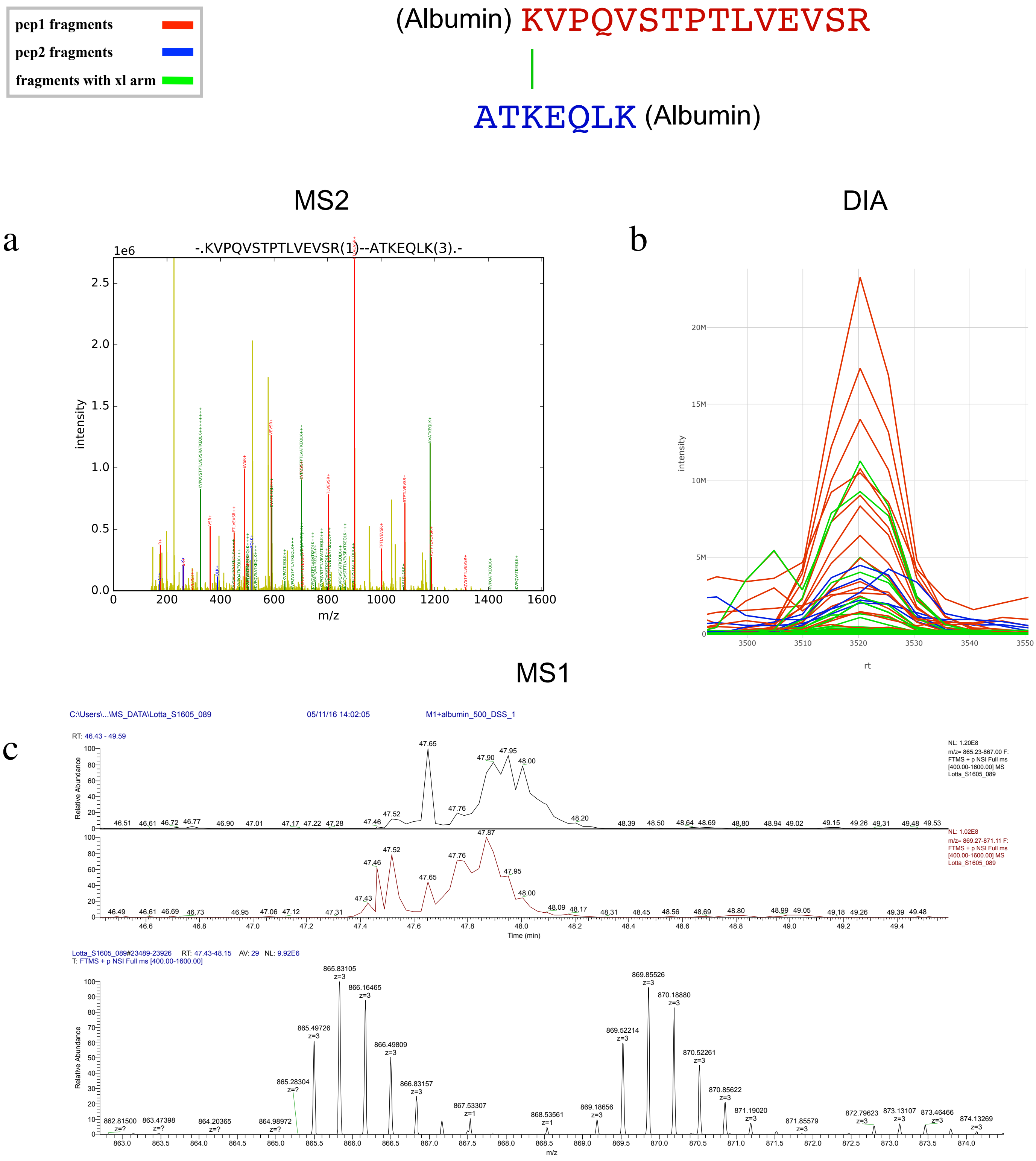

pep1 fragments
pep2 fragments
fragments with xl arm
(Albumin) KVPQVSTPTLVEVSR
ATKEQLK (Albumin)
MS2
DIA
a
-.KVPQVSTPTLVEVSR(1)--ATKEQLK(3).-
1e6
intensity
m/z
b
intensity
rt
MS1
c
C:\Users\...\MS_DATA\Lotta_S1605_089
05/11/16 14:02:05
M1+albumin_500_DSS_1
RT: 46.43 - 49.59
NL: 1.20E8
m/z= 865.23-867.00 F: FTMS + p NSI Full ms [400.00-1600.00] MS Lotta_S1605_089
NL: 1.02E8
m/z= 869.27-871.11 F: FTMS + p NSI Full ms [400.00-1600.00] MS Lotta_S1605_089
Relative Abundance
Time (min)
Lotta_S1605_089#23489-23926 RT: 47.43-48.15 AV: 29 NL: 9.92E6
T: FTMS + p NSI Full ms [400.00-1600.00]
Relative Abundance
m/z

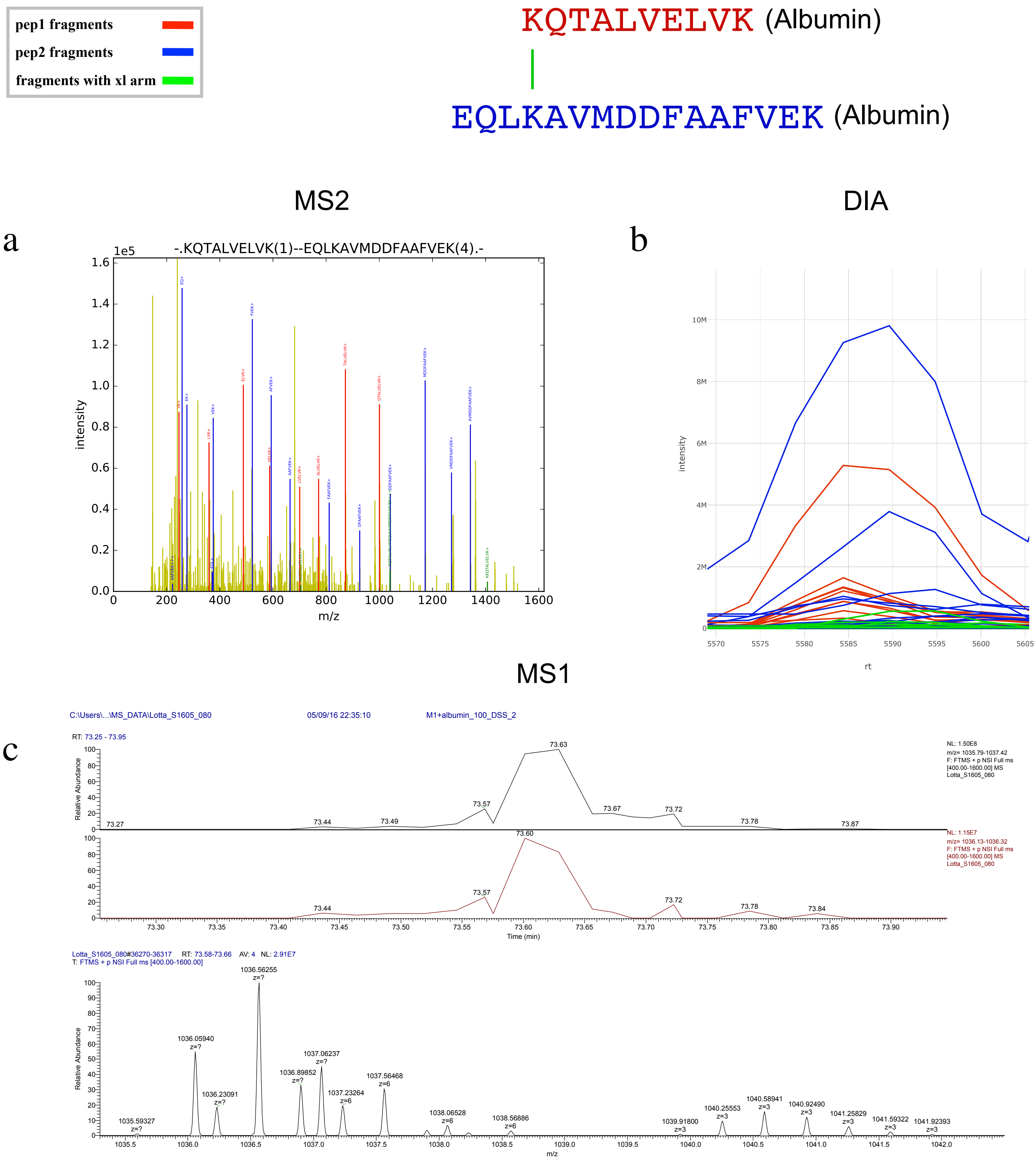

pep1 fragments
pep2 fragments
fragments with xl arm
KQTALVELVK (Albumin)
EQLKAVMDDFAAFVEK (Albumin)
MS2
DIA
a
-.KQTALVELVK(1)--EQLKAVMDDFAAFVEK(4).-
intensity
m/z
b
intensity
rt
MS1
c
C:\Users\...\MS_DATA\Lotta_S1605_080
05/09/16 22:35:10
M1+albumin_100_DSS_2
RT: 73.25 - 73.95
NL: 1.50E8
m/z= 1035.79-1037.42
F: FTMS + p NSI Full ms [400.00-1600.00] MS
Lotta_S1605_080
NL: 1.15E7
m/z= 1036.13-1036.32
F: FTMS + p NSI Full ms [400.00-1600.00] MS
Lotta_S1605_080
Relative Abundance
Time (min)
Lotta_S1605_080#36270-36317 RT: 73.58-73.66 AV: 4 NL: 2.91E7
T: FTMS + p NSI Full ms [400.00-1600.00]
Relative Abundance
m/z

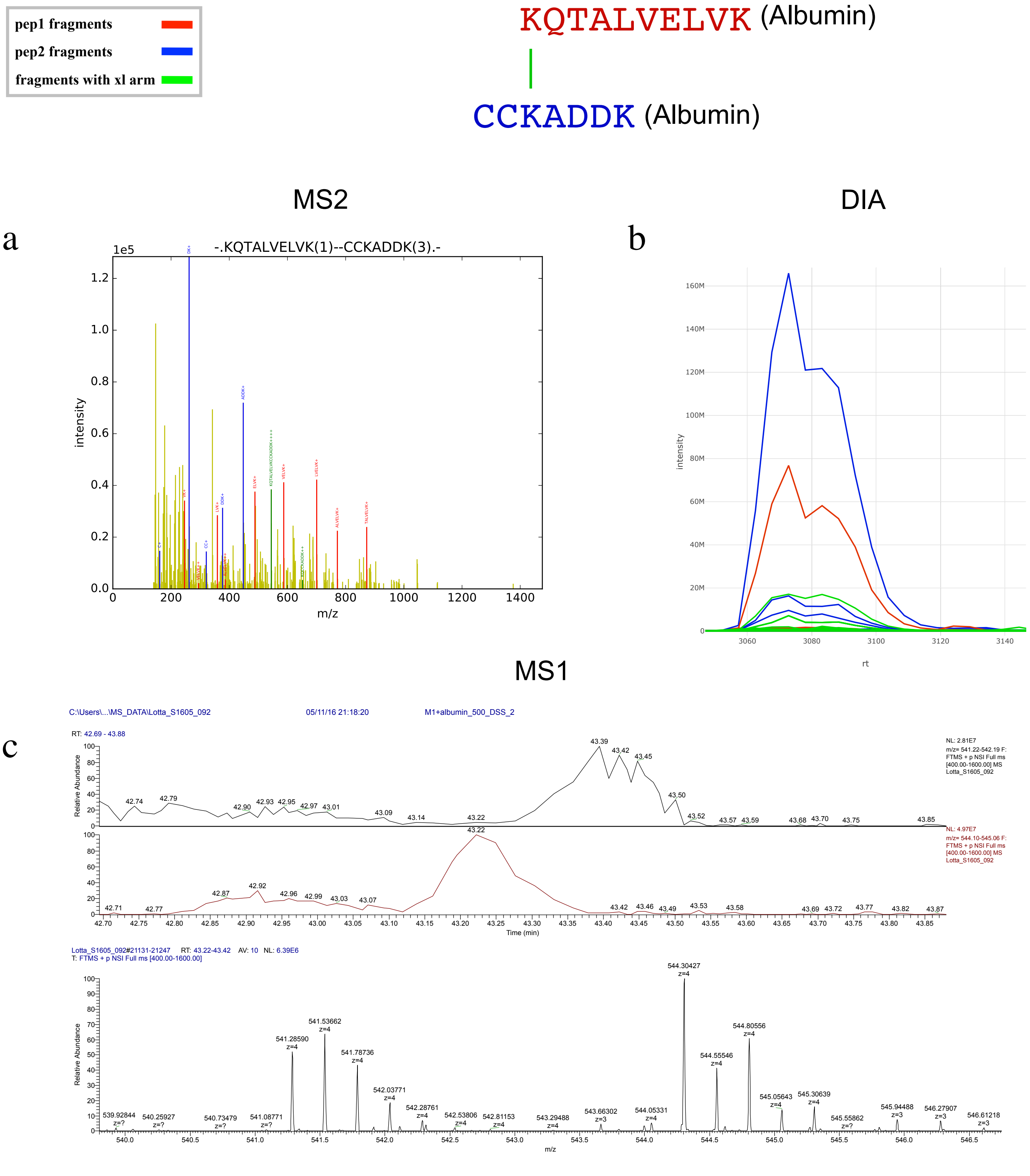

pep1 fragments
pep2 fragments
fragments with xl arm
KQTALVELVK (Albumin)
CCKADDK (Albumin)
MS2
DIA
a
-.KQTALVELVK(1)--CCKADDK(3).-
1e5
intensity
m/z
b
intensity
rt
MS1
c
C:\Users\...\MS_DATA\Lotta_S1605_092
05/11/16 21:18:20
M1+albumin_500_DSS_2
RT: 42.69 - 43.88
Relative Abundance
Time (min)
Lotta_S1605_092#21131-21247 RT: 43.22-43.42 AV: 10 NL: 6.39E6
T: FTMS + p NSI Full ms [400.00-1600.00]
Relative Abundance
m/z

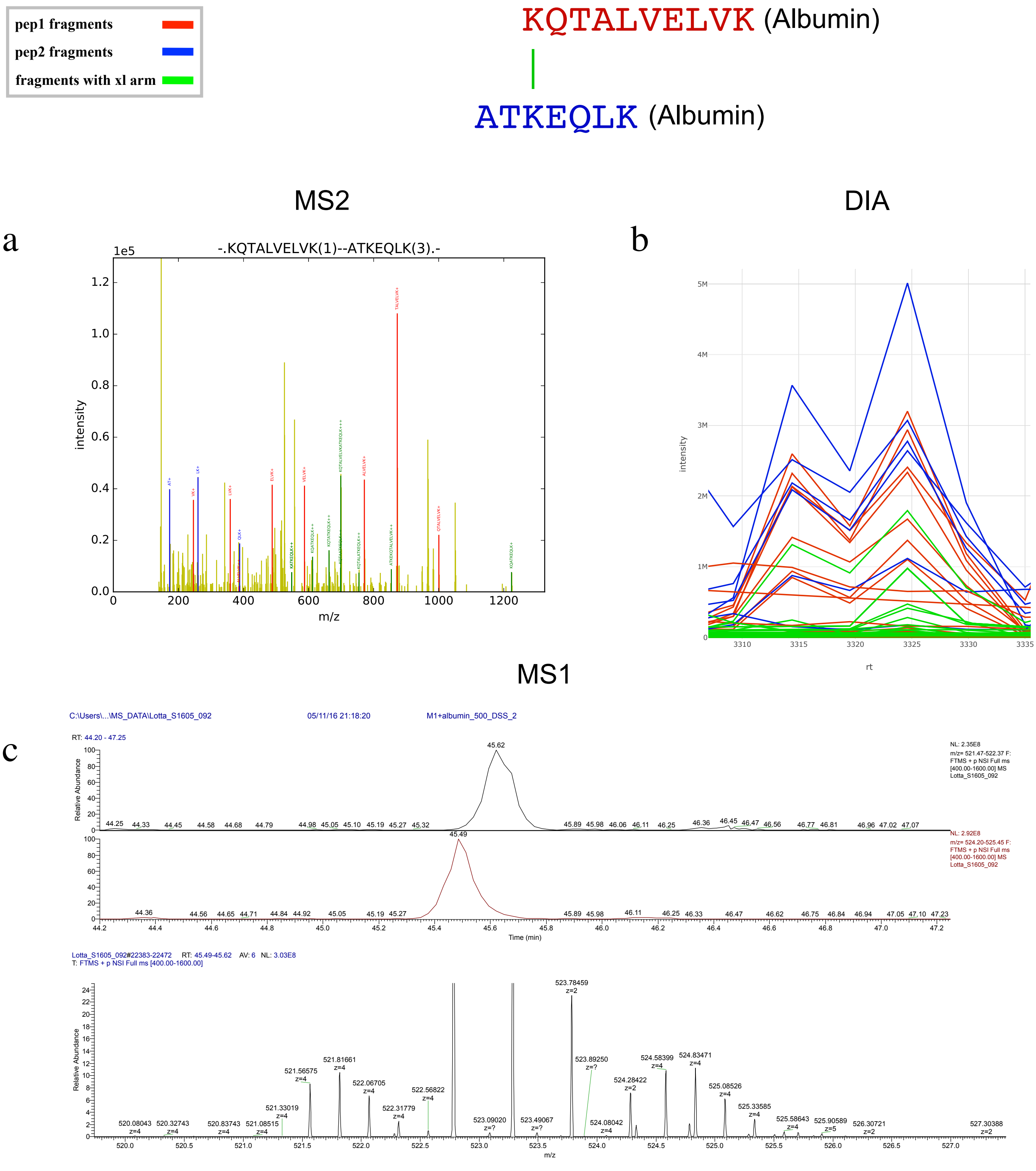

pep1 fragments
pep2 fragments
fragments with xl arm
KQTALVELVK (Albumin)
ATKEQLK (Albumin)
MS2
DIA
a
b
-.KQTALVELVK(1)--ATKEQLK(3).-
1e5
intensity
m/z
rt
MS1
c
C:\Users\...\MS_DATA\Lotta_S1605_092
05/11/16 21:18:20
M1+albumin_500_DSS_2
RT: 44.20 - 47.25
Relative Abundance
Time (min)
NL: 2.35E8
m/z= 521.47-522.37 F: FTMS + p NSI Full ms [400.00-1600.00] MS Lotta_S1605_092
NL: 2.92E8
m/z= 524.20-525.45 F: FTMS + p NSI Full ms [400.00-1600.00] MS Lotta_S1605_092
Lotta_S1605_092#22383-22472 RT: 45.49-45.62 AV: 6 NL: 3.03E8
T: FTMS + p NSI Full ms [400.00-1600.00]

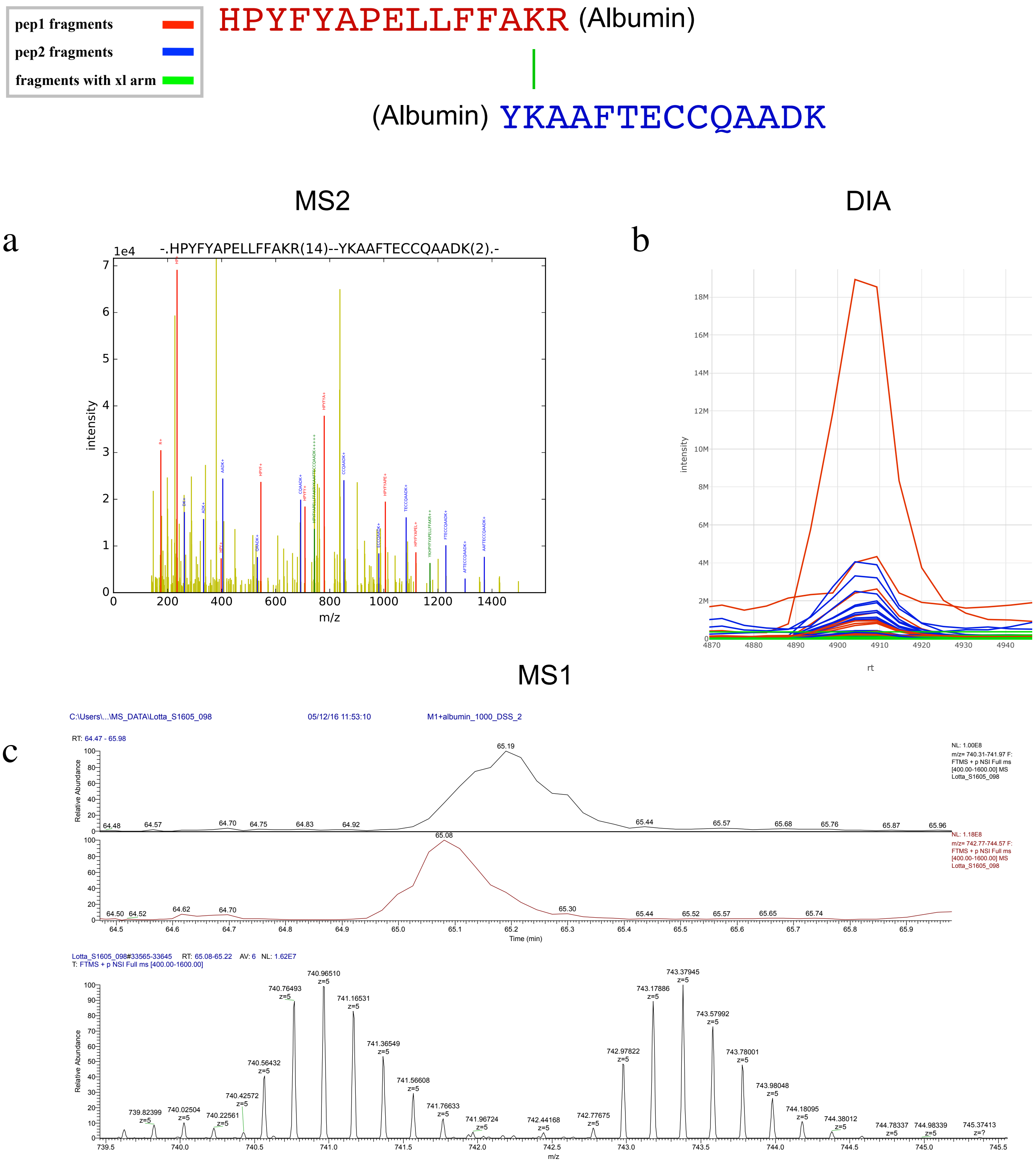
pep1 fragments
pep2 fragments
fragments with xl arm
HPYFYAPELLFFAKR (Albumin)
(Albumin) YKAAFTECCQAADK
MS2
DIA
a
-.HPYFYAPELLFFAKR(14)--YKAAFTECCQAADK(2).-
1e4
intensity
m/z
b
intensity
rt
MS1
c
C:\Users\...\MS_DATA\Lotta_S1605_098
05/12/16 11:53:10
M1+albumin_1000_DSS_2
RT: 64.47 - 65.98
Relative Abundance
NL: 1.00E8
m/z= 740.31-741.97 F: FTMS + p NSI Full ms [400.00-1600.00] MS Lotta_S1605_098
NL: 1.18E8
m/z= 742.77-744.57 F: FTMS + p NSI Full ms [400.00-1600.00] MS Lotta_S1605_098
Time (min)
Lotta_S1605_098#33565-33645 RT: 65.08-65.22 AV: 6 NL: 1.62E7
T: FTMS + p NSI Full ms [400.00-1600.00]
Relative Abundance
m/z

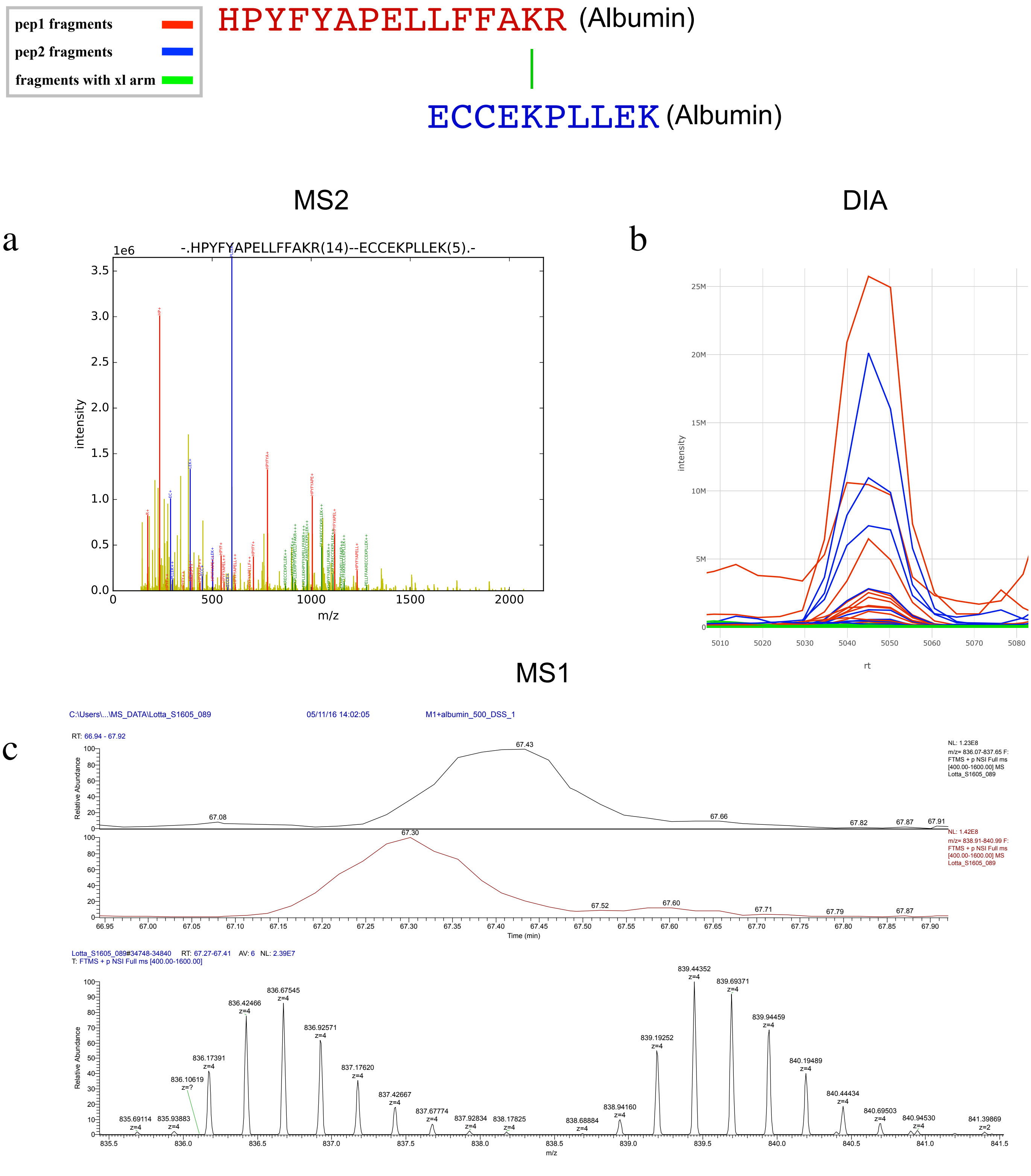

pep1 fragments
pep2 fragments
fragments with xl arm
HPYFYAPELLFFAKR (Albumin)
ECCEKPLLEK (Albumin)
MS2
DIA
a
-.HPYFYAPELLFFAKR(14)--ECCEKPLLEK(5).-
intensity
m/z
b
intensity
rt
MS1
c
C:\Users\...\MS_DATA\Lotta_S1605_089
05/11/16 14:02:05
M1+albumin_500_DSS_1
RT: 66.94 - 67.92
NL: 1.23E8
m/z= 836.07-837.65 F: FTMS + p NSI Full ms [400.00-1600.00] MS Lotta_S1605_089
NL: 1.42E8
m/z= 838.91-840.99 F: FTMS + p NSI Full ms [400.00-1600.00] MS Lotta_S1605_089
Relative Abundance
Time (min)
Lotta_S1605_089#34748-34840 RT: 67.27-67.41 AV: 6 NL: 2.39E7
T: FTMS + p NSI Full ms [400.00-1600.00]
Relative Abundance
m/z

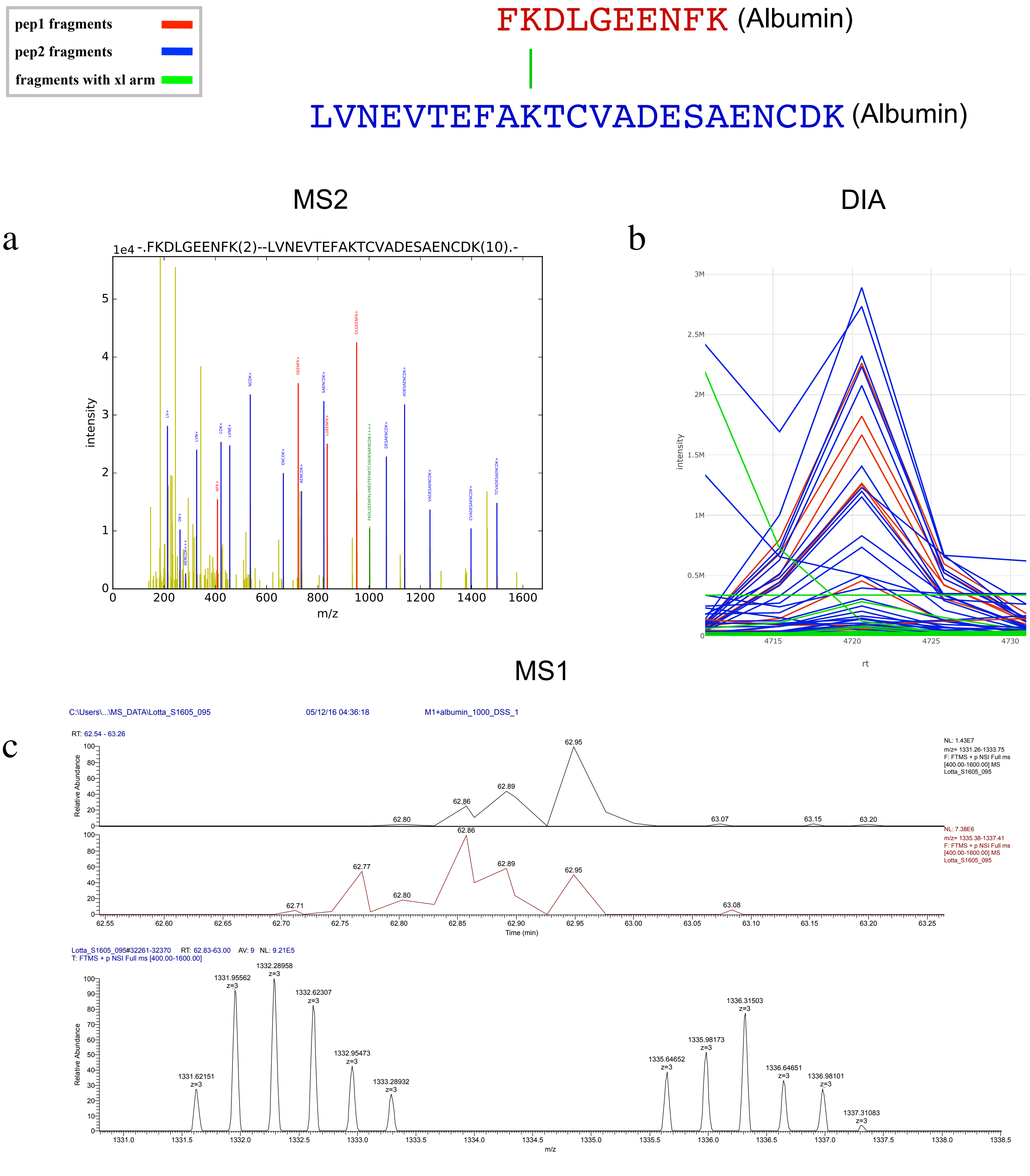

pep1 fragments
pep2 fragments
fragments with xl arm
FKDLGEENFK (Albumin)
LVNEVTEFAKTCVADESAENCDK (Albumin)
MS2
DIA
a
-.FKDLGEENFK(2)--LVNEVTEFAKTCVADESAENCDK(10).-
intensity
m/z
b
intensity
rt
MS1
c
C:\Users\...\MS_DATA\Lotta_S1605_095
05/12/16 04:36:18
M1+albumin_1000_DSS_1
RT: 62.54 - 63.26
NL: 1.43E7
m/z= 1331.26-1333.75
F: FTMS + p NSI Full ms [400.00-1600.00] MS
Lotta_S1605_095
NL: 7.38E6
m/z= 1335.38-1337.41
F: FTMS + p NSI Full ms [400.00-1600.00] MS
Lotta_S1605_095
Relative Abundance
Time (min)
Lotta_S1605_095#32261-32370 RT: 62.83-63.00 AV: 9 NL: 9.21E5
T: FTMS + p NSI Full ms [400.00-1600.00]
Relative Abundance
m/z

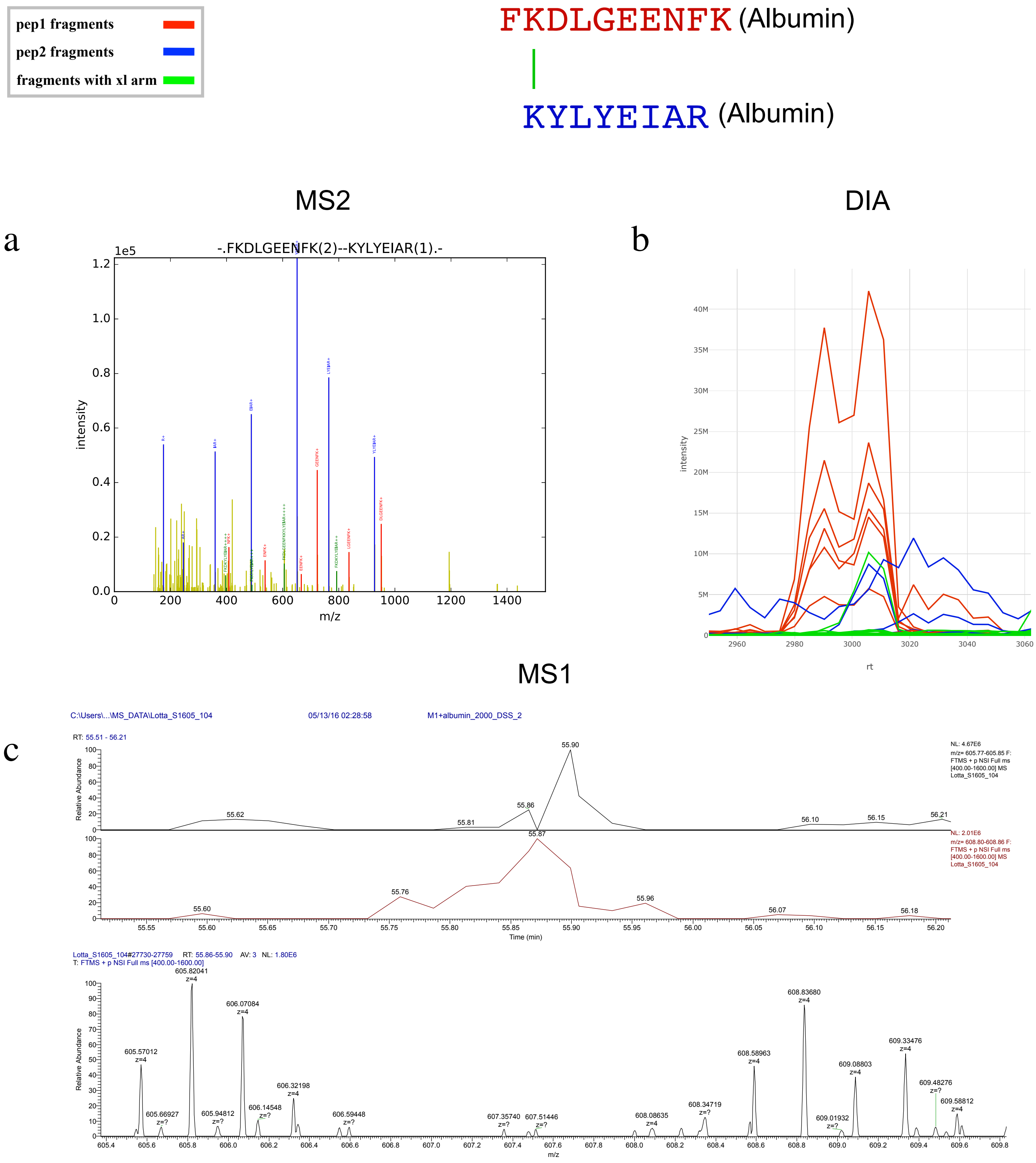

pep1 fragments
pep2 fragments
fragments with xl arm
FKDLGEENFK (Albumin)
KYLYEIAR (Albumin)
MS2
DIA
a
-.FKDLGEENFK(2)--KYLYEIAR(1).-
1e5
intensity
m/z
b
intensity
rt
MS1
c
C:\Users\...\MS_DATA\Lotta_S1605_104
05/13/16 02:28:58
M1+albumin_2000_DSS_2
RT: 55.51 - 56.21
Relative Abundance
NL: 4.67E6
m/z= 605.77-605.85 F: FTMS + p NSI Full ms [400.00-1600.00] MS Lotta_S1605_104
NL: 2.01E6
m/z= 608.80-608.86 F: FTMS + p NSI Full ms [400.00-1600.00] MS Lotta_S1605_104
Time (min)
Lotta_S1605_104#27730-27759 RT: 55.86-55.90 AV: 3 NL: 1.80E6
T: FTMS + p NSI Full ms [400.00-1600.00]
m/z

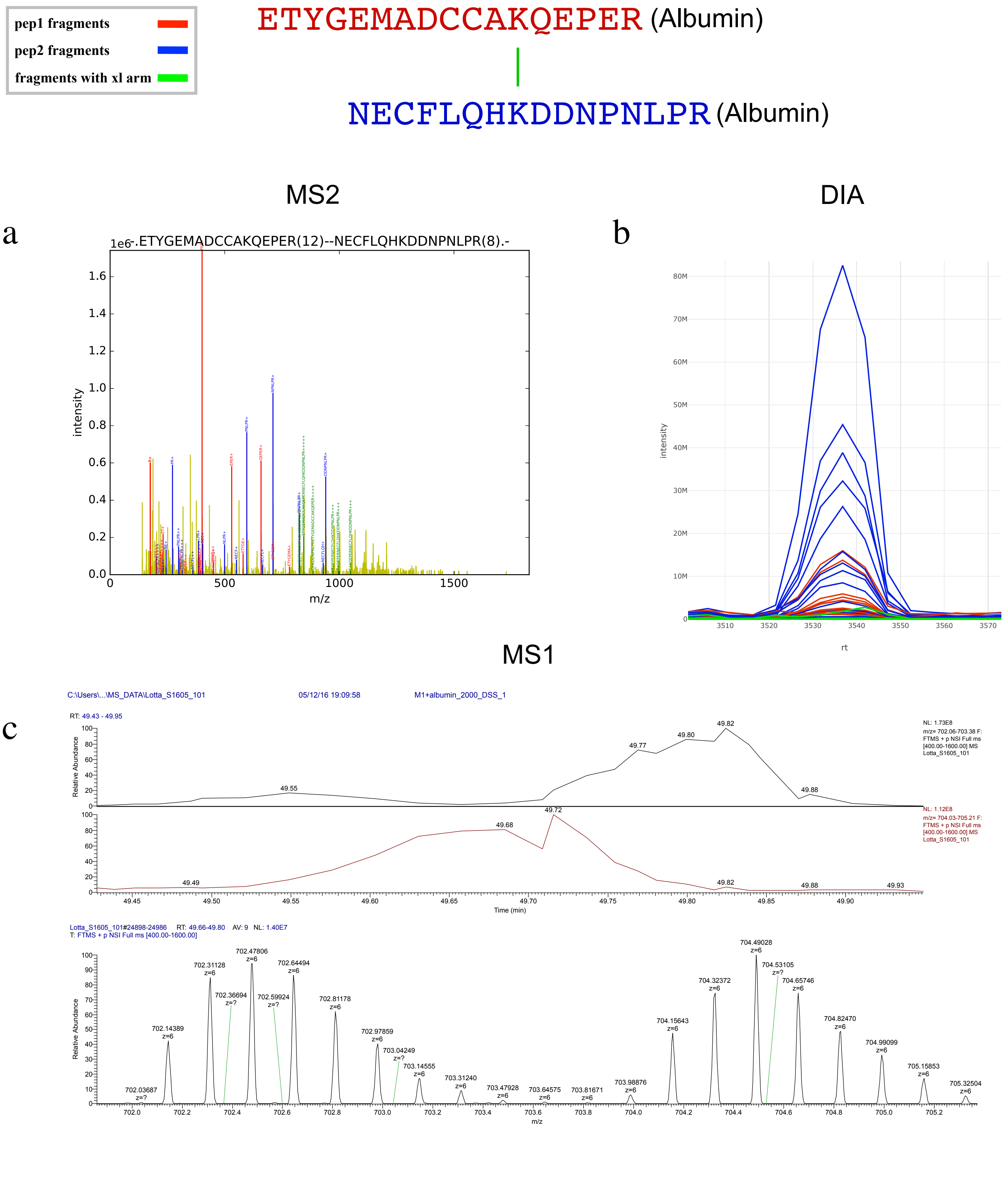

pep1 fragments
pep2 fragments
fragments with xl arm
ETYGEMADCCAKQEPER (Albumin)
NECFLQHKDDNPNLPR (Albumin)
MS2
DIA
a
1e6 -.ETYGEMADCCAKQEPER(12)--NECFLQHKDDNPNLPR(8).-
intensity
m/z
b
intensity
rt
MS1
c
C:\Users\...\MS_DATA\Lotta_S1605_101
05/12/16 19:09:58
M1+albumin_2000_DSS_1
RT: 49.43 - 49.95
NL: 1.73E8
m/z= 702.06-703.38 F: FTMS + p NSI Full ms [400.00-1600.00] MS Lotta_S1605_101
NL: 1.12E8
m/z= 704.03-705.21 F: FTMS + p NSI Full ms [400.00-1600.00] MS Lotta_S1605_101
Relative Abundance
Time (min)
Lotta_S1605_101#24898-24986 RT: 49.66-49.80 AV: 9 NL: 1.40E7
T: FTMS + p NSI Full ms [400.00-1600.00]
Relative Abundance
m/z

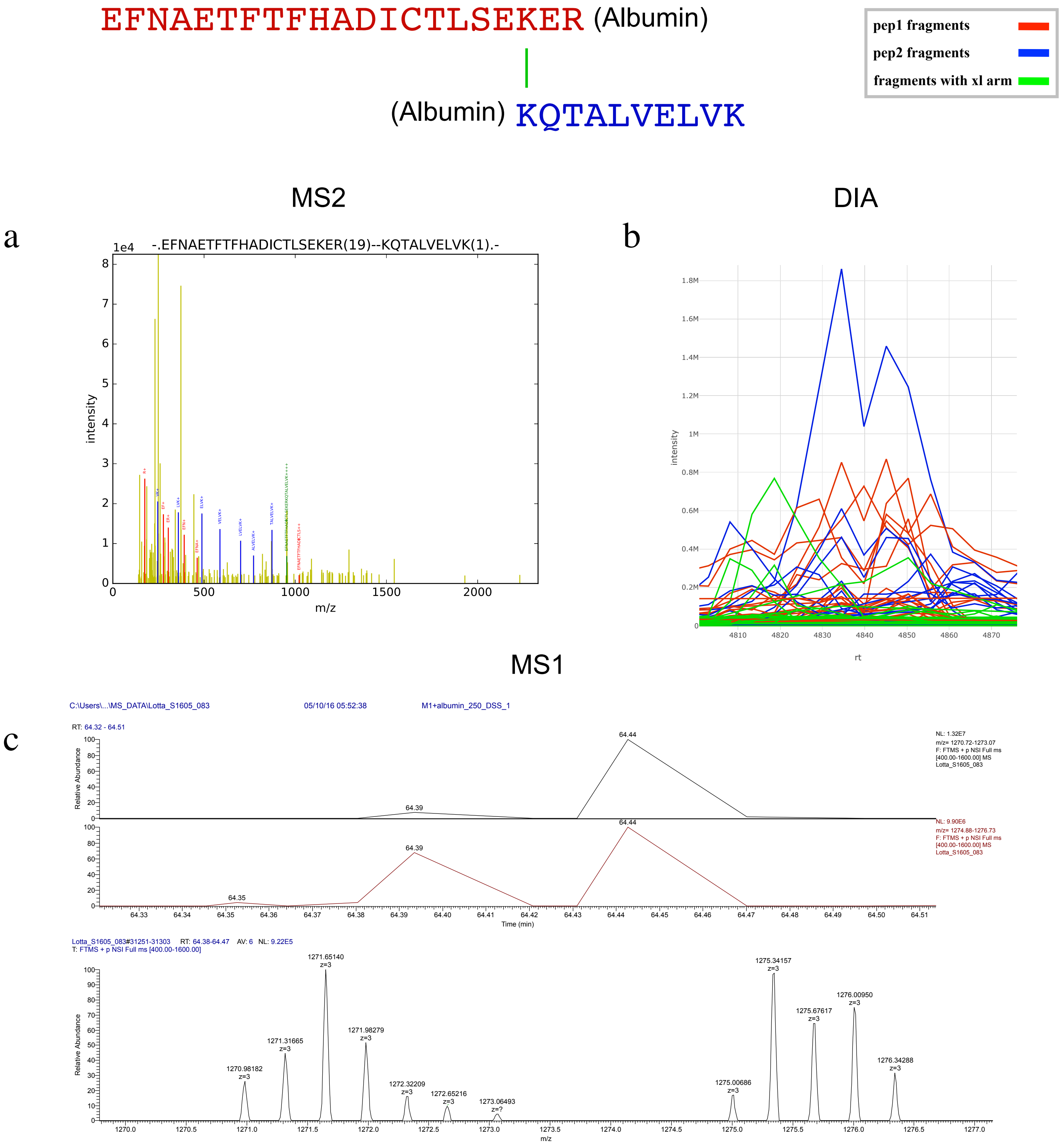
EFNAETFTFHADICTLSEKER (Albumin)
(Albumin) KQTALVELVK
pep1 fragments
pep2 fragments
fragments with xl arm
MS2
a
-.EFNAETFTFHADICTLSEKER(19)--KQTALVELVK(1).-
1e4
intensity
m/z
DIA
b
intensity
rt
MS1
c
C:\Users\...\MS_DATA\Lotta_S1605_083
05/10/16 05:52:38
M1+albumin_250_DSS_1
RT: 64.32 - 64.51
NL: 1.32E7
m/z= 1270.72-1273.07
F: FTMS + p NSI Full ms [400.00-1600.00] MS
Lotta_S1605_083
NL: 9.90E6
m/z= 1274.88-1276.73
F: FTMS + p NSI Full ms [400.00-1600.00] MS
Lotta_S1605_083
Relative Abundance
Time (min)
Lotta_S1605_083#31251-31303 RT: 64.38-64.47 AV: 6 NL: 9.22E5
T: FTMS + p NSI Full ms [400.00-1600.00]
Relative Abundance
m/z

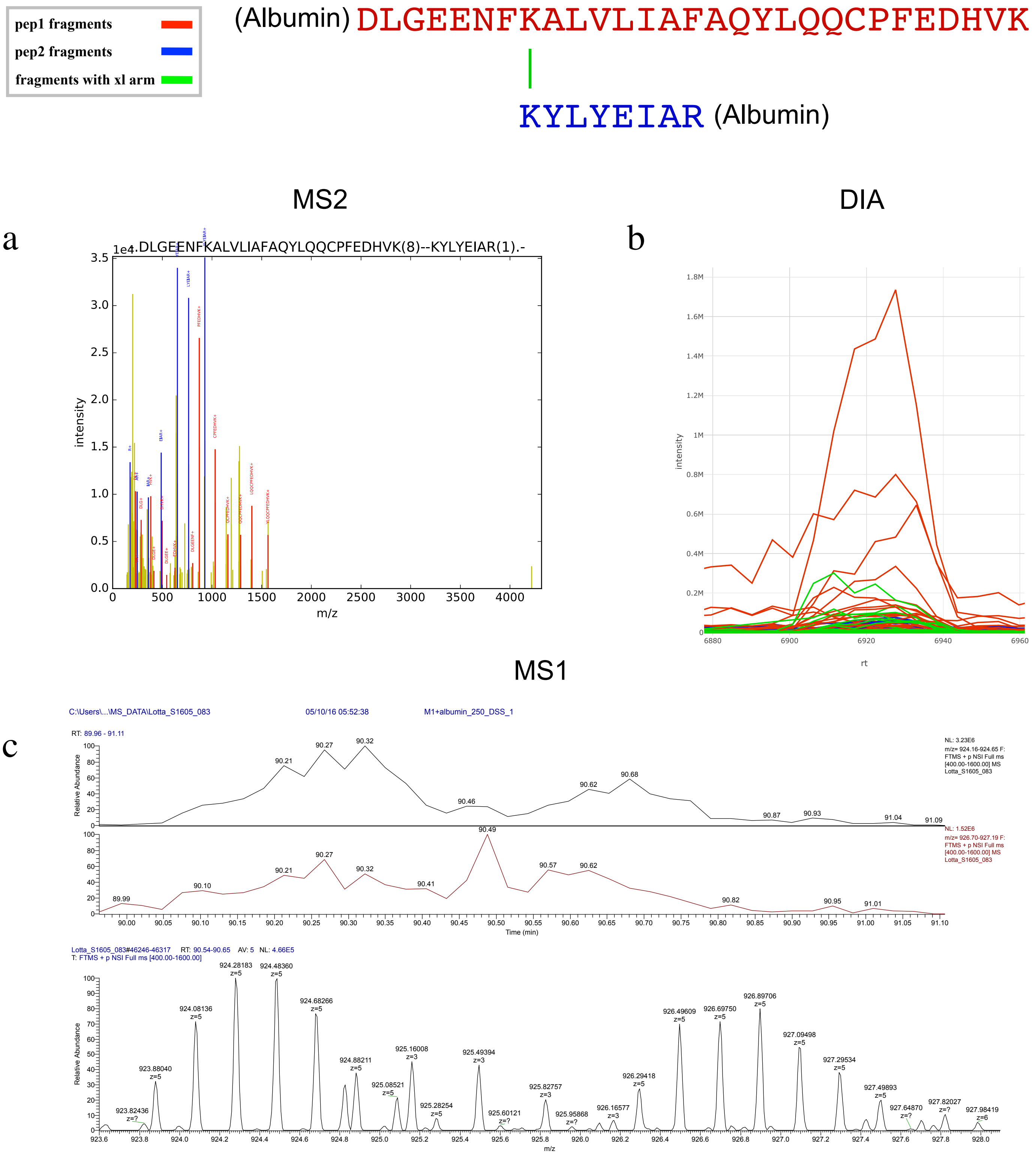
pep1 fragments
pep2 fragments
fragments with xl arm
(Albumin) DLGEENFKALVLIAFAQYLQQCPFEDHVK
KYLYEIAR (Albumin)
MS2
DIA
a
.DLGEENFKALVLIAFAQYLQQCPFEDHVK(8)--KYLYEIAR(1).-
1e4
intensity
m/z
b
intensity
rt
MS1
c
C:\Users\...\MS_DATA\Lotta_S1605_083
05/10/16 05:52:38
M1+albumin_250_DSS_1
RT: 89.96 - 91.11
Relative Abundance
NL: 3.23E6
m/z= 924.16-924.65 F: FTMS + p NSI Full ms [400.00-1600.00] MS Lotta_S1605_083
NL: 1.52E6
m/z= 926.70-927.19 F: FTMS + p NSI Full ms [400.00-1600.00] MS Lotta_S1605_083
Time (min)
Lotta_S1605_083#46246-46317 RT: 90.54-90.65 AV: 5 NL: 4.66E5
T: FTMS + p NSI Full ms [400.00-1600.00]
Relative Abundance
m/z

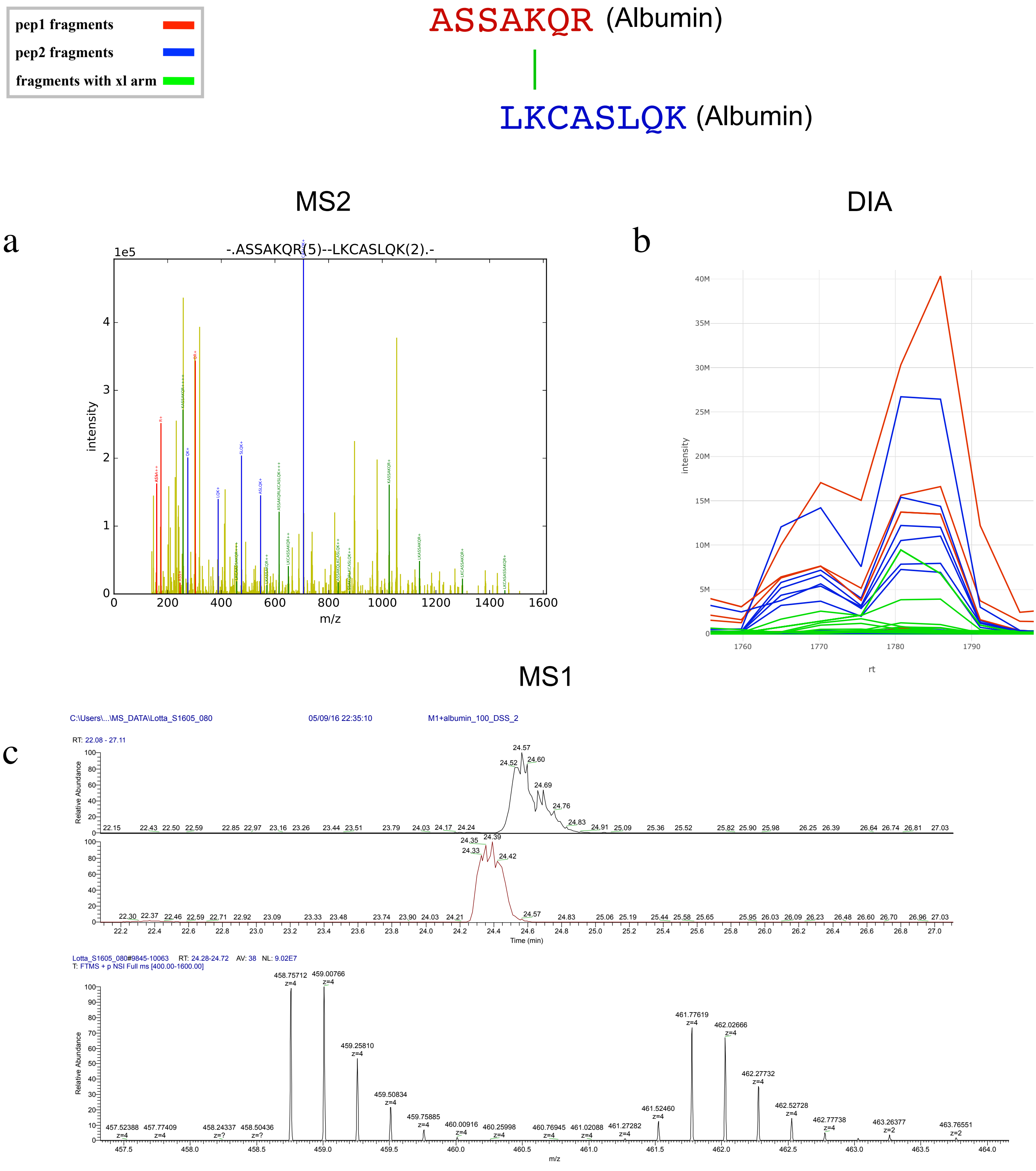
pep1 fragments
pep2 fragments
fragments with xl arm
ASSAKQR (Albumin)
LKCASLQK (Albumin)
MS2
DIA
a
-.ASSAKQR(5)--LKCASLQK(2).-
1e5
intensity
m/z
b
intensity
rt
MS1
c
C:\Users\...\MS_DATA\Lotta_S1605_080
05/09/16 22:35:10
M1+albumin_100_DSS_2
RT: 22.08 - 27.11
Relative Abundance
Time (min)
Lotta_S1605_080#9845-10063 RT: 24.28-24.72 AV: 38 NL: 9.02E7
T: FTMS + p NSI Full ms [400.00-1600.00]
Relative Abundance
m/z

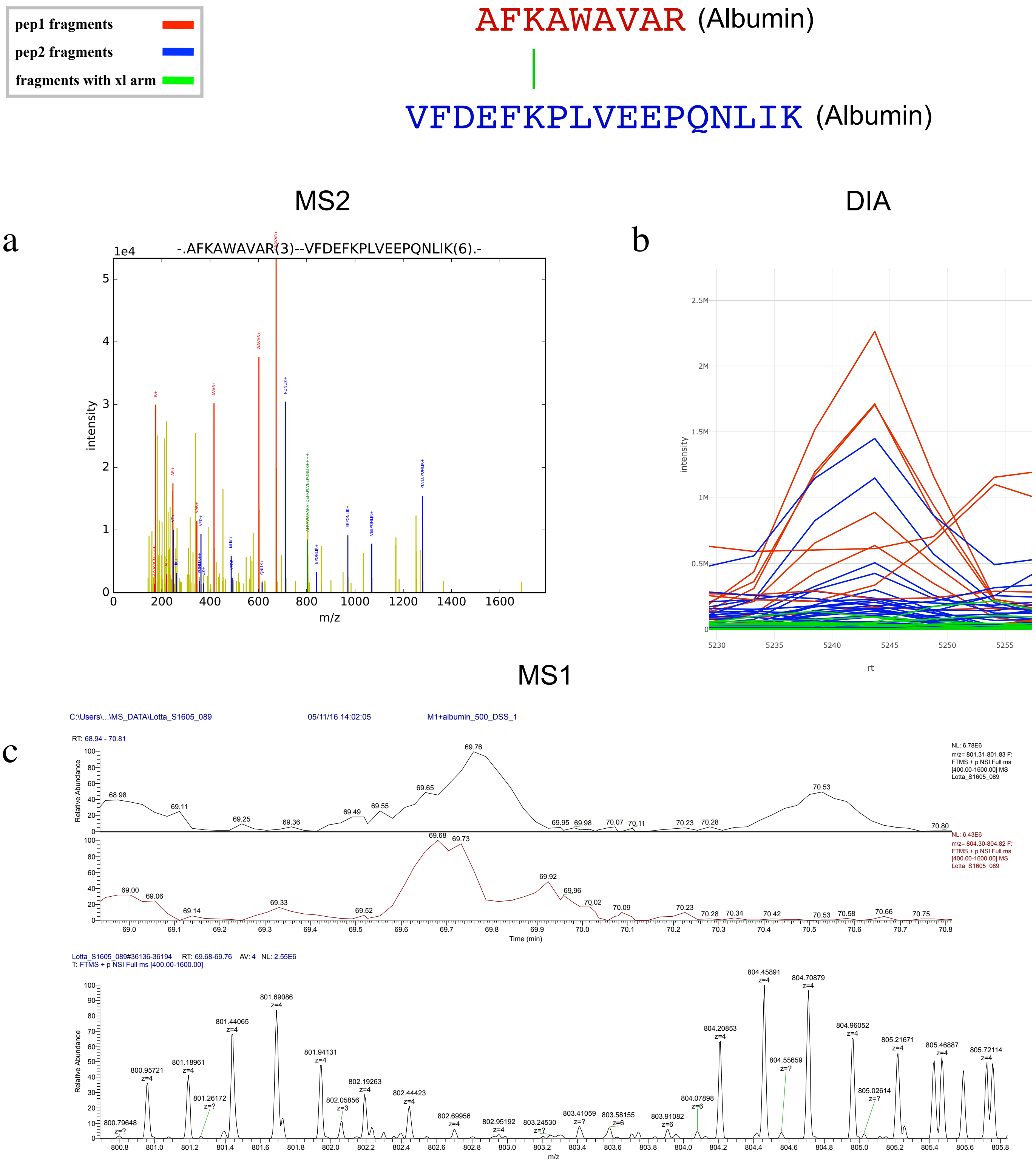

pep1 fragments
pep2 fragments
fragments with xl arm
AFKAWAVAR (Albumin)
VFDEFKPLVEEPQNLIK (Albumin)
MS2
DIA
a
-.AFKAWAVAR(3)--VFDEFKPLVEEPQNLIK(6).-
1e4
intensity
m/z
b
intensity
rt
MS1
c
C:\Users\...\MS_DATA\Lotta_S1605_089
05/11/16 14:02:05
M1+albumin_500_DSS_1
RT: 68.94 - 70.81
Relative Abundance
NL: 6.78E6
m/z= 801.31-801.83 F: FTMS + p NSI Full ms [400.00-1600.00] MS Lotta_S1605_089
NL: 6.43E6
m/z= 804.30-804.82 F: FTMS + p NSI Full ms [400.00-1600.00] MS Lotta_S1605_089
Time (min)
Lotta_S1605_089#36136-36194 RT: 69.68-69.76 AV: 4 NL: 2.55E6
T: FTMS + p NSI Full ms [400.00-1600.00]
Relative Abundance
m/z

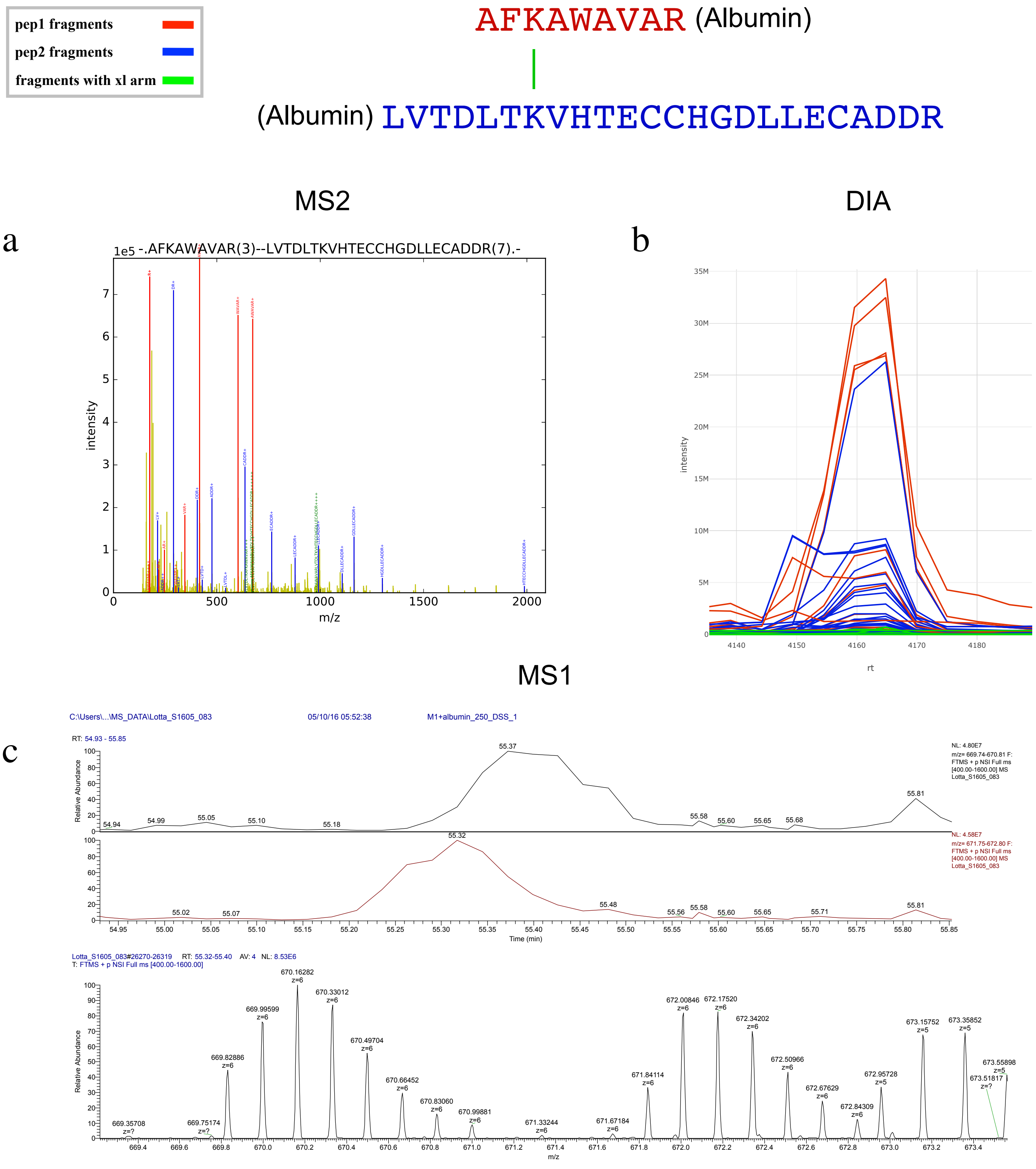

pep1 fragments
pep2 fragments
fragments with xl arm
AFKAWAVAR (Albumin)
(Albumin) LVTDLTKVHTECCHGDLLECADDR
MS2
DIA
a
b
1e5 -.AFKAWAVAR(3)--LVTDLTKVHTECCHGDLLECADDR(7).-
intensity
m/z
rt
MS1
c
C:\Users\...\MS_DATA\Lotta_S1605_083
05/10/16 05:52:38
M1+albumin_250_DSS_1
RT: 54.93 - 55.85
Relative Abundance
NL: 4.80E7
m/z= 669.74-670.81 F: FTMS + p NSI Full ms [400.00-1600.00] MS Lotta_S1605_083
NL: 4.58E7
m/z= 671.75-672.80 F: FTMS + p NSI Full ms [400.00-1600.00] MS Lotta_S1605_083
Time (min)
Lotta_S1605_083#26270-26319 RT: 55.32-55.40 AV: 4 NL: 8.53E6
T: FTMS + p NSI Full ms [400.00-1600.00]

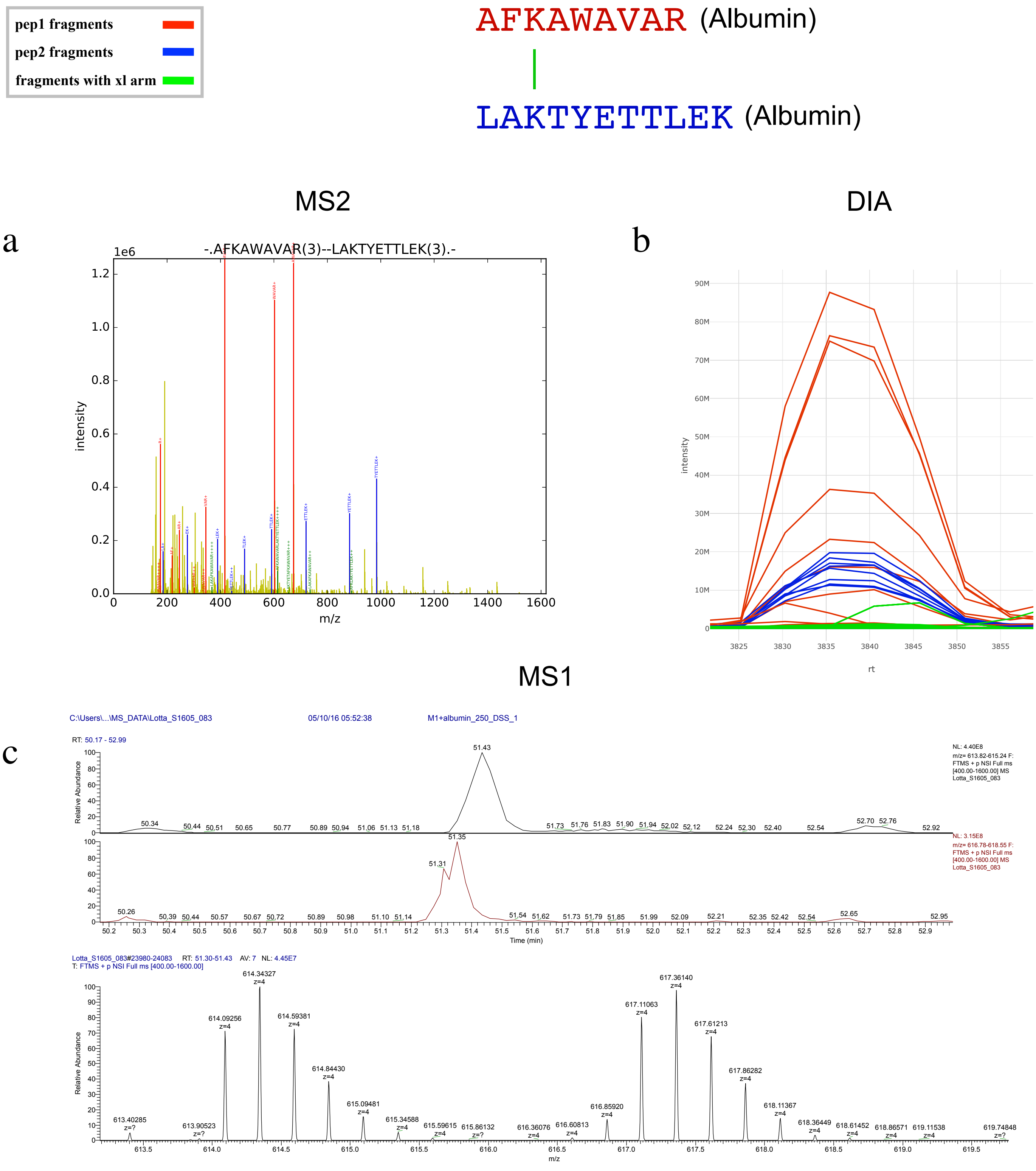
pep1 fragments
pep2 fragments
fragments with xl arm
AFKAWAVAR (Albumin)
LAKTYETTLEK (Albumin)
MS2
DIA
a
-.AFKAWAVAR(3)--LAKTYETTLEK(3).-
1e6
intensity
m/z
b
intensity
rt
MS1
c
C:\Users\...\MS_DATA\Lotta_S1605_083
05/10/16 05:52:38
M1+albumin_250_DSS_1
RT: 50.17 - 52.99
NL: 4.40E8
m/z= 613.82-615.24 F: FTMS + p NSI Full ms [400.00-1600.00] MS Lotta_S1605_083
NL: 3.15E8
m/z= 616.78-618.55 F: FTMS + p NSI Full ms [400.00-1600.00] MS Lotta_S1605_083
Relative Abundance
Time (min)
Lotta_S1605_083#23980-24083 RT: 51.30-51.43 AV: 7 NL: 4.45E7
T: FTMS + p NSI Full ms [400.00-1600.00]
Relative Abundance
m/z

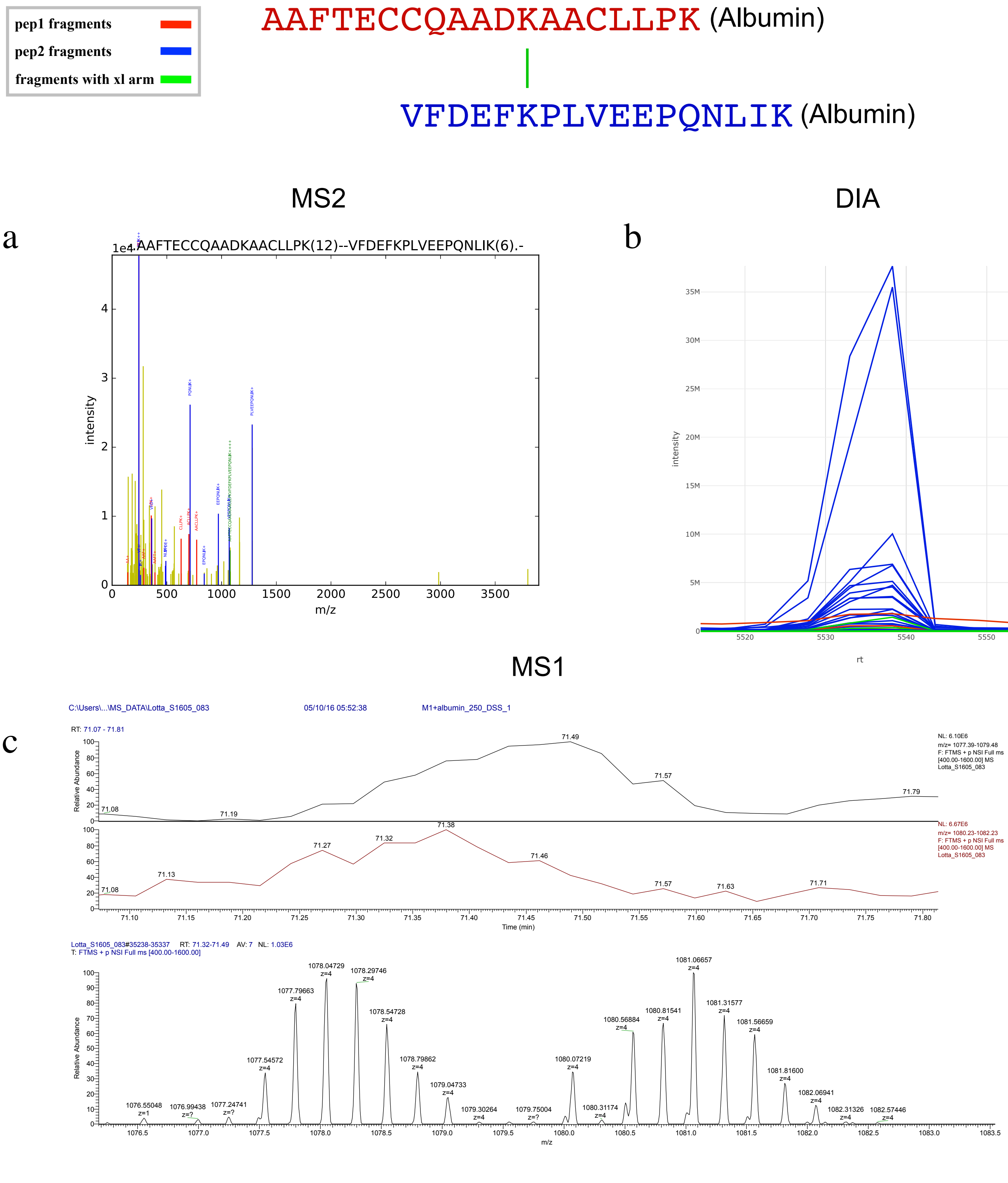

pep1 fragments
pep2 fragments
fragments with xl arm
AAFTECCQAADKAACLLPK (Albumin)
VFDEFKPLVEEPQNLIK (Albumin)
MS2
DIA
a
1e4 AAFTECCQAADKAACLLPK(12)--VFDEFKPLVEEPQNLIK(6).-
intensity
m/z
b
intensity
rt
MS1
c
C:\Users\...\MS_DATA\Lotta_S1605_083
05/10/16 05:52:38
M1+albumin_250_DSS_1
RT: 71.07 - 71.81
NL: 6.10E6
m/z= 1077.39-1079.48
F: FTMS + p NSI Full ms [400.00-1600.00] MS
Lotta_S1605_083
NL: 6.67E6
m/z= 1080.23-1082.23
F: FTMS + p NSI Full ms [400.00-1600.00] MS
Lotta_S1605_083
Relative Abundance
Time (min)
Lotta_S1605_083#35238-35337 RT: 71.32-71.49 AV: 7 NL: 1.03E6
T: FTMS + p NSI Full ms [400.00-1600.00]
Relative Abundance
m/z

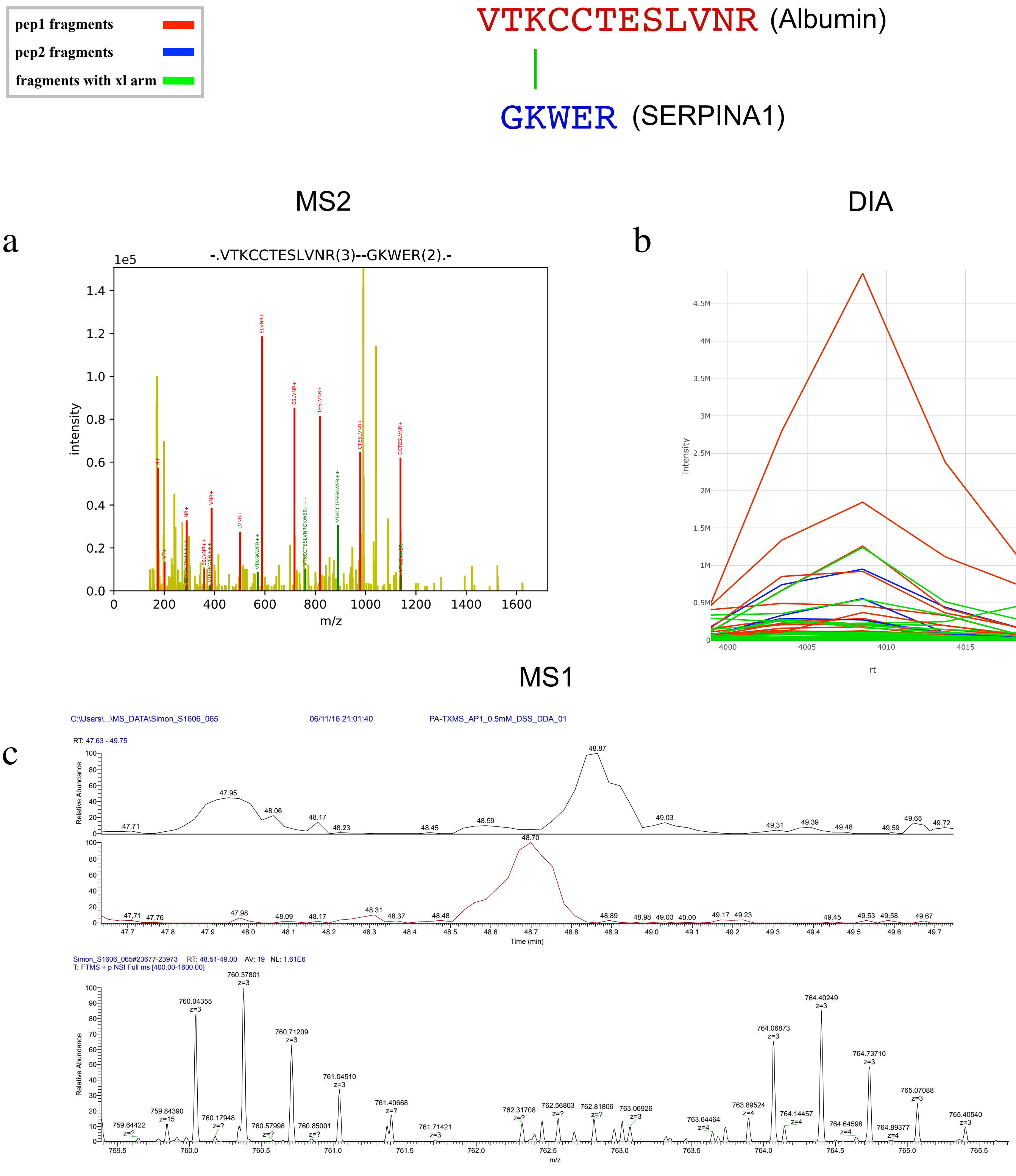

pep1 fragments
pep2 fragments
fragments with xl arm
VTKCCTESLVNR (Albumin)
GKWER (SERPINA1)
MS2
DIA
a
-.VTKCCTESLVNR(3)--GKWER(2).-
1e5
intensity
m/z
SLVNR+
ESLVNR+
TESLVNR+
CTESLVNR+
CCTESLVNR+
VNR+
NR+
LVNR+
VTKCCTESGKWER++
b
intensity
rt
MS1
c
C:\Users\...\MS_DATA\Simon_S1606_065
06/11/16 21:01:40
PA-TXMS_AP1_0.5mM_DSS_DDA_01
RT: 47.63 - 49.75
Relative Abundance
Time (min)
Simon_S1606_065#23677-23973 RT: 48.51-49.00 AV: 19 NL: 1.61E6
T: FTMS + p NSI Full ms [400.00-1600.00]
m/z

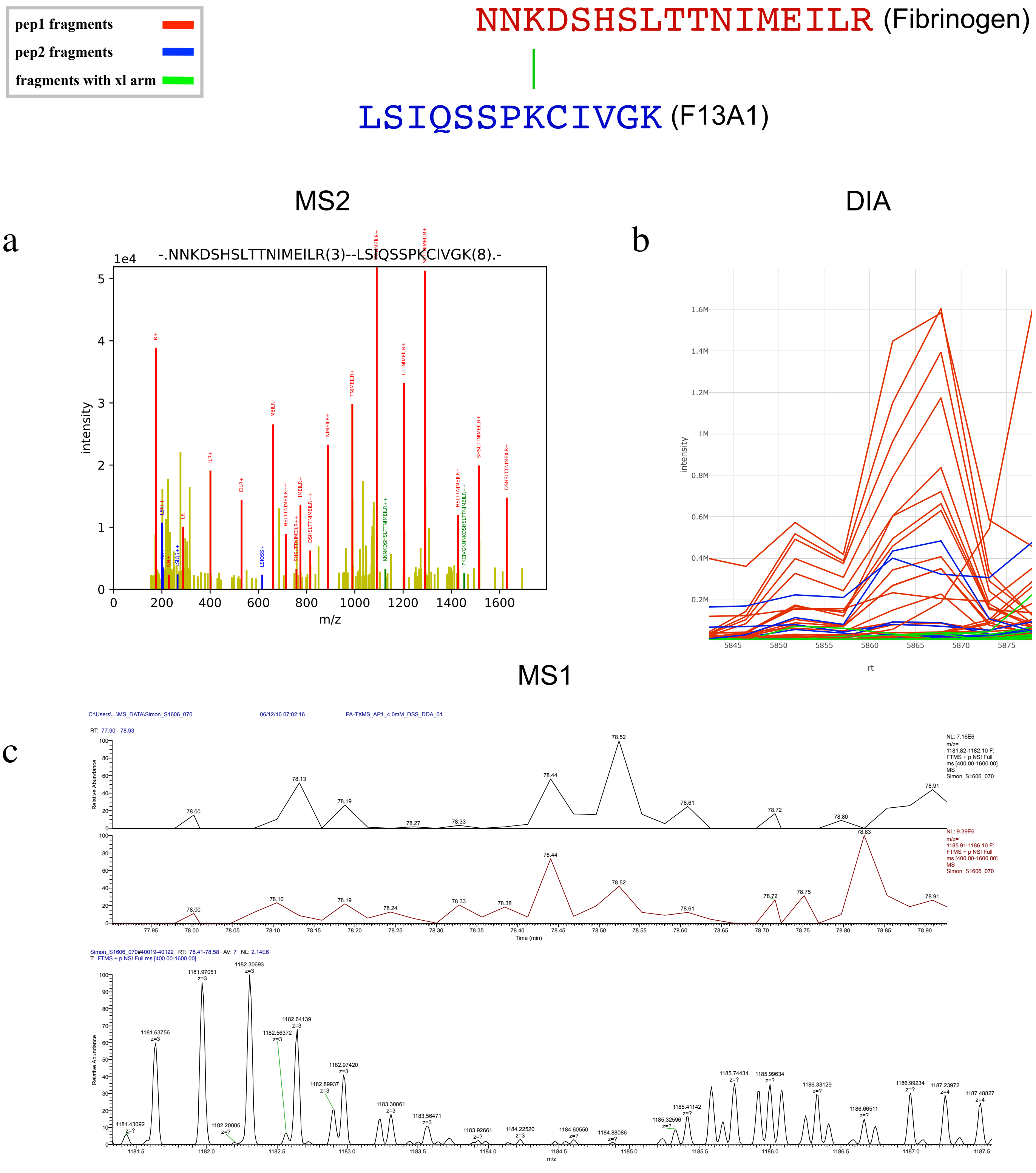

pep1 fragments
pep2 fragments
fragments with xl arm
NNKDSHSLTTNIMEILR (Fibrinogen)
LSIQSSPKCIVGK (F13A1)
MS2
DIA
a
-.NNKDSHSLTTNIMEILR(3)--LSIQSSPKCIVGK(8).-
1e4
intensity
m/z
b
intensity
rt
MS1
c
C:\Users\...\MS_DATA\Simon_S1606_070
06/12/16 07:02:16
PA-TXMS_AP1_4.0mM_DSS_DDA_01
RT: 77.90 - 78.93
Relative Abundance
Time (min)
Simon_S1606_070#40019-40122 RT: 78.41-78.58 AV: 7 NL: 2.14E6
T: FTMS + p NSI Full ms [400.00-1600.00]
m/z

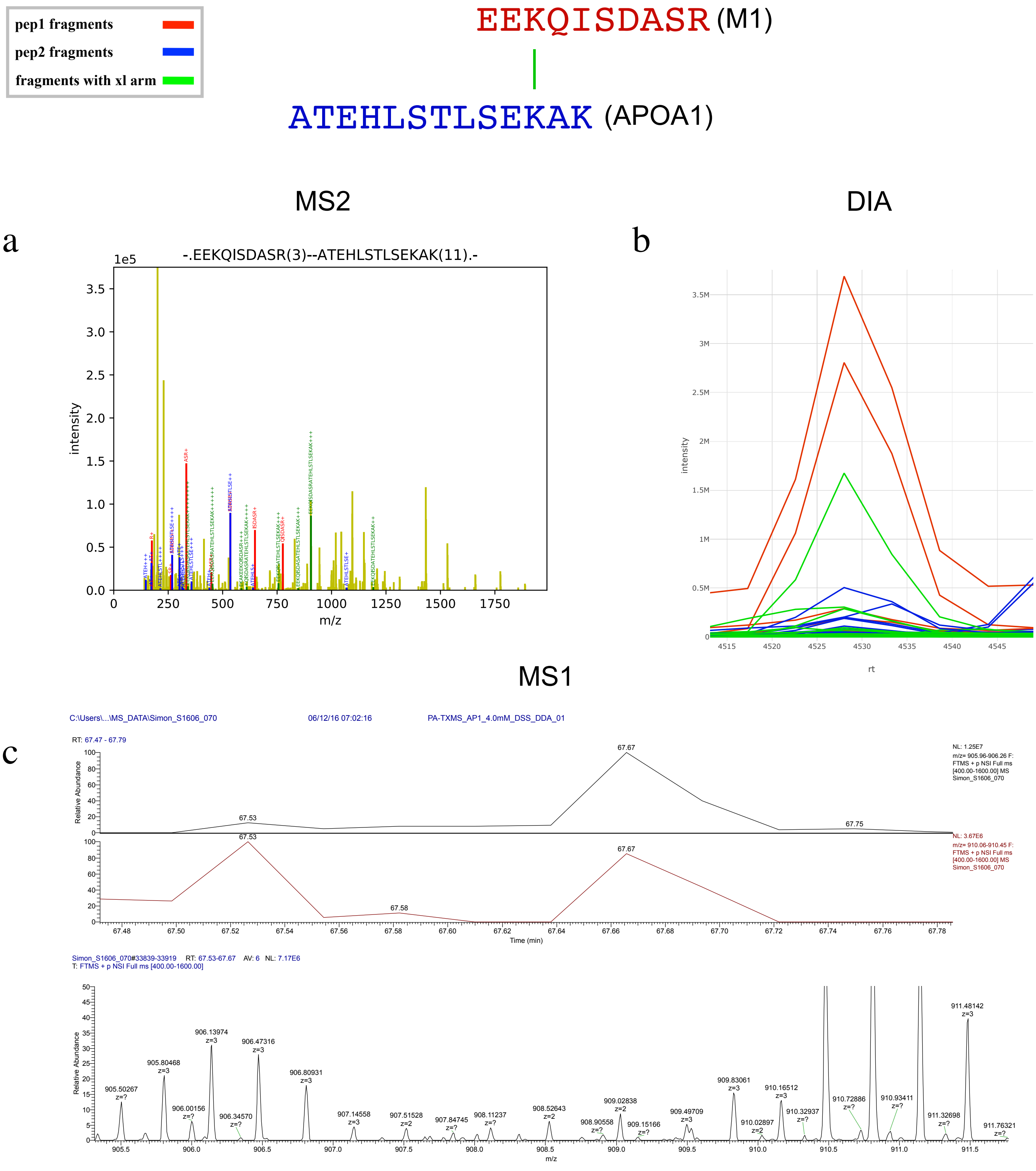
pep1 fragments
pep2 fragments
fragments with xl arm
EEKQISDASR (M1)
ATEHLSTLSEKAK (APOA1)
MS2
DIA
a
-.EEKQISDASR(3)--ATEHLSTLSEKAK(11).-
intensity
m/z
b
intensity
rt
MS1
c
C:\Users\...\MS_DATA\Simon_S1606_070
06/12/16 07:02:16
PA-TXMS_AP1_4.0mM_DSS_DDA_01
RT: 67.47 - 67.79
Relative Abundance
Time (min)
Simon_S1606_070#33839-33919 RT: 67.53-67.67 AV: 6 NL: 7.17E6
T: FTMS + p NSI Full ms [400.00-1600.00]
Relative Abundance
m/z

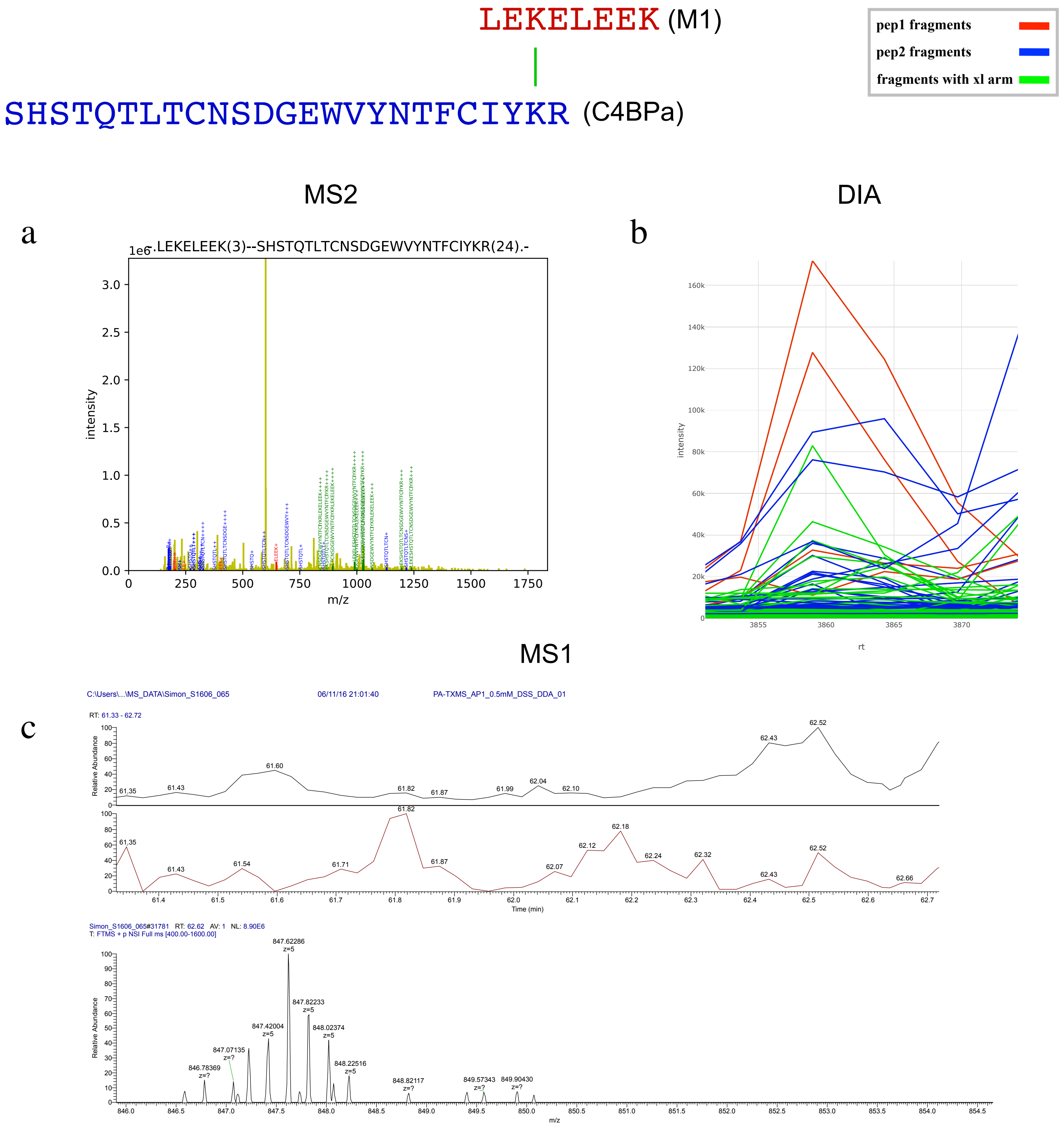
LEKELEEK (M1)
SHSTQTLTCNSDGEWVYNTFCIYKR (C4BPa)
pep1 fragments
pep2 fragments
fragments with xl arm
MS2
DIA
a
.LEKELEEK(3)--SHSTQTLTCNSDGEWVYNTFCIYKR(24).-
intensity
m/z
b
intensity
rt
MS1
c
C:\Users\...\MS_DATA\Simon_S1606_065
06/11/16 21:01:40
PA-TXMS_AP1_0.5mM_DSS_DDA_01
RT: 61.33 - 62.72
Relative Abundance
Time (min)
Simon_S1606_065#31781 RT: 62.62 AV: 1 NL: 8.90E6
T: FTMS + p NSI Full ms [400.00-1600.00]
847.62286 z=5
847.82233 z=5
847.42004 z=5
848.02374 z=5
847.07135 z=?
846.78369 z=?
848.22516 z=5
848.82117 z=?
849.57343 z=?
849.90430 z=?
m/z

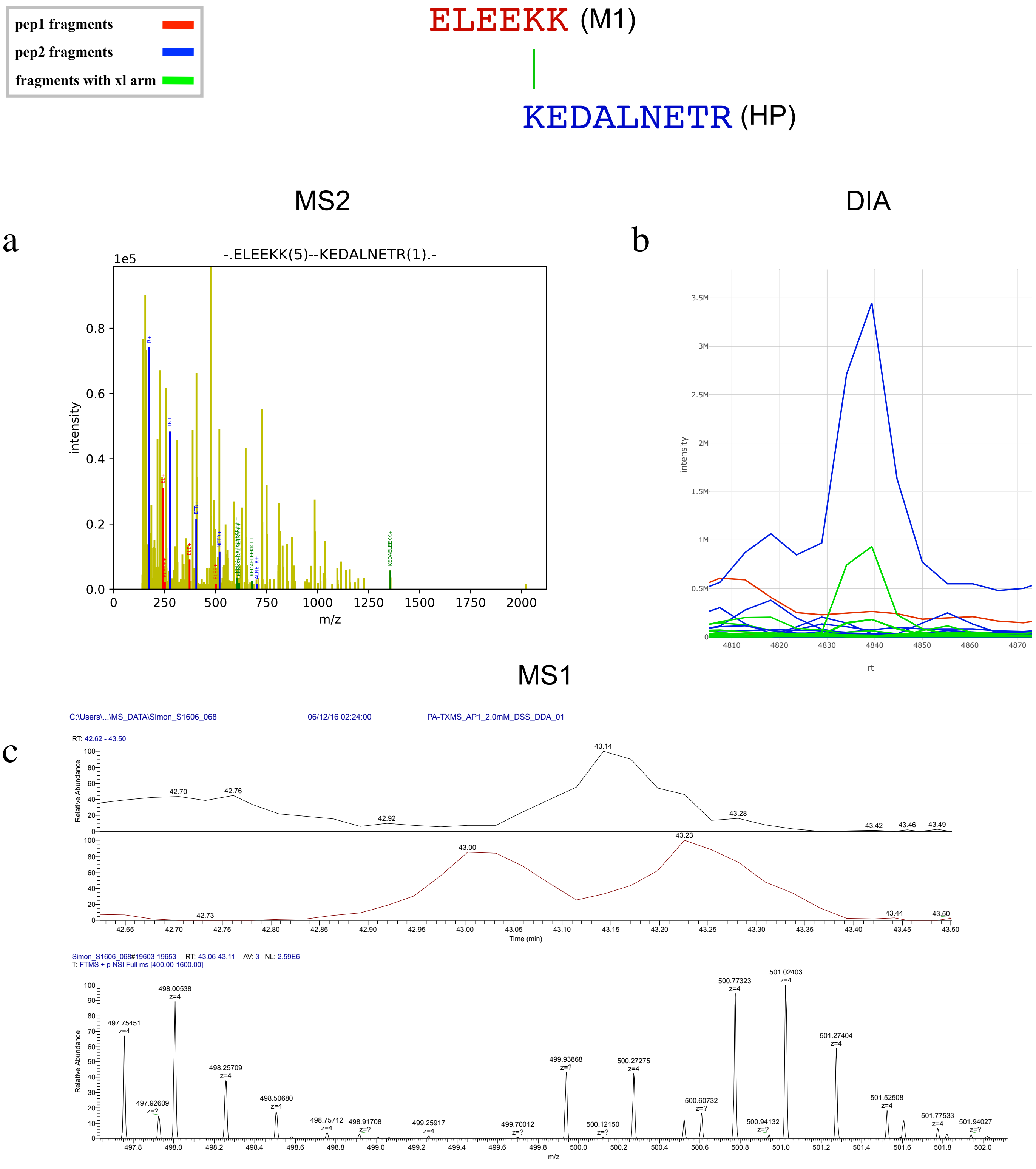
pep1 fragments
pep2 fragments
fragments with xl arm
ELEEKK (M1)
KEDALNETR (HP)
MS2
DIA
a
-.ELEEKK(5)--KEDALNETR(1).-
1e5
intensity
m/z
b
intensity
rt
MS1
c
C:\Users\...\MS_DATA\Simon_S1606_068
06/12/16 02:24:00
PA-TXMS_AP1_2.0mM_DSS_DDA_01
RT: 42.62 - 43.50
Relative Abundance
Time (min)
Simon_S1606_068#19603-19653 RT: 43.06-43.11 AV: 3 NL: 2.59E6
T: FTMS + p NSI Full ms [400.00-1600.00]
Relative Abundance
m/z

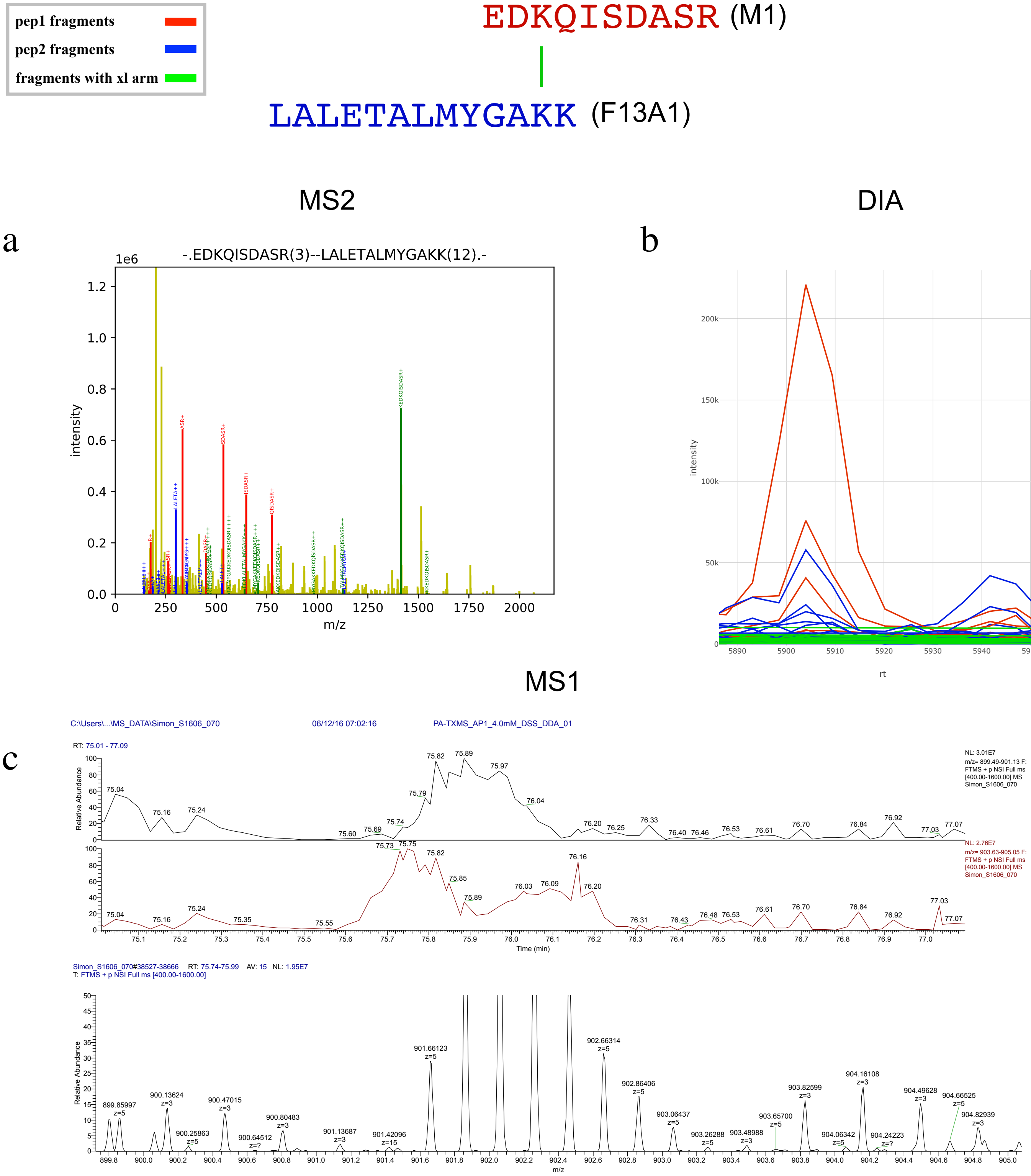
pep1 fragments
pep2 fragments
fragments with xl arm
EDKQISDASR (M1)
LALETALMYGAKK (F13A1)
MS2
DIA
a
-.EDKQISDASR(3)--LALETALMYGAKK(12).-
intensity
m/z
b
intensity
rt
MS1
c
C:\Users\...\MS_DATA\Simon_S1606_070
06/12/16 07:02:16
PA-TXMS_AP1_4.0mM_DSS_DDA_01
RT: 75.01 - 77.09
Relative Abundance
Time (min)
Simon_S1606_070#38527-38666 RT: 75.74-75.99 AV: 15 NL: 1.95E7
T: FTMS + p NSI Full ms [400.00-1600.00]
m/z

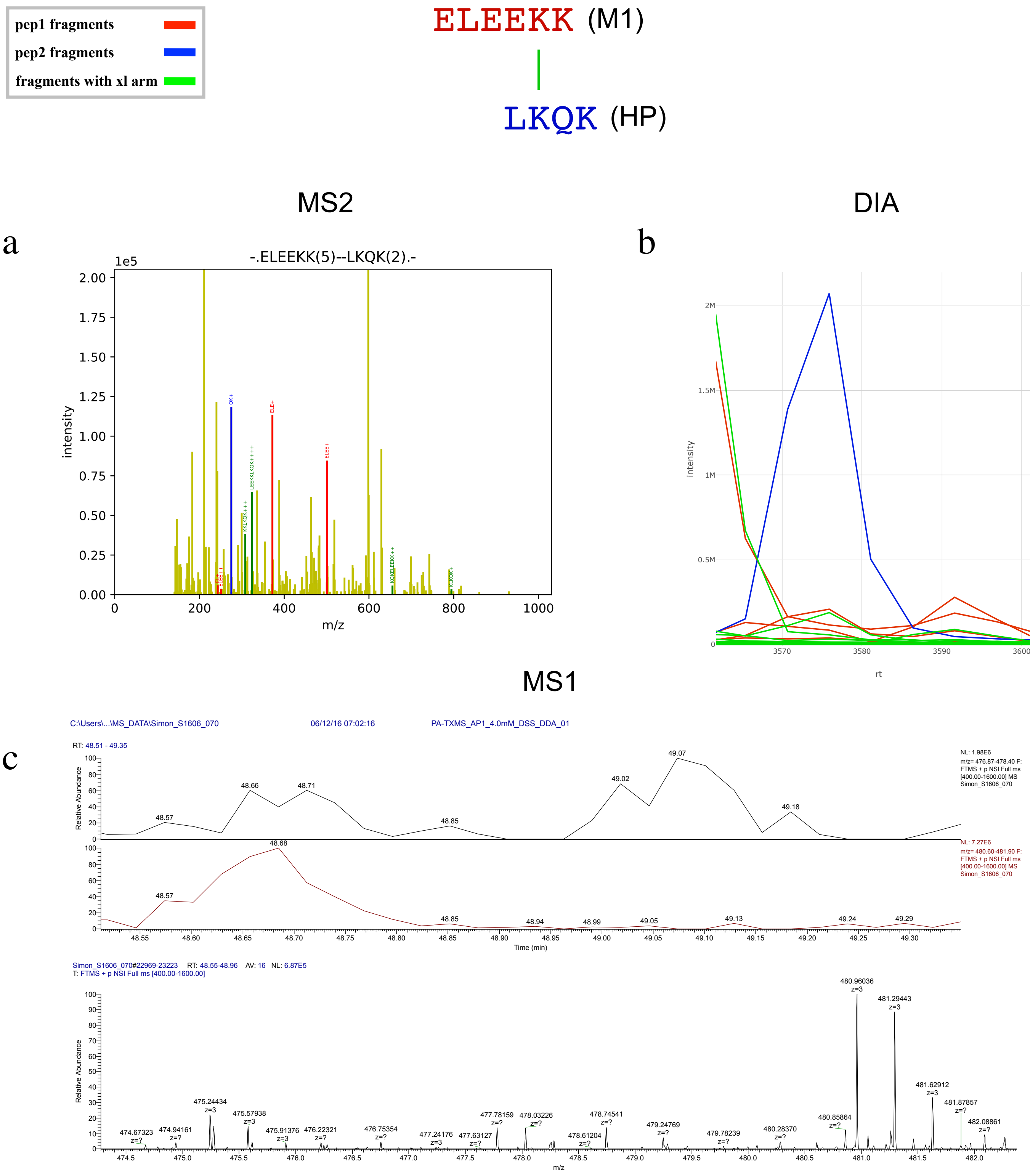

pep1 fragments
pep2 fragments
fragments with xl arm
ELEEKK (M1)
LKQK (HP)
MS2
DIA
a
-.ELEEKK(5)--LKQK(2).-
1e5
intensity
m/z
QK+
ELE+
ELEE+
LEEKKLKQK++++
KKLKQK+++
KQKELEEKK++
KLKQK+
b
intensity
rt
2M
1.5M
1M
0.5M
0
3570
3580
3590
3600
MS1
c
C:\Users\...\MS_DATA\Simon_S1606_070
06/12/16 07:02:16
PA-TXMS_AP1_4.0mM_DSS_DDA_01
RT: 48.51 - 49.35
Relative Abundance
NL: 1.98E6
m/z= 476.87-478.40 F: FTMS + p NSI Full ms [400.00-1600.00] MS Simon_S1606_070
NL: 7.27E6
m/z= 480.60-481.90 F: FTMS + p NSI Full ms [400.00-1600.00] MS Simon_S1606_070
Time (min)
Simon_S1606_070#22969-23223 RT: 48.55-48.96 AV: 16 NL: 6.87E5
T: FTMS + p NSI Full ms [400.00-1600.00]
480.96036 z=3
481.29443 z=3
481.62912 z=3
481.87857 z=?
482.08861 z=?
480.85864 z=?
480.28370 z=?
479.78239 z=?
479.24769 z=?
478.74541 z=?
478.61204 z=?
478.03226 z=?
477.78159 z=?
477.63127 z=?
477.24176 z=3
476.75354 z=?
476.22321 z=?
475.91376 z=3
475.57938 z=3
475.24434 z=3
474.94161 z=?
474.67323 z=?
m/z

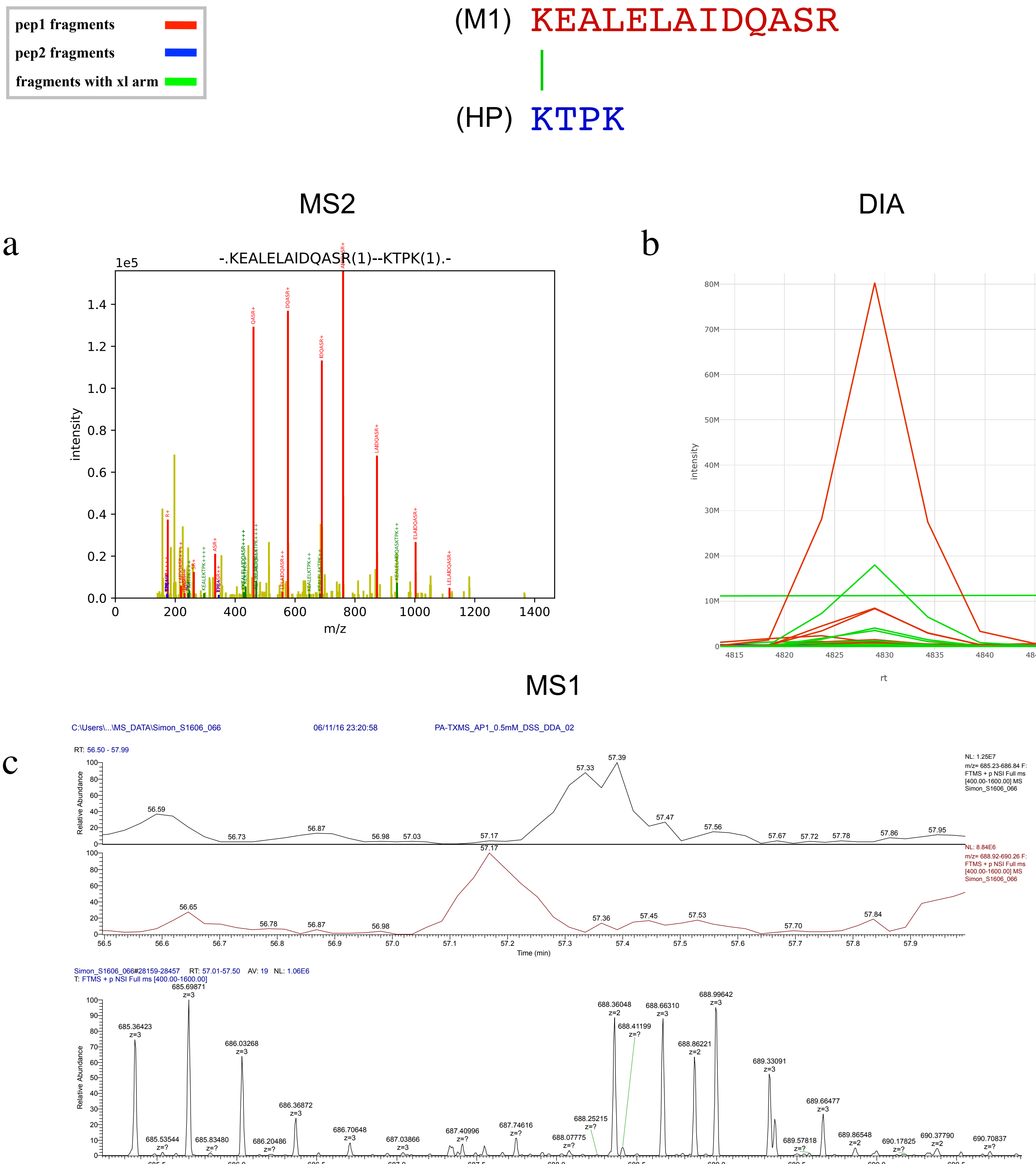

pep1 fragments
pep2 fragments
fragments with xl arm
(M1) KEALELAIDQASR
(HP) KTPK
MS2
DIA
a
-.KEALELAIDQASR(1)--KTPK(1).-
1e5
intensity
m/z
QASR+
DQASR+
IDQASR+
LAIDQASR+
ELAIDQASR+
LELAIDQASR+
ASR+
R+
b
intensity
rt
80M
70M
60M
50M
40M
30M
20M
10M
0
4815
4820
4825
4830
4835
4840
4845
MS1
c
C:\Users\...\MS_DATA\Simon_S1606_066
06/11/16 23:20:58
PA-TXMS_AP1_0.5mM_DSS_DDA_02
RT: 56.50 - 57.99
NL: 1.25E7
m/z= 685.23-686.84 F: FTMS + p NSI Full ms [400.00-1600.00] MS Simon_S1606_066
NL: 8.84E6
m/z= 688.92-690.26 F: FTMS + p NSI Full ms [400.00-1600.00] MS Simon_S1606_066
Relative Abundance
Time (min)
Simon_S1606_066#28159-28457 RT: 57.01-57.50 AV: 19 NL: 1.06E6
T: FTMS + p NSI Full ms [400.00-1600.00]
685.36423 z=3
685.69871 z=3
686.03268 z=3
686.36872 z=3
686.70648 z=3
687.03866 z=3
687.40996 z=?
687.74616 z=?
688.07775 z=?
688.25215 z=?
688.36048 z=2
688.41199 z=?
688.66310 z=3
688.86221 z=2
688.99642 z=3
689.33091 z=3
689.57818 z=?
689.66477 z=3
689.86548 z=2
690.17825 z=?
690.37790 z=2
690.70837 z=?
m/z

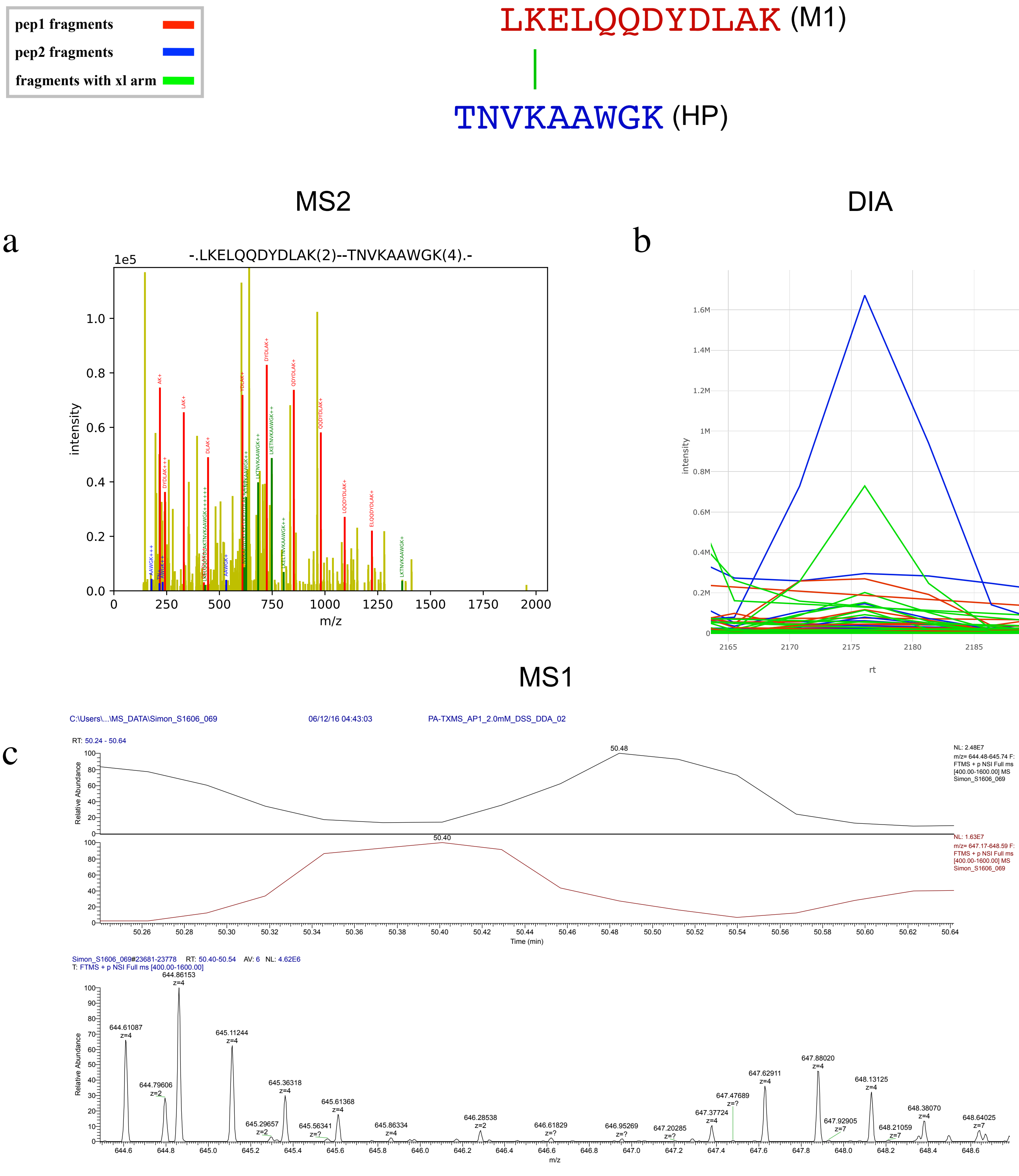
pep1 fragments
pep2 fragments
fragments with xl arm
LKELQQDYDLAK (M1)
TNVKAAWGK (HP)
MS2
DIA
a
b
-.LKELQQDYDLAK(2)--TNVKAAWGK(4).-
1e5
intensity
m/z
intensity
rt
MS1
c
C:\Users\...\MS_DATA\Simon_S1606_069
06/12/16 04:43:03
PA-TXMS_AP1_2.0mM_DSS_DDA_02
RT: 50.24 - 50.64
Relative Abundance
NL: 2.48E7
m/z= 644.48-645.74 F: FTMS + p NSI Full ms [400.00-1600.00] MS Simon_S1606_069
NL: 1.63E7
m/z= 647.17-648.59 F: FTMS + p NSI Full ms [400.00-1600.00] MS Simon_S1606_069
Time (min)
Simon_S1606_069#23681-23778 RT: 50.40-50.54 AV: 6 NL: 4.62E6
T: FTMS + p NSI Full ms [400.00-1600.00]
Relative Abundance
m/z

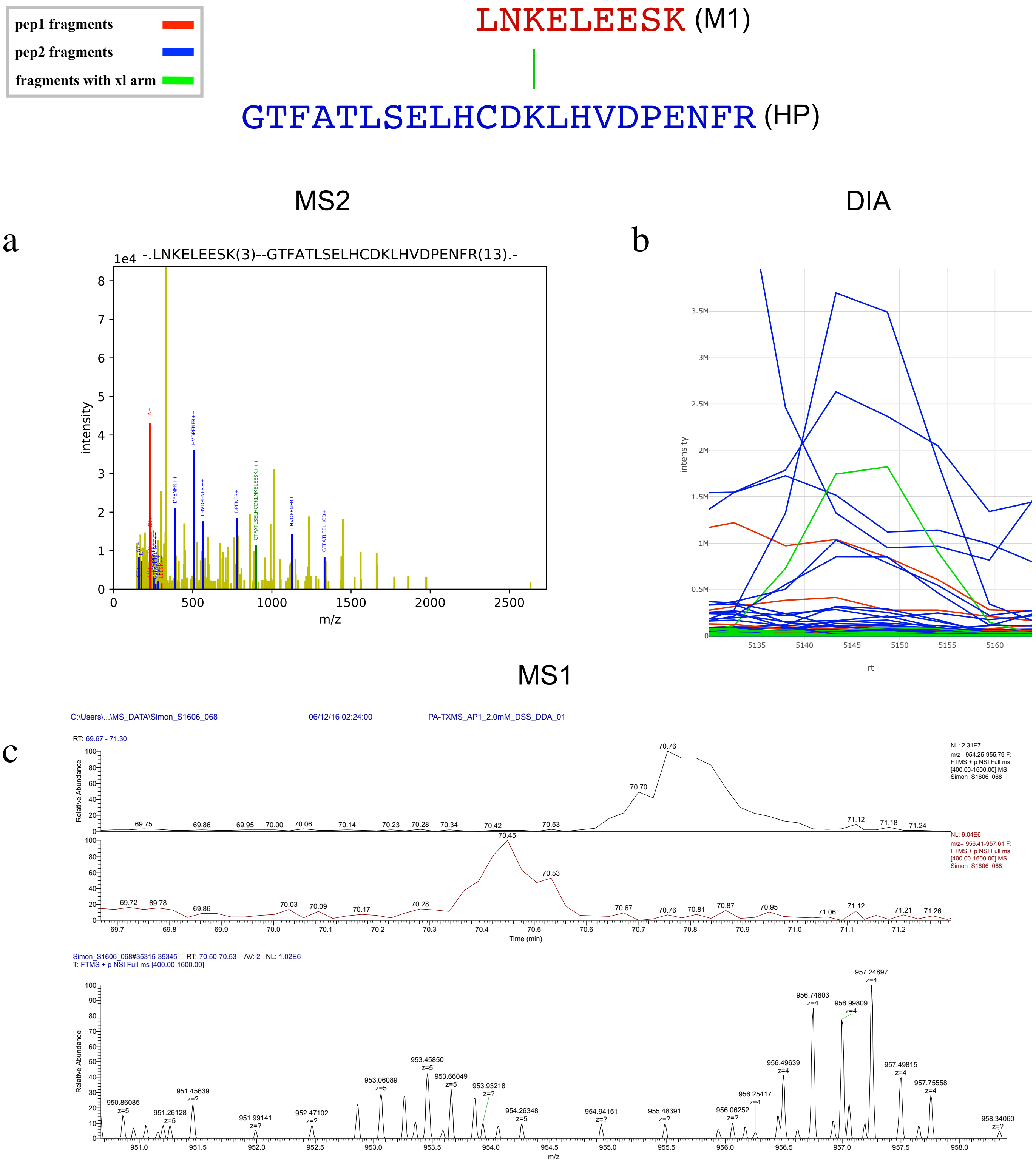
pep1 fragments
pep2 fragments
fragments with xl arm
LNKELEESK (M1)
GTFATLSELHCDKLHVDPENFR (HP)
MS2
DIA
a
-.LNKELEESK(3)--GTFATLSELHCDKLHVDPENFR(13).-
1e4
intensity
m/z
LN+
HVDPENFR++
DPENFR++
LHVDPENFR++
DPENFR+
GTFATLSELHCDKLNKELEESK+++
LHVDPENFR+
GTFATLSELHCD+
b
intensity
rt
MS1
c
C:\Users\...\MS_DATA\Simon_S1606_068
06/12/16 02:24:00
PA-TXMS_AP1_2.0mM_DSS_DDA_01
RT: 69.67 - 71.30
Relative Abundance
NL: 2.31E7
m/z= 954.25-955.79 F: FTMS + p NSI Full ms [400.00-1600.00] MS Simon_S1606_068
NL: 9.04E6
m/z= 956.41-957.61 F: FTMS + p NSI Full ms [400.00-1600.00] MS Simon_S1606_068
Time (min)
Simon_S1606_068#35315-35345 RT: 70.50-70.53 AV: 2 NL: 1.02E6
T: FTMS + p NSI Full ms [400.00-1600.00]
Relative Abundance
m/z

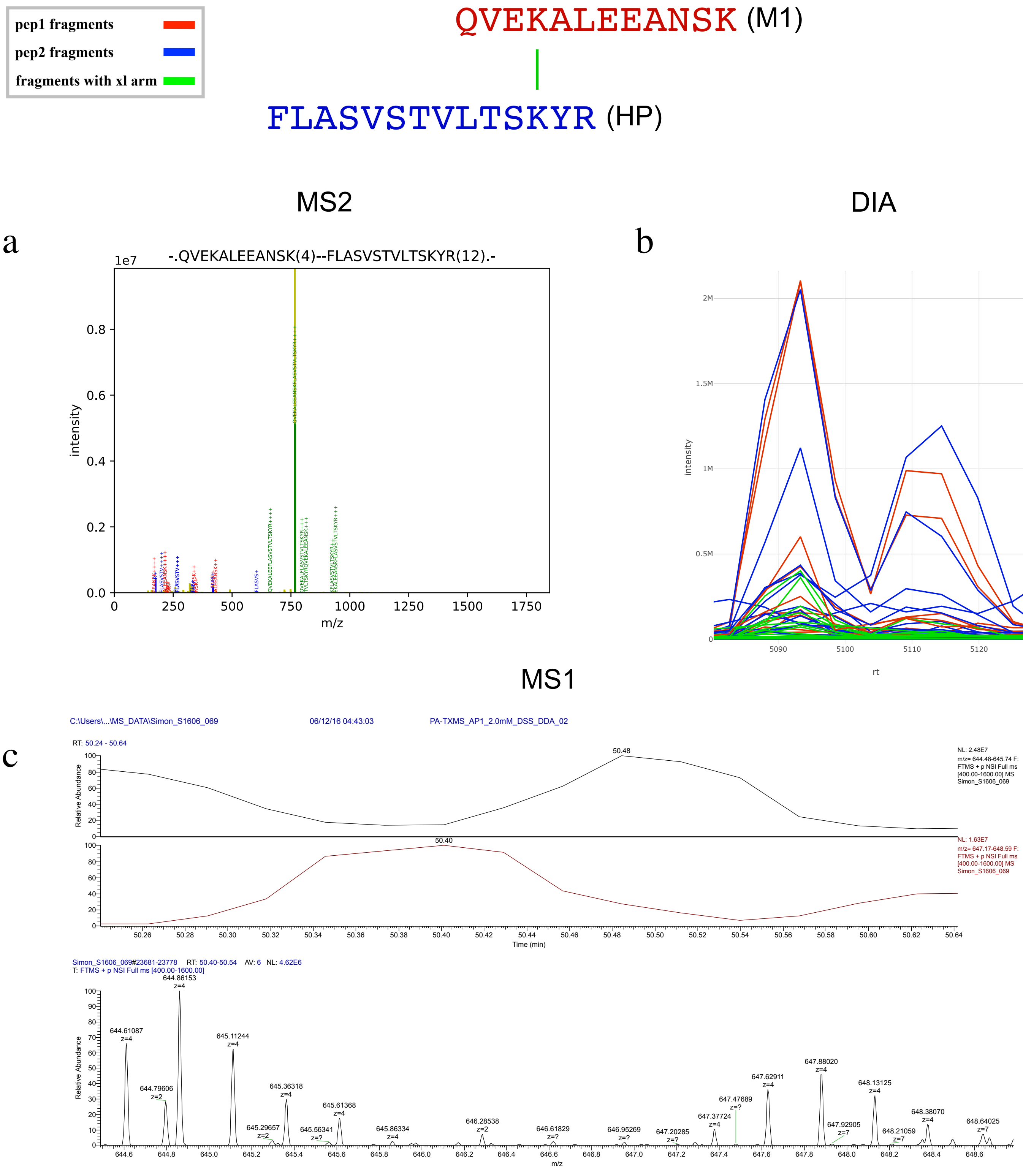
pep1 fragments
pep2 fragments
fragments with xl arm
QVEKALEEANSK (M1)
FLASVSTVLTSKYR (HP)
MS2
DIA
a
-.QVEKALEEANSK(4)--FLASVSTVLTSKYR(12).-
1e7
intensity
m/z
b
intensity
rt
MS1
c
C:\Users\...\MS_DATA\Simon_S1606_069
06/12/16 04:43:03
PA-TXMS_AP1_2.0mM_DSS_DDA_02
RT: 50.24 - 50.64
NL: 2.48E7
m/z= 644.48-645.74 F: FTMS + p NSI Full ms [400.00-1600.00] MS Simon_S1606_069
NL: 1.63E7
m/z= 647.17-648.59 F: FTMS + p NSI Full ms [400.00-1600.00] MS Simon_S1606_069
Relative Abundance
Time (min)
Simon_S1606_069#23681-23778 RT: 50.40-50.54 AV: 6 NL: 4.62E6
T: FTMS + p NSI Full ms [400.00-1600.00]
Relative Abundance
m/z

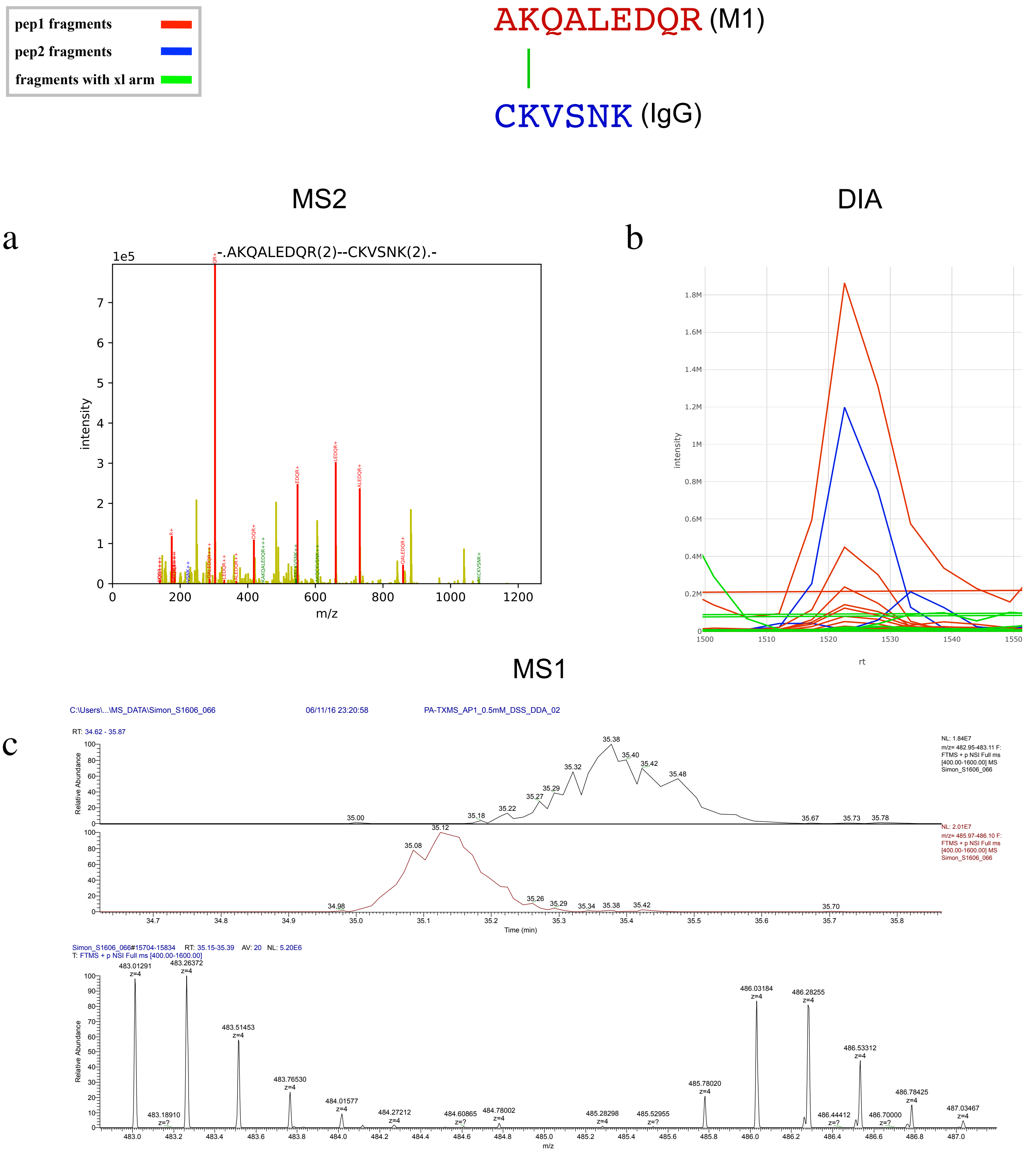
pep1 fragments
pep2 fragments
fragments with xl arm
AKQALEDQR (M1)
CKVSNK (IgG)
MS2
DIA
a
-.AKQALEDQR(2)--CKVSNK(2).-
1e5
intensity
m/z
b
intensity
rt
MS1
c
C:\Users\...\MS_DATA\Simon_S1606_066
06/11/16 23:20:58
PA-TXMS_AP1_0.5mM_DSS_DDA_02
RT: 34.62 - 35.87
NL: 1.84E7
m/z= 482.95-483.11 F: FTMS + p NSI Full ms [400.00-1600.00] MS Simon_S1606_066
NL: 2.01E7
m/z= 485.97-486.10 F: FTMS + p NSI Full ms [400.00-1600.00] MS Simon_S1606_066
Relative Abundance
Time (min)
Simon_S1606_066#15704-15834 RT: 35.15-35.39 AV: 20 NL: 5.20E6
T: FTMS + p NSI Full ms [400.00-1600.00]
Relative Abundance
m/z
