# Supplementary material for: Rapid determination of quaternary protein structures in complex biological samples
Source: Nat Commun. 2019 Jan 14;10:192. doi: 10.1038/s41467-018-07986-1 (PMC6331586; doi:10.1038/s41467-018-07986-1)
Supplement: Supplementary file 4 — Reporting Summary [file 41467_2018_7986_MOESM4_ESM.pdf]

nature research

Corresponding author(s): Lars Malmström and Johan Malmström

Reporting Summary

Nature Research wishes to improve the reproducibility of the work that we publish. This form provides structure for consistency and transparency in reporting. For further information on Nature Research policies, see Authors & Referees and the Editorial Policy Checklist.

Statistical parameters

When statistical analyses are reported, confirm that the following items are present in the relevant location (e.g. figure legend, table legend, main text, or Methods section).

n/a	Confirmed	
☐	☒	The exact sample size (n) for each experimental group/condition, given as a discrete number and unit of measurement
☐	☒	An indication of whether measurements were taken from distinct samples or whether the same sample was measured repeatedly
☐	☒	The statistical test(s) used AND whether they are one- or two-sided<br>*Only common tests should be described solely by name; describe more complex techniques in the Methods section.*
☒	☐	A description of all covariates tested
☒	☐	A description of any assumptions or corrections, such as tests of normality and adjustment for multiple comparisons
☒	☐	A full description of the statistics including central tendency (e.g. means) or other basic estimates (e.g. regression coefficient) AND variation (e.g. standard deviation) or associated estimates of uncertainty (e.g. confidence intervals)
☒	☐	For null hypothesis testing, the test statistic (e.g. F, t, r) with confidence intervals, effect sizes, degrees of freedom and P value noted<br>*Give P values as exact values whenever suitable.*
☒	☐	For Bayesian analysis, information on the choice of priors and Markov chain Monte Carlo settings
☒	☐	For hierarchical and complex designs, identification of the appropriate level for tests and full reporting of outcomes
☒	☐	Estimates of effect sizes (e.g. Cohen's d, Pearson's r), indicating how they were calculated
☒	☐	Clearly defined error bars<br>*State explicitly what error bars represent (e.g. SD, SE, CI)*

Our web collection on statistics for biologists may be useful.

Software and code

Policy information about availability of computer code

Data collection	All software available in the form of singularity containers. Access to containers are available in the manuscript. Rosetta was excluded from the containers but it is easily available to all academics.
Data analysis	All analysis carried out using Jupyterhub, an open-source and free technology.

For manuscripts utilizing custom algorithms or software that are central to the research but not yet described in published literature, software must be made available to editors/reviewers upon request. We strongly encourage code deposition in a community repository (e.g. GitHub). See the Nature Research guidelines for submitting code & software for further information.

Data

Policy information about availability of data

All manuscripts must include a data availability statement. This statement should provide the following information, where applicable:

- Accession codes, unique identifiers, or web links for publicly available datasets
- A list of figures that have associated raw data
- A description of any restrictions on data availability

Data is partly available in public repositories (zenodo.org, described in the manuscript) and partly directly from University of Zurich (described in manuscript)

Field-specific reporting

Please select the best fit for your research. If you are not sure, read the appropriate sections before making your selection.

- [x] Life sciences
- [ ] Behavioural & social sciences
- [ ] Ecological, evolutionary & environmental sciences

For a reference copy of the document with all sections, see nature.com/authors/policies/ReportingSummary-flat.pdf

Life sciences study design

All studies must disclose on these points even when the disclosure is negative.

Sample size	This is not a quantitative study;
Data exclusions	No data was excluded.
Replication	No experiments were repeated.
Randomization	Not a quantitative study, no randomization needed.
Blinding	No group allocation.

Reporting for specific materials, systems and methods

Materials & experimental systems

n/a	Involved in the study	
[x]	[ ]	Unique biological materials
[ ]	[x]	Antibodies
[x]	[ ]	Eukaryotic cell lines
[x]	[ ]	Palaeontology
[ ]	[x]	Animals and other organisms
[x]	[ ]	Human research participants

Methods

n/a	Involved in the study	
[x]	[ ]	ChIP-seq
[x]	[ ]	Flow cytometry
[x]	[ ]	MRI-based neuroimaging

Antibodies

Antibodies used	Only antibodies as part of the human plasma (available from a commercial source)
Validation	See above.

Animals and other organisms

Policy information about studies involving animals; ARRIVE guidelines recommended for reporting animal research

Laboratory animals	Streptococcus pyogenes, SF370 (obviously not an animal, but it asked for Animals or other organisms above)
Wild animals	The study did not involve wild animals.
Field-collected samples	The study did not involve samples colected from the field.
